# Planting the Seeds of a Decision Tree for Ionic Liquids: Steric and Electronic Impacts on Melting Points of Triarylphosponium Ionic Liquids

**DOI:** 10.1021/acs.jpcb.4c02196

**Published:** 2024-06-07

**Authors:** Marija Scheuren, Lara Teodoro, Andrew Witters, Muhammadiqboli Musozoda, Clinton Adu, Gary Guillet, Ronald Freeze, Matthias Zeller, Arsalan Mirjafari, Patrick C. Hillesheim

**Affiliations:** † Department of Chemistry and Physics, Ave Maria University, Ave Maria, Florida 34142, United States; ‡ Department of Chemistry, State University of New York at Oswego, Oswego, New York 13126, United States; § Department of Chemistry, Furman University, Greenville, South Carolina 29613, United States; ∥ Department of Chemistry, Purdue University, West Lafayette, Indiana 47907, United States

## Abstract

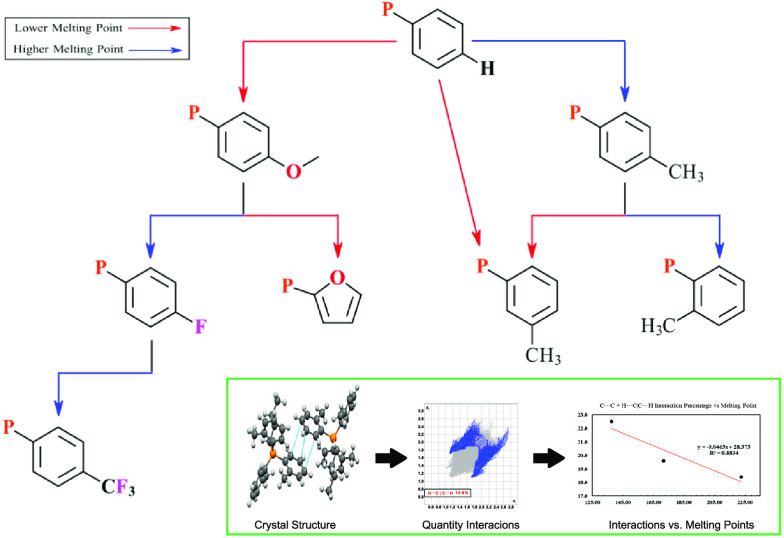

While machine learning and artificial intelligence offer promising avenues in the computer-aided design of materials, the complexity of these computational techniques remains a barrier for scientists outside of the specific fields of study. Leveraging decision tree models, inspired by empirical methodologies, offers a pragmatic solution to the knowledge barrier presented by artificial intelligence (AI). Herein, we present a model allowing for the qualitative prediction of melting points of ionic liquids derived from the crystallographic analysis of a series of phosphonium-based ionic liquids. By carefully tailoring the steric and electronic properties of the cations within these salts, trends in the melting points are observed, pointing toward the critical importance of π interactions to forming the solid state. Quantification of the percentage of these π interactions using modern quantum crystallographic approaches reveals a linear trend in the relationship of C–Hπ and π–π stacking interactions with melting points. These structure–property relationships are further examined by using computational studies, helping to demonstrate the inverse relationship of dipole moments and melting points for ionic liquids. The results provide valuable insights into the features and relationships that are consistent with achieving low *T*_m_ values in phosphonium salts, which were not apparent in earlier studies. The data gathered are presented in a simple decision tree format, allowing for visualization of the data and providing guidance toward developing yet unreported compounds.

## 1 Introduction

Ionic liquids (ILs) are a topic of significant interest among academic and industrial chemists alike due to their unique properties and applications.^1^ One fundamental principle in designing an IL involves finding molecular characteristics that prevent or hinder crystallization. This entails selecting asymmetrical ions, promoting charge delocalization, and minimizing adhesive interactions by avoiding established noncovalent interaction (NCI) synthons.^2,3^ These design principles are, in essence, anticrystal engineering strategies.^4^ Leveraging these principles has proven to be quite straightforward, and by doing so, we gained insights into IL structure–property relationships that add to and deepen those uncovered by our earlier works.^5^ However, the inherent complexity, structural diversity, and the presence of impurities (e.g., water and alkali salts) within ILs represent a fundamental obstacle to the accurate determination of their physiochemical properties.

Predicting the melting points (*T*_m_) of ILs formed from novel combinations of cations and anions remains challenging as each component can introduce complexities unique to the inherent composition of the ions. As a result, there is an unmet need to develop systematic methods for selecting new ion pairs, with the aim of minimizing cost and duration required to experimentally test different combinations of ions. These methods should be used as a predictive tool when designing novel and useful IL platforms. This topic has been at the forefront of ongoing research within the field, extensively covered in many articles.^6,7^ However, the paradigm for the development of an effective approach for accurately predicating the properties of ILs still remains the incorporation of new molecular design elements into materials as part of an iterative, linear process—an effective, albeit slow, approach to the discovery of new task-specific ILs.

Over the past 30 years of modern IL research, a tremendous wealth of data has been aggregated, particularly with respect to fundamental thermal properties such as decomposition temperatures and phase transitions.^8^ More recent approaches using machine learning (ML) or artificial intelligence (AI) have attempted to process these data to develop predictive models useful for the selection of the “optimal” IL for a specific purpose.^9^ However, the algorithms used in the development of these models remain complex, needing specialization in computational programming along with time to process and develop the learning models required. While the use of AI and ML will undoubtedly become simpler with time, concepts such as decision trees derived from empirical data have long existed prior to modern computational techniques.^10^ One such example of a decision tree is the Topliss tree,^11^ which offers a straightforward diagram simplifying decisions with respect to the design of potential pharmaceutical drugs with tailored biological activity. Likewise, our overall objective is to systematically create a decision tree to help tailoring the melting points of ILs with triarylphosphonium cations, with the objective of providing guidance toward the future development of new compounds without the complexities associated with modern machine learning approaches.

Several predictive modeling studies of ILs, particularly with respect to the prediction of melting points, have been reported in the literature. Recently, with the growing interest in AI and machine learning, numerous papers have emerged that develop various models for this purpose.^12^ These AI models have shown promise in potentially accelerating the development of novel ILs with tailored properties, thanks to the ability to rapidly process large volumes of data.^9^ However, within the studies, most authors particularly note that additional experimental data would help validate the models they developed as part of their studies.^13^ Crystallography has also played a key role in the development of predictive models, with one study focusing on the prediction of lattice energy of ILs.^14^ Within their study, Preiss et al. developed a model based on interaction percentages derived from Hirshfeld surfaces. To the best of our knowledge, this study remains one of the few reports that specifically leverages crystallography and Hirshfeld surface analysis to develop predictive models for ILs.

X-ray crystallography is a potent technique to decipher the intricate spatial relationships between cations and anions through the elucidation of crystal structures. It can thus provide a robust framework from which structural features of the ILs can be understood.^15^ To the point, crystallography can enhance the understanding of the physicochemical structure–property relationships in ILs by providing a clear picture into the NCIs present within the solid-state of the IL. It should be noted that the Coulombic interactions present in ILs are the largest contributors to the lattice energy of these organic salts.^16^ However, despite this dominant Coulombic contribution, the NCIs present within the ILs are observed to have a direct impact on thermophysical properties.^17^

To survey the properties of a specific class of materials, deliberate chemical modifications of the molecular structure of the ILs must be performed (e.g., methylation of the C2 position of imidazolium cations to prevent hydrogen bond formation).^18^ This strategy was applied to IL design previously and can be referred to as targeted modification.^19^ The concept could be further expanded to the broader idea of architectonics^20^ in that changes in the molecular structure of the ILs will affect both the intra- and intermolecular interactions and thus impact the aggregation behavior of the ILs, viz., crystallization. Using X-ray crystallography, targeted modifications (i.e., organic synthesis), spectroscopic techniques, theoretical studies (e.g., computational and quantum crystallography), and thermophysical analysis can all be combined and leveraged as powerful tools to probe the impact of molecular structure on the physicochemical properties of ILs.

**Figure 1:**
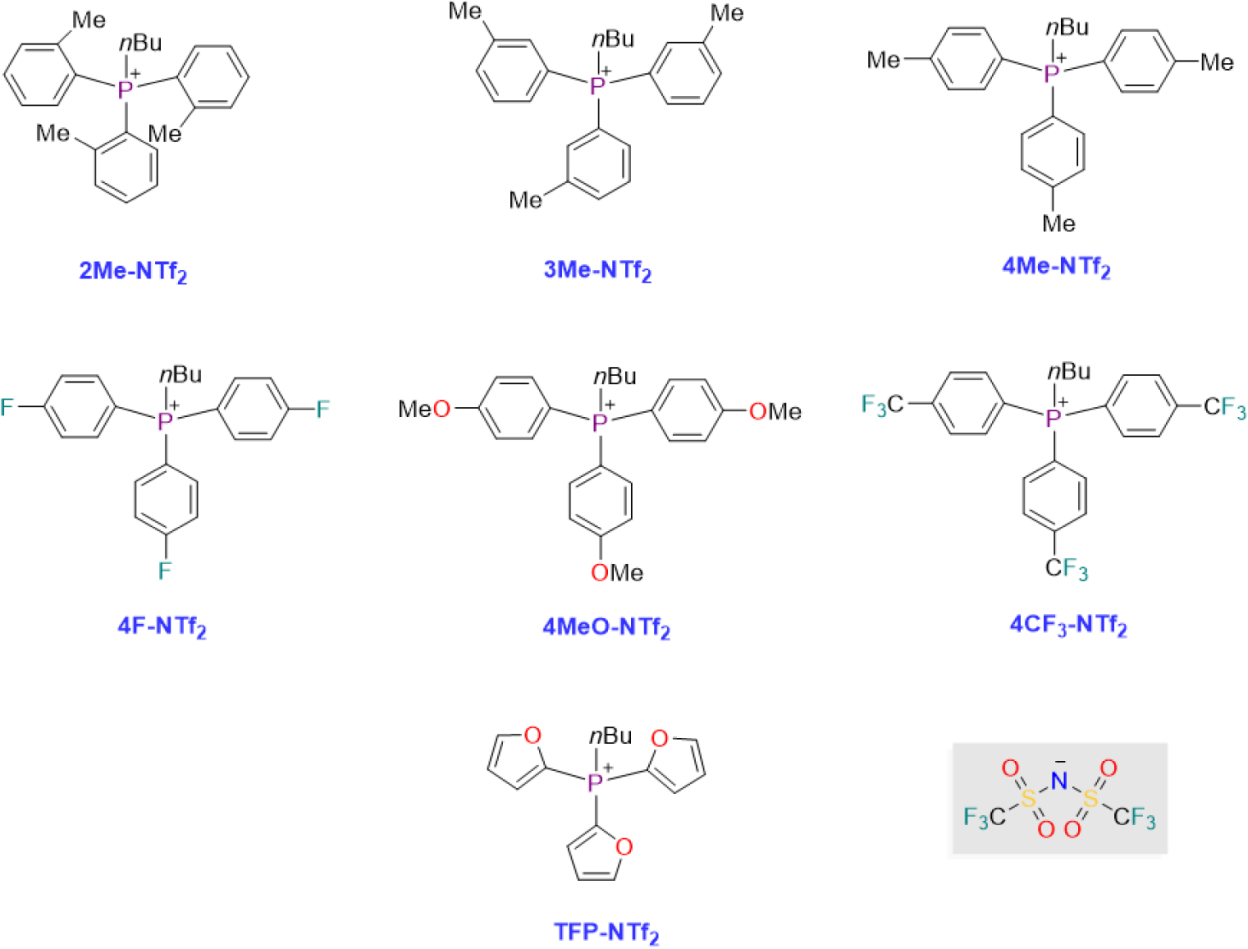
Depiction of the cations examined in this work. The NTf_2_ anion is shown in gray.

Herein, we present our initial decision tree—a sapling at this stage—for ILs bearing triarylphosphonium backbones. A series of seven steric and electronically varied phosphonium-based ILs are evaluated to draw out relevant structural principles (Figure 1). We reasoned that this approach offered an appealing framework for developing large libraries of structurally unique ILs for several reasons:(i) triarylphosphine derivatives are inexpensive and commercially available starting materials, (ii) the facile synthesis procedure allowed us to achieve extensive diversity, and (iii) purification steps are typically unnecessary as final IL products are readily crystallizable. Furthermore, the distinctive characteristics of the triarylphosphonium motifs, including their properties and potential applications as well as their dissimilarity to other commonly used structural components in ILs, make it an intriguing point of interest for further research.

We employed X-ray crystallography in combination with Hirshfeld surface analysis to comprehensively examine the intermolecular forces enabling quantification of the interactions with the π system of each molecule in the IL. Subsequently, we correlated the obtained structural data with the phase transitions of the ILs using thermophysical methods (differential scanning calorimetry, DSC, and thermogravimetric analysis, TGA). Finally, by integrating experimental data with computational analysis, we gained a deeper understanding of how the electronic properties of these ILs relate to their structure. Our studies found that there are predictable correlations between the NCIs observed in the crystal structures and the melting point of the ILs, specifically with those interactions involving the π systems. Following the discussion and rigorous analysis of these interactions, we present a simplified decision tree as a summary and visualization of the conclusions from our study.

## 2 Materials and Methods

Full synthetic procedures are provided in the Supporting Information.

### 2.1 Spectroscopy

^1^H, ^13^C, ^19^F, and ^31^P NMR spectroscopy was performed on a JEOL 400 MHz NMR. NMR solvents were purchased from Cambridge isotope laboratories. NMR shifts are referenced to the residual solvent peaks.

Mass spectra were recorded on an Agilent 1100 LC-MSD system. A 5 μL sample of the ionic liquids were injected into the system with a 10 mM formic acid in acetonitrile/water mobile phase with a linear gradient from 10% acetonitrile to 91% acetonitrile over 10 min and a flow rate of 1 mL/min.

### 2.2 Thermal Properties

Melting points, glass transitions, and crystallization temperatures were measured by using a PerkinElmer DSC 8000 differential scanning calorimeter (DSC). Each sample was placed in a crimped aluminum pan and cycled three consecutive times starting at −70 °C to the maximum temperature, which varied depending on the sample. The maximum temperature was set, depending on the melting point of the compound. The heating and cooling rates were 10 °C/min. A four-min isothermal step was included at the minimum and maximum temperatures of each cycle. All studies were completed under an atmosphere of nitrogen.

Decomposition temperatures were measured on a PerkinElmer 8000 thermogravimetric analyzer (TGA). A sample purge of nitrogen was used in all studies with a flow rate of 60 mL/min. Compounds were heated from 35 to 200 °C at a rate of 20 °C/min. The heating rate was slowed to 5 °C/min from 200 to 650 °C, which is the region wherein the largest mass loss is observed. After the sample reached 650 °C, the sample purge was changed to air, and the sample was heated to 1000 °C at a rate of 50 °C/min and held at 1000 °C for 10 min. This final step is simply to clean the pans and is not used for any analysis but is visible in the complete data that presented in the Supporting Information. Platinum pans were used for all of the studies.

Thermal decomposition data are shown in the Supporting Information. Onset temperatures (*T*_onset_) are reported for the major decomposition steps. The derivative thermogravimetric curves (DTG) were obtained from the experimental TGA data. Decomposition temperatures (*T*_dec._) were obtained by using the maximum thermal decomposition rate of each DTG curve. This was the method used in previous studies allowing us to make direct comparisons for relevant thermal data while attempting to follow established literature procedures.

### 2.3 Single-Crystal Diffraction

Crystallographic data for the compounds were collected on three instruments, as outlined within the CIF files.

Single crystals for the compound **4MeO-I** were coated in Cargille Type NVH immersion oil and transferred to the goniometer of a Rigaku XtalLAB Mini diffractometer with a Mo Kα wavelength (λ = 0.70926 Å) and a CCD area detector. Examination and data collection were performed at 170 K. For this compound, data were collected, reflections were indexed and processed, and the files were scaled and corrected for absorption using CrysAlis PRO.^21^

For the other compounds, single crystals were coated with Parabar 10 312 oil and transferred to the goniometer of either a Bruker D8 Quest Eco diffractometer or a Bruker Quest diffractometer with Mo Kα wavelength (λ = 0.71073 Å) and a Photon II area detector. Examination and data collection were performed at 150 K. Data were collected, reflections were indexed and processed, and the files were scaled and corrected for absorption using APEX3,^22^ SAINT, and SADABS.^23^

For all compounds, the space groups were assigned using XPREP within the SHELXTL suite of programs^24,25^ and the structures were solved by direct methods using ShelXS or ShelXT^26^ and refined by full matrix least-squares against *F*^2^ with all reflections using Shelxl2018^27^ using the graphical interfaces Shelxle^28^ and/or Olex2.^29^ H atoms were positioned geometrically and constrained to ride on their parent atoms. C–H bond distances were constrained to 0.95 Å for aromatic and alkene C–H moieties and to 0.99 and 0.98 Å for aliphatic CH_2_ and CH_3_ moieties, respectively. Methyl H atoms were allowed to rotate, but not to tip, to best fit the experimental electron density. U_iso_(H) values were set to a multiple of U_eq_(C) with 1.5 for CH_3_ and 1.2 for C–H and CH_2_ units, respectively.

Complete crystallographic data in CIF format have been deposited with the Cambridge Crystallographic Data Centre. CCDC 2308275–2308289 contain the supplementary crystallographic data for this paper. These data can be obtained free of charge from the Cambridge Crystallographic Data Centre via www.ccdc.cam.ac.uk/data_request/cif.

Crystallographic details are provided in the Supporting Information for this manuscript.

### 2.4 Structural Analysis Software

Hirshfeld surfaces, images, and fingerprint plots were calculated and produced using CrystalExplorer21.^30^ Images and analysis of the structures were accomplished using Olex2^29^ and Mercury.^31^ Note that for π interactions derived from Hirshfeld surfaces, carbon atoms are discussed as points of interaction on the rings themselves as these are the atoms present in the aromatic rings.

For all images shown of the crystal structures, the following color scheme is used to represent atoms: carbon = gray; nitrogen = blue; hydrogen = white; fluorine = green; oxygen = red; tan = bromine; iodine = purple; and sulfur = yellow. Thermal ellipsoids are shown at a 50% probability.

### 2.5 Computational Studies

The cations from the crystal structures were loaded into Spartan’20 (Wavefunction, 2023) and the structures optimized using the ωB97M-V functional^32^ with a 6–311+G** basis set. Hydrogen distances were optimized. A final energy calculation was performed using the 6–311+G(2df,2p) basis set. Results were checked for imaginary frequencies.

## 3 Results and Discussion

The following sections contain a detailed crystallographic discussion presented as the foundation for our decision tree. Detailed crystallographic studies are shown to allow for follow-up studies to be completed, allowing the tree to continue to grow branches following the rationale and interpretation of the crystallographic data. A summary and recap of the crystallographic studies are presented along the decision tree in Section 4.

### 3.1 Summary of Previous Work

Our initial study of triphenylphosphonium (TPP) ILs focused on understanding the fundamental synthesis^33^ and structural characteristics of the compounds.^34^ A brief summary and rationalization are provided here, establishing context for the study. Full details can be found within the manuscripts.^34,35^

Analysis of these butylated phosphonium salts revealed several key structural principles. For example, the alkyl chains readily adopt gauche and anti conformations in the solid state, a key molecular feature leading to lower melting solids.^36^ Additionally, the torsion angles of phenyl rings in the cation were similar across the series of compounds. This structural trend holds true for related crystal structures reported in the structural database (CSD).^37^ Concerning intermolecular interactions, an important feature that we observed was the occurrence of π interactions in the solid state. Specifically, alkyl−π interactions^38^ were theorized to be important NCIs for these cations. A follow-up study helped verify the formation and importance of π interactions wherein a significant percentage of π stacking interactions between the cation and anion were observed.^35^ Thus, for the present study, we examined how these π interactions can be altered through targeted modification of both the steric and electronic architecture of TPP ILs.

Examining the impact of electronic variation of the π system of the TPP cation was one of the goals for this study. A measure of control can be achieved by the introduction of electron-donating or electron-withdrawing groups to the aromatic ring, as discussed in introductory organic chemistry textbooks. However, the entire nature of the π system can be changed by moving from benzene rings to other heterocycles.^39^ For the study herein, we chose to use tri-2-furylphosphine (TFP) as a contrast to the TPP cations. The TFP core provides three important distinctions that are useful for our study. First, the inclusion of oxygen moieties into the alkyl chains of ILs has been shown to decrease viscosity by reducing ion interactions via introduction of repulsive O···O interactions.^40−42^ Second, the smaller size of the furyl rings was theorized to allow for more accessible rotations of the rings in contrast with the larger phenyl rings. This speculation is based on the well-established “cone angle” calculations.^43^ Finally, to the best of our knowledge, this study would be the first to use the TFP core in the formation of ILs, effectively broadening the accessible landscape of cations for ILs.

### 3.2 Molecular Structures of the Cations

**Figure 2:**
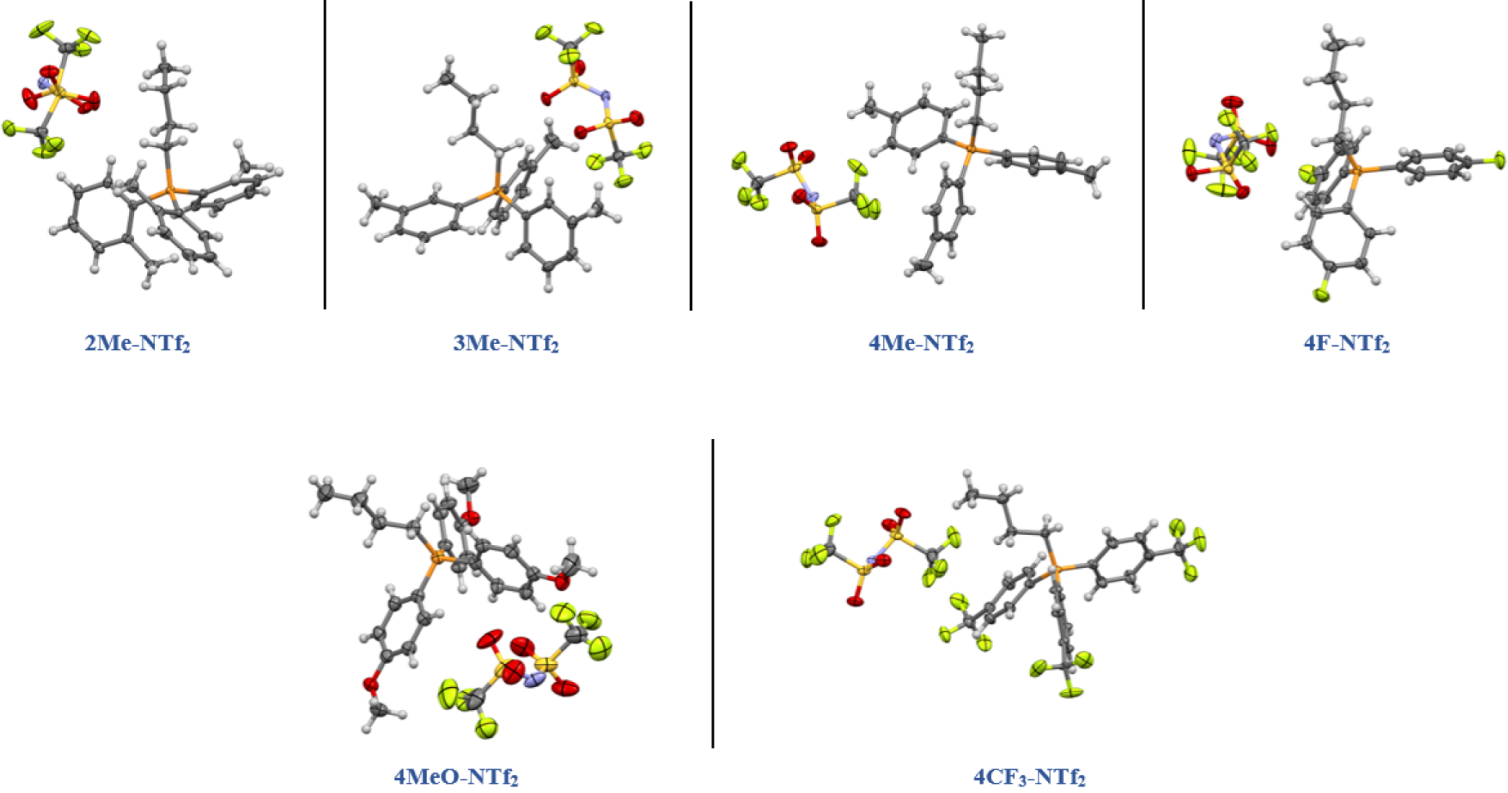
Asymmetric units of the NTf_2_-based compounds examined in this study are shown with 50% probability ellipsoids. Disorder is omitted for clarity in **4Me-NTf**_**2**_ and **4CF**_**3**_**NTf**_**2**_.

The asymmetric units of the NTf_2_-based ILs are shown in Figure 2. The compounds all contain a single cation–anion pair in the asymmetric unit with all of the NTf_2_ moieties in the lower energy trans configuration. The alkyl chains on compounds **4Me-NTf**_**2**_ and **4CF**_**3**_**–NTf**_**2**_ display disorder wherein both the gauche and trans conformations of the alkyl chains are observed. Our previous work^34^ examined this common feature of these class of salts noting that the gauche and anti conformations of the alkyl chains are close in energy.

The cations for the compounds share common geometric features with respect to their overall shape. To broadly describe the geometry (and naming) of the cation, ring A sits beneath the methylene hydrogens of the alkyl chain as the chain extends out from the central phosphorus atom. A more vertical arrangement of this ring, that is, a ring aligned with the P1—C1 bond, would cause steric clash with the alkyl chain, and therefore, ring A lies “flat” compared with the other rings. Ring B sits in the “spine” of the methylene units, often aligning one of the aromatic hydrogen atoms to reside between the alkyl methylene hydrogens H1A and H1B. Ring C typically aligns so that an aromatic hydrogen resides between the alkyl methylene hydrogens on C1 and C2 of the alkyl chain (see Figure S7).

### 3.3 Hirshfeld Surface Analysis

The intermolecular interactions of the crystal structures were examined and quantified via Hirshfeld surface analysis. From the previous work on this class of compounds, we theorized that interactions with the π system of the cations play an important role in the observed structure and properties of this class of compounds. As such, we wished to evaluate the impact on the π-interactions via systematic variation of both sterics and electronics, thereby establishing two conceptual branches in a decision tree.

**Figure 3:**
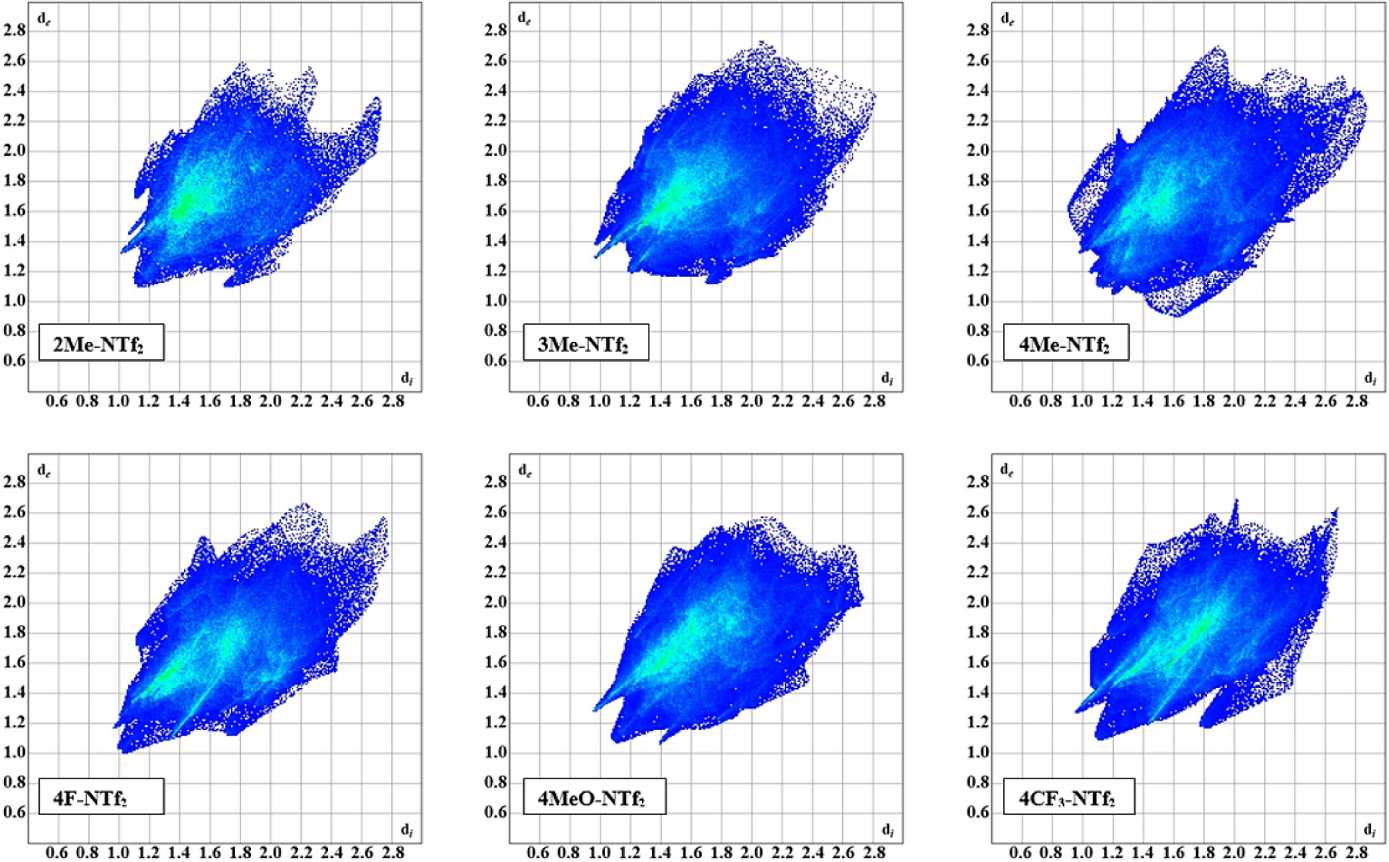
Fingerprint plots for the NTf_2_-based compounds. Similarities in the interactions and structures are noted by the related features within the plots.

The complete fingerprints of the compounds are shown in Figure 3. Cursory inspection of the fingerprints reveals several key similarities in the compounds. For example, each of the fingerprint plots for the compounds displays a set of spikes corresponding to hydrogen interactions. Furthermore, the fingerprints all show a “tail” of disperse interactions at longer distances (*d*_i_ ≈ *d*_e_ ≈ 2.6 Å), indicative of inefficient packing in the crystal. These longer interactions can be seen as dark blue regions on the Hirshfeld surfaces mapped with the *d*_norm_ function (see Figure S8). Finally, each fingerprint displays a green region within the body of the fingerprint, indicating a higher number of interactions within the range of those distances, typically corresponding to H···H intermolecular interactions.

### 3.4 Ortho, Meta, and Para Substitutions: Impacts on π Interactions

**Figure 4:**
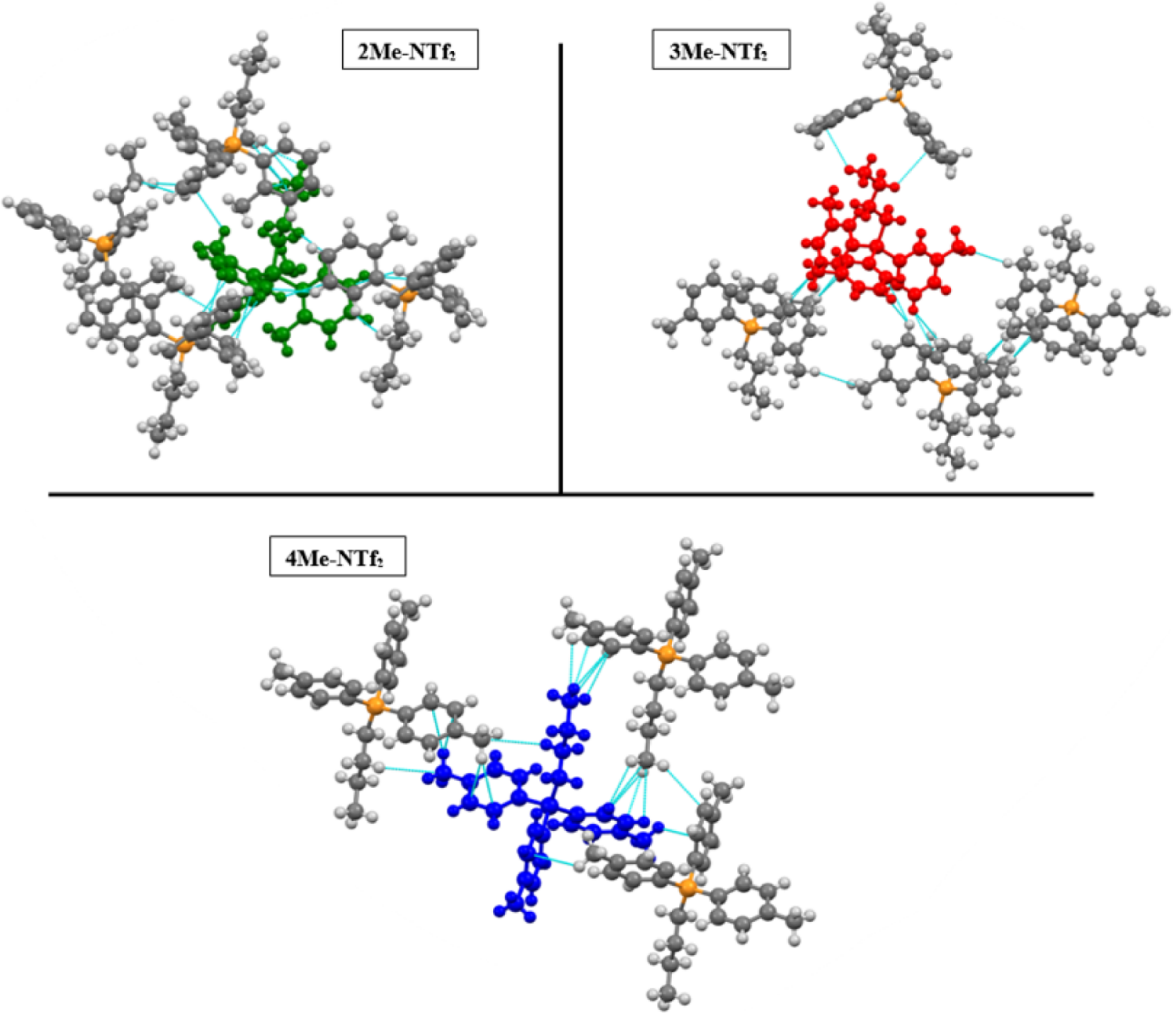
Depiction of the H···C|C···H interactions in **2, 3, and 4Me-NTf**_**2**_ shown with van der Waal radius +0.3 Å. The colored molecules are used simply to help clarify the pictures and to distinguish the “center” molecule.

**Figure 5:**
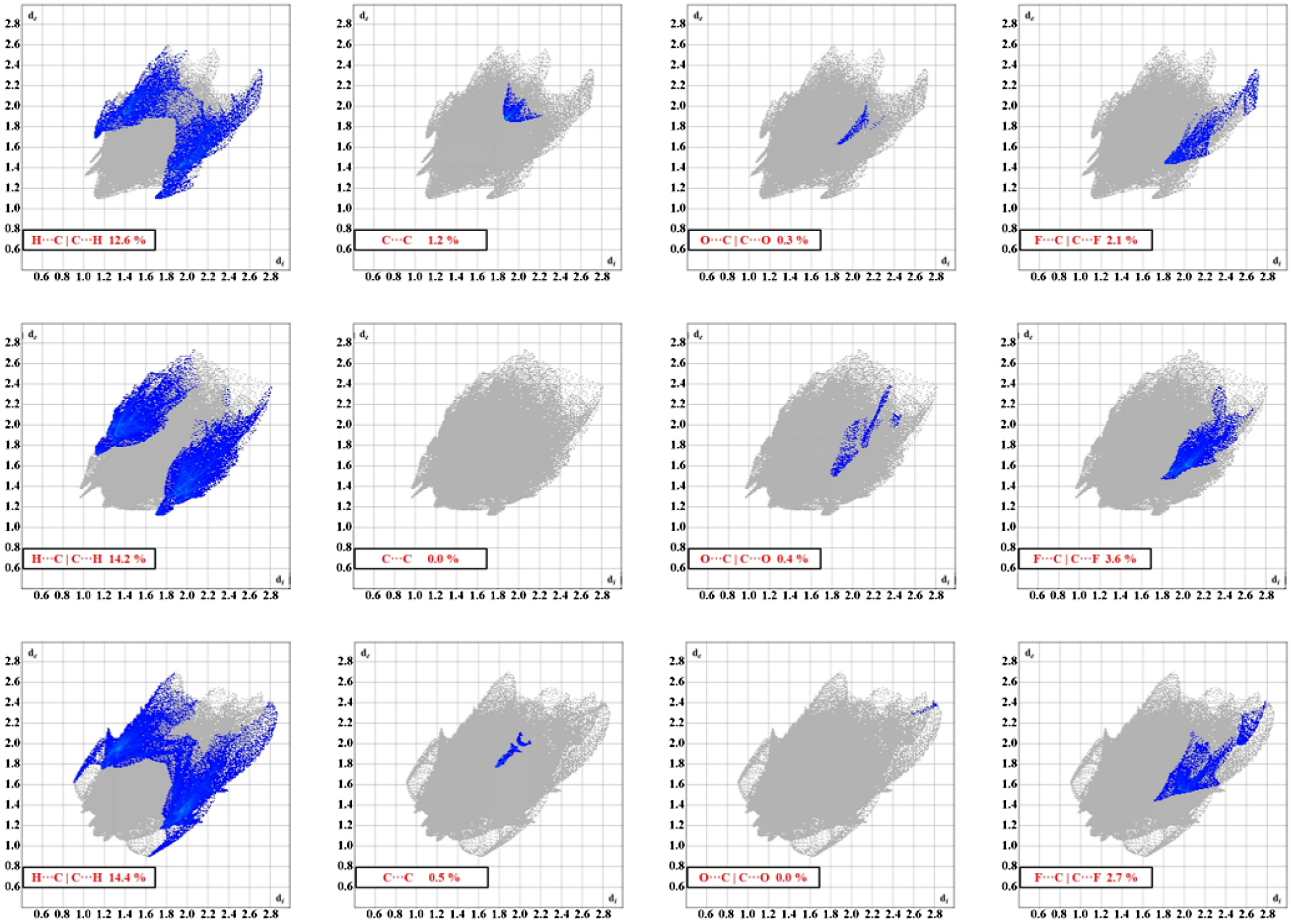
π interaction fingerprints (i.e., H···C|C···H, C···C, O···C|C···O, and F···C|C···F) for compounds **2Me-NTf**_**2**_, **3Me-NTf**_**2**_, and **4Me-NTf**_**2**_. Differences in the interactions are noted by the distinct shapes of the blue regions and overall percentages of interactions.

Compound **2Me-NTf**_**2**_ displays a prominent set of “wings” at *d*_i_ = 1.7 Å, *d*_e_ = 1.1 Å (and the reciprocal distances) corresponding to H···C|C···H π interactions. Predominantly, these interactions arise from the methyl hydrogens on the alkyl chain (C4) and a symmetry adjacent aromatic ring “C” (Figure 4). Additional short interactions do exist, yet these seem to arise more as a consequence of packing rather than as a stabilizing interaction given the geometry (viz., angles and distances) of the interactions. Notably, while compounds **2Me-**,**3Me-**, and **4Me-NTf**_**2**_ exhibit H···C|C···H interactions to an extent, only compound **2Me-NTf**_**2**_ has a distinct wing shape in the fingerprint (see Figure 5).

Curiously, the move from ortho to meta to para substitution increases the relative percentage of H···C|C···H interactions from 12.6% to 14.2% to 14.4% for **2Me-NTf**_**2**_, **3Me-NTf**_**2**_, and **4Me-NTf**_**2**_, respectively. Thus, it may seem that sterics play a role in influencing the total percentage of π interactions with respect to those arising from hydrogens. However, the nature and geometry of these H···π interactions are different for these three compounds, as seen in Figure 4. Both **2Me-NTf**_**2**_ and **4Me-NTf**_**2**_ display alkyl···π interactions in addition to π–π stacks. The π–π stacking is visualized in the C···C fingerprints for the three compounds in Figure 5 and in the Figure S9. These fingerprints clearly show that **3Me-NTf**_**2**_ is distinct in that no observable π–π stacking is seen. For **2Me-NTf**_**2**_ and **4Me-NTf**_**2**_, however, parallel displaced π–π stacking (C···C interactions) is observed. With respect to **2Me-NTf**_**2**_ and **4Me-NTf**_**2**_, two different stacking motifs are observed. For **4Me-NTf**_**2**_, the aromatic methyl groups are forming the π interactions, participating directly in the stacking interaction. In **2Me-NTf**_**2**_, however, the methyl groups are oriented in a manner wherein they do not interact with the stacked π system.

**3Me-I** complicates the analysis as π stacking is observed (see Figure S10). Herein, the aromatic methyl group forms direct interactions with a symmetry adjacent π system similar to that observed in **4Me-NTf**_**2**_. Curiously, **2Me-I** shows no stacking interactions, while **4Me-I** displays an offset, canted π interaction between rings. Thus, it appears that anion size has an influence on which interactions can form or are preferred, highlighting the complexity of analyzing homologous crystal structures of ILs^44^ while also pointing to the necessity for rigorous structural analysis. Speculatively, given that the iodide anion is smaller in size compared to NTf_2_, a closer association between the smaller anion and the phosphonium cation could favor the formation of new interactions, given that the cations will be naturally closer to each other. This theory is verified, to an extent, when comparing the void space (viz. packing efficiency) in **3Me-I** vs **3Me-NTf**_**2**_ (11.2% and 12.6%, respectively) indicating that the smaller anions facilitate a tighter packing of the cations within the solid state.

To summarize, multiple stacking motifs are observed in the crystal structures of the methyl substituted compounds. As anticipated, the interactions are quite complex as a balance of cation–cation and cation–anion π interactions must be considered. Furthermore, in each case, there are competitive interactions such as face-on π stacking vs end-on stacking vs alkyl hydrogen-π interactions. However, despite the complexities, it remains evident that these interactions are key in the formation of the solid-state structures, and thus the melting points, in addition to impacting other physicochemical behavior.

### 3.5 π Interactions in **4F** and **4MeO**: Electron-Rich Vs Electron-Deficient Systems

**Figure 6:**
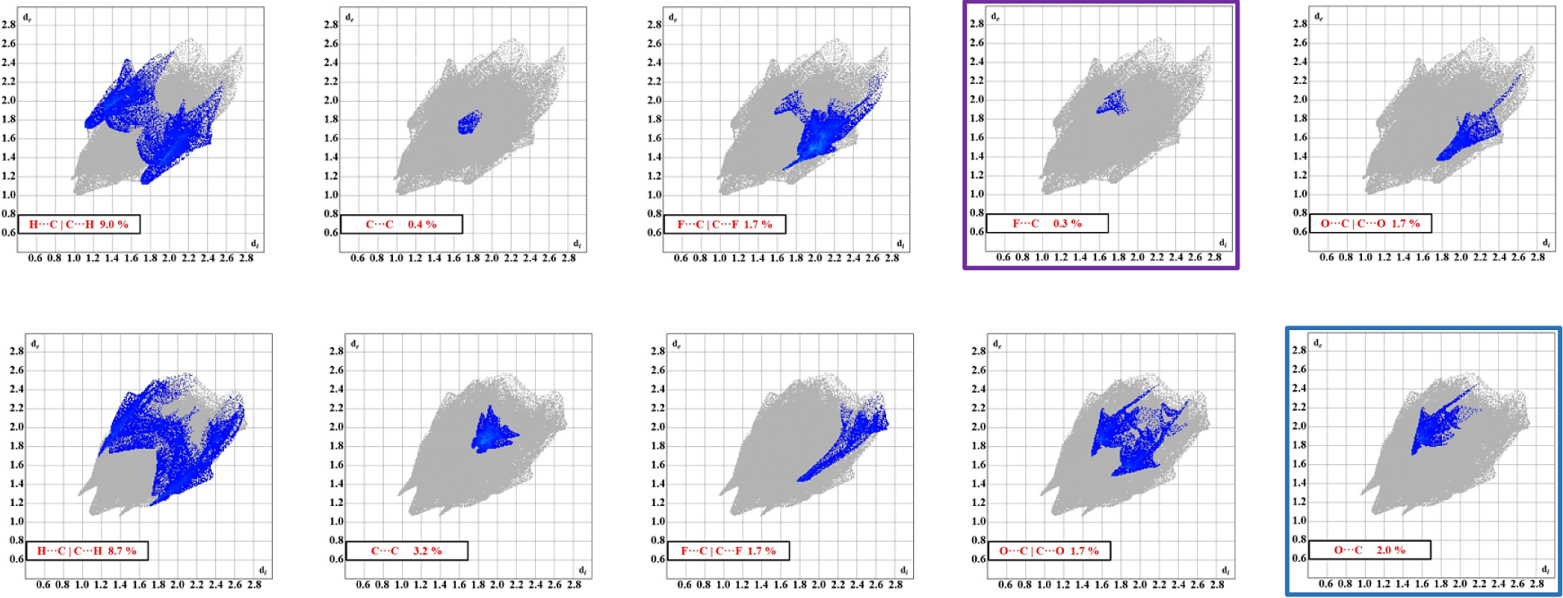
Fingerprint plots of the π interactions in **4F-NTf**_**2**_ (top row) and **4MeO-NTf**_**2**_ (bottom row). Highlighted boxes show interactions arising solely from atoms on the cations, indicating cation–cation interactions formed due to the functional groups in the para position.

Compounds **4F** and **4MeO** were synthesized to examine the impact of electron-withdrawing (fluoro) and electron-donating groups (methoxy) on the rings. The change in functional group and the corresponding changes in the electronic structure lead to several key differences in interactions for the compounds. As previously mentioned, there are similarities in the overall shape of the fingerprints (e.g., sharp H interaction spikes, a blunted central spike of H···H interactions, green regions, disperse spots, etc.); however, our specific interest revolved around changes in π interactions. Relevant π interaction fingerprints for **4F-NTf**_**2**_ and **4MeO-NTf**_**2**_ are shown in Figure 6.

#### 3.5.1 Alkyl····π and π–π Stacking Interactions

While both compounds exhibit a comparable percentage of C–H···π interactions, the geometry and nature of these interactions are quite different. **4MeO-NTf**_**2**_ has two sets of interactions visualized as two distinct, sharp outer and inner wings. **4F-NTf**_**2**_, on the contrary, displays two blunted wings with the same inner and outer motifs. A visualization of these interactions is shown in Figure S11. In **4F-NTf**_**2**_, the shortest interactions arise from the alkyl methyl hydrogens (C4) and a symmetry adjacent aromatic ring at H···C distances in the range 2.941–2.955 Å. The other alkyl hydrogens on the chain also interact with the aromatic ring at increasing distances and are partly responsible for the blunted and disperse shape of outer wings in the fingerprints. The inner wings correspond, in part, to H···C|C···H contacts due to parallel offset stacking interactions between aromatic moieties. Thus, the two wing features in the fingerprint of **4F-NTf**_**2**_ arise due to two distinct interaction motifs: π-stacking and alkyl hydrogen···π.

The H···C|C···H interactions in **4MeO-NTf**_**2**_ arise from the methoxy hydrogens in addition to longer interactions with aromatic and methylene moieties, making the interactions more complex than those in **4F-NTf**_**2**_. The sharper outer wing in the fingerprint comprises interactions from the methoxy methyl hydrogen atoms with a symmetry adjacent π system at a distance of 2.935 Å (*d*(H···C)), which are marginally shorter interactions than comparable interactions observed in **4F-NTf**_**2**_. Curiously, the methoxy hydrogens also make close contacts (∼2.98 Å, *d*(H···C)) with adjacent methylene units. While it has been shown that methyl groups can act as Lewis bases,^45^ these interactions herein are likely a consequence of packing rather than any stabilizing or guiding interaction for this compound.

**Figure 7:**
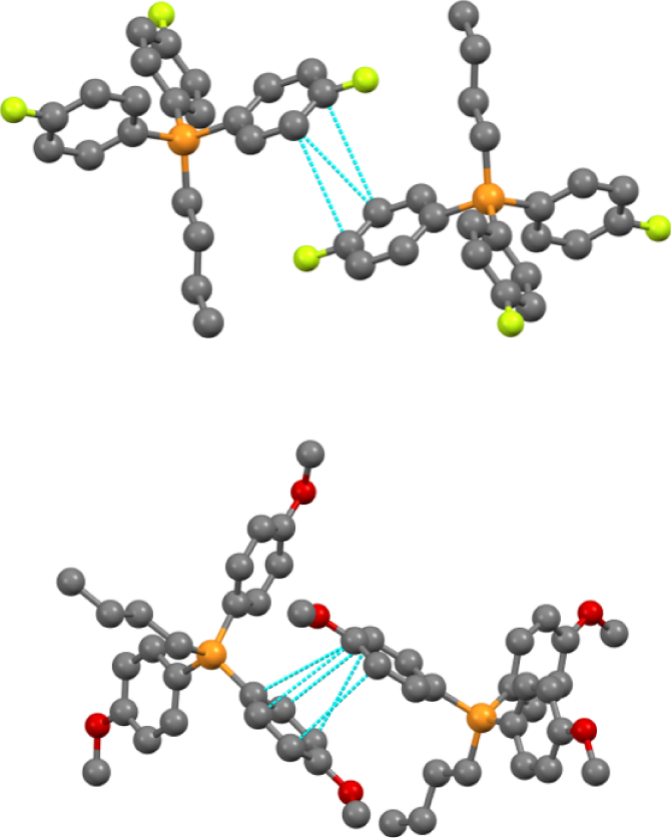
Depiction of the C···C stacking interactions in **4F-NTf**_**2**_ (top) and **4MeO-NTf**_**2**_ (bottom). Hydrogens are omitted for clarity. Blue lines show atom–atom contacts, specifically, C···C interactions.

The C···C interactions, which correspond to π-stacking, are quite distinct between the two systems (see Figure 7). **4MeO-NTf**_**2**_ shows a significantly higher percentage (3.2%) than **4F-NTf**_**2**_ (0.4%). However, the stacking interactions are shorter for **4F-NTf**_**2**_ than in **4MeO-NTf**_**2**_, ranging from ∼3.36 to 3.67 Å. In **4MeO-NTf**_**2**_, the C···C distances range from ∼3.60 to 4.0 Å. While both compounds display parallel offset π stacking, **4F-NTf**_**2**_ shows barely any overlap, with only the edges of the aromatic rings being in close contact. **4MeO-NTf**_**2**_, however, displays a more common offset interaction with rings “A” and “B” residing in a more traditional stacking motif. The more pronounced stacking interactions in **4MeO-NTf**_**2**_ are theorized to be part of the contribution to the lower melting point of these compounds (see Section 4.2). Additional π interactions, however, are also a contributing factor as described in the subsequent sections.

#### 3.5.2 F···π Interactions

Examining the F*···*C|C*···*F fingerprints for both molecules reveals several key features (Figure 6). First, for both **4F-NTf**_**2**_ and **4MeO-NTf**_**2**_, the shortest F*···*C interactions arise from an anion fluorine ( making close contact with an aromatic carbon in the para position (C8) likely due to the inductive effects of the electron-withdrawing group they are bonded with. This interaction is shorter in **4F-NTf**_**2**_ than in **4MeO-NTf**_**2**_. Specifically, the shortest interactions in **4F-NTf**_**2**_ are between C8C and F6A at a distance of 2.959(4) Å (*d*(C8C*···*F6A*^i^*), *i* = 1-x, 1-y, 1-z). In **4MeO-NTf**_**2**_, the shortest interaction is between C8A and F5 at a distance of 3.215 (4) Å (*d*(C8A···F5)). The different distances can also be seen in the fingerprint plots upon examination of the crescent shapes of the interactions.

Second, both compounds show additional ···C interactions wherein a fluorine atom is interacting in a side-on manner with an aromatic ring. While these interactions are present in both compounds, given the geometry of these interactions, it appears that these specific C···F contacts, as calculated in the Hirshfeld analysis, arise from contacts with the hydrogens rather than interactions with the carbons themselves. It should be noted that in **4F-NTf**_**2**_, these side-on ···C distances range from 3.01–3.17 Å, making them shorter than the sum of the radii of the carbon and fluorine atoms, however.^46^ For **4MeO-NTf**_**2**_, the analogous interactions range from 3.37 to 3.56 Å making them longer than in **4F-NTf**_**2**_ and slightly longer than the sum of the atomic radii.

Finally, and of particular importance, the shortest C···F interactions arising from the fluorine atoms residing on the cation in **4F-NTf**_**2**_ are the symmetry adjacent alkyl chain carbons. Thus, no direct contact with carbon atoms is observed from the fluorine atoms. This observation explains why there is only a negligible difference in the total percentages of F*···*C|C*···*F interactions when comparing **4F-NTf**_**2**_ to **4MeO-NTf**_**2**_ since these interactions arise from the aforementioned aromatic carbon interactions with the anion fluorines. Thus, the inclusion of a fluorine or methoxy moiety on the TPP cation does not appear to change the formation of F*···*C|C*···*F interactions, specifically, as the anion fluorine atoms form the shortest interactions with the π systems of the aromatic rings. A visualization of these C*···*F interactions is shown in Figure S12.

#### 3.5.3 O···π Interactions

The O···C|C···O interactions represent the other key set of interactions involving the π system of the TPP cations. Herein, we observe a more pronounced distinction between the two compounds, with **4MeO-NTf**_**2**_ having a significantly higher percentage of O···C|C···O interactions than **4F-NTf**_**2**_.

In **4F-NTf**_**2**_, the majority of the close contacts between the oxygen atoms on the anion () and carbon atoms are in a side-on manner with the aryl rings, likely manifesting as a consequence of H···O|O···H close contacts rather than arising due to specific interactions with the carbons. However, there are several face-on ···π interactions as well, ranging in distances from 3.14 to 3.43 Å (*d*(O···C)). Unlike with the ···C interactions, these face-on interactions are not with the carbon in the para position.

**4MeO-NTf**_**2**_ shows a clear point of distinction in that the shortest C···O interaction is between two cation moieties wherein the methoxy oxygen (O1A) is interacting with a symmetry adjacent aromatic carbon C6 in the ortho position at a distance of 3.207 (4) Å (d(C*^j^*···O), *j* = 1/2 + x, 1/2-y, 1/2+z). Of note, these rings are the same, which participate in the π–π stacking previously discussed (Section 3.5.1). Additional longer interactions arising from the methoxy oxygen do exist with other carbon atoms in the rings, making the interactions in **4MeO-NTf**_**2**_ quite complex. A visualization of these C···O interactions is shown in Figure S13.

### 3.6 Summary of π Interactions for **4F-NTf**_**2**_ and **4MeO-NTf**_**2**_

In summary, several key observations are relevant within the context of rationalizing the thermal properties of these compounds via crystallographic interactions.

- Both **4F-NTf**_**2**_ and **4MeO-NTf**_**2**_ display parallel offset stacking interactions of the rings. For **4MeO-NTf**_**2**_, however, the stacking interactions are far more prominent, showing greater overlap of the π systems and thus increased cation–cation interactions.
- Despite the addition of a fluorine moiety on the cation in **4F-NTf**_**2**_, only a negligible amount of F···π interactions between cations is observed. In fact, both **4F-NTf**_**2**_ and **4MeO-NTf**_**2**_ show comparable sets of these interactions, leading to the conclusion that these interactions are not responsible for the differences in thermophysical properties.
- Additional cation–cation interactions are observed in **4MeO-NTf**_**2**_ arising from the methoxy oxygen. Specifically, we note that the methoxy oxygen is interacting with the central phosphonium atom on a symmetry adjacent cation, further adding to the list of observed cation–cation interactions while also helping stabilize the close contact between cations, a key feature leading to lower melting points of ILs. The cation fluorine atom in **4F-NTf**_**2**_, however, does not show comparable interactions, preferring the formation of cation···anion, ···P interactions instead.

## 4 Thermal Characterization and Discussion

### 4.1 Overview of Phase Transitions

**Figure 8:**
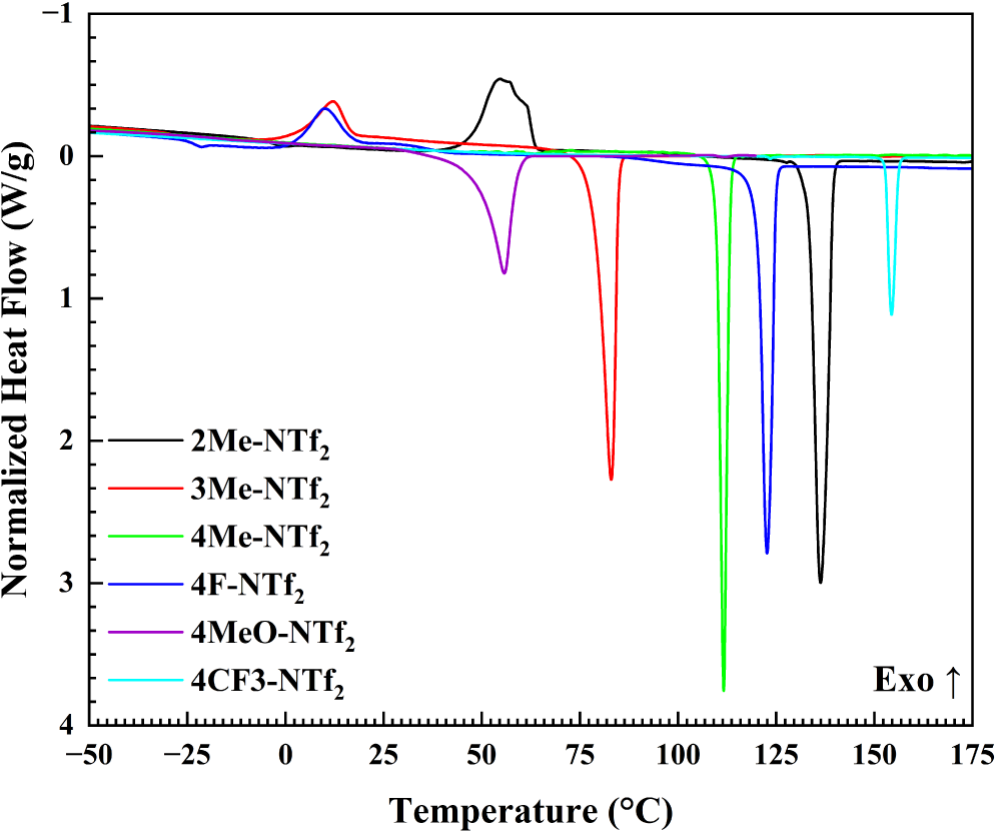
DSC traces for the NTf_2_-based compounds, showing the phase transitions of the salts examined herein.

Targeted modification of the aromatic rings on the TPP cations has a profound impact on the phase transitions of the compounds. A summary of relevant thermal data is provided in Table 1, and the DSC traces are shown in Figure 8. Key trends are observed when contrasting the different groups of the compounds examined herein.

**Table 1:** Compiled Experimental Data for the Compounds Examined Herein^a^

	MW g/mol	V_HS_^a^Å^3^	A_HS_^b^Å^2^	*T*_onset_^c^ (°C)	*T*_dec_^d^ (°C)	*T*_m_^e^ (°C)	*T*_CC_^f^ (°C)	*T*_g_^g^ (°C)	Dipole^h^ (D)
2Me	361.49	514.62	412.71	419.84	453.47	136.30	54.70	–3.97	1.58
3Me	361.49	538.13	457.75	414.94	459.90	82.99	12.11	–28.58	1.83
4Me	361.49	533.89	448.48	420.52	462.52	111.63	-	–15.92	1.53
4F	373.38	470.49	404.33	386.91	428.04	122.68	9.84	–24.88	1.27
4MeO	409.49	565.70	490.53	414.96	474.89	55.73	-	–17.26	2.43
4CF3	523.41	603.51	506.18	403.83	434.57	154.40	-	-	2.62
TFP	289.29	-	-	394.39	435.52	-	-	-	3.84

a a, Volume of cation based on calculated Hirshfeld surface; b, surface area of cation based on calculated Hirshfeld surface; c, onset temperature of thermal decomposition; d, decomposition temperature based on maximum from first derivative; e, melting point; f, cold crystallization temperature; g, glass transition temperature; h, calculated dipole moment of the cation.

With respect to positional substitutions (i.e., ortho vs meta vs para), the **2Me-NTf**_**2**_ derivative displays the highest melting point at ca. 136 °C, followed by **4Me-NTf**_**2**_ at ca. 112 °C, and **3Me-NTf**_**2**_ at ca. 83 °C. Thus, a range of approximately 53 °C (i.e., Δ*T*_m_ = 53 °C) is realized through simply changing the position of the methyl groups. Even more notable is that **3Me-NTf**_**2**_ has a lower *T*_m_ than the unsubstituted counterpart (i.e., **4H-NTf**_**2**_), which has a *T*_m_ of ca. 89 °C.

Addition of a fluorine moiety to the para position raises the melting point to approximately 123 °C. This addition mirrors what is observed in **4Me-NTf**_**2**_ wherein para substitution coincides with an increase in *T*_m_ values when contrasted with the unsubstituted derivative. Substitution for a trifluoromethyl group causes a drastic increase in the melting point to ca. 155 °C. In one way, this continues the observed trend wherein substitution at the para positions increases *T*_m_. However, the CF_3_ moiety has a significantly higher molecular weight than the **4F** or **4Me** derivatives, which inevitably will affect the melting points of the compounds.

### 4.2 Computational Studies and Ties to Interactions

To delve further into the structural impacts of the functional group modifications, a point to be addressed is the discussion of the electronic impacts of the change from methyl to fluoro to trifluoromethyl. As has been shown by Davis et al., the dipole of the cation in an IL is inversely proportional to the melting point.^17^ That is, increasing dipoles lead to lower melting points. It is speculated that this relationship is due to the increased ordering of the cation in the liquid state as dipole moments increase, leading to lower entropic and enthalpic contributions to phase transitions. This ordering of the cations arises due to the formation of specific, directional NCIs between the cations. Indeed, we observed this theory to hold true in other related triaryl-bearing compounds.^47^

Our previous work also revealed that in addition to the magnitude of the dipole, the direction is also relevant. In brief, changes in the orientation of the cation dipole can allow (or prevent) the formation of cation–cation interactions. Increased cation–cation interactions, or “exchanging” cation–anion interactions for cation–cation interactions, would then lower the melting point. Thus, as is often the case with ILs, a complex relationship of sterics, electronics, and melting points is observed. As such, preliminary computational studies were conducted to help connect the observed interactions with the phase transitions. Several key details from the theoretical studies help to draw out conclusions when paired with the experimental data.

The dipole moments of the cations vary quite significantly, even when examining the methyl derivatives. The calculated dipoles for 2Me, 3Me, and 4Me cations are 1.58 1.83, and 1.53 D respectively. This does follow previous trends, wherein higher dipole moments correspond to lower melting points, particularly with 3Me. When contrasting 2Me and 4Me, there is a change in the dipole, yet 2Me has a much higher melting point (ca. 136 °C) than 4Me (ca. 111 °C) despite having a higher dipole moment. However, we have observed in previous works that ortho substitution of triaryl groups raises melting points of ILs.^47^ We theorize that the substitutions in the ortho position affect the melting point on the basis of not only the interactions but also intramolecular sterics rather than solely changes in the molecular dipole moments. A more in-depth discussion of these intramolecular interactions and their influence on melting points is provided in the Supporting Information (see Section 2 and Section 3 and Figure S14).

The TFP cation has the highest dipole moment (3.89 D) and the lowest *T*_m_, existing as a free-flowing liquid at room temperature. We believe the more readily accessible conformations of the smaller furan rings play a significant role in the phase behavior of this compound, with the increased rotational orientations preventing the formation of π interactions, which stabilize the solid state of the TPP compounds. To this point, past studies of ILs have shown that the addition of ether moieties to alkyl chains has a similar effect of lowering melting points by introducing repulsive O···O interactions between chains. Thus, the inclusion of the oxygen moiety in the aromatic system could have a similar effect, hindering the formation of stacking interactions. Indeed, there is no observed π-stacking in the crystal of **TFP-I**. These conclusions are speculative, however, given that we do not have a crystal structure of the NTf_2_ derivative and the heavy disorder present in the iodide crystal.

## 5 Relationships Between the Crystal Interactions and Melting Point Values

**Figure 9:**
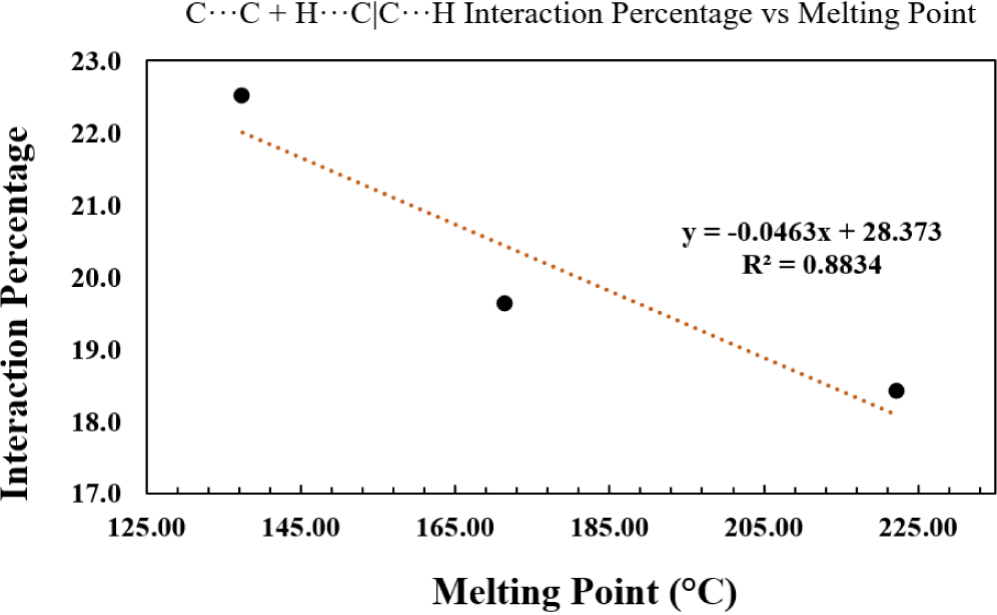
Graph of the best fit for melting points of **2Me-I**, **3Me-I**, and **4Me-I** vs noncovalent interaction percentages calculated from the Hirshfeld surface analysis.

Herein, we present a rigorous examination of the π interactions of several TPP-based compounds. The question remains as to how or if these interactions are relevant with respect to influencing the melting point of the compounds. As is well established, Coulombic forces within ILs dominate the interactions present within organic salts yet still require a balance between repulsive and attractive interactions.^16^ Thus, the noncovalent interactions will still influence the properties of ILs. To simplify the present study and to provide evidence toward the impact of these π interactions, the melting points of the iodide salts of the ortho, meta, and para isomers (i.e., **2Me-I**, **3Me-I**, **4Me-I**) were examined and compared with the H···C|C···H and C···C interactions drawn from the Hirshfeld surface analysis. Figure 9 shows the graphed data with the best-fit lines. From the graph, several key details emerge.

As theorized, the π interactions have a strong correlation with the melting point of the compounds. The contribution of the H···C|C···H and C···C interactions provided the strongest correlation with the melting points. These sets of interactions, H···C|C···H and C···C, do not represent the totality of the interactions or the largest overall contribution based on percentage. However, these two interactions arise from π interactions, which structurally comprise the largest percentage (by area) of the molecules. Inclusion of the H···H interaction percentages into the linear fitting reduces the fit for this set of data. Thus, the percentage of stacking and π interactions are the two best interactions to model with respect to the prediction of melting points for these TPP-based compounds.

### 5.1 Decision Tree

The preceding crystallographic discussion lays a rational foundation for the interactions examined, provides a framework leading to our conclusions, and is meant as a template for future studies, enabling others to follow our steps in developing empirical thermophysical models of ILs. Using the crystallographic data, we developed a model wherein it was demonstrated that π interactions have a strong correlation to the melting point. In taking the next step and deriving a decision tree from the discussion, however, it is crucial to summarize several key aspects: positional substitution, electronics, and cation geometry.

With respect to positional substitutions, incorporating a methyl group in the meta position resulted in the lowest melting point within this homologous series of compounds. To the point, the melting point of **3Me-NTf**_**2**_ is lower than that of the unsubstituted derivative^34^ (viz. **4H-NTf**_**2**_). Conversely, both the ortho and para positions had higher melting points than **4H-NTf**_**2**_, providing another branching path. While perhaps more speculative, and a subject for future investigation, 2Me and 4Me also branch given that 2Me displays a notably higher melting point than 4Me. Examining previously reported data on related compounds does reveal a similar trend in that ortho substitutions on triaryl phosphonium cations produces higher melting points than substitutions in the para position.^17^

While the branches stemming from the electronic studies are somewhat more nuanced, a distinct branching occurs when comparing 4MeO with 4F. Specifically, **4MeO-NTf**_**2**_ had the lowest melting point of all of the phenyl-based compounds examined. **4F-NTf**_**2**_, on the contrary, had a melting point above that of **4H-NTf**_**2**_ and between 2Me and 4Me. Thus, electron-rich aromatic rings appear to have a notable decrease in the melting point, leading to a clear branching point in the proposed tree. A strong electron-withdrawing group, namely **4CF**_**3**_**–NTf**_**2**_, further increases the melting point when contrasted with 4F. One caveat when comparing 4MeO with 4F with 4CF_3_ is that 4CF_3_ has the highest dipole moment, a property that has been shown to correlate inversely with melting point (viz. high dipole moments, lower melting points).

With respect to the overall cation geometry, phosphonium compounds bearing furyl rings have lower melting points than those with phenyl rings. We theorize this is due to increased cation disorder due to increased ring rotations, as evidenced by the multiple orientations observed in the **TFP-I** crystal (see Supporting Information). This theory relating the ability of the rings to rotate to changes in the melting point is supported partly by the lack of disorder observed in previously reported crystal TPP-based structures. Tentatively, we can point toward the idea of relating cone angles^48^ and melting points as a future point of investigation as TFP has a smaller cone angle than TPP.

**Figure 10:**
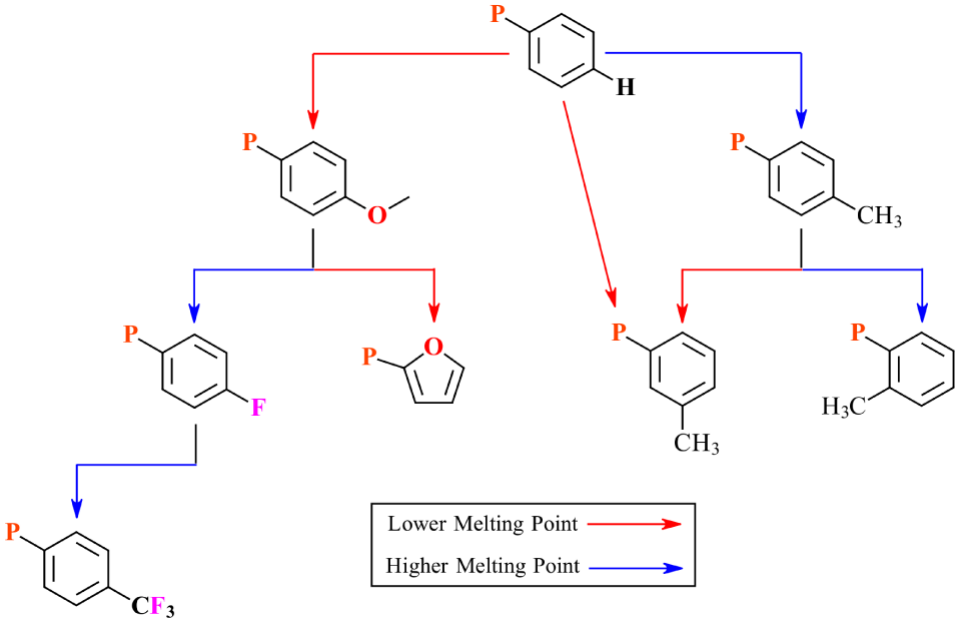
Preliminary decision tree for aryl-containing ILs based on controlling the melting point.

With these points in mind, we present a preliminary decision tree as a visual summary of the data and discussion herein (see Figure 10).

## 6 Conclusions

A complex relationship for ILs exists that encompasses many fundamental aspects of crystal engineering and molecular design: sterics, identity of the functional group, dipole moments, directional interactions, molecular synthons, and molecular weight, all of which contribute to the properties and behavior of these compounds. Despite this complexity, we have managed to establish key ideas that are useful for the future development of ionic liquids. Several key points can be drawn in addition to those directly related to the development of a decision tree:

i With respect to thermal stability, substitution of the aromatic rings did not appear to influence the decomposition temperatures. While displaying the lowest thermal stability of the series herein, **TFP-NTf**_**2**_ still displays a high overall stability characteristic of phosphonium ILs.
ii Inclusion of some functional groups (e.g., 4F) did not introduce interactions from those specific moieties. While this manuscript was focused on assessing melting points and drawing correlations to interactions, phase transitions are not the only physicochemical property that could be assessed. As stated, thermal stability was not significantly affected with the addition of these groups. Furthermore, from a qualitative perspective, introduction of the 4F moiety changed the solubility of the formed IL, making it more soluble in water than the 4CF_3_ derivative. Solubility, as an example, is an important property to note when choosing the appropriate materials for a task. Likewise, tailoring hydrophilicity or lipophilicity is an important property to consider when studying ILs.
iii There could exist a more complex relationship of interactions that could, perhaps, better account for the melting points observed herein. We anticipate that our subsequent manuscripts within this field will continue to nurture and grow this tree, refining the branches and relationships therein. Indeed, there is fertile ground yet to develop this model so as to more accurately predict and present future ILs.
iv The data presented herein is predictive. Specifically, we anticipate that the yet unreported compound **3MeO-NTf**_**2**_ will have a notably lower melting point than **4MeO-NTf**_**2**_ based on our conclusions herein, perhaps even existing as a room temperature liquid, albeit likely a viscous liquid. Furthermore, we anticipate that the **3F-NTf**_**2**_ will have a lower melting point than the **2F-NTf**_**2**_ and **4F-NTf**_**2**_. We further speculate that the **3MeO-NTf**_**2**_ derivative will have a lower melting point than the **3F-NTf**_**2**_ derivative. These two compounds, among others from a curated set, are part of our ongoing study to continue to expand the decision tree.

The triphenylphosphine moiety has proven useful in the pursuit of developing and understanding the structure of ILs. In particular, the highly crystalline nature of these compounds allows single crystals to be readily grown. As with other structural studies of ILs in the past, careful examination of these crystals can provide a wealth of information to help unravel the complexities of ILs. The information gathered from the crystal structures helps supplement and validate past studies regarding the relationships between dipole moments and *T*_m_ values. With the results gathered herein, we continue to examine the structures and properties of these compounds, with a particular focus on controlling their thermal properties. Our future work will be aimed at addressing some of the lingering questions herein (e.g., long-term thermal stability, the role of the furyl oxygen moiety, ring sterics, etc.).

## Supporting Information Available

Full synthetic procedures are provided. Furthermore, the complete TGA, DSC, NMR spectra, and computational data are provided (ZIP)
Crystallographic details have been provided (ZIP)
Background and rationalization; ring rotations and the impact on the melting point; alkyl chain confirmations, halide confirmational polymorphs, impacts of the halide salt, interactions of **4CF_3_-NTf_2_**, on the shape and geometry of the functional groups **(**PDF**)**
