# Supplementary material for: Planting the Seeds of a Decision Tree for Ionic Liquids: Steric and Electronic Impacts on Melting Points of Triarylphosponium Ionic Liquids
Source: J Phys Chem B. 2024 Jun 7;128(24):5895–907. doi: 10.1021/acs.jpcb.4c02196 (PMC11194809; doi:10.1021/acs.jpcb.4c02196)

checkCIF/PLATON report

Structure factors have been supplied for datablock(s) fgcupch0128_0m

THIS REPORT IS FOR GUIDANCE ONLY. IF USED AS PART OF A REVIEW PROCEDURE FOR PUBLICATION, IT SHOULD NOT REPLACE THE EXPERTISE OF AN EXPERIENCED CRYSTALLOGRAPHIC REFEREE.

No syntax errors found. CIF dictionary Interpreting this report

Datablock: fgcupch0128_0m

Bond precision:	C-C = 0.0038 A		Wavelength=0.71073
Cell:	a=15.6186(4)	b=14.8511(4)	c=23.9557(6)
	alpha=90	beta=103.749(1)	gamma=90
Temperature:	150 K		

	Calculated	Reported
Volume	5397.4(2)	5397.4(2)
Space group	C 2/c	C 1 2/c 1
Hall group	-C 2yc	-C 2yc
Moiety formula	2(C25 H21 F9 P), C2 H6 O, 2(I)	I, C25 H21 F9 P, 0.5(C2 H6 O)
Sum formula	C52 H48 F18 I2 O P2	C26 H24 F9 I O0.50 P
Mr	1346.64	673.32
Dx,g cm-3	1.657	1.657
Z	4	8
Mu (mm-1)	1.324	1.324
F000	2664.0	2664.0
F000'	2662.81	
h,k,lmax	22,21,34	22,21,34
Nref	8315	8297
Tmin,Tmax	0.779,0.894	0.664,0.746
Tmin'	0.779	

Correction method= # Reported T Limits: Tmin=0.664 Tmax=0.746
AbsCorr = MULTI-SCAN

Data completeness= 0.998 Theta(max)= 30.601

R(reflections)= 0.0454(7091) wR2(reflections)= 0.0819(8297)

S = 1.171 Npar= 355

The following ALERTS were generated. Each ALERT has the format
test-name_ALERT_alert-type_alert-level.
Click on the hyperlinks for more details of the test.

Alert level C

```
PLAT042_ALERT_1_C Calc. and Reported MoietyFormula Strings  Differ     Please Check
PLAT213_ALERT_2_C Atom F2B            has ADP max/min Ratio .....        3.1 prolat
PLAT213_ALERT_2_C Atom F2C            has ADP max/min Ratio .....        3.7 prolat
PLAT906_ALERT_3_C Large K Value in the Analysis of Variance ......      6.666 Check
```

Alert level G

```
PLAT007_ALERT_5_G Number of Unrefined Donor-H Atoms .............          1 Report
PLAT045_ALERT_1_G Calculated and Reported Z Differ by a Factor ...      0.500 Check
PLAT083_ALERT_2_G SHELXL Second Parameter in WGHT  Unusually Large      17.09 Why ?
PLAT187_ALERT_4_G The CIF-Embedded .res File Contains RIGU Records          1 Report
PLAT242_ALERT_2_G Low    'MainMol' Ueq as Compared to Neighbors of       C11A Check
PLAT242_ALERT_2_G Low    'MainMol' Ueq as Compared to Neighbors of       C11B Check
PLAT242_ALERT_2_G Low    'MainMol' Ueq as Compared to Neighbors of       C11C Check
PLAT300_ALERT_4_G Atom Site Occupancy of O1        Constrained at        0.5 Check
PLAT300_ALERT_4_G Atom Site Occupancy of C1A       Constrained at        0.5 Check
PLAT300_ALERT_4_G Atom Site Occupancy of C1B       Constrained at        0.5 Check
PLAT300_ALERT_4_G Atom Site Occupancy of H1        Constrained at        0.5 Check
PLAT300_ALERT_4_G Atom Site Occupancy of H1AA      Constrained at        0.5 Check
PLAT300_ALERT_4_G Atom Site Occupancy of H1AB      Constrained at        0.5 Check
PLAT300_ALERT_4_G Atom Site Occupancy of H1BA      Constrained at        0.5 Check
PLAT300_ALERT_4_G Atom Site Occupancy of H1BB      Constrained at        0.5 Check
PLAT300_ALERT_4_G Atom Site Occupancy of H1BC      Constrained at        0.5 Check
PLAT302_ALERT_4_G Anion/Solvent/Minor-Residue Disorder (Resd  2  )       100% Note
PLAT304_ALERT_4_G Non-Integer Number of Atoms in ..... (Resd  2  )       4.50 Check
PLAT415_ALERT_2_G Short Inter D-H..H-X       H1       ..H10C     .       2.03 Ang.
                                                     x,y,z  =      1_555 Check
PLAT720_ALERT_4_G Number of Unusual/Non-Standard Labels .........          5 Note
PLAT773_ALERT_2_G Check long C-C Bond in CIF: C1A      --C1B             1.72 Ang.
PLAT789_ALERT_4_G Atoms with Negative _atom_site_disorder_group  #          9 Check
PLAT802_ALERT_4_G CIF Input Record(s) with more than 80 Characters          2 Info
PLAT822_ALERT_4_G CIF-embedded .res Contains Negative PART Numbers          1 Check
PLAT860_ALERT_3_G Number of Least-Squares Restraints ............          9 Note
PLAT910_ALERT_3_G Missing # of FCF Reflection(s) Below Theta(Min).          3 Note
PLAT912_ALERT_4_G Missing # of FCF Reflections Above STh/L=  0.600         14 Note
PLAT978_ALERT_2_G Number C-C Bonds with Positive Residual Density.          8 Info
```

0 **ALERT level A** = Most likely a serious problem - resolve or explain
0 **ALERT level B** = A potentially serious problem, consider carefully
4 **ALERT level C** = Check. Ensure it is not caused by an omission or oversight
28 **ALERT level G** = General information/check it is not something unexpected

2 ALERT type 1 CIF construction/syntax error, inconsistent or missing data
9 ALERT type 2 Indicator that the structure model may be wrong or deficient
3 ALERT type 3 Indicator that the structure quality may be low
17 ALERT type 4 Improvement, methodology, query or suggestion
1 ALERT type 5 Informative message, check

It is advisable to attempt to resolve as many as possible of the alerts in all categories. Often the minor alerts point to easily fixed oversights, errors and omissions in your CIF or refinement strategy, so attention to these fine details can be worthwhile. In order to resolve some of the more serious problems it may be necessary to carry out additional measurements or structure refinements. However, the purpose of your study may justify the reported deviations and the more serious of these should normally be commented upon in the discussion or experimental section of a paper or in the "special_details" fields of the CIF. checkCIF was carefully designed to identify outliers and unusual parameters, but every test has its limitations and alerts that are not important in a particular case may appear. Conversely, the absence of alerts does not guarantee there are no aspects of the results needing attention. It is up to the individual to critically assess their own results and, if necessary, seek expert advice.

Publication of your CIF in IUCr journals

A basic structural check has been run on your CIF. These basic checks will be run on all CIFs submitted for publication in IUCr journals (*Acta Crystallographica*, *Journal of Applied Crystallography*, *Journal of Synchrotron Radiation*); however, if you intend to submit to *Acta Crystallographica Section C* or *E* or *IUCrData*, you should make sure that full publication checks are run on the final version of your CIF prior to submission.

Publication of your CIF in other journals

Please refer to the *Notes for Authors* of the relevant journal for any special instructions relating to CIF submission.

PLATON version of 06/07/2023; check.def file version of 30/06/2023

Datablock fgcupch0128_0m - ellipsoid plot

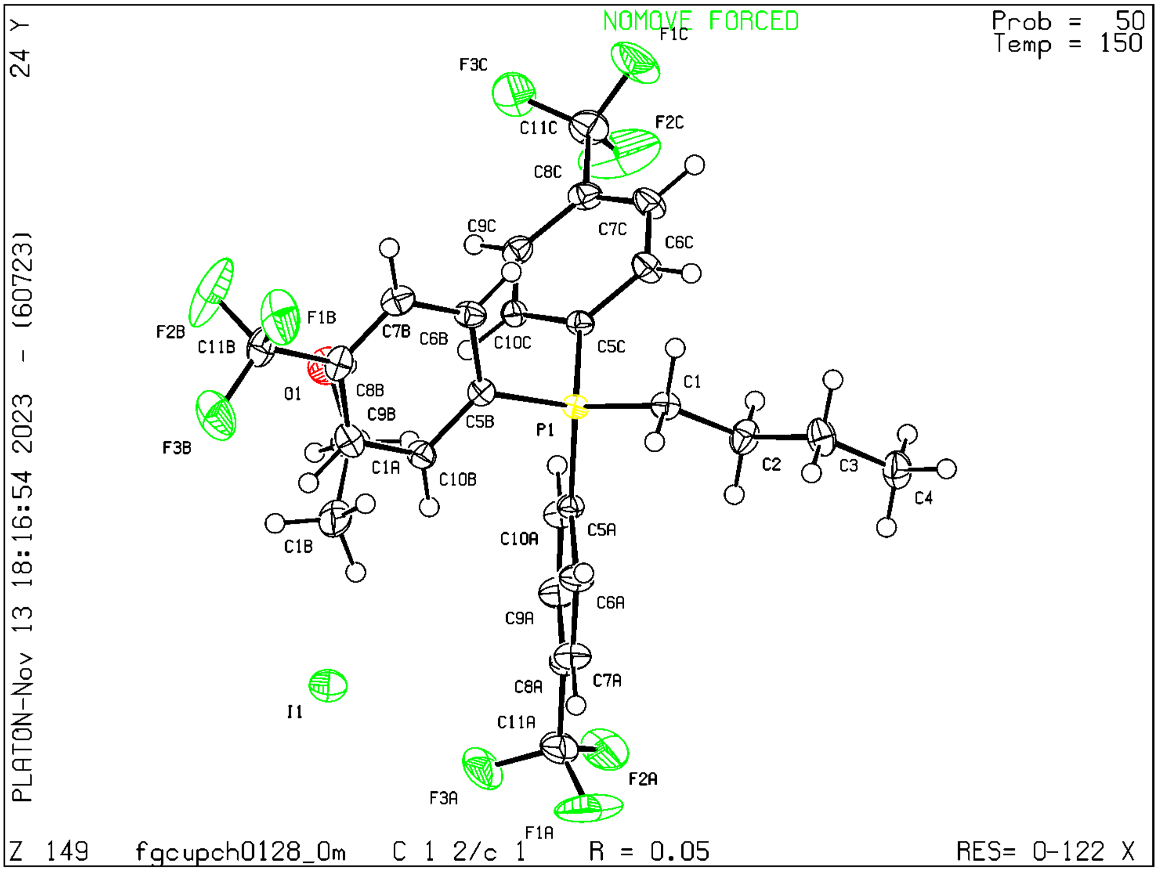

Supplement: Supplementary file 2 — jp4c02196_si_002.zip [file jp4c02196_si_002.zip › 4CF3 Iodo checkcif.pdf]
