# Supplementary material for: Planting the Seeds of a Decision Tree for Ionic Liquids: Steric and Electronic Impacts on Melting Points of Triarylphosponium Ionic Liquids
Source: J Phys Chem B. 2024 Jun 7;128(24):5895–907. doi: 10.1021/acs.jpcb.4c02196 (PMC11194809; doi:10.1021/acs.jpcb.4c02196)

checkCIF/PLATON report

Structure factors have been supplied for datablock(s) fgcupch0114_0mx

THIS REPORT IS FOR GUIDANCE ONLY. IF USED AS PART OF A REVIEW PROCEDURE FOR PUBLICATION, IT SHOULD NOT REPLACE THE EXPERTISE OF AN EXPERIENCED CRYSTALLOGRAPHIC REFEREE.

No syntax errors found. CIF dictionary Interpreting this report

Datablock: fgcupch0114_0mx

Bond precision:	C-C = 0.0018 A		Wavelength=0.71073
Cell:	a=8.8186(3)	b=10.6052(4)	c=15.5757(6)
	alpha=90.307(1)	beta=96.667(1)	gamma=105.561(1)
Temperature:	150 K		

	Calculated	Reported
Volume	1392.79(9)	1392.79(9)
Space group	P -1	P -1
Hall group	-P 1	-P 1
Moiety formula	C22 H21 F3 P, C2 F6 N O4 S2	C2 F6 N O4 S2, C22 H21 F3 P
Sum formula	C24 H21 F9 N O4 P S2	C24 H21 F9 N O4 P S2
Mr	653.51	653.51
Dx,g cm-3	1.558	1.558
Z	2	2
Mu (mm-1)	0.341	0.341
F000	664.0	664.0
F000'	665.20	
h,k,lmax	12,15,22	12,15,22
Nref	8614	8561
Tmin,Tmax	0.919,0.944	0.710,0.746
Tmin'	0.814	

Correction method= # Reported T Limits: Tmin=0.710 Tmax=0.746
AbsCorr = MULTI-SCAN

Data completeness= 0.994 Theta(max)= 30.657

R(reflections)= 0.0345(7300) wR2(reflections)= 0.0934(8561)

S = 1.042 Npar= 506

The following ALERTS were generated. Each ALERT has the format
test-name_ALERT_alert-type_alert-level.
Click on the hyperlinks for more details of the test.

Alert level C

```
PLAT042_ALERT_1_C Calc. and Reported MoietyFormula Strings  Differ     Please Check
PLAT911_ALERT_3_C Missing FCF Refl Between Thmin & STh/L=    0.600          7 Report
PLAT918_ALERT_3_C Reflection(s) with I(obs) much Smaller I(calc) .          1 Check
```

Alert level G

```
PLAT063_ALERT_4_G Crystal Size Possibly too Large for Beam Size ..       0.61 mm
PLAT154_ALERT_1_G The s.u.'s on the Cell Angles are Equal ..(Note)      0.001 Degree
PLAT187_ALERT_4_G The CIF-Embedded .res File Contains RIGU Records          2 Report
PLAT300_ALERT_4_G Atom Site Occupancy of S1A        Constrained at        0.5 Check
PLAT300_ALERT_4_G Atom Site Occupancy of S2A        Constrained at        0.5 Check
PLAT300_ALERT_4_G Atom Site Occupancy of F2A        Constrained at        0.5 Check
PLAT300_ALERT_4_G Atom Site Occupancy of F3A        Constrained at        0.5 Check
PLAT300_ALERT_4_G Atom Site Occupancy of F4A        Constrained at        0.5 Check
PLAT300_ALERT_4_G Atom Site Occupancy of F5A        Constrained at        0.5 Check
PLAT300_ALERT_4_G Atom Site Occupancy of F6A        Constrained at        0.5 Check
PLAT300_ALERT_4_G Atom Site Occupancy of F7A        Constrained at        0.5 Check
PLAT300_ALERT_4_G Atom Site Occupancy of O1A        Constrained at        0.5 Check
PLAT300_ALERT_4_G Atom Site Occupancy of O2A        Constrained at        0.5 Check
PLAT300_ALERT_4_G Atom Site Occupancy of O3A        Constrained at        0.5 Check
PLAT300_ALERT_4_G Atom Site Occupancy of O4A        Constrained at        0.5 Check
PLAT300_ALERT_4_G Atom Site Occupancy of N1A        Constrained at        0.5 Check
PLAT300_ALERT_4_G Atom Site Occupancy of C11A       Constrained at        0.5 Check
PLAT300_ALERT_4_G Atom Site Occupancy of C12A       Constrained at        0.5 Check
PLAT300_ALERT_4_G Atom Site Occupancy of S1B        Constrained at        0.5 Check
PLAT300_ALERT_4_G Atom Site Occupancy of S2B        Constrained at        0.5 Check
PLAT300_ALERT_4_G Atom Site Occupancy of F2B        Constrained at        0.5 Check
PLAT300_ALERT_4_G Atom Site Occupancy of F3B        Constrained at        0.5 Check
PLAT300_ALERT_4_G Atom Site Occupancy of F4B        Constrained at        0.5 Check
PLAT300_ALERT_4_G Atom Site Occupancy of F5B        Constrained at        0.5 Check
PLAT300_ALERT_4_G Atom Site Occupancy of F6B        Constrained at        0.5 Check
PLAT300_ALERT_4_G Atom Site Occupancy of F7B        Constrained at        0.5 Check
PLAT300_ALERT_4_G Atom Site Occupancy of O1B        Constrained at        0.5 Check
PLAT300_ALERT_4_G Atom Site Occupancy of O2B        Constrained at        0.5 Check
PLAT300_ALERT_4_G Atom Site Occupancy of O3B        Constrained at        0.5 Check
PLAT300_ALERT_4_G Atom Site Occupancy of O4B        Constrained at        0.5 Check
PLAT300_ALERT_4_G Atom Site Occupancy of N1B        Constrained at        0.5 Check
PLAT300_ALERT_4_G Atom Site Occupancy of C11B       Constrained at        0.5 Check
PLAT300_ALERT_4_G Atom Site Occupancy of C12B       Constrained at        0.5 Check
PLAT302_ALERT_4_G Anion/Solvent/Minor-Residue Disorder (Resd  2  )       100% Note
PLAT302_ALERT_4_G Anion/Solvent/Minor-Residue Disorder (Resd  3  )       100% Note
PLAT304_ALERT_4_G Non-Integer Number of Atoms in ..... (Resd  2  )       7.50 Check
PLAT304_ALERT_4_G Non-Integer Number of Atoms in ..... (Resd  3  )       7.50 Check
PLAT432_ALERT_2_G Short Inter X...Y Contact  F6A      ..C8C      .       2.96 Ang.
                                              1-x,1-y,1-z  =      2_666 Check
PLAT789_ALERT_4_G Atoms with Negative _atom_site_disorder_group  #         30 Check
PLAT802_ALERT_4_G CIF Input Record(s) with more than 80 Characters          1 Info
PLAT811_ALERT_5_G No ADDSYM Analysis: Too Many Excluded Atoms ....          ! Info
PLAT822_ALERT_4_G CIF-embedded .res Contains Negative PART Numbers          1 Check
PLAT860_ALERT_3_G Number of Least-Squares Restraints ............         218 Note
```

```
PLAT910_ALERT_3_G Missing # of FCF Reflection(s) Below Theta(Min).          4 Note
PLAT912_ALERT_4_G Missing # of FCF Reflections Above STh/L=  0.600         43 Note
PLAT933_ALERT_2_G Number of HKL-OMIT Records in Embedded .res File          4 Note
PLAT941_ALERT_3_G Average HKL Measurement Multiplicity ..........         4.3 Low
PLAT978_ALERT_2_G Number C-C Bonds with Positive Residual Density.         16 Info
```

```
   0 ALERT level A = Most likely a serious problem - resolve or explain
   0 ALERT level B = A potentially serious problem, consider carefully
   3 ALERT level C = Check. Ensure it is not caused by an omission or oversight
  48 ALERT level G = General information/check it is not something unexpected

   2 ALERT type 1 CIF construction/syntax error, inconsistent or missing data
   3 ALERT type 2 Indicator that the structure model may be wrong or deficient
   5 ALERT type 3 Indicator that the structure quality may be low
  40 ALERT type 4 Improvement, methodology, query or suggestion
   1 ALERT type 5 Informative message, check
```

It is advisable to attempt to resolve as many as possible of the alerts in all categories. Often the minor alerts point to easily fixed oversights, errors and omissions in your CIF or refinement strategy, so attention to these fine details can be worthwhile. In order to resolve some of the more serious problems it may be necessary to carry out additional measurements or structure refinements. However, the purpose of your study may justify the reported deviations and the more serious of these should normally be commented upon in the discussion or experimental section of a paper or in the "special_details" fields of the CIF. checkCIF was carefully designed to identify outliers and unusual parameters, but every test has its limitations and alerts that are not important in a particular case may appear. Conversely, the absence of alerts does not guarantee there are no aspects of the results needing attention. It is up to the individual to critically assess their own results and, if necessary, seek expert advice.

Publication of your CIF in IUCr journals

A basic structural check has been run on your CIF. These basic checks will be run on all CIFs submitted for publication in IUCr journals (*Acta Crystallographica*, *Journal of Applied Crystallography*, *Journal of Synchrotron Radiation*); however, if you intend to submit to *Acta Crystallographica Section C* or *E* or *IUCrData*, you should make sure that full publication checks are run on the final version of your CIF prior to submission.

Publication of your CIF in other journals

Please refer to the *Notes for Authors* of the relevant journal for any special instructions relating to CIF submission.

PLATON version of 06/07/2023; check.def file version of 30/06/2023

Datablock fgcupch0114_0mx - ellipsoid plot

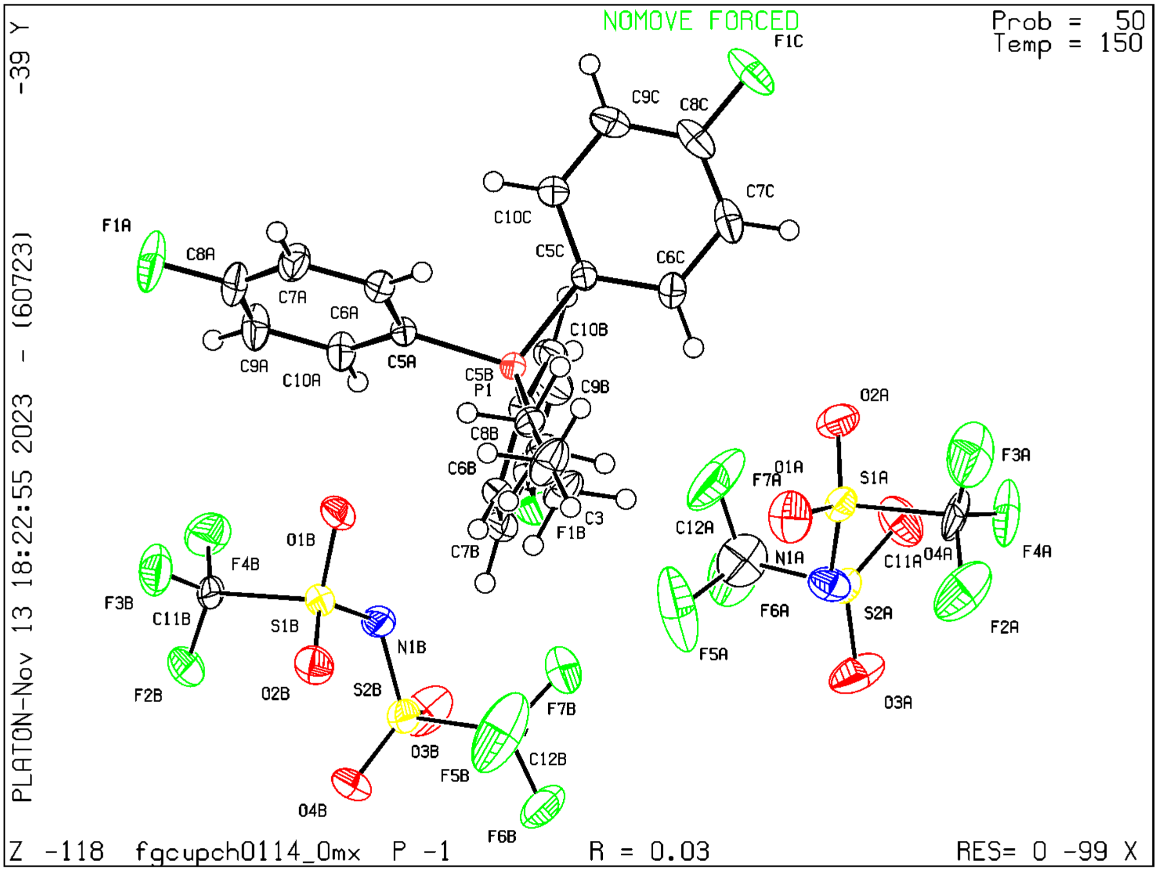

Supplement: Supplementary file 2 — jp4c02196_si_002.zip [file jp4c02196_si_002.zip › 4F NTf2 checkcif.pdf]
