# Supplementary material for: Planting the Seeds of a Decision Tree for Ionic Liquids: Steric and Electronic Impacts on Melting Points of Triarylphosponium Ionic Liquids
Source: J Phys Chem B. 2024 Jun 7;128(24):5895–907. doi: 10.1021/acs.jpcb.4c02196 (PMC11194809; doi:10.1021/acs.jpcb.4c02196)

checkCIF/PLATON report

Structure factors have been supplied for datablock(s) fgcupch0116_0m

THIS REPORT IS FOR GUIDANCE ONLY. IF USED AS PART OF A REVIEW PROCEDURE FOR PUBLICATION, IT SHOULD NOT REPLACE THE EXPERTISE OF AN EXPERIENCED CRYSTALLOGRAPHIC REFEREE.

No syntax errors found.   CIF dictionary   Interpreting this report

Datablock: fgcupch0116_0m

Bond precision: C-C = 0.0022 A   Wavelength=0.71073

Cell:	a=10.4479(7)	b=11.1180(7)	c=14.5534(9)
	alpha=82.221(2)	beta=86.383(3)	gamma=63.915(2)
Temperature:	150 K		

	Calculated	Reported
Volume	1504.34(17)	1504.34(17)
Space group	P -1	P -1
Hall group	-P 1	-P 1
Moiety formula	C25 H30 P, C2 F6 N O4 S2	C2 F6 N O4 S2, C25 H30 P
Sum formula	C27 H30 F6 N O4 P S2	C27 H30 F6 N O4 P S2
Mr	641.61	641.61
Dx,g cm-3	1.416	1.416
Z	2	2
Mu (mm-1)	0.301	0.301
F000	664.0	664.0
F000'	665.11	
h,k,lmax	14,15,20	14,15,20
Nref	9228	9184
Tmin,Tmax	0.935,0.980	0.707,0.746
Tmin'	0.883	

Correction method= # Reported T Limits: Tmin=0.707 Tmax=0.746
AbsCorr = MULTI-SCAN

Data completeness= 0.995   Theta(max)= 30.558

R(reflections)= 0.0407(7880)   wR2(reflections)= 0.1035(9184)

S = 1.069   Npar= 394

The following ALERTS were generated. Each ALERT has the format
test-name_ALERT_alert-type_alert-level.
Click on the hyperlinks for more details of the test.

Alert level C

```
PLAT042_ALERT_1_C Calc. and Reported MoietyFormula Strings  Differ     Please Check
PLAT230_ALERT_2_C Hirshfeld Test Diff for    S1       --C12      .       5.1 s.u.
PLAT906_ALERT_3_C Large K Value in the Analysis of Variance ......      2.620 Check
PLAT911_ALERT_3_C Missing FCF Refl Between Thmin & STh/L=    0.600          7 Report
```

Alert level G

```
PLAT171_ALERT_4_G The CIF-Embedded .res File Contains EADP Records          1 Report
PLAT230_ALERT_2_G Hirshfeld Test Diff for    P1       --C1'      .       5.4 s.u.
PLAT230_ALERT_2_G Hirshfeld Test Diff for    C1'      --C2'      .      18.7 s.u.
PLAT242_ALERT_2_G Low    'MainMol' Ueq as Compared to Neighbors of       C12 Check
PLAT242_ALERT_2_G Low    'MainMol' Ueq as Compared to Neighbors of       C13 Check
PLAT301_ALERT_3_G Main Residue  Disorder ..............(Resd  1  )       15% Note
PLAT410_ALERT_2_G Short Intra H...H Contact  H6B      ..H1"A     .      2.11 Ang.
                                                      x,y,z  =      1_555 Check
PLAT720_ALERT_4_G Number of Unusual/Non-Standard Labels ..........        18 Note
PLAT802_ALERT_4_G CIF Input Record(s) with more than 80 Characters         1 Info
PLAT910_ALERT_3_G Missing # of FCF Reflection(s) Below Theta(Min).         3 Note
PLAT912_ALERT_4_G Missing # of FCF Reflections Above STh/L=  0.600        34 Note
PLAT933_ALERT_2_G Number of HKL-OMIT Records in Embedded .res File         6 Note
PLAT941_ALERT_3_G Average HKL Measurement Multiplicity ..........        3.5 Low
PLAT978_ALERT_2_G Number C-C Bonds with Positive Residual Density.        15 Info
```

0 **ALERT level A** = Most likely a serious problem - resolve or explain
0 **ALERT level B** = A potentially serious problem, consider carefully
4 **ALERT level C** = Check. Ensure it is not caused by an omission or oversight
14 **ALERT level G** = General information/check it is not something unexpected

1 ALERT type 1 CIF construction/syntax error, inconsistent or missing data
8 ALERT type 2 Indicator that the structure model may be wrong or deficient
5 ALERT type 3 Indicator that the structure quality may be low
4 ALERT type 4 Improvement, methodology, query or suggestion
0 ALERT type 5 Informative message, check

It is advisable to attempt to resolve as many as possible of the alerts in all categories. Often the minor alerts point to easily fixed oversights, errors and omissions in your CIF or refinement strategy, so attention to these fine details can be worthwhile. In order to resolve some of the more serious problems it may be necessary to carry out additional measurements or structure refinements. However, the purpose of your study may justify the reported deviations and the more serious of these should normally be commented upon in the discussion or experimental section of a paper or in the "special_details" fields of the CIF. checkCIF was carefully designed to identify outliers and unusual parameters, but every test has its limitations and alerts that are not important in a particular case may appear. Conversely, the absence of alerts does not guarantee there are no aspects of the results needing attention. It is up to the individual to critically assess their own results and, if necessary, seek expert advice.

Publication of your CIF in IUCr journals

A basic structural check has been run on your CIF. These basic checks will be run on all CIFs submitted for publication in IUCr journals (*Acta Crystallographica*, *Journal of Applied Crystallography*, *Journal of Synchrotron Radiation*); however, if you intend to submit to *Acta Crystallographica Section C* or *E* or *IUCrData*, you should make sure that full publication checks are run on the final version of your CIF prior to submission.

Publication of your CIF in other journals

Please refer to the *Notes for Authors* of the relevant journal for any special instructions relating to CIF submission.

PLATON version of 06/07/2023; check.def file version of 30/06/2023

Datablock fgcupch0116_0m - ellipsoid plot

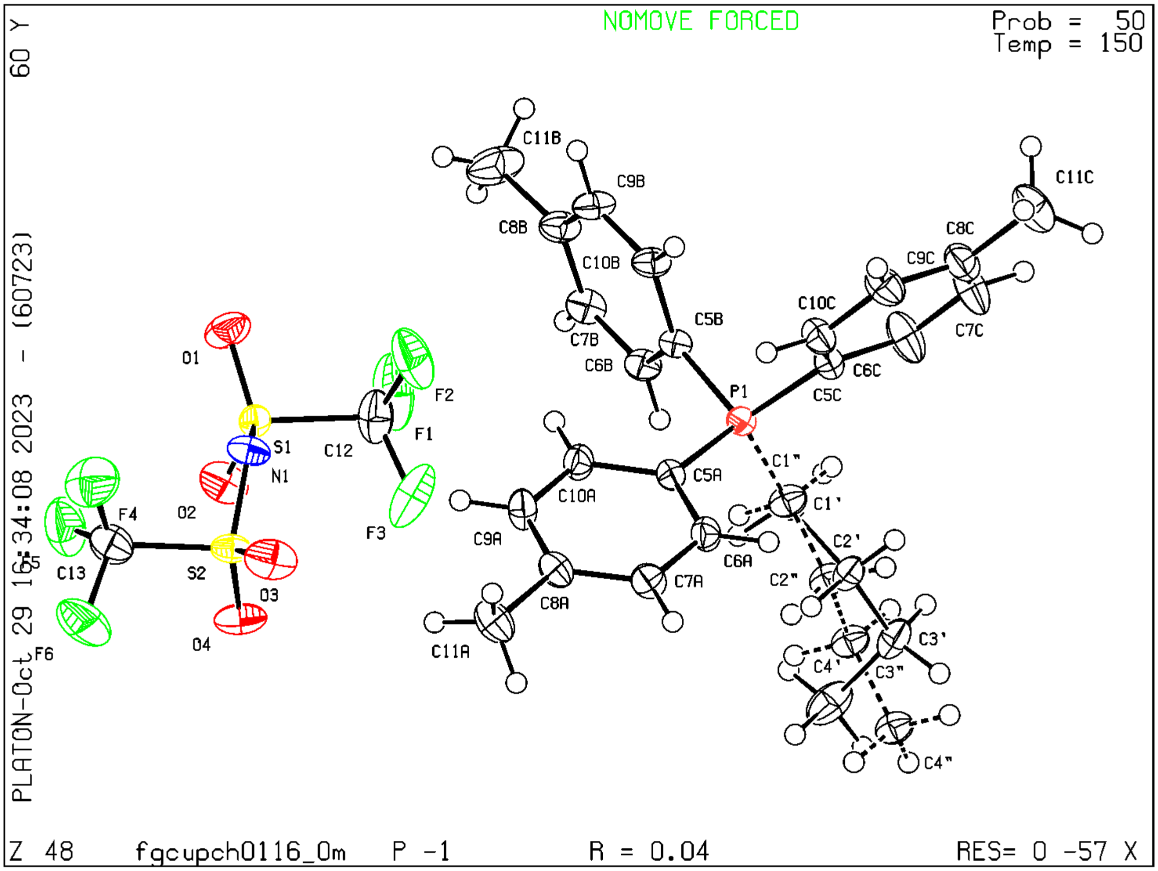

Supplement: Supplementary file 2 — jp4c02196_si_002.zip [file jp4c02196_si_002.zip › 4Me NTf2 checkcif.pdf]
