# Supplementary material for: Planting the Seeds of a Decision Tree for Ionic Liquids: Steric and Electronic Impacts on Melting Points of Triarylphosponium Ionic Liquids
Source: J Phys Chem B. 2024 Jun 7;128(24):5895–907. doi: 10.1021/acs.jpcb.4c02196 (PMC11194809; doi:10.1021/acs.jpcb.4c02196)

checkCIF/PLATON report

Structure factors have been supplied for datablock(s) C4_MeOTPPI_0m

THIS REPORT IS FOR GUIDANCE ONLY. IF USED AS PART OF A REVIEW PROCEDURE FOR PUBLICATION, IT SHOULD NOT REPLACE THE EXPERTISE OF AN EXPERIENCED CRYSTALLOGRAPHIC REFEREE.

No syntax errors found. CIF dictionary Interpreting this report

Datablock: C4_MeOTPPI_0m

Bond precision: C-C = 0.0041 A Wavelength=0.71073

Cell:	a=9.7909(5)	b=12.4942(7)	c=19.8422(8)
	alpha=90	beta=93.155(2)	gamma=90
Temperature:	150 K		

	Calculated	Reported
Volume	2423.6(2)	2423.6(2)
Space group	P 21/n	P 21/n
Hall group	-P 2yn	-P 2yn
Moiety formula	C25 H30 O3 P, I	?
Sum formula	C25 H30 I O3 P	C25 H30 I O3 P
Mr	536.36	536.36
Dx,g cm-3	1.470	1.470
Z	4	4
Mu (mm-1)	1.410	1.410
F000	1088.0	1088.0
F000’	1086.90	
h,k,lmax	15,19,30	15,19,30
Nref	9261	9050
Tmin,Tmax	0.673,0.844	0.550,0.747
Tmin’	0.462	

Correction method= # Reported T Limits: Tmin=0.550 Tmax=0.747
AbsCorr = MULTI-SCAN

Data completeness= 0.977 Theta(max)= 33.171

R(reflections)= 0.0494(6555) wR2(reflections)= 0.1358(9050)

S = 1.025 Npar= 349

The following ALERTS were generated. Each ALERT has the format
test-name_ALERT_alert-type_alert-level.
Click on the hyperlinks for more details of the test.

Alert level C

```
PLAT905_ALERT_3_C Negative K value in the Analysis of Variance ...     -0.250 Report
PLAT910_ALERT_3_C Missing # of FCF Reflection(s) Below Theta(Min).          5 Note
PLAT911_ALERT_3_C Missing FCF Refl Between Thmin & STh/L=    0.600         22 Report
PLAT913_ALERT_3_C Missing # of Very Strong Reflections in FCF ....          9 Note
PLAT971_ALERT_2_C Check Calcd Resid. Dens.  1.50Ang From I1              2.12 eA-3
PLAT972_ALERT_2_C Check Calcd Resid. Dens.  0.72Ang From I1             -1.99 eA-3
```

Alert level G

```
PLAT002_ALERT_2_G Number of Distance or Angle Restraints on AtSite         25 Note
PLAT003_ALERT_2_G Number of Uiso or Uij Restrained non-H Atoms ...         16 Report
PLAT175_ALERT_4_G The CIF-Embedded .res File Contains SAME Records          2 Report
PLAT176_ALERT_4_G The CIF-Embedded .res File Contains SADI Records          1 Report
PLAT178_ALERT_4_G The CIF-Embedded .res File Contains SIMU Records          1 Report
PLAT188_ALERT_3_G A Non-default SIMU Restraint Value has been used     0.0100 Report
PLAT301_ALERT_3_G Main Residue  Disorder .............(Resd  1  )         28% Note
PLAT811_ALERT_5_G No ADDSYM Analysis: Too Many Excluded Atoms ....          ! Info
PLAT860_ALERT_3_G Number of Least-Squares Restraints ............         304 Note
PLAT899_ALERT_4_G SHELXL2018 is Deprecated and Succeeded by SHELXL     2019/3 Note
PLAT912_ALERT_4_G Missing # of FCF Reflections Above STh/L=  0.600        185 Note
PLAT933_ALERT_2_G Number of HKL-OMIT Records in Embedded .res File          2 Note
PLAT941_ALERT_3_G Average HKL Measurement Multiplicity ..........         4.4 Low
PLAT978_ALERT_2_G Number C-C Bonds with Positive Residual Density.         10 Info
```

```
   0 ALERT level A = Most likely a serious problem - resolve or explain
   0 ALERT level B = A potentially serious problem, consider carefully
   6 ALERT level C = Check. Ensure it is not caused by an omission or oversight
  14 ALERT level G = General information/check it is not something unexpected

   0 ALERT type 1 CIF construction/syntax error, inconsistent or missing data
   6 ALERT type 2 Indicator that the structure model may be wrong or deficient
   8 ALERT type 3 Indicator that the structure quality may be low
   5 ALERT type 4 Improvement, methodology, query or suggestion
   1 ALERT type 5 Informative message, check
```

It is advisable to attempt to resolve as many as possible of the alerts in all categories. Often the minor alerts point to easily fixed oversights, errors and omissions in your CIF or refinement strategy, so attention to these fine details can be worthwhile. In order to resolve some of the more serious problems it may be necessary to carry out additional measurements or structure refinements. However, the purpose of your study may justify the reported deviations and the more serious of these should normally be commented upon in the discussion or experimental section of a paper or in the "special_details" fields of the CIF. checkCIF was carefully designed to identify outliers and unusual parameters, but every test has its limitations and alerts that are not important in a particular case may appear. Conversely, the absence of alerts does not guarantee there are no aspects of the results needing attention. It is up to the individual to critically assess their own results and, if necessary, seek expert advice.

Publication of your CIF in IUCr journals

A basic structural check has been run on your CIF. These basic checks will be run on all CIFs submitted for publication in IUCr journals (*Acta Crystallographica*, *Journal of Applied Crystallography*, *Journal of Synchrotron Radiation*); however, if you intend to submit to *Acta Crystallographica Section C* or *E* or *IUCrData*, you should make sure that full publication checks are run on the final version of your CIF prior to submission.

Publication of your CIF in other journals

Please refer to the *Notes for Authors* of the relevant journal for any special instructions relating to CIF submission.

PLATON version of 06/07/2023; check.def file version of 30/06/2023

Datablock C4_MeOTPPI_0m - ellipsoid plot

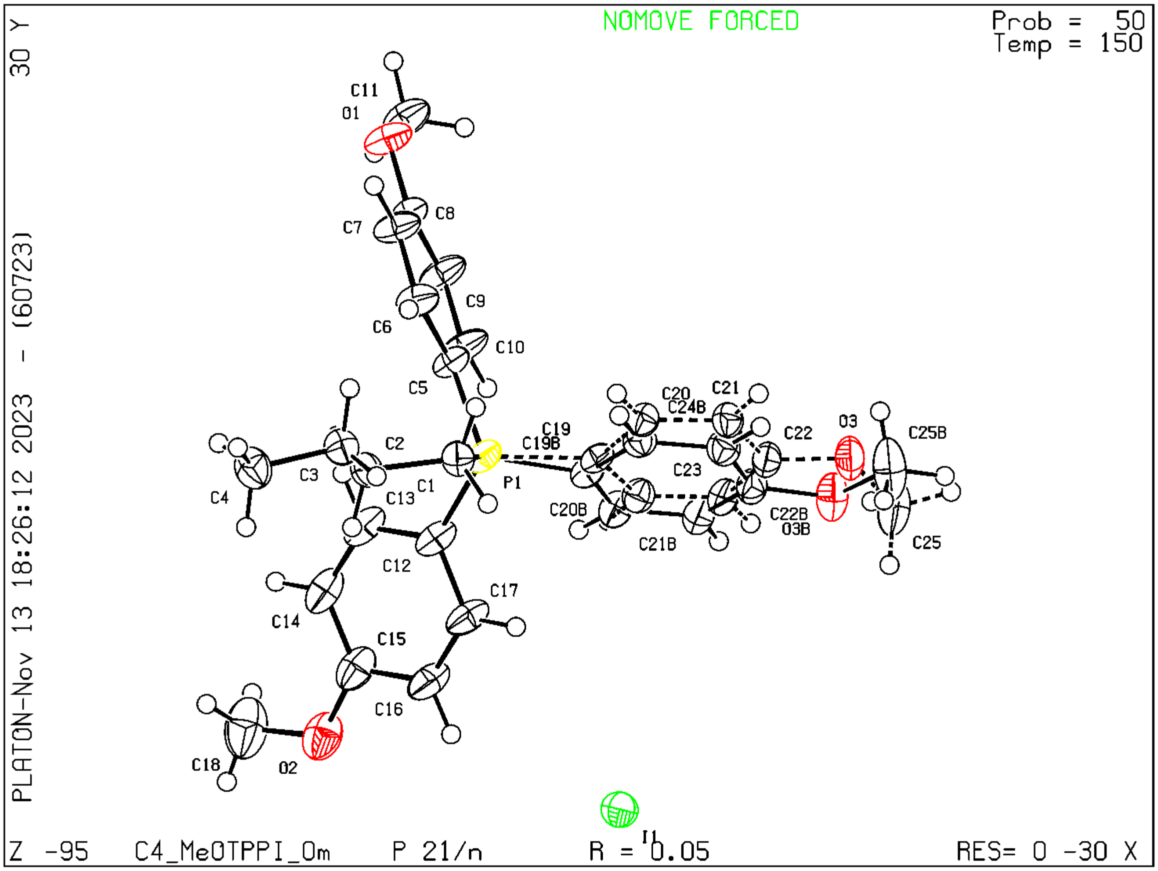

Supplement: Supplementary file 2 — jp4c02196_si_002.zip [file jp4c02196_si_002.zip › 4MeO Iodo Polymorph 1 checkcif.pdf]
