# Supplementary material for: Planting the Seeds of a Decision Tree for Ionic Liquids: Steric and Electronic Impacts on Melting Points of Triarylphosponium Ionic Liquids
Source: J Phys Chem B. 2024 Jun 7;128(24):5895–907. doi: 10.1021/acs.jpcb.4c02196 (PMC11194809; doi:10.1021/acs.jpcb.4c02196)

checkCIF/PLATON report

Structure factors have been supplied for datablock(s) c4_4meotpp_tf2n_0m

THIS REPORT IS FOR GUIDANCE ONLY. IF USED AS PART OF A REVIEW PROCEDURE FOR PUBLICATION, IT SHOULD NOT REPLACE THE EXPERTISE OF AN EXPERIENCED CRYSTALLOGRAPHIC REFEREE.

No syntax errors found. CIF dictionary Interpreting this report

Datablock: c4_4meotpp_tf2n_0m

```
Bond precision:   C-C = 0.0044 A          Wavelength=1.54178

Cell:              a=10.932(3)     b=22.657(7)         c=12.759(3)
                   alpha=90        beta=96.130(14)     gamma=90
Temperature:       150 K

                 Calculated                      Reported
Volume           3142.2(15)                      3142.2(14)
Space group      P 21/n                          P 1 21/n 1
Hall group       -P 2yn                          -P 2yn
Moiety formula   C25 H30 O3 P, C2 F6 N O4 S2     C2 F6 N O4 S2, C25 H30 O3 P
Sum formula      C27 H30 F6 N O7 P S2            C27 H30 F6 N O7 P S2
Mr               689.61                          689.61
Dx,g cm-3        1.458                           1.458
Z                4                               4
Mu (mm-1)        2.744                           2.744
F000             1424.0                          1424.0
F000'            1432.93
h,k,lmax         13,28,16                        13,28,16
Nref             6845                            6668
Tmin,Tmax        0.794,0.947                     0.636,0.754
Tmin'            0.739

Correction method= # Reported T Limits: Tmin=0.636 Tmax=0.754
AbsCorr = MULTI-SCAN

Data completeness= 0.974           Theta(max)= 79.790

R(reflections)= 0.0563( 4594)                          wR2(reflections)=
                                                       0.1551( 6668)
S = 1.035                    Npar= 401
```

The following ALERTS were generated. Each ALERT has the format
test-name_ALERT_alert-type_alert-level.
Click on the hyperlinks for more details of the test.

Alert level C

```
PLAT042_ALERT_1_C Calc. and Reported MoietyFormula Strings  Differ     Please Check
PLAT242_ALERT_2_C Low    'MainMol' Ueq as Compared to Neighbors of         S1 Check
PLAT340_ALERT_3_C Low Bond Precision on  C-C Bonds ...............    0.00438 Ang.
PLAT906_ALERT_3_C Large K Value in the Analysis of Variance ......      3.900 Check
PLAT911_ALERT_3_C Missing FCF Refl Between Thmin & STh/L=    0.600          9 Report
```

Alert level G

```
PLAT242_ALERT_2_G Low    'MainMol' Ueq as Compared to Neighbors of        C12 Check
PLAT242_ALERT_2_G Low    'MainMol' Ueq as Compared to Neighbors of        C13 Check
PLAT912_ALERT_4_G Missing # of FCF Reflections Above STh/L=  0.600        168 Note
PLAT933_ALERT_2_G Number of HKL-OMIT Records in Embedded .res File          1 Note
PLAT941_ALERT_3_G Average HKL Measurement Multiplicity ...........        4.8 Low
PLAT978_ALERT_2_G Number C-C Bonds with Positive Residual Density.          3 Info
```

0 **ALERT level A** = Most likely a serious problem - resolve or explain
0 **ALERT level B** = A potentially serious problem, consider carefully
5 **ALERT level C** = Check. Ensure it is not caused by an omission or oversight
6 **ALERT level G** = General information/check it is not something unexpected

1 ALERT type 1 CIF construction/syntax error, inconsistent or missing data
5 ALERT type 2 Indicator that the structure model may be wrong or deficient
4 ALERT type 3 Indicator that the structure quality may be low
1 ALERT type 4 Improvement, methodology, query or suggestion
0 ALERT type 5 Informative message, check

It is advisable to attempt to resolve as many as possible of the alerts in all categories. Often the minor alerts point to easily fixed oversights, errors and omissions in your CIF or refinement strategy, so attention to these fine details can be worthwhile. In order to resolve some of the more serious problems it may be necessary to carry out additional measurements or structure refinements. However, the purpose of your study may justify the reported deviations and the more serious of these should normally be commented upon in the discussion or experimental section of a paper or in the "special_details" fields of the CIF. checkCIF was carefully designed to identify outliers and unusual parameters, but every test has its limitations and alerts that are not important in a particular case may appear. Conversely, the absence of alerts does not guarantee there are no aspects of the results needing attention. It is up to the individual to critically assess their own results and, if necessary, seek expert advice.

Publication of your CIF in IUCr journals

A basic structural check has been run on your CIF. These basic checks will be run on all CIFs submitted for publication in IUCr journals (*Acta Crystallographica*, *Journal of Applied Crystallography*, *Journal of Synchrotron Radiation*); however, if you intend to submit to *Acta Crystallographica Section C* or *E* or *IUCrData*, you should make sure that full publication checks are run on the final version of your CIF prior to submission.

Publication of your CIF in other journals

Please refer to the *Notes for Authors* of the relevant journal for any special instructions relating to CIF submission.

PLATON version of 06/07/2023; check.def file version of 30/06/2023

Datablock c4_4meotpp_tf2n_0m - ellipsoid plot

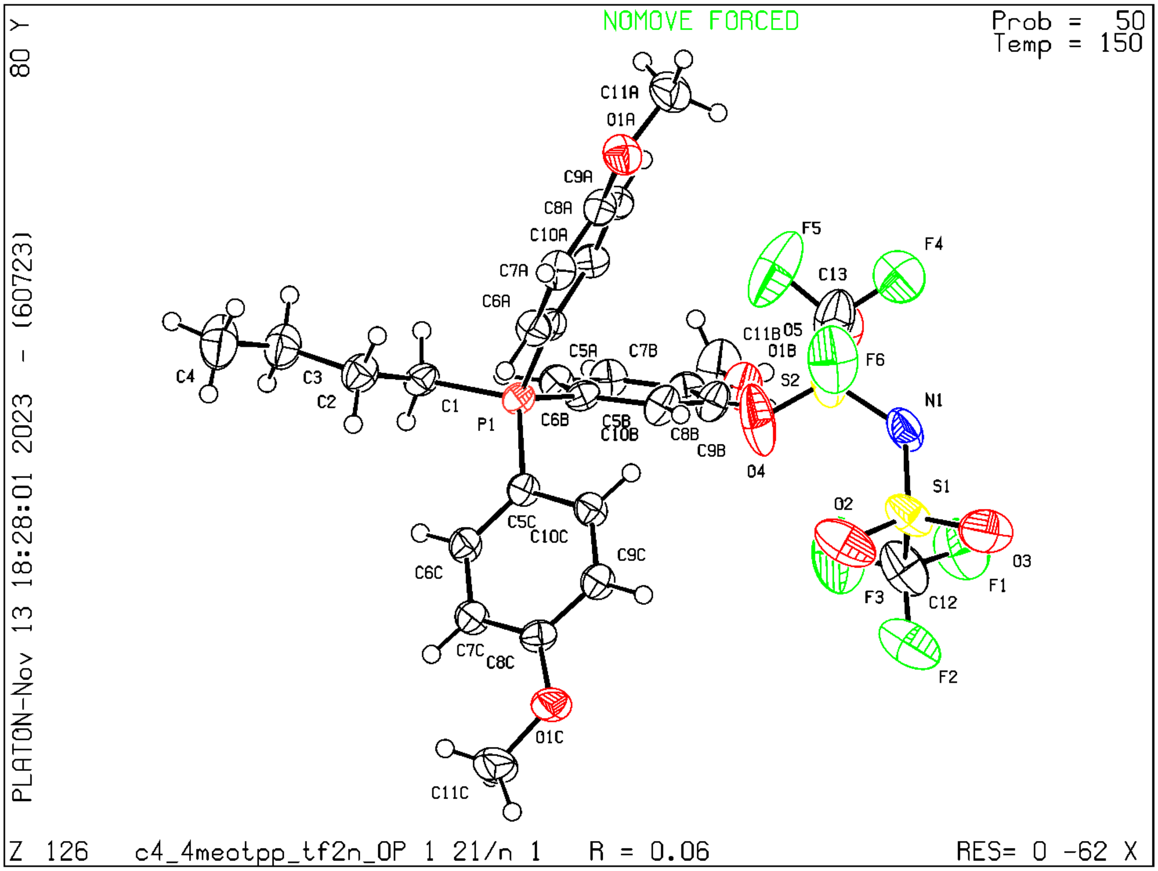

Supplement: Supplementary file 2 — jp4c02196_si_002.zip [file jp4c02196_si_002.zip › 4MeO NTf2 checkcif.pdf]
