# Supplementary material for: Planting the Seeds of a Decision Tree for Ionic Liquids: Steric and Electronic Impacts on Melting Points of Triarylphosponium Ionic Liquids
Source: J Phys Chem B. 2024 Jun 7;128(24):5895–907. doi: 10.1021/acs.jpcb.4c02196 (PMC11194809; doi:10.1021/acs.jpcb.4c02196)

checkCIF/PLATON report

Structure factors have been supplied for datablock(s) C4TFPI_0m

THIS REPORT IS FOR GUIDANCE ONLY. IF USED AS PART OF A REVIEW PROCEDURE FOR PUBLICATION, IT SHOULD NOT REPLACE THE EXPERTISE OF AN EXPERIENCED CRYSTALLOGRAPHIC REFEREE.

No syntax errors found. CIF dictionary Interpreting this report

Datablock: C4TFPI_0m

Bond precision: C-C = 0.0147 A Wavelength=0.71073

Cell:	a=8.834(3)	b=46.773(14)	c=9.6236(18)
	alpha=90	beta=117.224(14)	gamma=90
Temperature:	150 K		

	Calculated	Reported
Volume	3535.9(18)	3535.9(18)
Space group	P 21	P 21
Hall group	P 2yb	P 2yb
Moiety formula	C16 H18 O3 P, I	?
Sum formula	C16 H18 I O3 P	C16 H18 I O3 P
Mr	416.17	416.17
Dx,g cm-3	1.564	1.564
Z	8	8
Mu (mm-1)	1.908	1.908
F000	1648.0	1648.0
F000’	1645.65	
h,k,lmax	11,62,12	11,62,12
Nref	17515[8861]	16517
Tmin,Tmax	0.780,0.842	0.620,0.746
Tmin’	0.780	

Correction method= # Reported T Limits: Tmin=0.620 Tmax=0.746
AbsCorr = MULTI-SCAN

Data completeness= 1.86/0.94 Theta(max)= 28.258

R(reflections)= 0.0400(14645) wR2(reflections)= 0.0985(16517)

S = 1.043 Npar= 1035

The following ALERTS were generated. Each ALERT has the format
test-name_ALERT_alert-type_alert-level.
Click on the hyperlinks for more details of the test.

Alert level C

```
PLAT342_ALERT_3_C Low Bond Precision on  C-C Bonds ...............    0.01473 Ang.
PLAT911_ALERT_3_C Missing FCF Refl Between Thmin & STh/L=    0.600         49 Report
```

Alert level G

```
PLAT002_ALERT_2_G Number of Distance or Angle Restraints on AtSite         80 Note
PLAT003_ALERT_2_G Number of Uiso or Uij Restrained non-H Atoms ...        110 Report
PLAT174_ALERT_4_G The CIF-Embedded .res File Contains FLAT Records          1 Report
PLAT175_ALERT_4_G The CIF-Embedded .res File Contains SAME Records          4 Report
PLAT176_ALERT_4_G The CIF-Embedded .res File Contains SADI Records          1 Report
PLAT178_ALERT_4_G The CIF-Embedded .res File Contains SIMU Records          2 Report
PLAT301_ALERT_3_G Main Residue  Disorder ..............(Resd  1  )        50% Note
PLAT301_ALERT_3_G Main Residue  Disorder ..............(Resd  4  )       100% Note
PLAT301_ALERT_3_G Main Residue  Disorder ..............(Resd  5  )       100% Note
PLAT304_ALERT_4_G Non-Integer Number of Atoms in ..... (Resd  4  )      30.40 Check
PLAT304_ALERT_4_G Non-Integer Number of Atoms in ..... (Resd  5  )       7.60 Check
PLAT398_ALERT_2_G Deviating  C-O-C   Angle From 120 for O1_3     .      104.6 Degree
PLAT398_ALERT_2_G Deviating  C-O-C   Angle From 120 for O3_3     .      106.5 Degree
PLAT398_ALERT_2_G Deviating  C-O-C   Angle From 120 for O2_3     .      106.2 Degree
PLAT398_ALERT_2_G Deviating  C-O-C   Angle From 120 for O2B_3    .      106.5 Degree
PLAT398_ALERT_2_G Deviating  C-O-C   Angle From 120 for O1_1     .      106.1 Degree
PLAT398_ALERT_2_G Deviating  C-O-C   Angle From 120 for O2_1     .      105.9 Degree
PLAT398_ALERT_2_G Deviating  C-O-C   Angle From 120 for O3_1     .      105.6 Degree
PLAT398_ALERT_2_G Deviating  C-O-C   Angle From 120 for O1_2     .      104.8 Degree
PLAT398_ALERT_2_G Deviating  C-O-C   Angle From 120 for O2_2     .      105.0 Degree
PLAT398_ALERT_2_G Deviating  C-O-C   Angle From 120 for O3_2     .      104.2 Degree
PLAT398_ALERT_2_G Deviating  C-O-C   Angle From 120 for O1_4     .      105.3 Degree
PLAT398_ALERT_2_G Deviating  C-O-C   Angle From 120 for O2_4     .      106.3 Degree
PLAT398_ALERT_2_G Deviating  C-O-C   Angle From 120 for O3_4     .      106.3 Degree
PLAT398_ALERT_2_G Deviating  C-O-C   Angle From 120 for O1B_4    .      105.7 Degree
PLAT398_ALERT_2_G Deviating  C-O-C   Angle From 120 for O2B_4    .      104.5 Degree
PLAT398_ALERT_2_G Deviating  C-O-C   Angle From 120 for O3B_4    .      106.1 Degree
PLAT720_ALERT_4_G Number of Unusual/Non-Standard Labels ..........        216 Note
PLAT811_ALERT_5_G No ADDSYM Analysis: Too Many Excluded Atoms ....          ! Info
PLAT860_ALERT_3_G Number of Least-Squares Restraints .............       1597 Note
PLAT899_ALERT_4_G SHELXL2018 is Deprecated and Succeeded by SHELXL     2019/3 Note
PLAT910_ALERT_3_G Missing # of FCF Reflection(s) Below Theta(Min).          3 Note
PLAT912_ALERT_4_G Missing # of FCF Reflections Above STh/L=  0.600        146 Note
PLAT933_ALERT_2_G Number of HKL-OMIT Records in Embedded .res File          1 Note
PLAT941_ALERT_3_G Average HKL Measurement Multiplicity ...........        3.5 Low
PLAT978_ALERT_2_G Number C-C Bonds with Positive Residual Density.          0 Info
```

0 **ALERT level A** = Most likely a serious problem - resolve or explain
0 **ALERT level B** = A potentially serious problem, consider carefully
2 **ALERT level C** = Check. Ensure it is not caused by an omission or oversight
36 **ALERT level G** = General information/check it is not something unexpected

0 ALERT type 1 CIF construction/syntax error, inconsistent or missing data

```
 20 ALERT type 2 Indicator that the structure model may be wrong or deficient
  8 ALERT type 3 Indicator that the structure quality may be low
  9 ALERT type 4 Improvement, methodology, query or suggestion
  1 ALERT type 5 Informative message, check
```

It is advisable to attempt to resolve as many as possible of the alerts in all categories. Often the minor alerts point to easily fixed oversights, errors and omissions in your CIF or refinement strategy, so attention to these fine details can be worthwhile. In order to resolve some of the more serious problems it may be necessary to carry out additional measurements or structure refinements. However, the purpose of your study may justify the reported deviations and the more serious of these should normally be commented upon in the discussion or experimental section of a paper or in the "special_details" fields of the CIF. checkCIF was carefully designed to identify outliers and unusual parameters, but every test has its limitations and alerts that are not important in a particular case may appear. Conversely, the absence of alerts does not guarantee there are no aspects of the results needing attention. It is up to the individual to critically assess their own results and, if necessary, seek expert advice.

Publication of your CIF in IUCr journals

A basic structural check has been run on your CIF. These basic checks will be run on all CIFs submitted for publication in IUCr journals (*Acta Crystallographica*, *Journal of Applied Crystallography*, *Journal of Synchrotron Radiation*); however, if you intend to submit to *Acta Crystallographica Section C* or *E* or *IUCrData*, you should make sure that full publication checks are run on the final version of your CIF prior to submission.

Publication of your CIF in other journals

Please refer to the *Notes for Authors* of the relevant journal for any special instructions relating to CIF submission.

PLATON version of 06/07/2023; check.def file version of 30/06/2023

Datablock C4TFPI_0m - ellipsoid plot

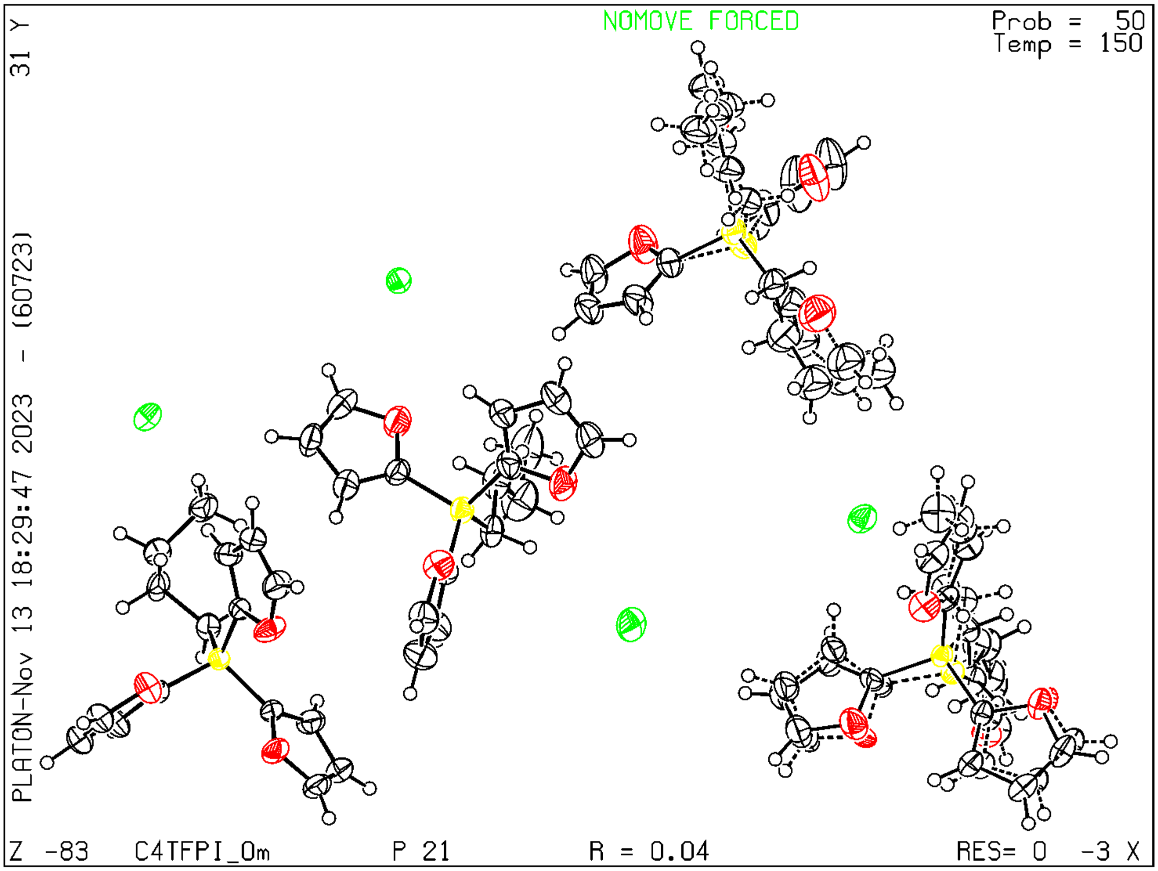

Supplement: Supplementary file 2 — jp4c02196_si_002.zip [file jp4c02196_si_002.zip › TFP Iodo checkcif.pdf]
