# Supplementary material for: Planting the Seeds of a Decision Tree for Ionic Liquids: Steric and Electronic Impacts on Melting Points of Triarylphosponium Ionic Liquids
Source: J Phys Chem B. 2024 Jun 7;128(24):5895–907. doi: 10.1021/acs.jpcb.4c02196 (PMC11194809; doi:10.1021/acs.jpcb.4c02196)
Supplement: Supplementary file 3 — jp4c02196_si_003.pdf [file jp4c02196_si_003.pdf]

Supporting information for:

Planting the Seeds of a Decision Tree for Ionic Liquids: Steric and Electronic Impacts on Melting Points: of Triarylphospounium Ionic Liquids

Marija Scheuren,[a,†] Lara Teodoro,[a,†] Andrew Witters,[a] Muhammadiqboli Musozoda,[b] Clinton Adu,[b] Gary Guillet,[c] Ronald Freeze,[a] Matthias Zeller,[d] Arsalan Mirjafari,*[,b] Patrick C. Hillesheim*[,a]

[a] Department of Chemistry and Physics, Ave Maria University, Ave Maria, Florida, 34142, United States

[b] Department of Chemistry, State University of New York at Oswego, Oswego, New York 13126, United States

[c] Department of Chemistry, Furman University, Greenville, South Carolina 29613, United States

[d] Department of Chemistry, Purdue University, West Lafayette, Indiana, 47907, United States

[†] These authors contributed equally to the manuscript.

S.1 Background and Rationalization

Herein we discuss aspects of the experiment and analysis which, while relevant to the overall discussion, appeared to be better suited for a separate area to allow for a more coherent story within the main text. Additionally, we provide images of the interactions mentioned within the main text though we highly recommend that readers so interested in examining the structures do so within the appropriate software to allow for a more clear perspective on how the molecules interact.

The chemistry of triphenylphosphine (TPP) is vast, spanning the fields of catalysis,[1] synthesis,[2] and pharmaceuticals.[3] As such, the literature revolving around the functionalization of this system is robust, with detailed synthetic procedures for the development of a vast library of TPP-based molecules. Additionally, there exist many commercially available TPP derivatives with diverse functional groups. Specifically, however, our intent is to probe the electronic and steric influence of modification of the π system of the aromatic moieties following fundamental principles. We chose to break apart our study into two parts: i) examining the steric influence *via* ortho, meta, para substitution of a methyl group; ii) electronic control by contrasting an electron withdrawing fluorine moiety vs. a methoxy donating group in the para position of the benzene rings.

Fundamental studies of ILs typically revolve around the synthesis and thermophysical characterization of the materials.[4] Indeed, a large wealth of information has been generated for the past three decades with regards to structure-property relationships of ILs.[5] With this gathered data, several broad trends have emerged as "understood" with regards to ILs. For example, using perfluorinated anions, such as bis(trifluoromethanesulfonyl)imide or NTf_2, leads to lower melting points when compared with halide ions.[6] Further, correlations between cation alkyl chain length and melting points are well established, allowing a measure of control over the liquefaction points of ILs.[7] There are, of course, exceptions to these trends, and thus there still exists a push for the development of new ILs.

One of these aforementioned trends is that phosphonium-based ILs tend to have higher thermal stability than ammonium-based compounds. Further, phosphonium ILs have, within the realm of ILs, lower than anticipated viscosities and conductivities. It is speculated that these properties are due to the association of the ion pairs in phosphonium salts being closer, by distance, than in the ammonium congeners due to the larger radius of the phosphorous atom.[8] It follows, then, that as the distance between ions change, interactions will change as well.

While electrostatic interactions between the ion pairs is the dominant non-covalent interaction (NCI), the distances and potential formation of additional NCIs between the component ions of an IL is of importance for understanding the properties of the material.[9,10] As shown in our previous works, ionic liquds (ILs) can readily crystallize, offering a wealth of information regarding the interactions found in the systems.[11,12] Further, while complicated due to the ionic charges present, the solid state and liquid state of ILs can be correlated, to an extent, offering insight into the behavior of this material *via* interpretation of the interactions that exist.[13–15] Specifically, however, is the fundamental idea that the melting transition is due to the weakening of the intermolecular interactions found within the solid state. Thus, studying the crystalline state of ILs allows for quantification and identification of these interactions.

S.2 Ring Rotations and the Impact on Melting Point

The crystal structure of the iodide salt of compound **TFP-NTf$_2$** (herein referred to as **TFP-I**) was acquired since the NTf$_2$ derivative is a liquid at room temperature. The asymmetric unit of **TFP-I** (figure S1) contains multiple cations in distinct rotational conformations. The crystal structure of **TFP-I** reveals key details to explain the lower melting point of the TFP compounds as well as offering insight into the structures and properties of the TPP compounds.

With respect to cation design for ILs, one of the governing principles leading to low melting compounds is that multiple, readily accessible conformations will depress the melting point.[16] This applies to all distinct moieties of the material (i.e., the alkyl chain, the heterocycle, the anion). Thus, one reason for the lower melting point of **TFP-NTf$_2$** is that the alkylated TFP cation has significantly more energetically accessible conformations when compared with the TPP cations. This is observed by the crystal structure of **TFP-I** displaying a variety of distinct orientations of the furyl rings.

To distinguish these conformations, we arbitrarily assigned 'up' to be when the furyl oxygen is pointed roughly in the direction of the alkyl chain (the P1—C1 bond). We began the naming with the furyl ring sitting between the hydrogens of the alkyl chain, and named the others in a counter-clockwise pattern. Further, in addition to the furyl rings showing multiple orientations, the alkyl chain also displays both anti and gauche conformations. Following these naming conventions, several distinct orientations of the cations exist in TFP-I: *down-down-down-gauche* (5-A); *down-down-up-gauche* (5-B); *down-up-down-anti* (5-C); *down-up-up-anti* (5-D); *up-up-down-gauche* (5-E); see Figure S2.

Several structural principles can be deduced from examining **TFP-I**. First, as mentioned previously, the cation displays many energetically accessible conformations in the solid state. It is logical to assume that these conformations are also accessible in the liquid state, i.e., when the iodide is exchanged for the NTf$_2$ anion. Thus, the many accessible conformations of TFP cations help in the formation of low-melting ILs. Second, the rotation of the furyl rings is less energetically costly than that of phenyl rings in TPP. To the point, for the TPP-based compounds herein, we observe no rotational disorder of phenyl rings. Further, alkylated TPP structures deposited in the CSD do not show rotational disorder. In contrast, examining the TFP structures in the CSD reveals several different orientations of the furyl rings within the structures to be reported (e.g. *down-up-up*, all down, etc.) indicative of ring rotations in multiple systems. As a point of reference, the crystal structure of the unalkylated trifurylphosphine shows an all-down conformation.[17] It should be stated however, that despite not showing complete rotations of the rings in the crystal structures, clearly the TPP phenyl rings do have some freedom of rotation as evidenced by the subtle differences in the ring torsion angles. However, the furyl rings appear far more prone to rotation simply based on reported structures.

The crystal structure of compounds **2Me-NTf$_2$** and **2Me-I** can offer further insight into the sterics of the rings (see figure S3). Specifically, the cations in both structures show distinct orientations of the methyl group implying that there is rotation of the rings. Following the naming convention discussed for TFP-I, the cation in **2Me-NTf$_2$** shows an *up-up-down-anti* conformation and in **2Me-I** a conformation of *up-up-down-gauche* is observed, with up and down being in reference to the methyl groups.

Examining the deposited structures in the CSD helps shed light onto these molecular scaffolds. As a point of reference, the unalkylated compound, that is tris(2-methylphenyl)phosphine, shows an all-up conformation of the compound.[18] Notably, less sterically demanding cationic forms of the compound show an all-up conformation as well.[19] Unsurprisingly, the bulkier the groups bound to the central phosphorous, the more likely that the *up-up-down* conformation of the rings is observed in the structures. Curiously, despite the presence of bulkier substituents on the phosphorous atom, some crystals still show the all-up conformation.[20] Thus, cursory observation of these compounds leads to the hypothesis that the rings can rotate for the *o*-methyl derivative. However, there appears to be a threshold of steric encumbrance that forces the inversion of a ring. A systematic study involving stepwise alkylation of the phosphorous atom could help to clarify this.

In the 2Me cations there is the 'down' facing ring wherein the methyl group is oriented toward the positively charged phosphonium 'pocket' formed by the three aromatic rings, on the opposite side of the alkyl chain. This methyl group in this orientation hinders the formation of certain cation-cation interactions which are observed in 3Me and 4Me. In 2Me, however, only cation-anion interactions are observed in the region near this pocket. Thus, the increased cation-anion interactions in 2Me raise the melting point of the compound while the potential to form cation-cation interactions in 3Me and 4Me depresses the melting points, to a degree, following on the principles discussed in related literature.[21] A representation of these 'pocket interactions' is shown in figure S4 which contrasts 2Me vs 3Me.

To summarize the discussion on ring sterics, several key ideas can be drawn from the crystal structures.

- First, the TPP-based ILs display common plane angles with respect to the cation structure, leading to broad similarity in the cation geometry. The ring torsion angles, and thus the geometry of the cation, are influenced by intramolecular interactions with the alkyl chain.
- Second, the smaller five-membered furyl rings have increased rotation of the rings as evidenced by multiple conformations observed in the crystal structure (**TFP-I**). These readily accessible conformations help depress the melting point of these TFP-based compounds.
- Finally, there is evidence that alkylated TPP structures can rotate as shown by the orientation of the methyl groups in **2Me-NTf$_2$** and **2Me-I**. However, some reported structures do not show any "down" orientation despite having moieties attached to the central phosphorous atom. Thus, a more thorough investigation in the future is warranted to clarify the rotational barriers for these compounds.

S.3 Alkyl Chain Conformations

To gain a more thorough understanding of the molecular and crystal structures of alkylated TPP compounds, and the underlying interactions, we elected to examine the halide salts. The asymmetric units of these compounds are shown in figure S5. Several unique features are noted in the halide salts which both supplement previous discussions while also prompting new questions. To keep naming as simple as possible, we simply refer to the halide salts as **2Me-I**, **3Me-I**, **4Me-I**, etc.

As with the NTf_2 compounds, the halide salts are similar with respect to cation structure. Again, we observe a similar set of plane angles for the rings within the cations. However, further details emerge which shed insight into the cation structure. For example, in contrast to the NTf_2 compounds wherein the predominant conformation of the alkyl chain is the anti-conformation, the halide anions seem to affect a change in crystal packing wherein the gauche conformation is more frequently observed. Of the eight reported halide structures, three have a gauche conformation of the chain. For the NTf_2^- structures only two structures display a gauche conformation as part of a disorder in the crystal.

With respect to the cause for this change, the size of the anions (i.e., I^- vs. NTf_2^-) is one obvious difference in the structures. Phosphonium ILs behave in a unique manner with regards to cation-anion pairing, as opposed to the more common heterocyclic imidazolium ILs.[8] Specifically, due to the larger radius of the phosphorous atom, anions more closely associate to the positive atom in the cation than in charge disperse cations (e.g. imidazolium cation). Thus, certain phosphonium-based ILs show unusual trends in conductivity, as an example, and can be thought to behave somewhat as a hybrid of molecular and ionic liquids.[22] In brief, the smaller volume iodide anions allow for closer interactions with the phosphonium atom facilitating different packing in the crystal state wherein the gauche conformation is more viable due to packing efficiency[23] or due to different interactions arising from the alkyl chain conformations as observed in our previous work.[24]

With respect to conformational changes in the alkyl chain, compound **4Me-I** has a unique cation geometry when compared with all other reported structures. The alkyl chain in **4Me-I** has a P1—C1—C2—C3 torsion angle of 98.17(13)° while in other cations the similar torsion angle is approximately 180°. Thus **4Me-I** has a pseudo-eclipsed conformation with respect to the C1—C2 bond. Therefore, in addition to the anticipated anti/gauche conformations, the structure of **4Me-I** shows that additional alkyl chain arrangements are possible.

S.4 Halide Conformational Polymorphs

Crystals of both the gauche and anti-conformations of **4MeO-I** were obtained. Compound **4MeO-I** crystallizes in both a triclinic ($P\bar{1}$) and monoclinic ($P2_1/n$) crystal system. Certainly, solvents can influence the formation of polymorphs, as the triclinic system crystallizes from evaporation of tetrahydrofuran while the monoclinic system forms from the melt upon cooling. This result is relatively unsurprising given how similar the conformers are in energy. As is often quoted: "*...the number of forms known for that compound is proportional to the time and money spent in research on that compound.*"[25] Thus, as a layer of complexity, the potential formation of polymorphs should be considered as part of the discussion of these compounds. A more rigorous thermal and/or crystallographic screening of all the samples could help shed light onto the formation of polymorphs and the influence of solvent.[13] Of note to the interested reader, polymorphism has been shown to be an important feature of ILs, particularly when related to the alkyl chain conformations.[26]

S.5 Impacts of the Halide Salt

The bromide salt of the methoxy derivative (i.e., **4MeO-Br**) was synthesized and crystallized as well. This compound was synthesized to address specific issues not directly related to our investigations into the structure of the cations, but rather to address solubility problems with the iodide salt. However, the solubility of the bromide salt vs. the iodide salt was not significantly different and thus did not present any useful qualities with respect to production of the ILs. However, the crystal structure shows no notable changes when contrasted with the iodide. Synthesis of the chloride salt proved unsuccessful with our current equipment or methods. Despite these issues, however, the bromide derivative does show similar structural features as the iodide.

In summary of the observations from the halide crystal structures, there are distinct, key points drawn from visual inspection of the molecular structures relevant to the overall analysis of these compounds. First, the butylated-TPP based compounds all exhibit comparable structures with respect to their ring torsion angles. This appears true regardless of the anion used. Second, we observe a slight increased preference for the gauche conformation of the alkyl chains with three of the eight halide structures showing this arrangement. For the NTf_2 compounds, however, the anti-conformation is preferred, when solely examining the crystal structures collected. Finally, we have observed polymorphic forms of the TPP compounds. A more thorough understanding of the influence of solvents and interactions on the formation of these polymorphs is part of our on-going studies.

S.6 Interactions of 4CF3-NTf$_2$

In an attempt to examine both the steric and electronic effects of functionalization, compound **4CF$_3$-NTf$_2$** was synthesized and crystallized. Given the addition of multiple fluorine atoms, the overall percentage of F···C|C···F interactions is quite high (see figure S6). As opposed to **4F-NTf$_2$**, **4CF$_3$-NTf$_2$** does exhibit a number of inter-cationic $F_{CF_3}\cdots\pi$ interactions arising from the added fluorines. With respect to these cation-cation interactions, both side-on and face-on $F_{CF_3}\cdots\pi$ interactions are observed with several different carbon atoms around the aromatic rings. The shortest of these interactions arises between the aromatic carbons C9A and a symmetry adjacent fluorine F3C at a distance of 3.029 (7) Å (d(C···F^k), k = 1+x, +y, +z). Additional inter-cationic $F_{CF_3}\cdots\pi$ interactions arise with the other rings ranging in distances from 3.35 to 3.45 Å (d(C···F)). Thus, while $4CF_3$-NTF_2 shows comparable F···π interaction distances as with 4F-NTf_2, the trifluoromethyl group introduces a greater number of inter-cationic interactions not observed 4F-NTf_2.

As expected, interactions are affected due to the more sterically demanding nature of the CF_3 moiety. As discussed within the main narrative with respect to **4F-NTf$_2$** and **4MeO-NTf$_2$**, interactions were noted with the carbon atoms in the para positions (e.g., C8). While these interactions with C8 are also present in **4CF$_3$-NTf$_2$**, they are only formed from the anion. Curiously, not every C8 position in the cation is seen to form interactions, however. Further, while no π-stacking is observed between cations, there is a long $F_{CF_3}\cdots P$ interaction at 4.346 Å (d(F···P)). Thus, while introduction of a sterically demanding CF_3 moiety led to the formation of significant inter-cationic interactions, the nature of these interactions is quite distinct from **4F-NTf$_2$** and **4MeO-NTf$_2$**. The steric bulk of the CF_3 necessitates a change in the interactions with the aromatic carbons, likely preventing the formation of π stacking and favoring $F_{CF_3}\cdots\pi$ interactions instead.

S.7 On the Shape and Geometry of the Functional Groups

While **4MeO-NTf$_2$** has the lowest T_m of the TPP-based compounds, **4CF$_3$-NTf$_2$** has a higher dipole. However, direct comparisons of these two compounds solely based on dipole moments could be misleading. Rather, coupling the idea of the impacts of dipole moments with observations drawn from the crystal structures allows for a more complete understanding of the compounds. For example, both compounds show free rotation of functional groups in the crystal structure as evidenced by the presence of crystallographic rotational disorder. The 4MeO cation shows two distinct orientations of the methoxy methyl group, as seen in the iodide crystal (**4MeO-I**), while **4CF$_3$-NTf$_2$** shows rotational disorder in the CF_3. Thus, while both compounds introduce new cation-cation interactions *via* the functional groups which could lead to lower melting points, distinctions arise from

which portion of the functional group is interacting and how rotational freedom can influence the formation of these interactions.

Rotations of the methoxy group would lead to a change in the interactions arising from the different moieties (i.e., CH_3 and O), thus affecting the cation-cation and cation-anion interactions, resulting in lower melting points through rotationally induced frustration hindering (or slowing) the formation of these stabilizing interactions. Simply, the methoxy oxygen moiety is observed to make interactions with adjacent π systems, a stabilizing interaction. A rotation of the methoxy methyl group would result in a significant movement of any adjacent interacting molecules, thus disrupting these $O\cdots\pi$ interactions. For the CF_3 moiety, however, rotation would likely not cause as significant a disruption of the interactions since the interactions can only happen from the peripheral F_{CF_3} atoms. As a final thought, this rotational disorder may account for the observed tendency of the **4MeO-NTf$_2$** to exist as a supercooled liquid for extended times when left undisturbed.

(1) Mitsunobu, O. The Use of Diethyl Azodicarboxylate and Triphenylphosphine in Synthesis and Transformation of Natural Products. *Synthesis* **2002**, *1981* (01), 1–28. https://doi.org/10.1055/s-1981-29317.
(2) Moussa, Z.; Judeh, Z. M. A.; Ahmed, S. A. Polymer-Supported Triphenylphosphine: Application in Organic Synthesis and Organometallic Reactions. *RSC Adv.* **2019**, *9* (60), 35217–35272. https://doi.org/10.1039/C9RA07094J.
(3) Zielonka, J.; Joseph, J.; Sikora, A.; Hardy, M.; Ouari, O.; Vasquez-Vivar, J.; Cheng, G.; Lopez, M.; Kalyanaraman, B. Mitochondria-Targeted Triphenylphosphonium-Based Compounds: Syntheses, Mechanisms of Action, and Therapeutic and Diagnostic Applications. *Chem. Rev.* **2017**, *117* (15), 10043–10120. https://doi.org/10.1021/acs.chemrev.7b00042.
(4) Deetlefs, M.; Fanselow, M.; Seddon, K. R. Ionic Liquids: The View from Mount Improbable. *RSC Adv.* **2016**, *6* (6), 4280–4288. https://doi.org/10.1039/C5RA05829E.
(5) *Ionic Liquids: Physicochemical Properties*, 1st ed.; Zhang, S., Ed.; Elsevier: Amsterdam, The Netherlands ; Boston ; London, 2009.
(6) MacFarlane, D. R.; Kar, M.; Pringle, J. M. *Fundamentals of Ionic Liquids*; Wiley-VCH Verlag GmbH & Co. KGaA: Weinheim, Germany, 2017. https://doi.org/10.1002/9783527340033.
(7) Murray, S. M.; O'Brien, R. A.; Mattson, K. M.; Ceccarelli, C.; Sykora, R. E.; West, K. N.; Davis Jr., J. H. The Fluid-Mosaic Model, Homeoviscous Adaptation, and Ionic Liquids: Dramatic Lowering of the Melting Point by Side-Chain Unsaturation. *Angew. Chem. Int. Ed.* **2010**, *49* (15), 2755–2758. https://doi.org/10.1002/anie.200906169.
(8) Fraser, K. J.; MacFarlane, D. R. Phosphonium-Based Ionic Liquids: An Overview. *Aust. J. Chem.* **2009**, *62* (4), 309. https://doi.org/10.1071/CH08558.
(9) Cosby, T.; Vicars, Z.; Heres, M.; Tsunashima, K.; Sangoro, J. Dynamic and Structural Evidence of Mesoscopic Aggregation in Phosphonium Ionic Liquids. *J. Chem. Phys.* **2018**, *148* (19), 193815. https://doi.org/10.1063/1.5009765.
(10) Wang, Y.-L.; Li, B.; Sarman, S.; Mocci, F.; Lu, Z.-Y.; Yuan, J.; Laaksonen, A.; Fayer, M. D. Microstructural and Dynamical Heterogeneities in Ionic Liquids. *Chem. Rev.* **2020**, *120* (13), 5798–5877. https://doi.org/10.1021/acs.chemrev.9b00693.
(11) Bellia, S. A.; Metzler, M.; Huynh, M.; Zeller, M.; Mirjafari, A.; Cohn, P.; Hillesheim, P. C. Bridging the Crystal and Solution Structure of a Series of Lipid-Inspired Ionic Liquids. *Soft Matter* **2023**, *19* (4), 749–765. https://doi.org/10.1039/D2SM01478E.
(12) Anderson, G. I.; Hardy, D.; Hillesheim, P. C.; Wagle, D. V.; Zeller, M.; Baker, G. A.; Mirjafari, A. Anticancer Agents as Design Archetypes: Insights into the Structure–Property Relationships of Ionic Liquids with a Triarylmethyl Moiety. *ACS Phys. Chem. Au* **2023**, *3* (1), 94–106. https://doi.org/10.1021/acsphyschemau.2c00048.
(13) Ymén, I. Introduction to the Solid State – Physical Properties and Processes. In *Solid State Characterization of Pharmaceuticals*; John Wiley & Sons, Ltd, 2011; pp 1–34. https://doi.org/10.1002/9780470656792.ch1.
(14) Dupont, J. On the Solid, Liquid and Solution Structural Organization of Imidazolium Ionic Liquids. *J. Braz. Chem. Soc.* **2004**, *15* (3), 341–350. https://doi.org/10.1590/S0103-50532004000300002.
(15) Winterton, N. Crystallography of Ionic Liquids. In *Ionic Liquids Completely UnCOILed*; John Wiley & Sons, Ltd, 2015; pp 231–534. https://doi.org/10.1002/9781118840061.ch11.
(16) Endo, T.; Kato, T.; Tozaki, K.; Nishikawa, K. Phase Behaviors of Room Temperature Ionic Liquid Linked with Cation Conformational Changes: 1-Butyl-3-Methylimidazolium Hexafluorophosphate. *J. Phys. Chem. B* **2010**, *114* (1), 407–411. https://doi.org/10.1021/jp909256j.
(17) Monkowius, U. V.; Nogai, S.; Schmidbaur, H. Unsuccessful/Successful Attempts to Produce Penta(Heteroaryl)-Phosphoranes/-Arsoranes R5E (E = P, As; R = 2-Furyl, 2-Thienyl). *Dalton Trans* **2004**, No. 10, 1610–1617. https://doi.org/10.1039/B401988A.
(18) Cameron, T. S.; Dahlèn, B. Investigation of Phosphorus–Carbon Bond Lengths in Aromatic Phosphines. Part I. Crystal and Molecular Structures of Tri-o-Tolylphosphine, -Phosphine Oxide, -Phosphine Sulphide,

and -Phosphine Selenide. *J Chem Soc Perkin Trans 2* **1975**, No. 15, 1737–1751. https://doi.org/10.1039/P29750001737.
(19) Zhou, J.; Cao, L. L.; Liu, L. L.; Stephan, D. W. FLP Reactivity of [Ph3C]+ and (o-Tolyl)3P and the Capture of a Staudinger Reaction Intermediate. *Dalton Trans* **2017**, *46* (29), 9334–9338. https://doi.org/10.1039/C7DT01726J.
(20) Santini, C.; Pettinari, C.; Lobbia, G. G.; Leonesi, D.; Valle, G.; Calogero, S. Silver(I) and Gold(I) Complexes of Hydrotris(3,5-Dimethylpyrazol-1-Yl)Borate: Synthesis, Spectroscopic and Structural Characterization, and Reactivity toward C-, N- and S-Donor Ligands. *Polyhedron* **1998**, *17* (18), 3201–3210. https://doi.org/10.1016/S0277-5387(98)00092-8.
(21) Rabideau, B. D.; Soltani, M.; Parker, R. A.; Siu, B.; Salter, E. A.; Wierzbicki, A.; West, K. N.; Davis, J. H. Tuning the Melting Point of Selected Ionic Liquids through Adjustment of the Cation's Dipole Moment. *Phys. Chem. Chem. Phys.* **2020**, *22* (21), 12301–12311. https://doi.org/10.1039/D0CP01214A.
(22) Fraser, K. J.; Izgorodina, E. I.; Forsyth, M.; Scott, J. L.; MacFarlane, D. R. Liquids Intermediate between "Molecular" and "Ionic" Liquids: Liquid Ion Pairs? *Chem Commun* **2007**, No. 37, 3817–3819. https://doi.org/10.1039/B710014K.
(23) Hayes, R.; Warr, G. G.; Atkin, R. Structure and Nanostructure in Ionic Liquids. *Chem. Rev.* **2015**, *115* (13), 6357–6426. https://doi.org/10.1021/cr500411q.
(24) O'Rourke, B.; Lauderback, C.; Teodoro, L. I.; Grimm, M.; Zeller, M.; Mirjafari, A.; Guillet, G. L.; Hillesheim, P. C. Developing Structural First Principles for Alkylated Triphenylphosphonium-Based Ionic Liquids. *ACS Omega* **2021**, *6* (47), 32285–32296. https://doi.org/10.1021/acsomega.1c05241.
(25) Lee, E. H. A Practical Guide to Pharmaceutical Polymorph Screening & Selection. *Asian J. Pharm. Sci.* **2014**, *9* (4), 163–175. https://doi.org/10.1016/j.ajps.2014.05.002.
(26) Saouane, S.; Norman, S. E.; Hardacre, C.; Fabbiani, F. P. A. Pinning down the Solid-State Polymorphism of the Ionic Liquid [Bmim][PF6]. *Chem. Sci.* **2013**, *4* (3), 1270. https://doi.org/10.1039/c2sc21959j.

Supplemental Images for Discussion

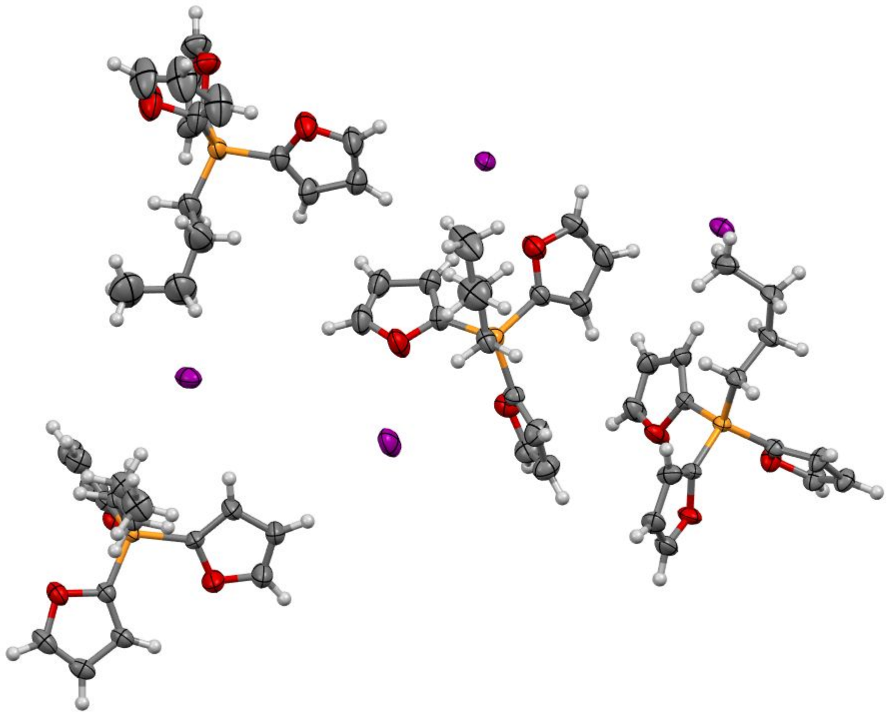

Figure S1. Asymmetric unit of **TFP-I** shown with 50% probability ellipsoids. Disorder omitted for clarity.

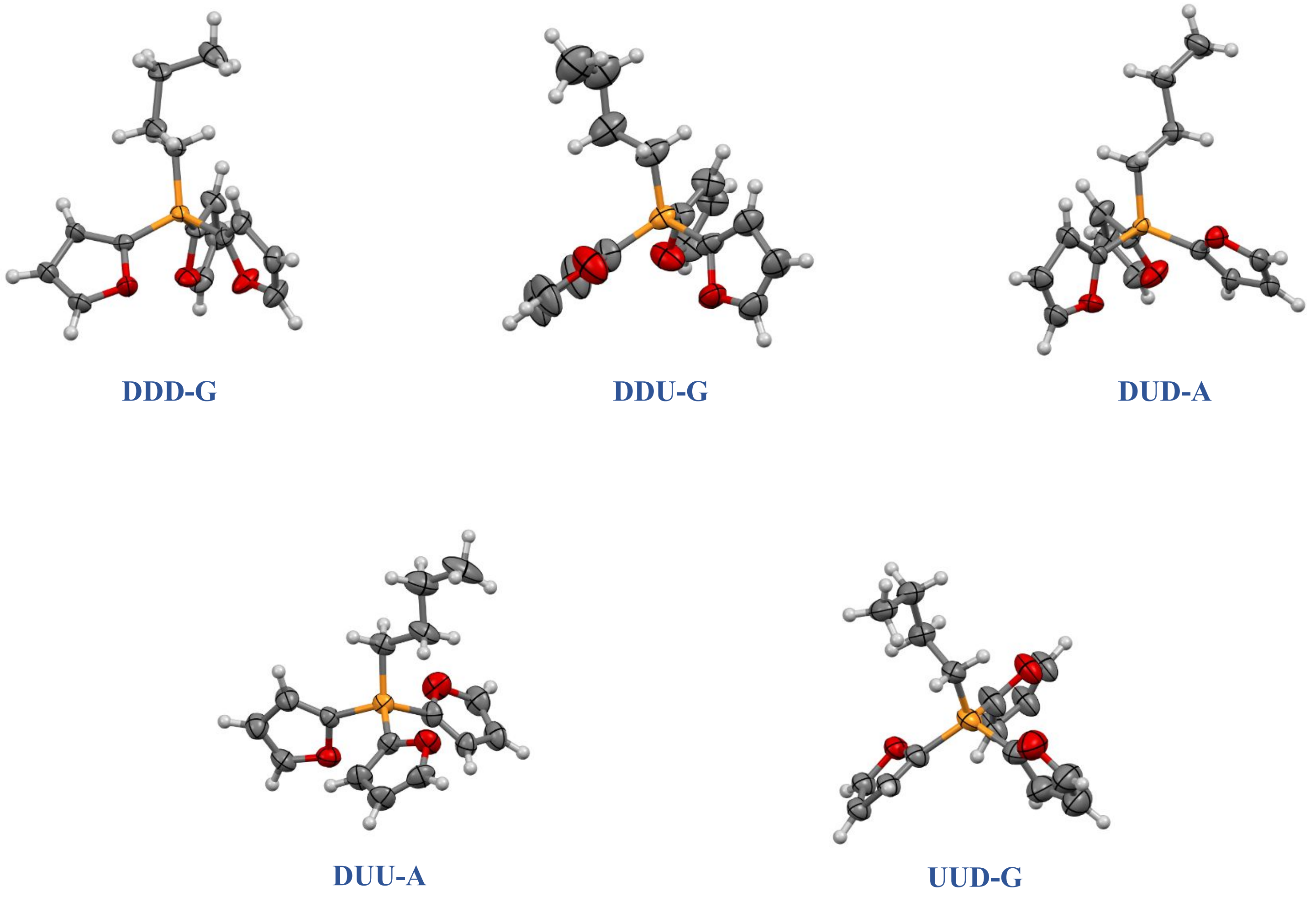

Figure S2. The different conformations of the cation from **TFP-I**. Up (U) is arbitrarily when the furyl oxygen is oriented in approximately the same direction as the P1—C1 bond of the alkyl moiety. Down (D) is facing in the opposite direction; A – Anti conformation of the alkyl chain; G – Gauche conformation of the alkyl chain. Note that within the crystal there is also a conformation wherein the orientation is down-down-up-anti, yet this would be equivalent to the down-up-down-anti from a purely geometric perspective (moving clockwise vs counter-clockwise).

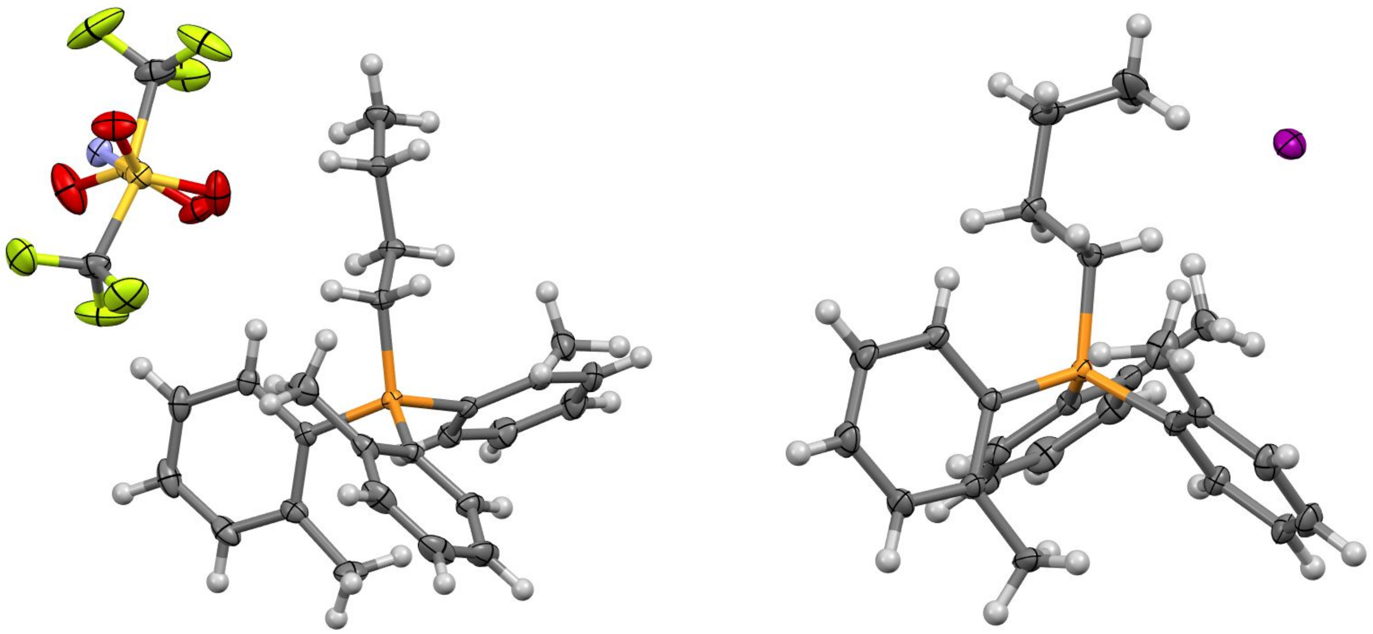

Figure S3. The asymmetric units of 2Me-NTf2 (left) and 2Me-I (right).

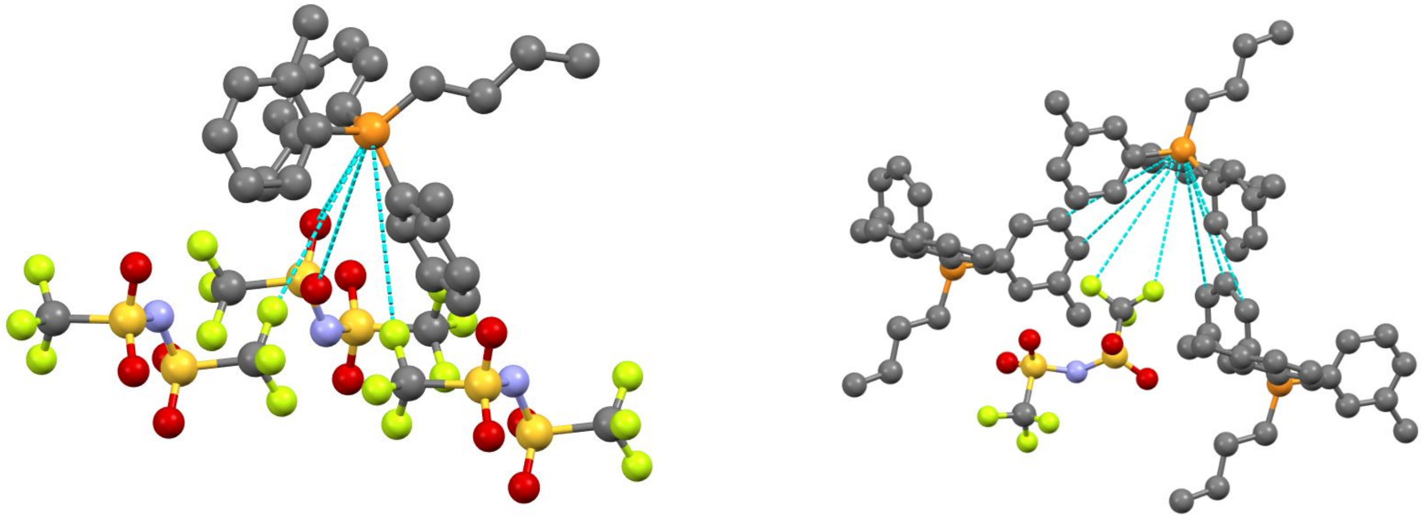

Figure S4. Depiction of interactions near the lower phosphonium pocket of **2Me-NTf$_2$** (left) and **3Me-NTf$_2$** (right). Blue lines are shown merely to help guide the eye rather than imply interactions with all the atoms. More cation-cation interactions are observed in **3Me-NTf$_2$** than in **2Me-NTf$_2$** arising in this “pocket.”

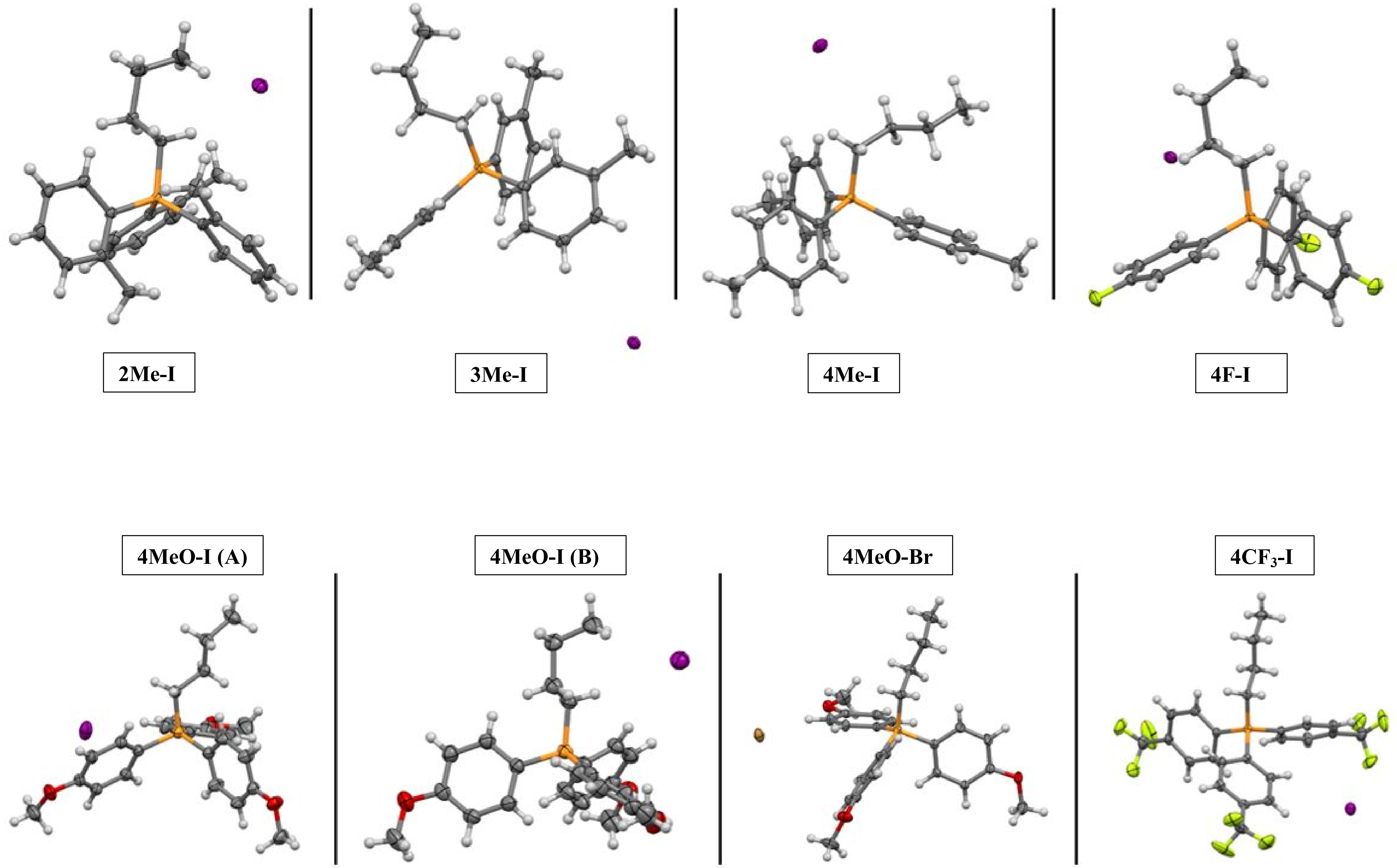

Figure S5. Asymmetric units of the halide salts for the TPP-based cations studied herein. Images shown with 50% probability ellipsoids. Disorder omitted for clarity.

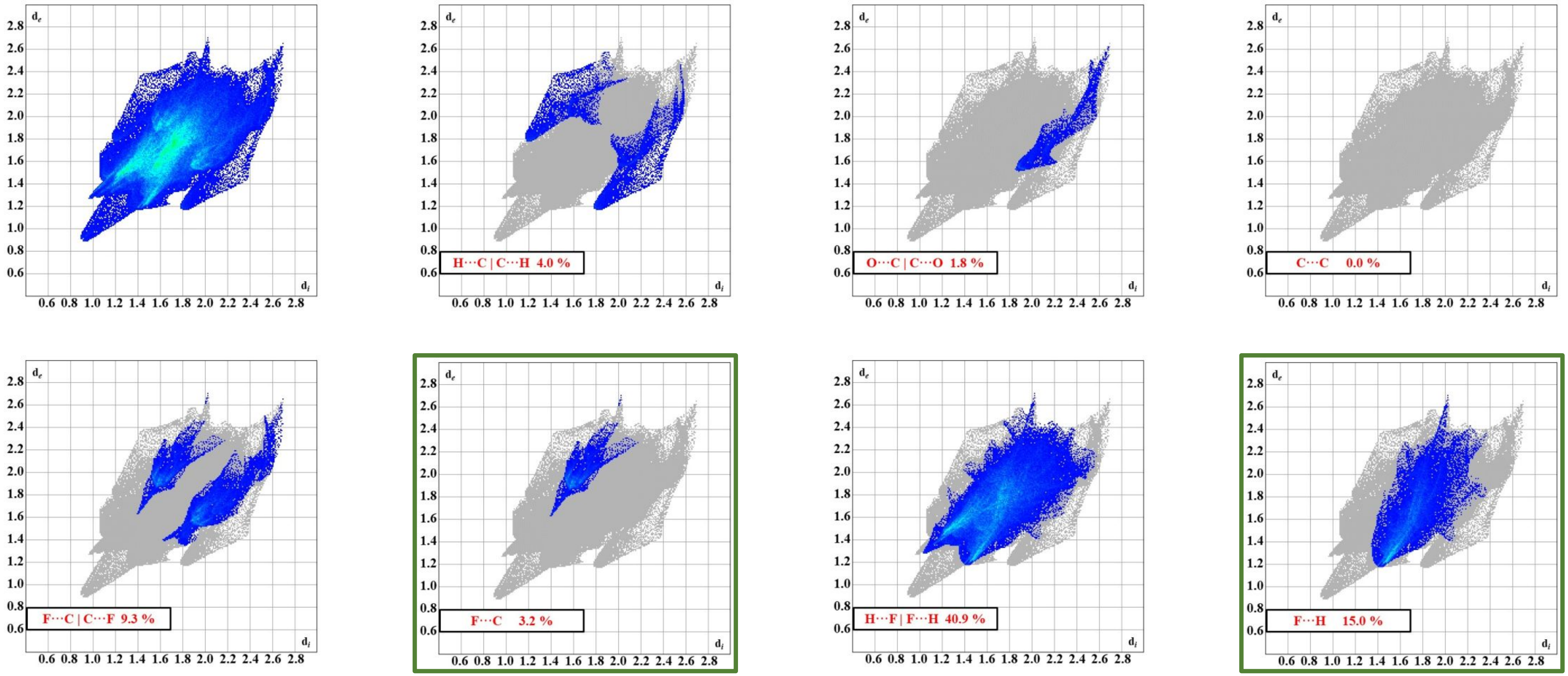

Figure S6. Interaction fingerprint for **4CF$_3$-NTf$_2$**. Highlighted fingerprints show interactions arising from the fluorine moieties on the cation exclusively.

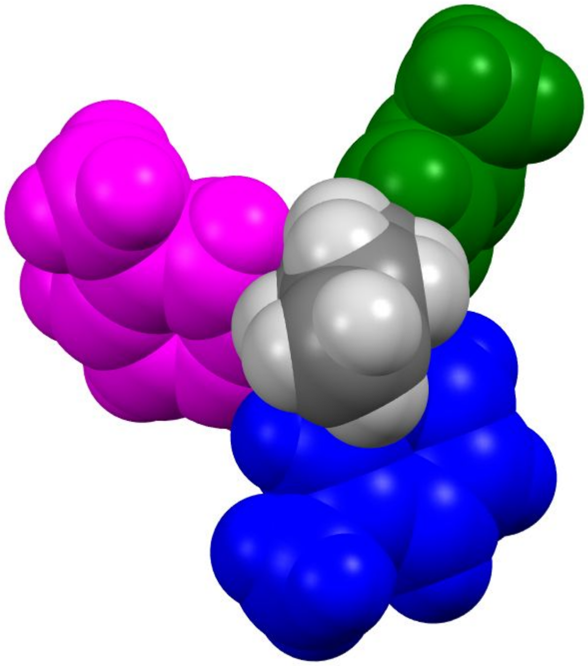

Figure S7. Space-filling model of the cation from **3Me-NTf$_2$** shown as an example of the naming and intramolecular sterics. Ring A (blue) resides beneath the butyl chain (gray/white). Ring B (green) resides in the 'spine' of the butyl chain. Ring C (magenta) site between the methylene hydrogens of C1 and C2.

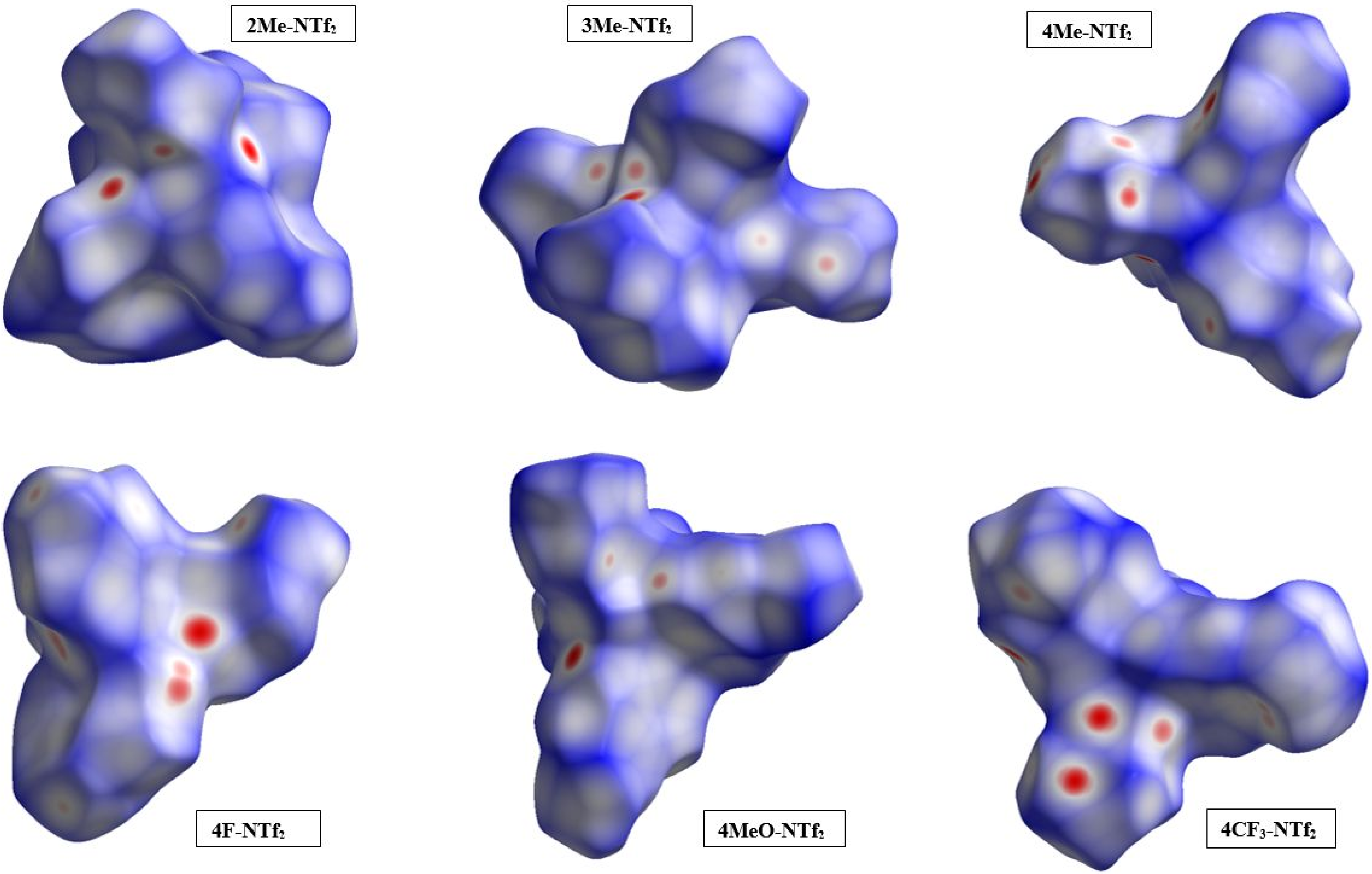

Figure S8. The Hirshfeld surfaces of the compounds mapped with the d_{norm} function.

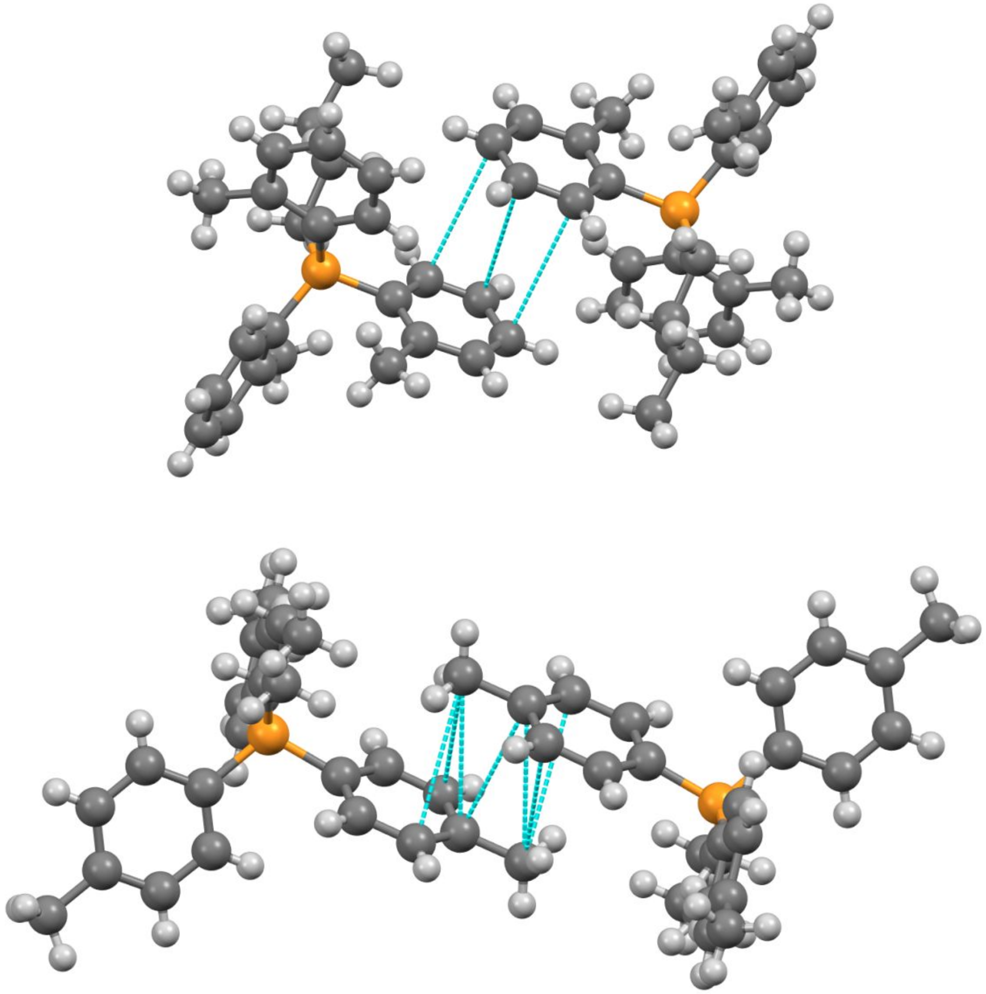

Figure S9. Stacking interactions in **$2Me\text{-}NTf_2$** (top) and **$4Me\text{-}NTf_2$** (bottom). Compound **$3Me\text{-}NTf_2$** shows no π-π stacking. Interactions shown to the sum of the van der Waal radii + 0.3 Å.

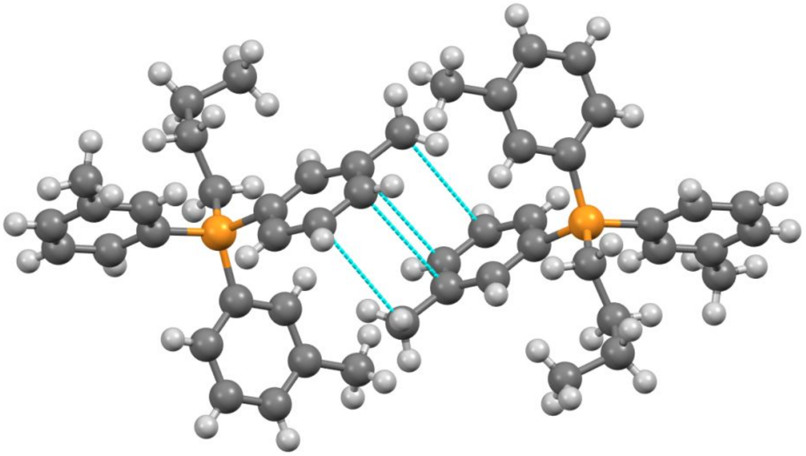

Figure S10. Stacking interactions in **3Me-I**. Interactions shown to the sum of the van der Waal radii + 0.3 Å

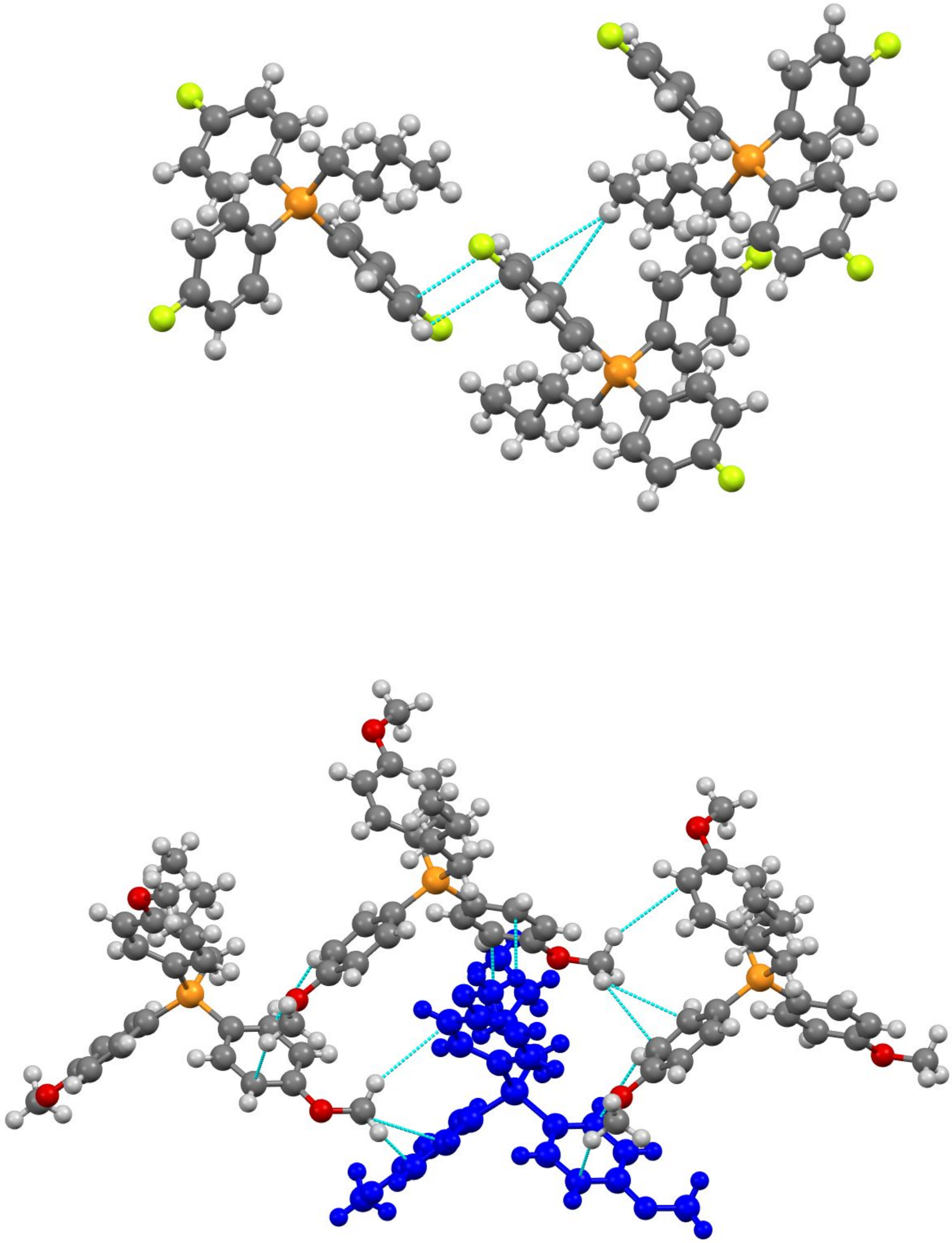

Figure S11. Depiction of the H···C|C···H interactions in **4F-NTf$_2$** (top) and **4MeO-NTf$_2$** (bottom). Interactions shown to the sum of the van der Waal radii + 0.3 Å. Molecule in blue is shown to provide clarity.

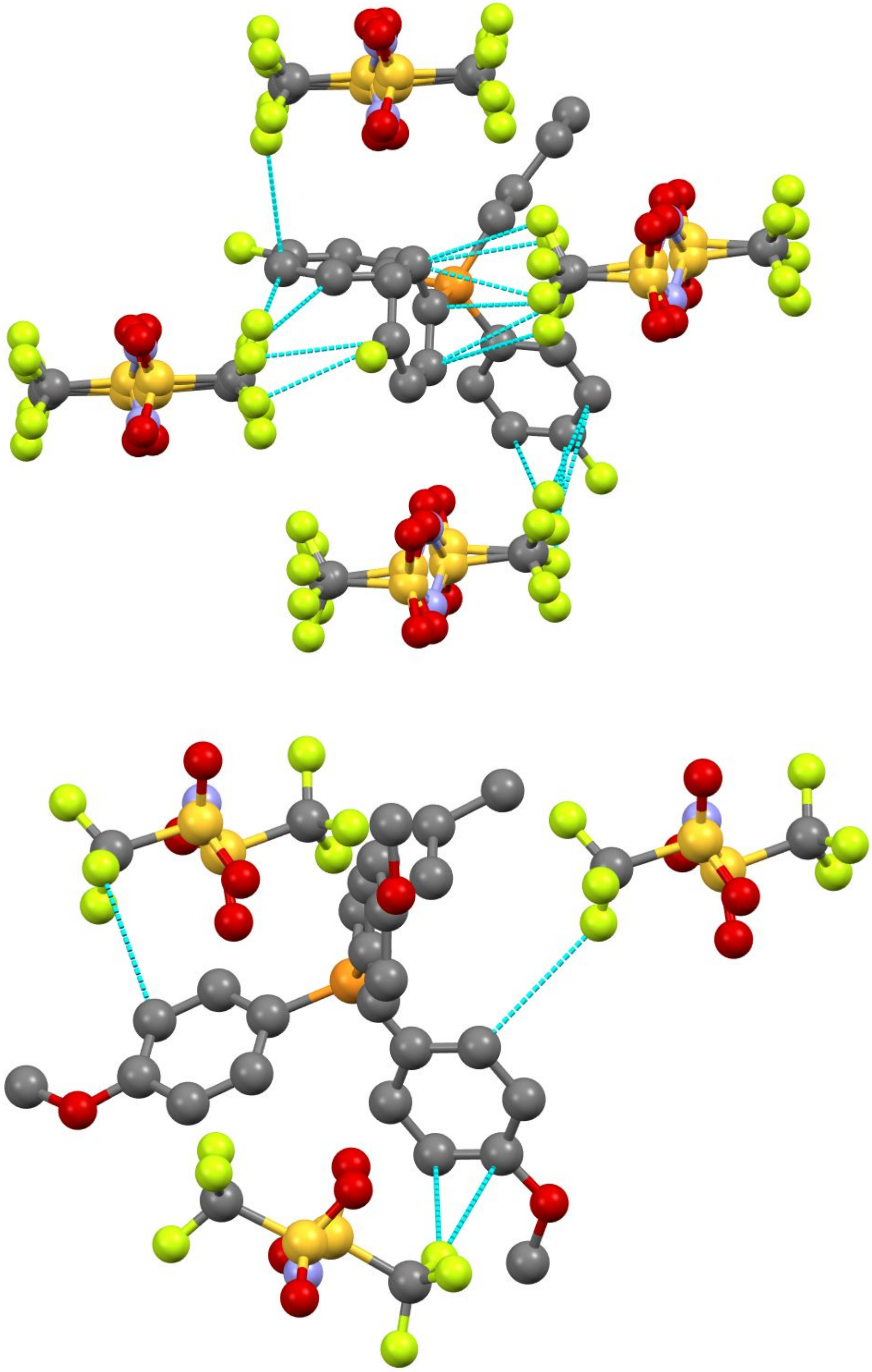

Figure S12. Depiction of the F···C|C···F interactions in **4F-NTf$_2$** (top) and **4MeO-NTf$_2$** (bottom). Interactions shown to the sum of the van der Waal radii + 0.3 Å. Hydrogens omitted for clarity. Anion disorder is shown in **4F-NTf$_2$**.

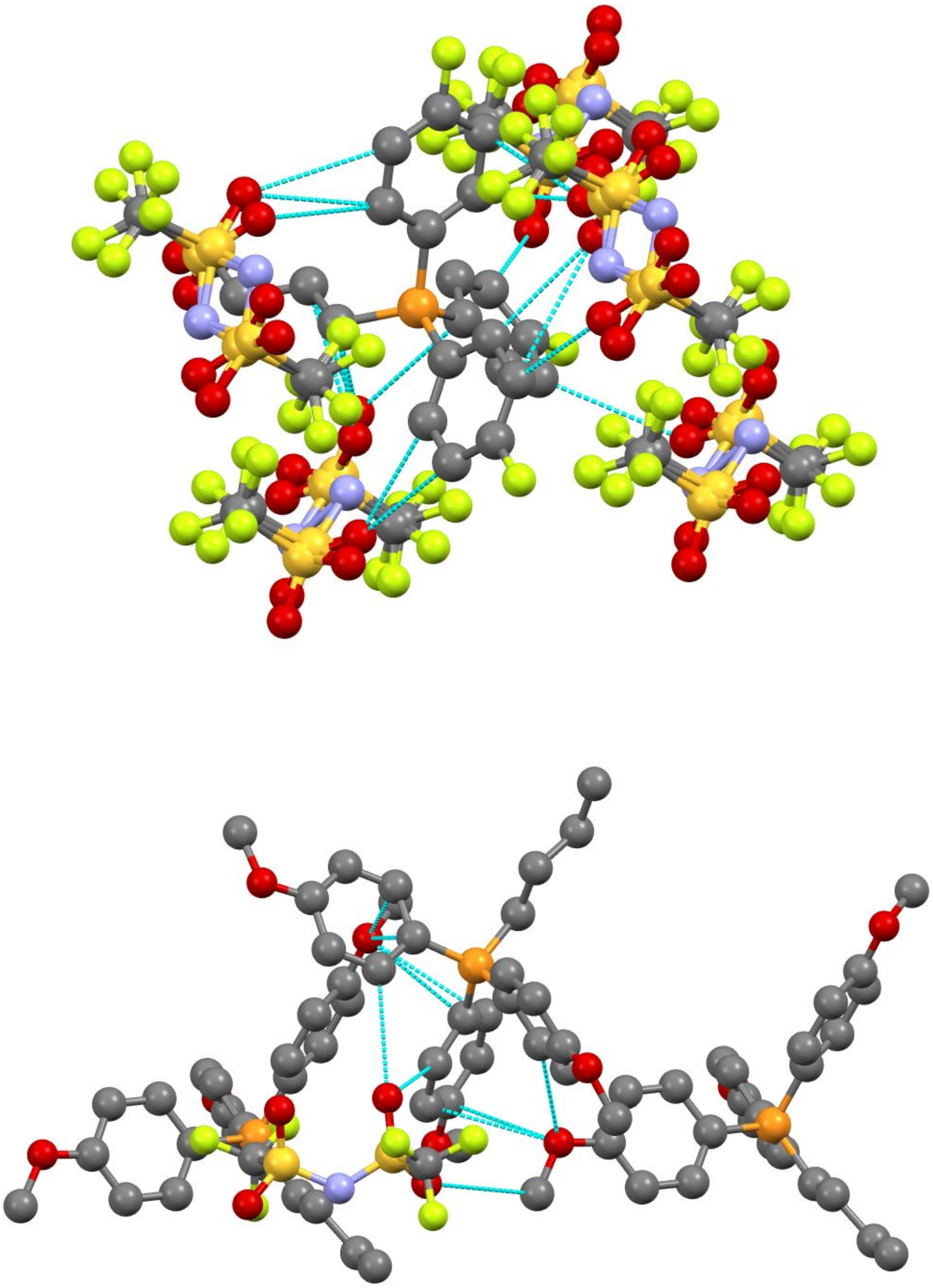

Figure S13. Depiction of the O···C|C···O interactions in **4F-NTf$_2$** (top) and **4MeO-NTf$_2$** (bottom). Interactions shown to the sum of the van der Waal radii + 0.3 Å. Hydrogens omitted for clarity. Anion disorder is observed in **4F-NTf$_2$**

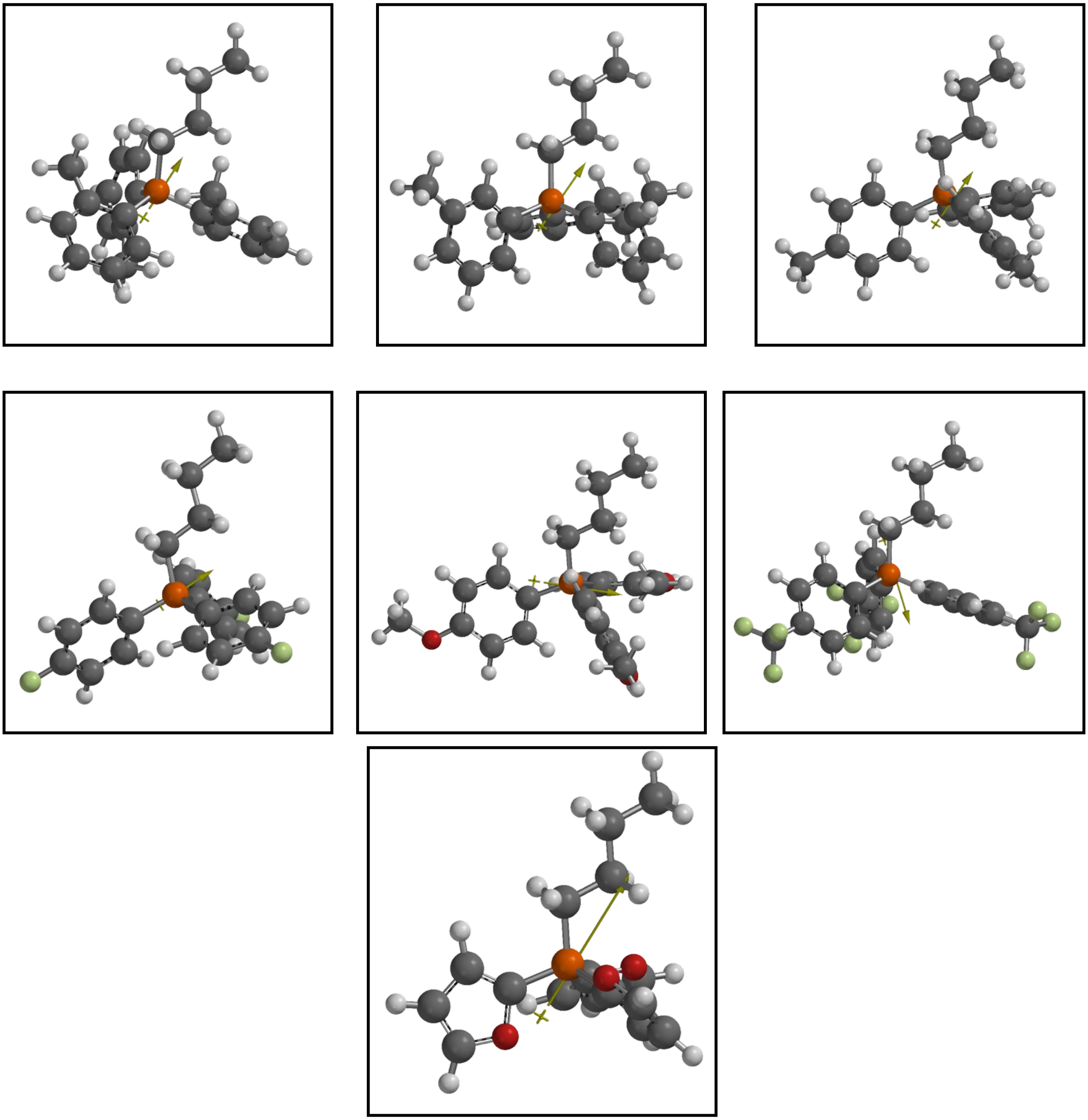

Figure S14. Optimized cations from the crystals shown with the calculated dipole vector (yellow arrow).

Two procedures were used for the synthesis of the halide salts of the compounds.

SP1

Procedure 1 is a modification of a previously published synthesis.[26] 1-iodobutane (3 eq), the appropriate triarylphophine (1 eq), 1 mL of toluene, and a stir bar were added to a container and sealed. The container was heated for up to 48 hours or until solid had stopped forming. The reaction was cooled and then the solid filtered. The solid was washed with ethyl acetate (3 x 10 mL) and methyl tert-butyl ether (3 x 10 mL). The resulting halide salt was used as is without further purification.

SP2

Procedure 2 is a modification of SP1. 1-iodobutane (3 eq), the appropriate triarylphophine (1 eq), and a stir bar were added to a pressure vial and sealed. The mixture was heated to 175 °C overnight. The reaction was cooled and the resultant solid triturated with ethyl acetate (3 x 15 mL). The resulting halide salt was used as is without further purification.

Metathesis Procedure

The metathesis procedure described was used for all the compounds herein. The appropriate halide salt (1 eq) was dissolved in a minimal amount of water while stirring. In some cases, the water was heated to dissolve the compound due to low solubility of the halide salt. Lithium bis(trifluoromethanesulfonyl)imide (1.2 eq) was added to the solution and a white solid immediately precipitated. The mixture was stirred for two hours and then filtered. The solid was dissolved in ethyl acetate and washed with water until a negative halide test was observed. The solvent was removed under vacuum and the resultant product dried under vacuum at 55 °C overnight.

Butyl tris(2-methylphenyl)phosphonium bis(trifluoromethanesulfonyl)imide (2Me-NTf$_2$)

Synthesized following SP2. Yield (78 %)

Single crystals of the iodide salt were grown from slow diffusion of diethyl ether in a saturated solution of the compound in acetone. Single crystals of the NTf$_2$ salt were grown from slow diffusion of diethyl ether into a saturated solution of the compound in dichloromethane.

^{1}H NMR (400 MHz; DMSO-d$_6$) δ 7.81 (s, 3H), 7.57 (t, J = 14.3 Hz, 9H), 3.55 (s, 2H), 2.18 (s, 9H), 1.56 (s, 2H), 1.28 (s, 2H), 0.85 (s, 3H).
^{13}C NMR (101 MHz; DMSO-d$_6$) δ 143.21, 135.10, 134.58, 133.60, 127.73, 124.30—114.69, 116.29, 26.51, 22.92, 22.03, 22.15—21.67, 13.27.
^{19}F NMR (376 MHz; DMSO-d$_6$) δ -78.64 (s, 6F).
^{31}P NMR (162 MHz; DMSO-d$_6$) δ 27.29 (s, 1P).
(ESI) m/z: [M]$^+$ Calcd $C_{25}H_{30}P$ for 362.49; Found 361.2

Butyl tris(3-methylphenyl)phosphonium bis(trifluoromethanesulfonyl)imide (3Me-NTf$_2$)

Synthesized following SP2. Yield (72 %)

Single crystals of the iodide salt were grown from slow diffusion of methyl tertbutyl ether in a saturated solution of the compound in acetone. Single crystals of the NTf_2 salt were grown from slow diffusion of diethyl ether into a saturated solution of the compound in chloroform.

^{1}H NMR (400 MHz; DMSO-d_6) δ 7.64 (td, J = 23.6, 8.3 Hz, 12H), 3.49 (s, 2H), 2.40 (s, 9H), 1.48 (s, 4H), 0.89 (s, 3H).
^{13}C NMR (101 MHz; DMSO-d_6) δ 140.05, 135.52, 133.39, 130.42, 124.31—114.71, 118.68, 23.81, 23.11, 20.92, 19.93, 13.24.
^{19}F NMR (376 MHz; DMSO-d_6) δ -78.66 (s, 6F).
^{31}P NMR (162 MHz; DMSO-d_6) δ 24.15 (s, 1P).
(ESI) m/z: $[M]^+$ Calcd $C_{25}H_{30}P$ for 362.49; Found 361.2

Butyl tris(4-methylphenyl)phosphonium bis(trifluoromethanesulfonyl)imide (4Me-NTf$_2$)

Synthesized following SP2. Yield (80 %)

Single crystals of the iodide salt were grown from slow diffusion of hexanes in a saturated solution of the compound in tetrahydrofuran. Single crystals of the NTf_2 salt were grown from slow diffusion of methyl tertbuytl ether into a saturated solution of the compound in ethanol.

^{1}H NMR (400 MHz; DMSO-d_6) δ 7.67—7.58 (m, 12H), 3.44 (s, 2H), 2.44 (s, 9H), 1.48 (s, 4H), 0.88 (s, 3H).
^{13}C NMR (101 MHz; DMSO-d_6) δ 145.59, 133.43, 130.79, 124.31—114.71, 115.53, 23.85, 23.17, 21.2, 20.32, 13.29.
^{19}F NMR (376 MHz; DMSO-d_6) δ -78.65 (s, 6F).
^{31}P NMR (162 MHz; DMSO-d_6) δ 23.68 (s, 1P).
(ESI) m/z: $[M]^+$ Calcd $C_{25}H_{30}P$ for 362.49; Found 361.2

Butyl tris(4-fluorophenyl)phosphonium bis(trifluoromethanesulfonyl)imide (4F-NTf$_2$)

Synthesized following SP1. Yield (80 %)

Single crystals of the iodide salt were grown from slow diffusion of hexanes in a saturated solution of the compound in ethanol. Single crystals of the NTf_2 salt were grown from slow diffusion of diethyl ether into a saturated solution of the compound in ethyl acetate.

^{1}H NMR (400 MHz; DMSO-d_6) δ 7.91 (d, J = 3.4 Hz, 6H), 7.65 (s, 6H), 3.57 (s, 2H), 1.48 (s, 4H), 0.89 (s, 3H).
^{13}C NMR (101 MHz; DMSO-d_6) δ 166.05, 136.99, 124.30—114.69, 117.89, 115.18—114.25, 23.64, 23.17, 20.41, 13.23.
^{19}F NMR (376 MHz; DMSO-d_6) δ -78.66—-78.67 (m, 6F), -102.26 (t, J = 2.5 Hz, 3F).
^{31}P NMR (162 MHz; DMSO-d_6) δ 24.21 (s, 1P).
(ESI) m/z: $[M]^+$ Calcd $C_{22}H_{21}F_3P$ for 373.38; Found 373.1

Butyl tris(4-methoxyphenyl)phosphonium bis(trifluoromethanesulfonyl)imide (4MeO-NTf$_2$)

Synthesized following SP1. Yield (65 %)

Single crystals of the iodide salt were grown from slow evaporation of a saturated solution of isopropyl alcohol. Single crystals of the NTf_2 salt grew spontaneously upon cooling of the melted compound.

^{1}H NMR (400 MHz; DMSO-d_6) δ 7.67 (t, J = 9.1 Hz, 6H), 7.29 (s, 6H), 3.88 (s, 9H), 1.47 (s, 4H), 0.89 (s, 3H).
^{13}C NMR (101 MHz; DMSO-d_6) δ 163.99, 135.47, 124.31—114.71, 115.88, 109.61, 55.86, 23.89, 23.18, 21.04, 13.33.
^{19}F NMR (376 MHz; DMSO-d_6) δ -78.66 (s, 6F).
^{31}P NMR (162 MHz; DMSO-d_6) δ 22.34 (s, 1P).
(ESI) m/z: $[M]^+$ Calcd $C_{25}H_{30}O_3P$ for 409.49; Found 409.2

Butyl tris(4-trifluoromethylphenyl)phosphonium bis(trifluoromethanesulfonyl)imide (4CF$_3$-NTf$_2$)

Synthesized following SP2. Yield (74 %)

Single crystals of the iodide salt were grown from slow evaporation of ethanol. Single crystals of the NTf_2 salt were grown from slow diffusion of methyl tertbutyl ether in a saturated solution of the compound in ethyl acetate.

^{1}H NMR (400 MHz; DMSO-d_6) δ 8.15 (d, J = 15.4 Hz, 12H), 3.79 (s, 2H), 1.52 (s, 4H), 0.91 (d, J = 5.5 Hz, 3H).
^{13}C NMR (101 MHz; DMSO-d_6) δ 135.25, 135.03—134.21, 127.04, 127.35—119.19, 122.60, 117.90, 23.44, 23.23, 19.49, 13.15.
^{19}F NMR (376 MHz; DMSO-d_6) δ -62.13 (s, 9F), -78.72 (d, J = 0.7 Hz, 6F).
^{31}P NMR (162 MHz; DMSO-d_6) δ 26.20 (s, 1P).
(ESI) m/z: $[M]^+$ Calcd $C_{25}H_{21}F_9P$ for 523.40; Found 523.8

Butyl tri(2-furyl)phosphonium bis(trifluoromethanesulfonyl)imide (TFP-NTf$_2$)

Synthesized following SP2. Yield (77 %)

Single crystals of the iodide salt were grown from slow diffusion of methyl tertbutyl ether in a saturated solution of the compound in chloroform.

^{1}H NMR (400 MHz; DMSO-d_6) δ 8.49 (s, 3H), 7.91 (s, 3H), 7.01 (s, 3H), 3.50 (t, J = 7.0 Hz, 2H), 1.64 (s, 2H), 1.47 (dd, J = 13.5, 6.6 Hz, 2H), 0.90 (t, J = 6.8 Hz, 3H).
^{13}C NMR (101 MHz; DMSO-d_6) δ 154.65, 130.67, 131.29—129.91, 119.58, 113.28, 23.39, 22.85, 20.50, 13.11.
^{31}P NMR (162 MHz; DMSO-d_6) δ -11.50 (s, 1P).
(ESI) m/z: $[M]^+$ Calcd $C_{16}H_{18}O_3P$ for 289.29; Found 289.1

NMR Spectra

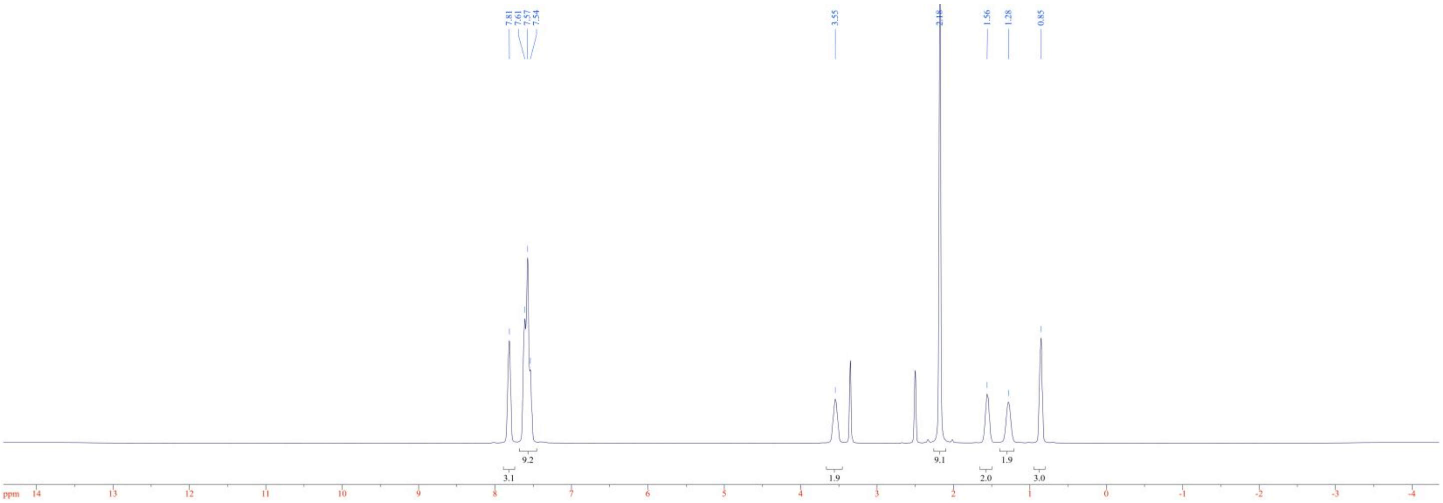

Figure S15. 1H NMR Spectra for 2Me-NTf_2

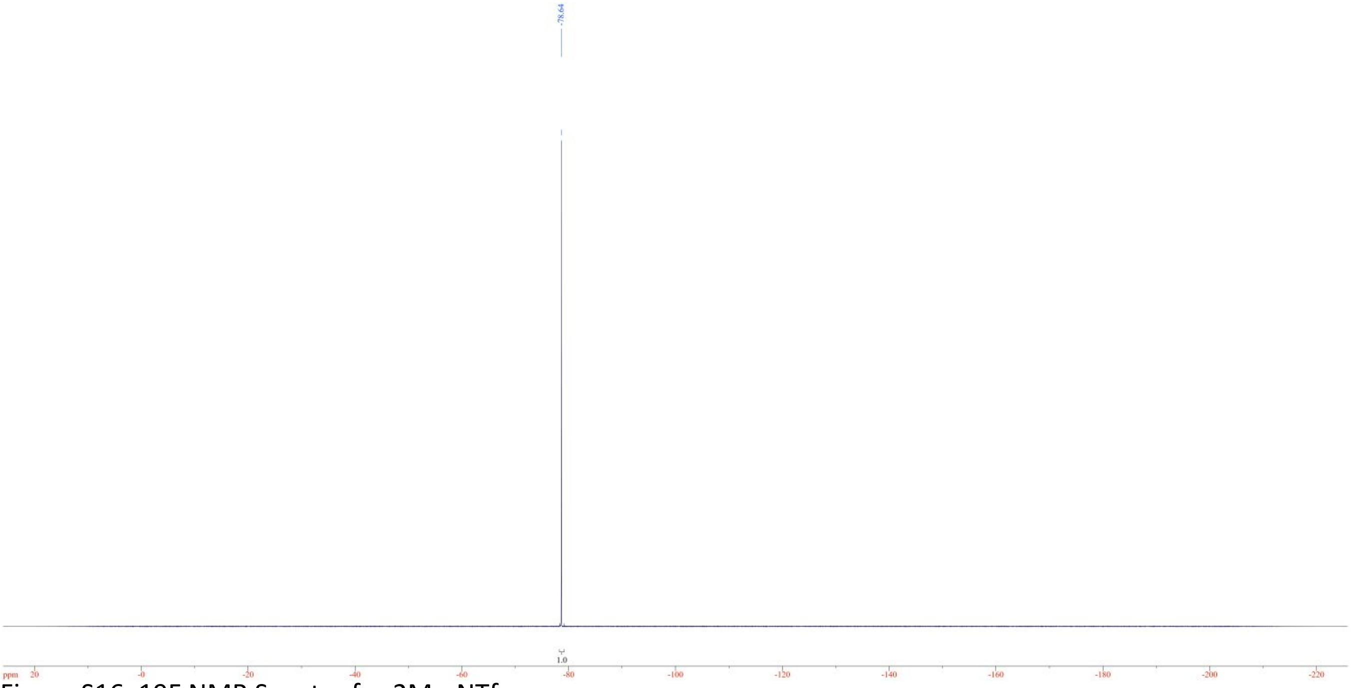

Figure S16. 19F NMR Spectra for 2Me-NTf_2

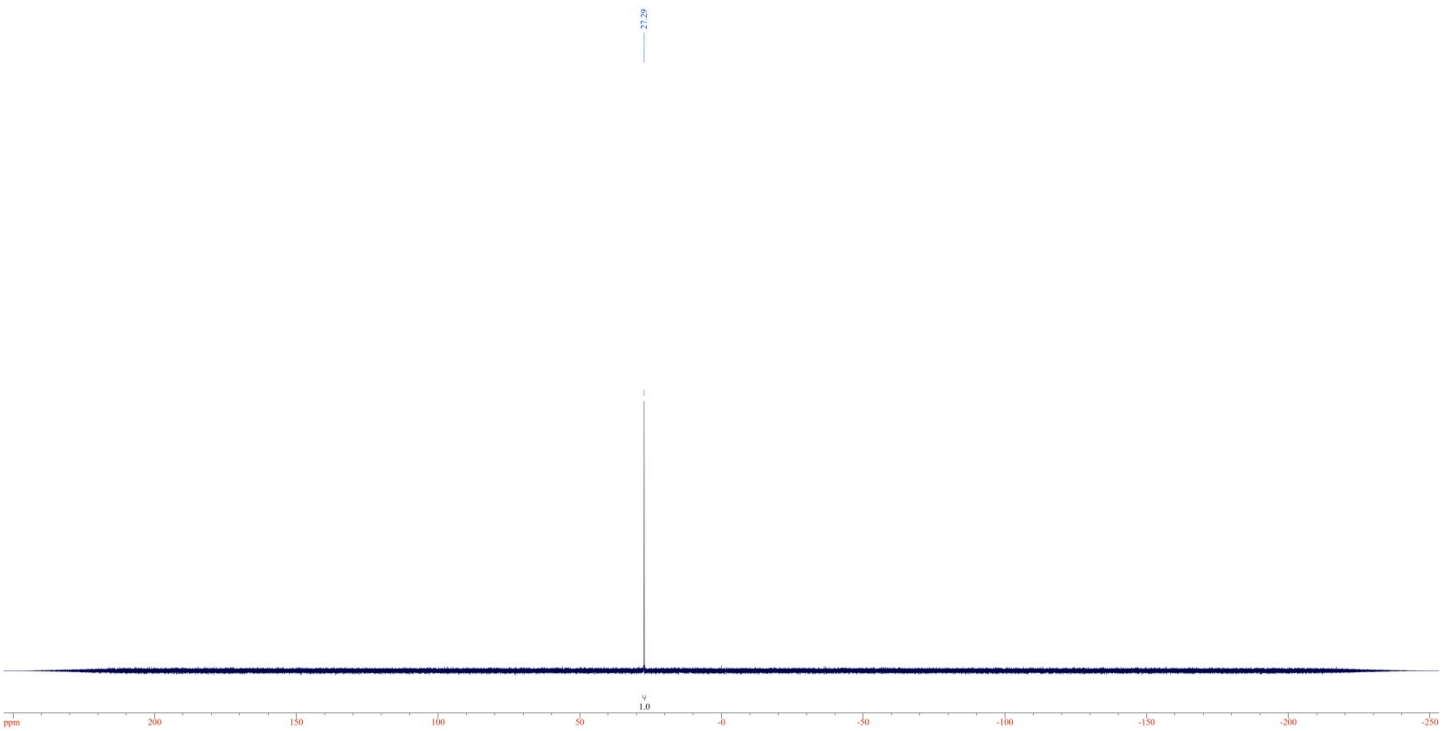

Figure S17. 31P NMR Spectra for 2Me-NTf_2

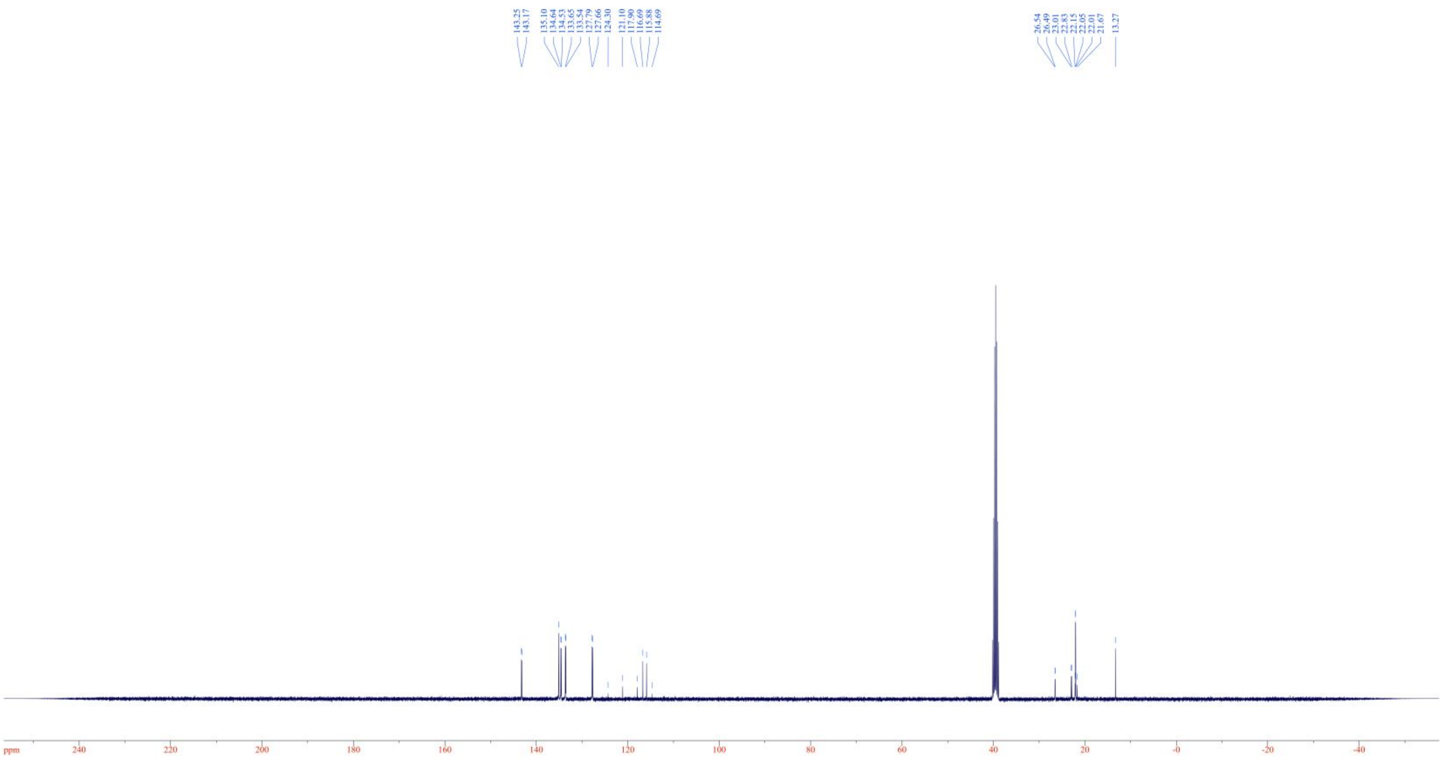

Figure S18. 13C NMR Spectra for 2Me-NTf_2

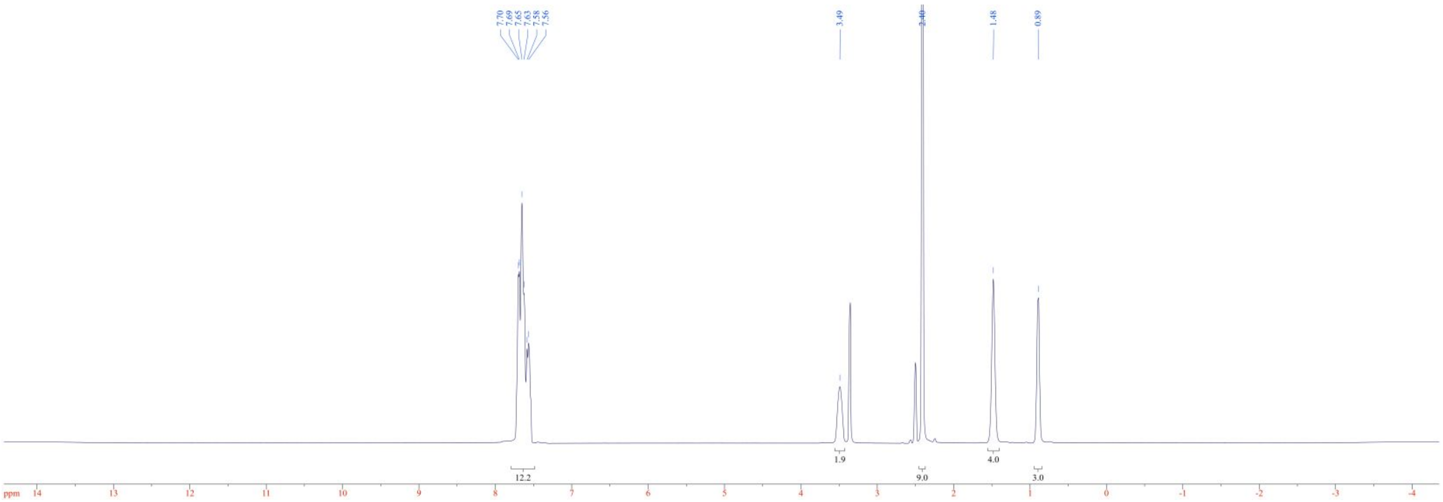

Figure S19. 1H NMR Spectra for 3Me-NTf_2

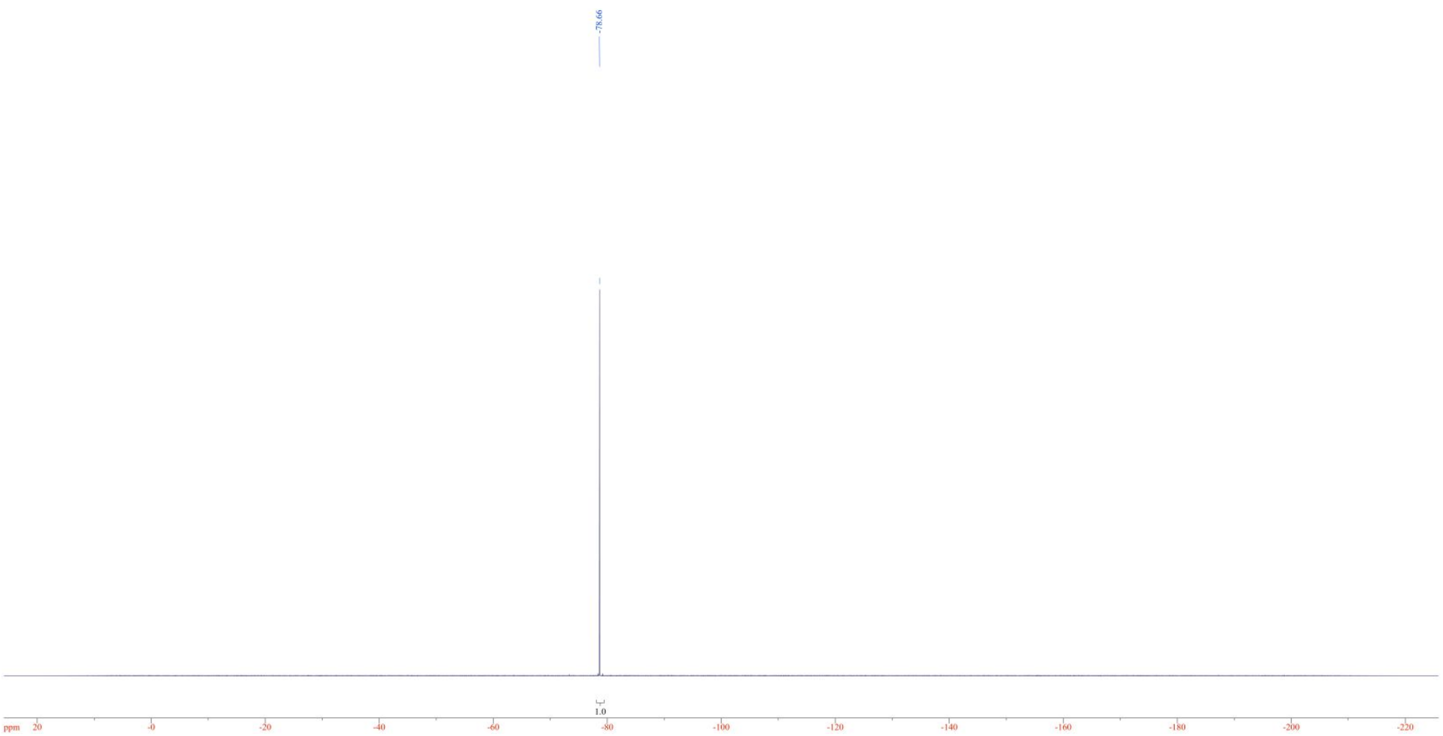

Figure S20. 19F NMR Spectra for 3Me-NTf_2

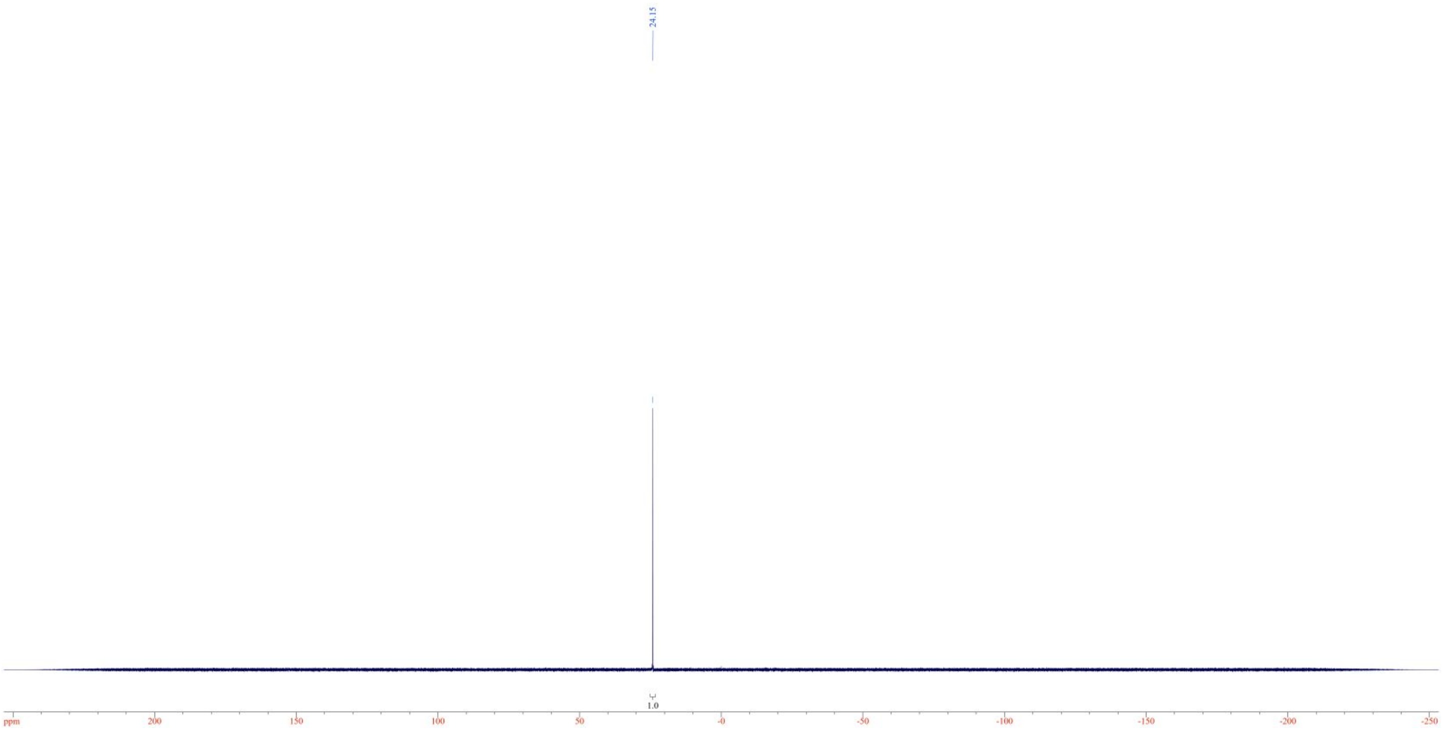

Figure S21. 31P NMR Spectra of 3Me-NTf_2

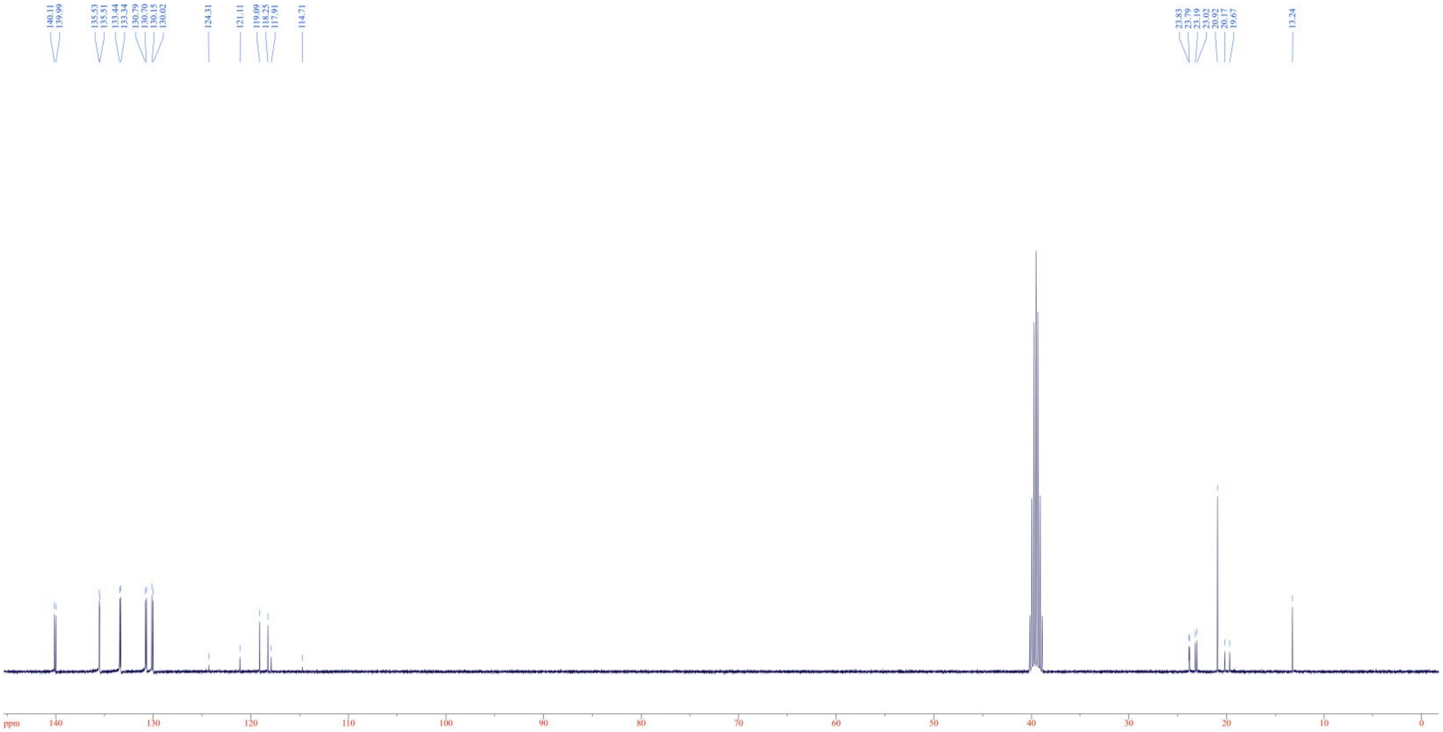

Figure S22. 13C NMR Spectra of 3Me-NTf_2

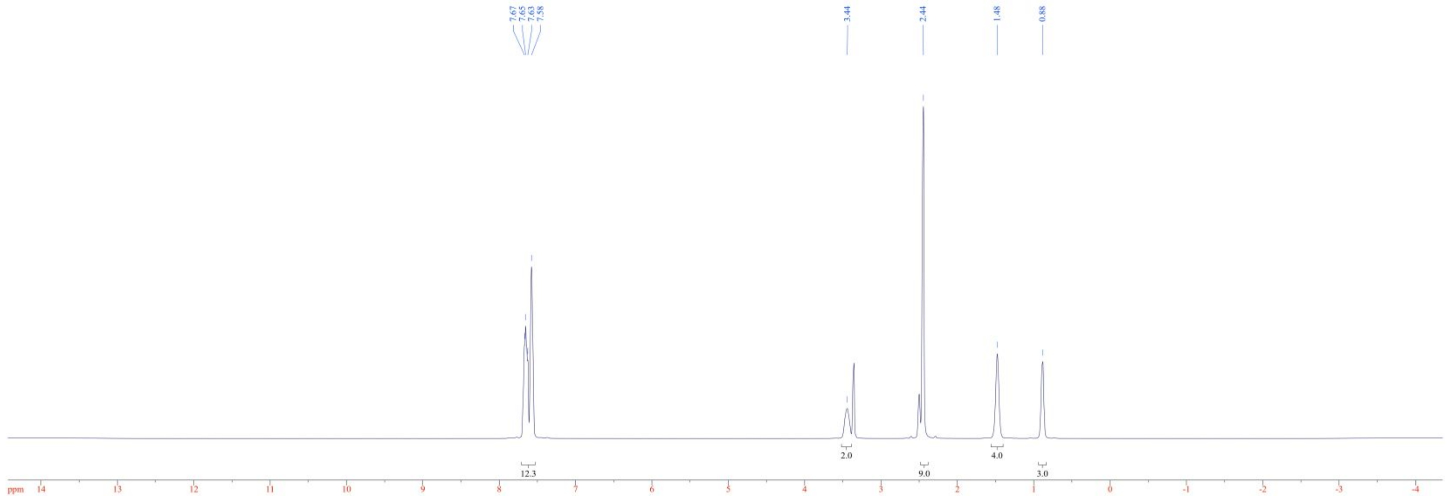

Figure S23. 1H NMR Spectra of 4Me-NTf_2

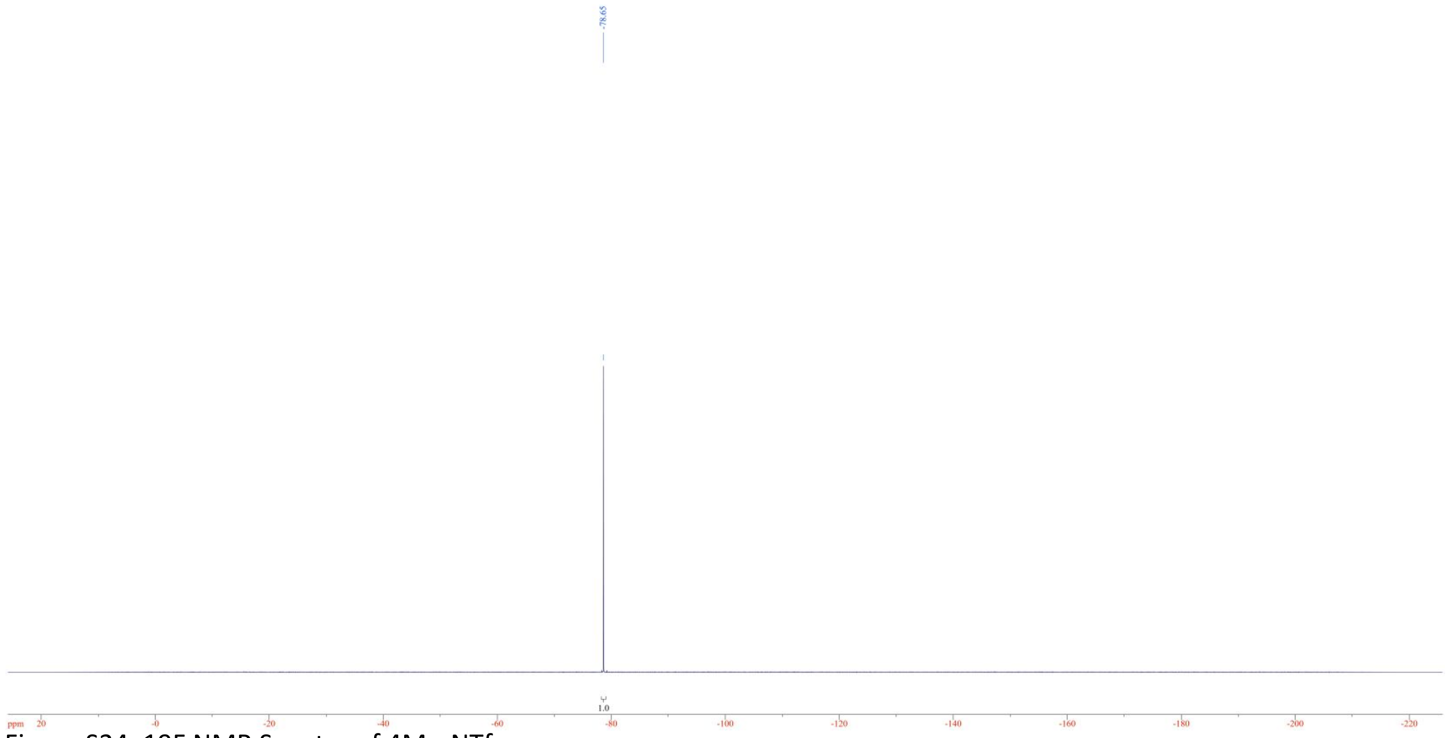

Figure S24. 19F NMR Spectra of 4Me-NTf_2

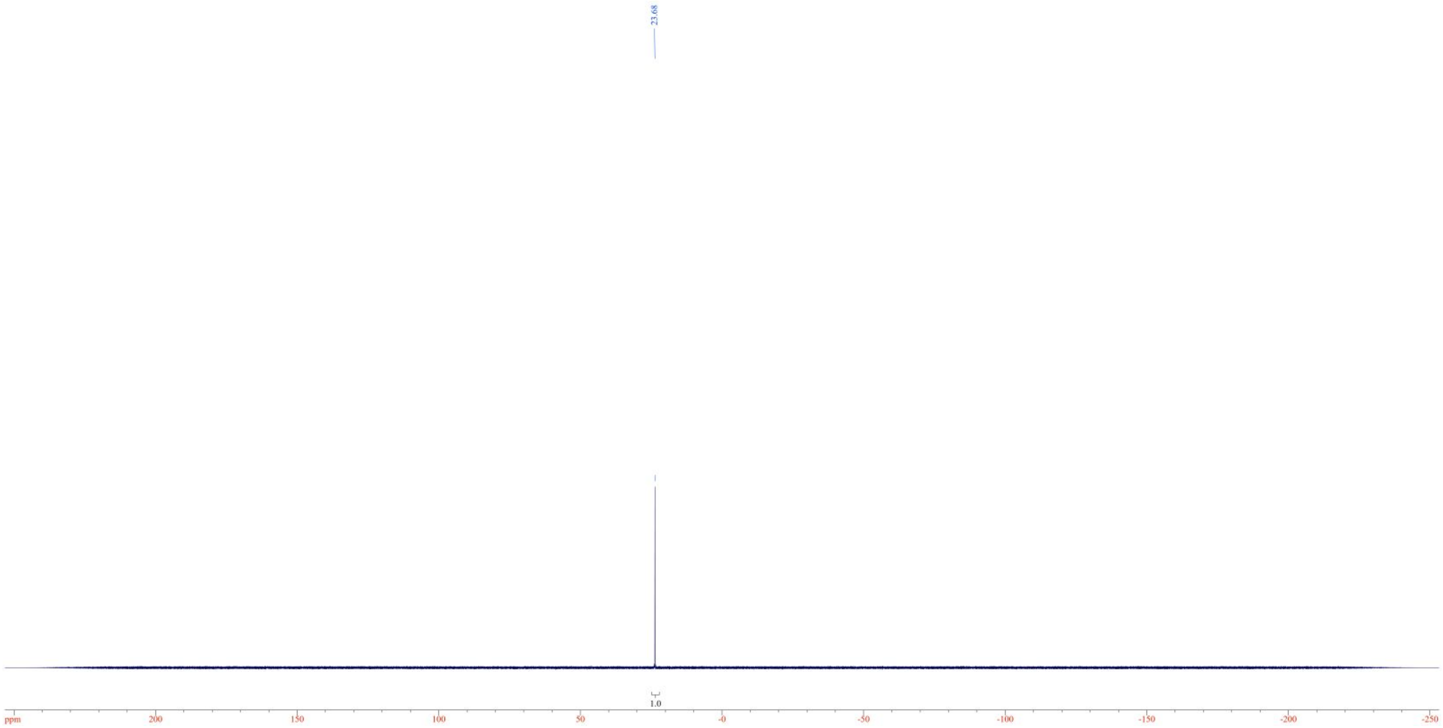

Figure S25. 31P NMR Spectra of 4Me-NTf_2

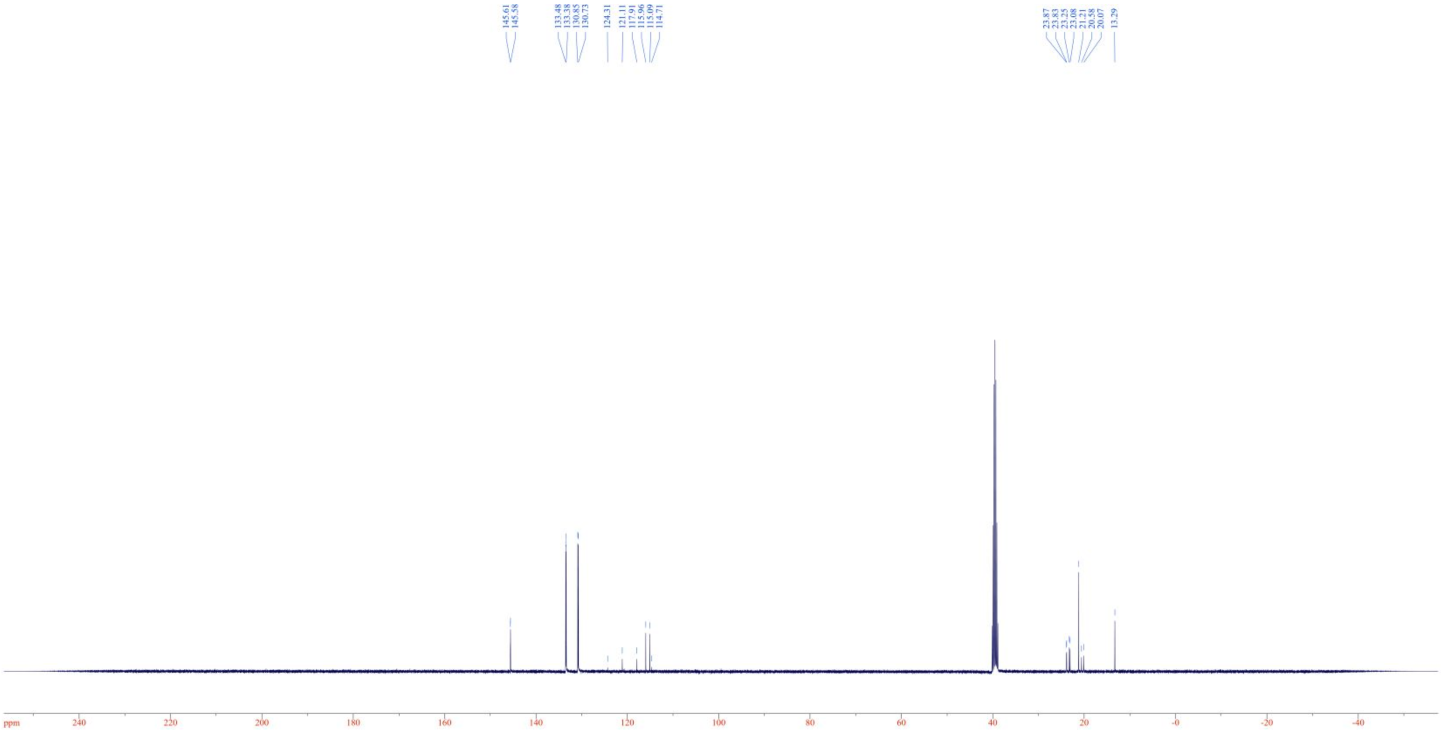

Figure S26. 13C NMR Spectra of 4Me-NTf_2

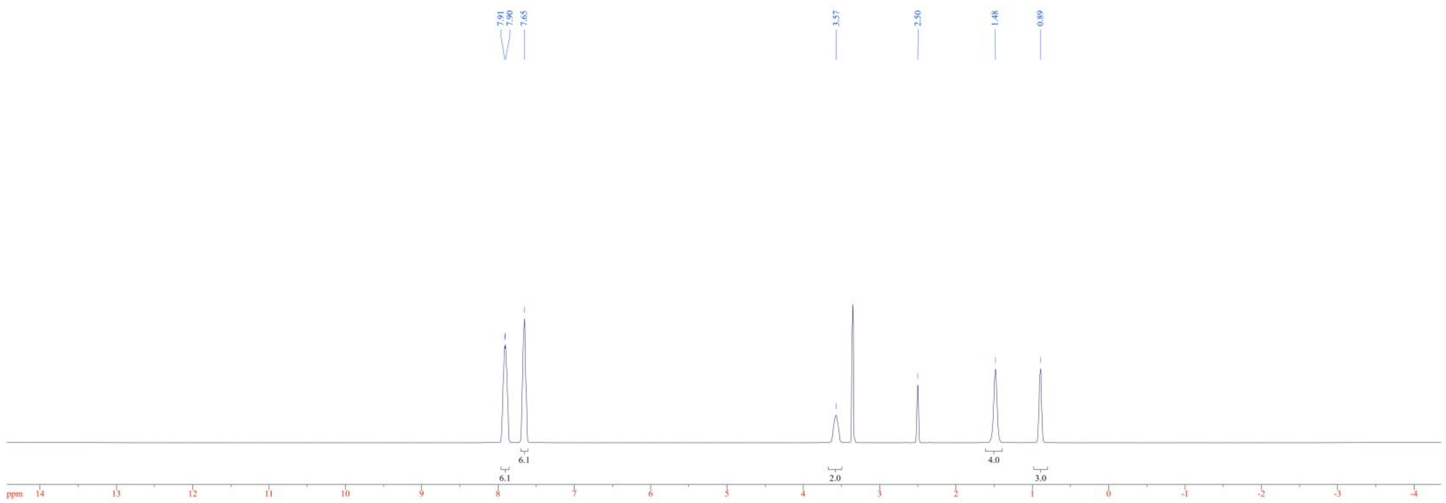

Figure S27. 1H NMR Spectra of 4F-NTf_2

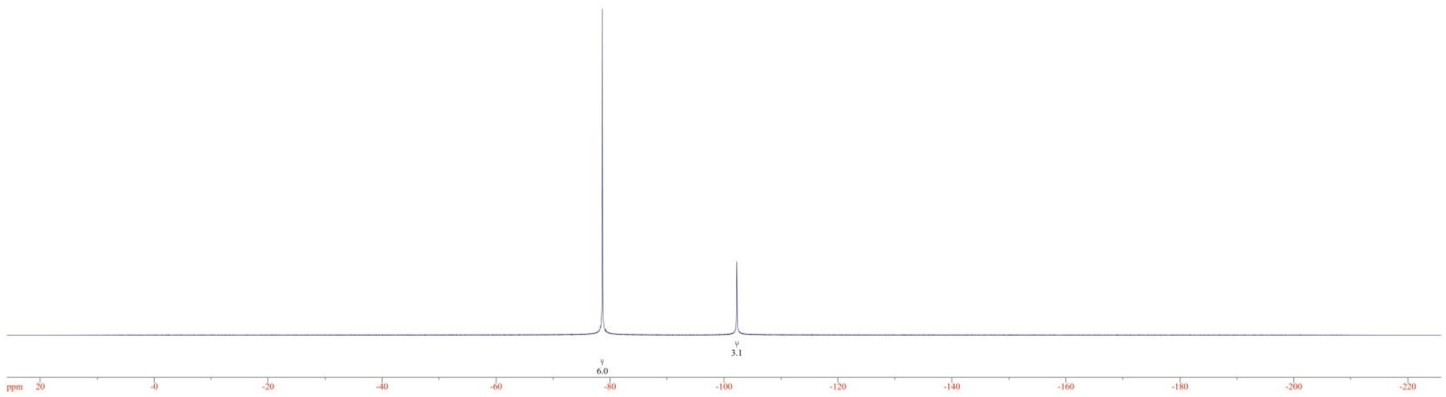

Figure S28. 19F NMR Spectra of 4F-NTf_2

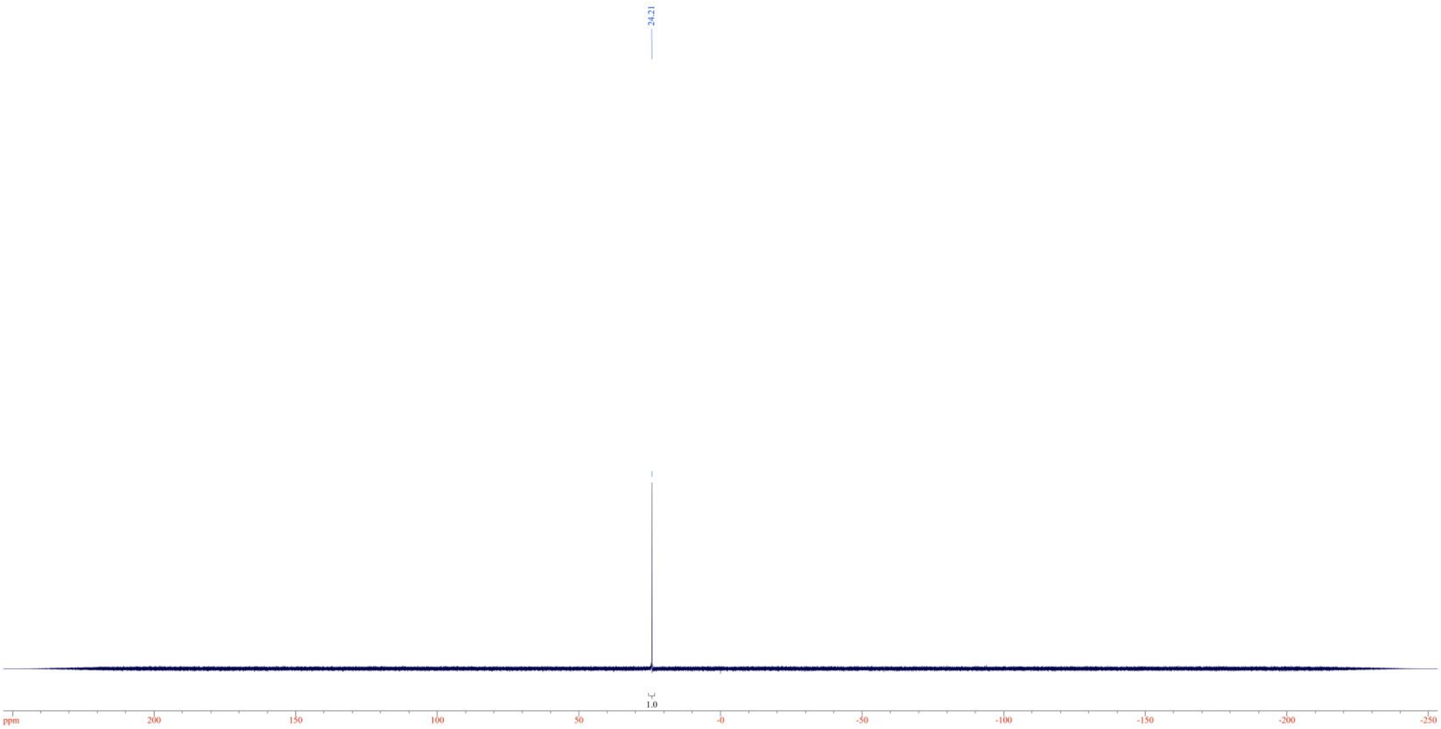

Figure S29. 31P NMR Spectra of 4F-NTf_2

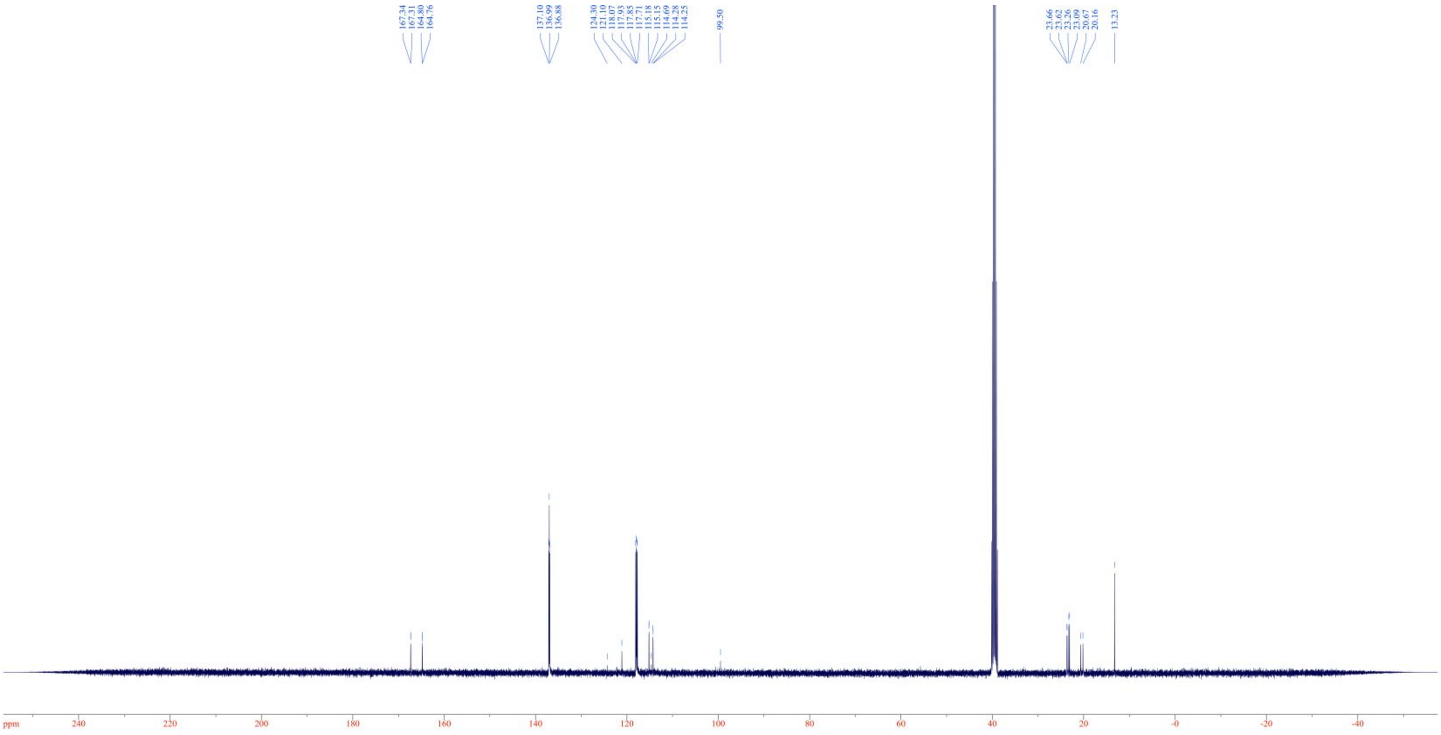

Figure S30. 13C NMR Spectra of 4F-NTf_2

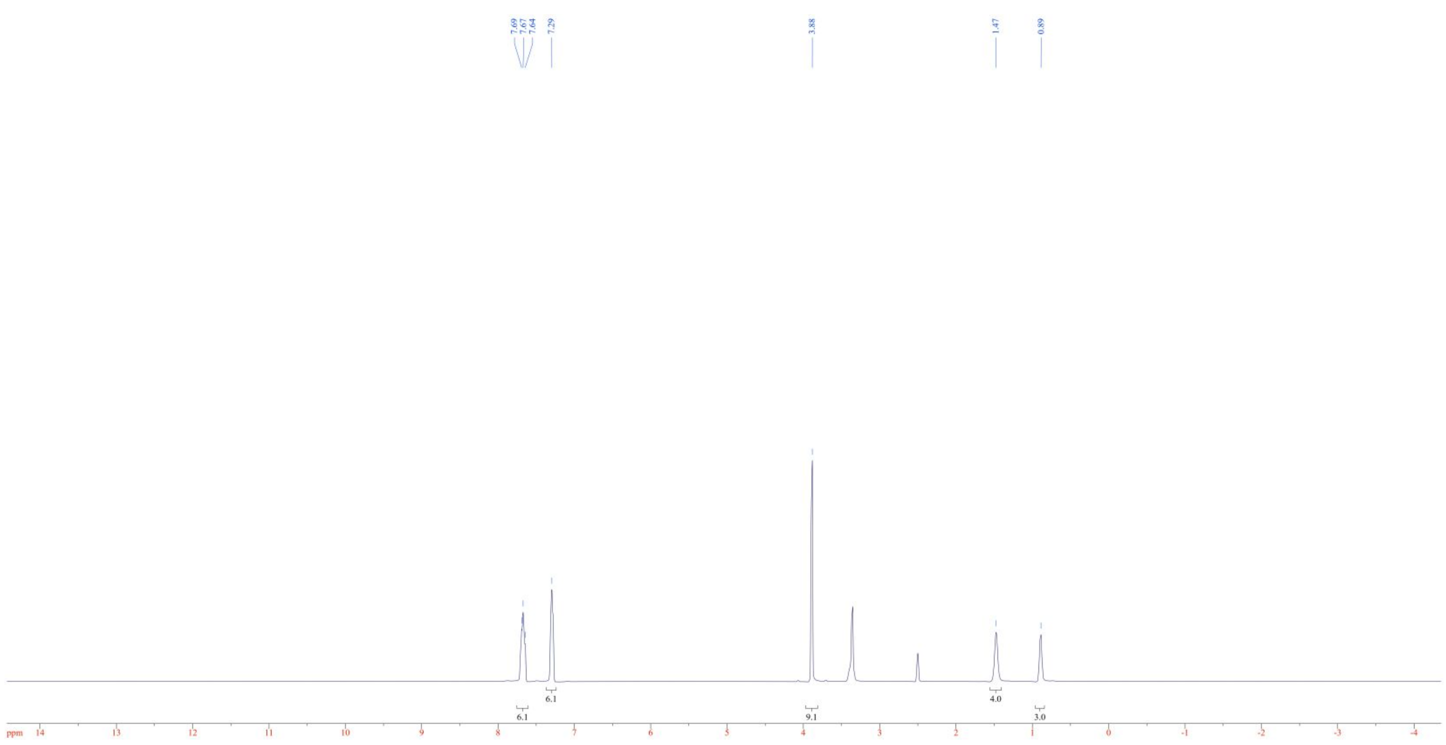

Figure S31. 1H NMR Spectra of 4MeO-NTf_2

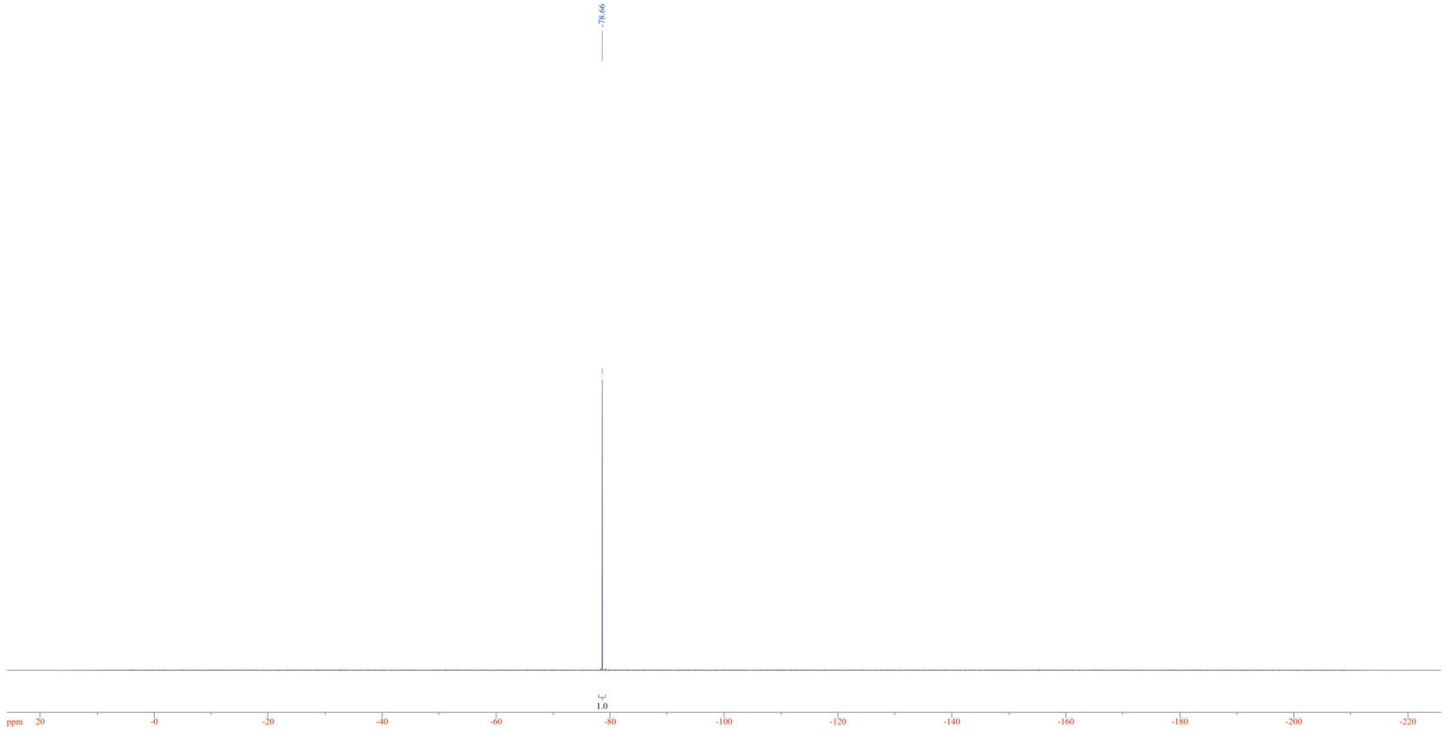

Figure S32. 19F NMR Spectra of 4MeO-NTf_2

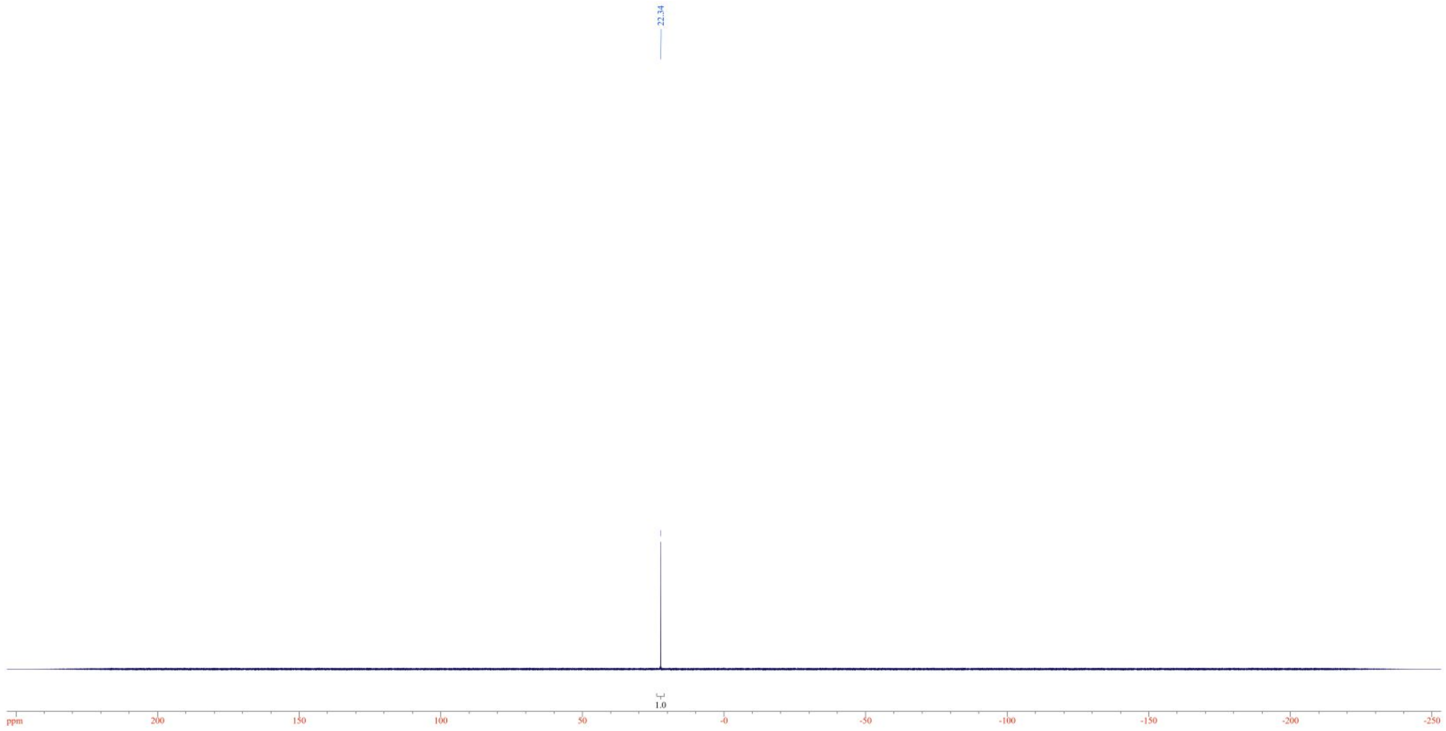

Figure S33. 31P NMR Spectra of 4MeO-NTf_2

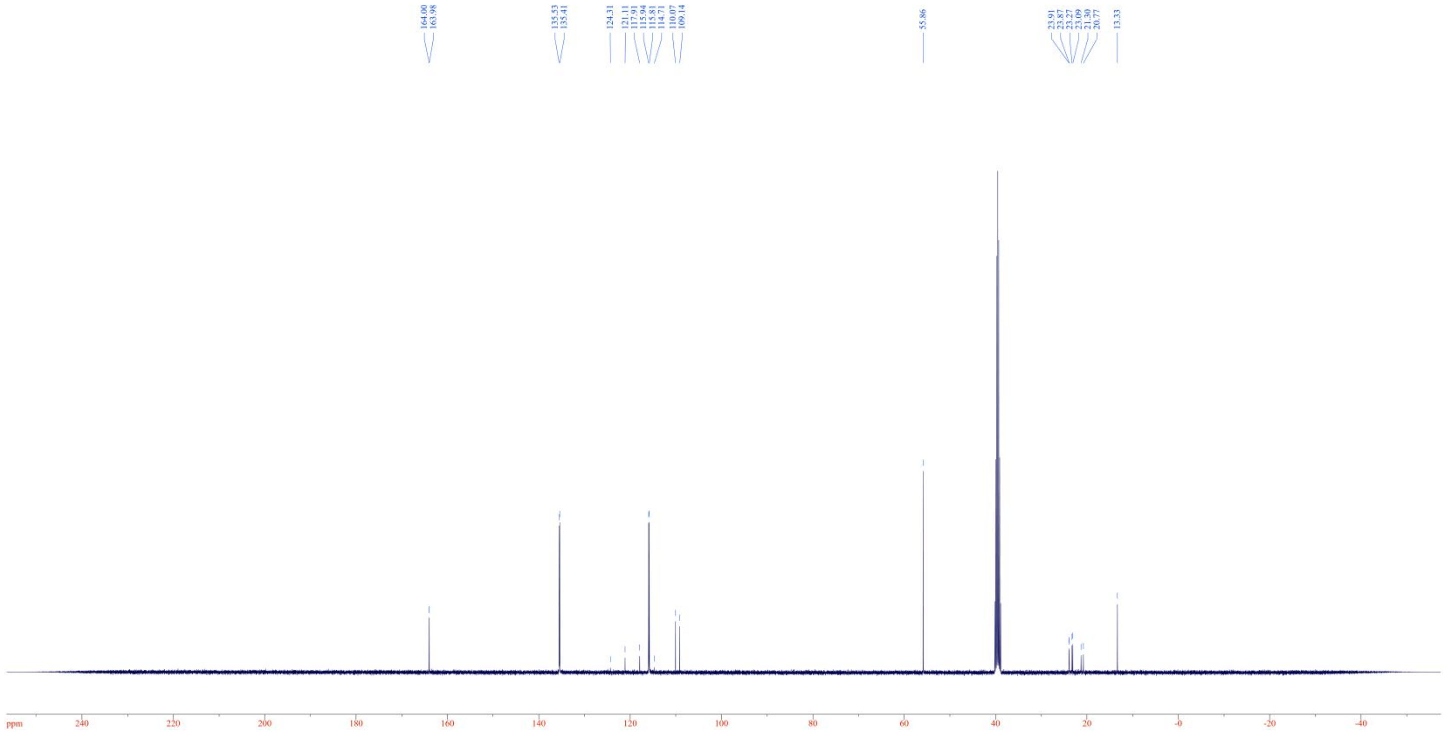

Figure S34. 13C NMR Spectra of 4MeO-NTf_2

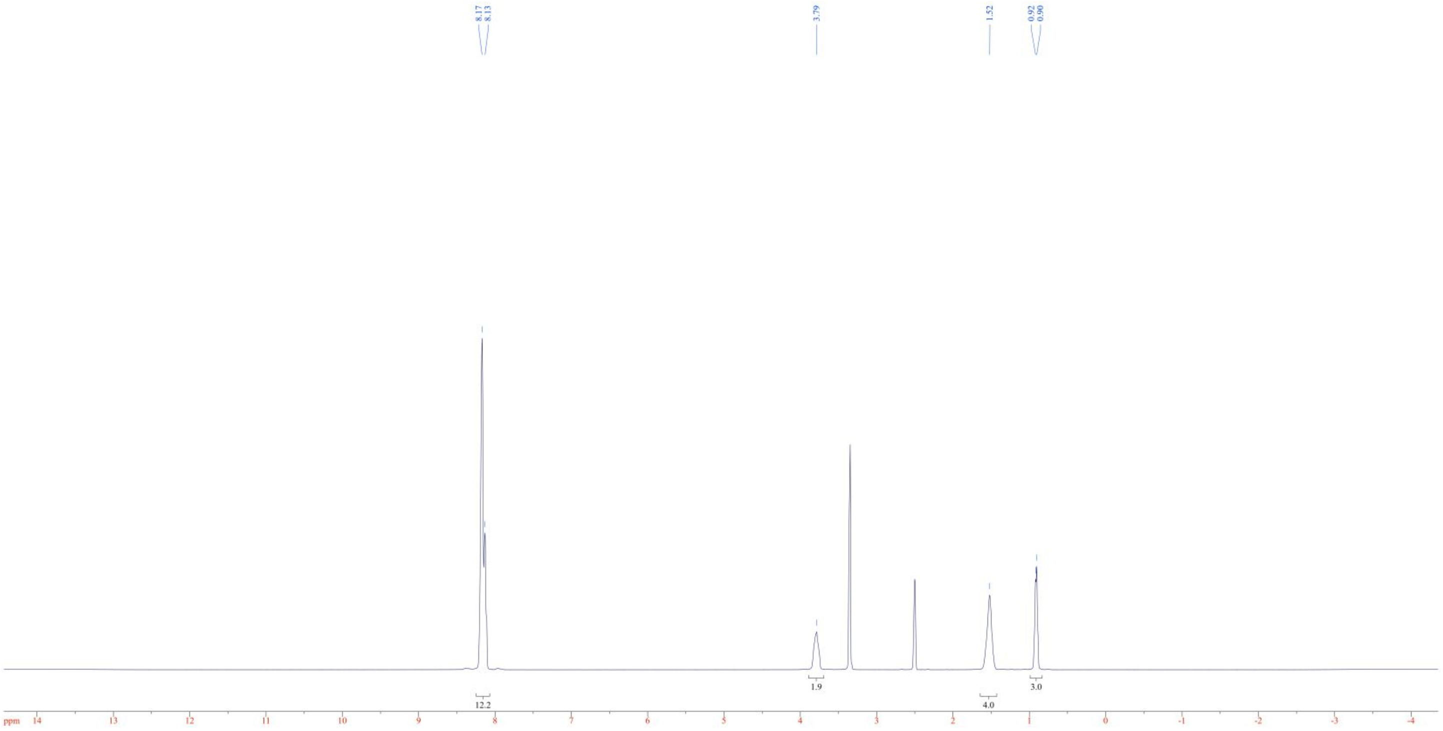

Figure S35. 1H NMR Spectra of $4CF_3$-NTf_2

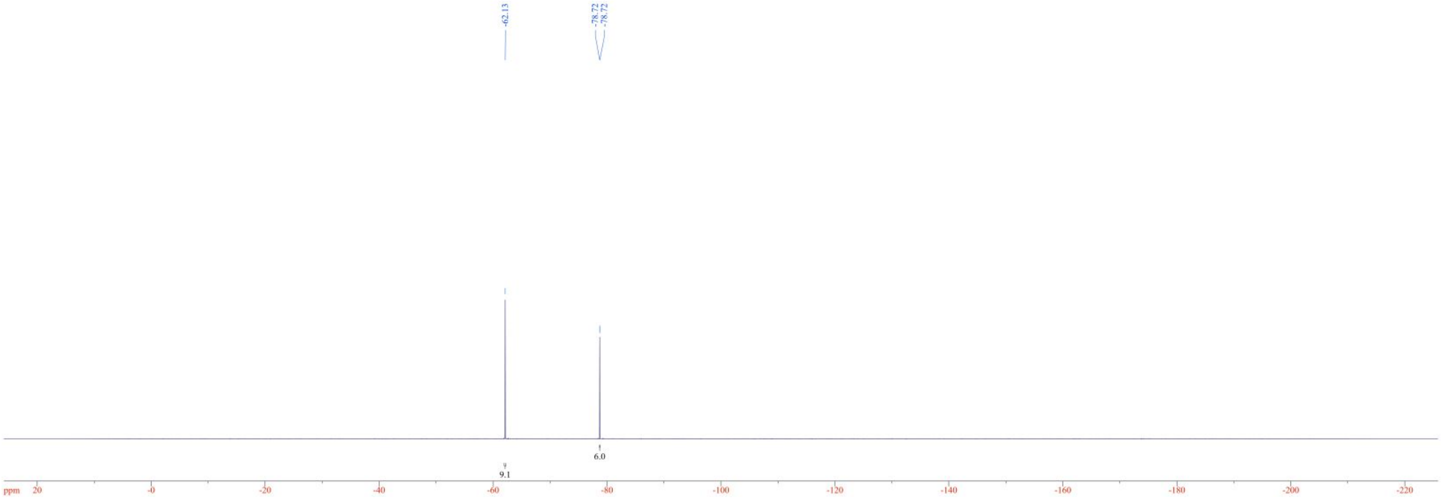

Figure S36. 19F NMR Spectra of $4CF_3$-NTf_2

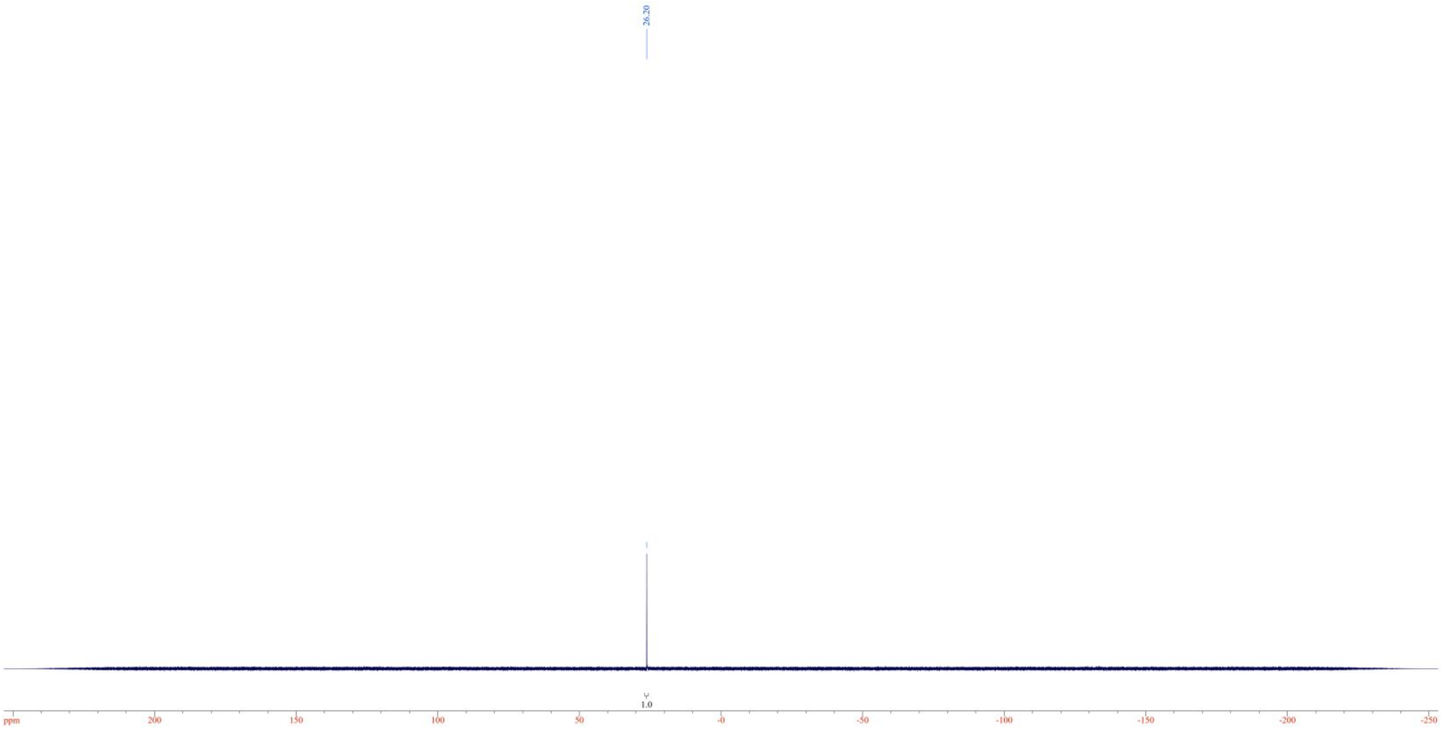

Figure S37. 31P NMR Spectra of $4CF_3$-NTf_2.

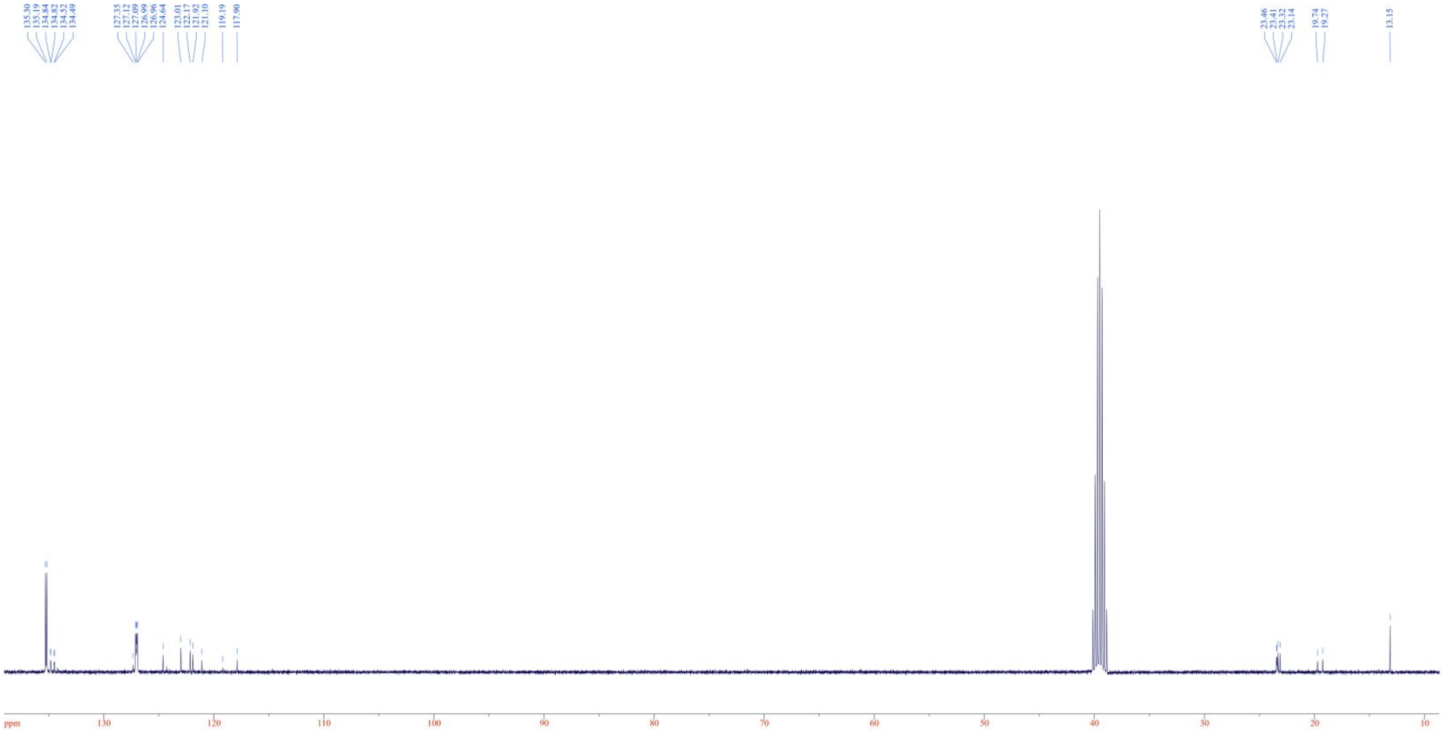

Figure S38. 13C NMR Spectra of $4CF_3$-NTf_2

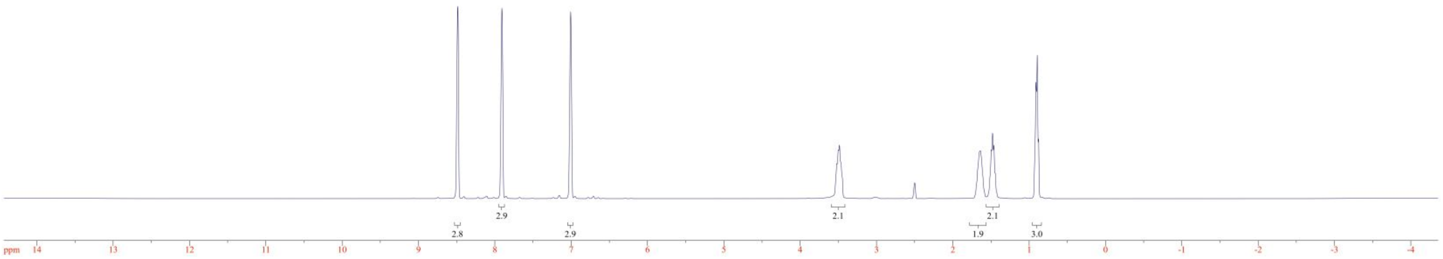

Figure S39. 1H NMR Spectra of TFP-NTf_2

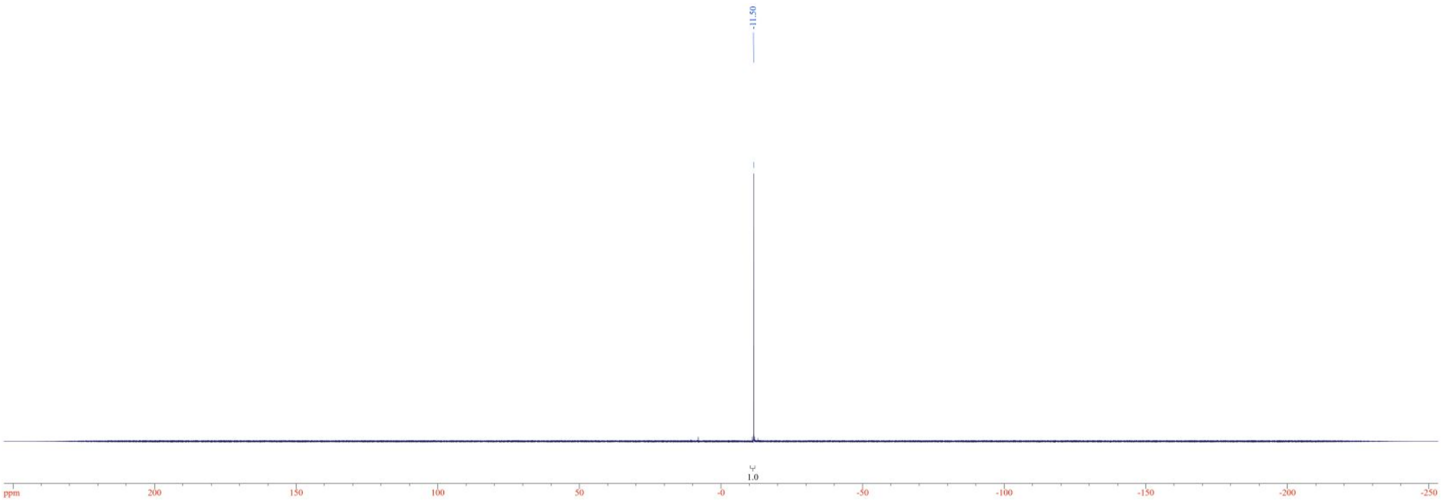

Figure S40. 31P NMR Spectra of TFP-NTf_2

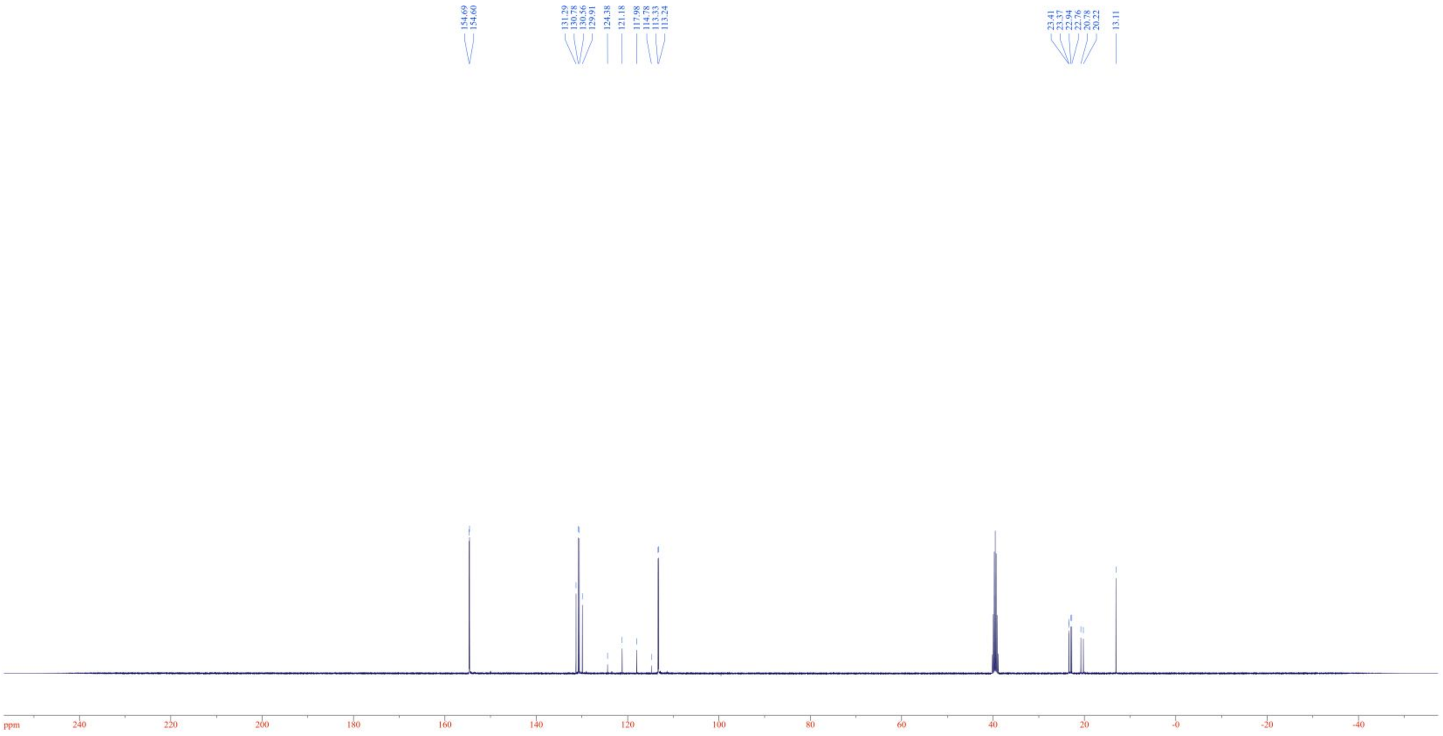

Figure S41. 13C NMR Spectra of TFP-NTf_2

TGA and DSC Graphs

Thermal Decomposition Temperatures

The TGA traces for the compounds are shown in figure S42. In contrast to the phase transitions, all of the compounds show very similar decomposition profiles, with a single large decomposition step accounting for the majority of the weight loss. The compounds lose little weight up until the onset of decomposition at *ca.* 420 °C. Several of the compounds produced a rather significant amount of residual carbonaceous material after the large decomposition step. This residue decomposed when the sample was heated under an atmosphere of air, leaving no remaining material. While admittedly speculative, all of the compounds for which residue remains have a carbon—heteroatom bond in the para position of the aromatic rings, perhaps hinting at the thermal decomposition mechanism for this class of compounds. Thus, while there are profound impacts on phase transitions of the TPP-based ILs, thermal stability appears to remain relatively constant despite the changes in sterics and electronics. A future study of these compounds and their thermal stability is certainly warranted given the high thermal stability observed.

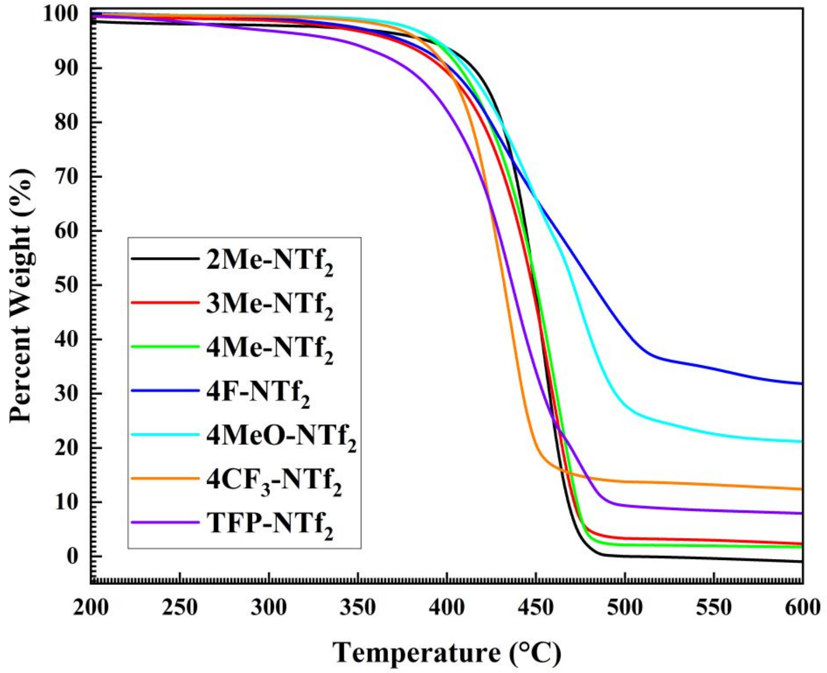

Figure S42. TGA traces for the NTf$_2$-based compounds.

A. Complete TGA traces for the NTf_2 based compounds.

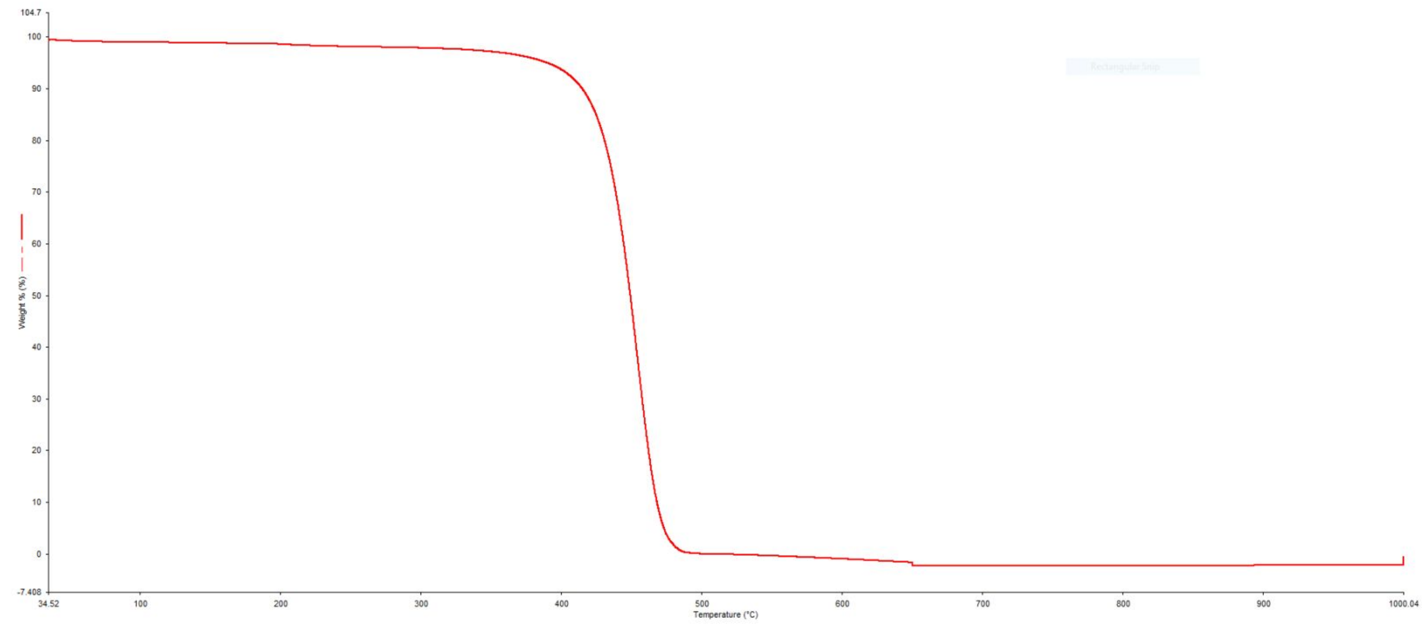

Figure S43. TGA trace for 2Me-NTf_2

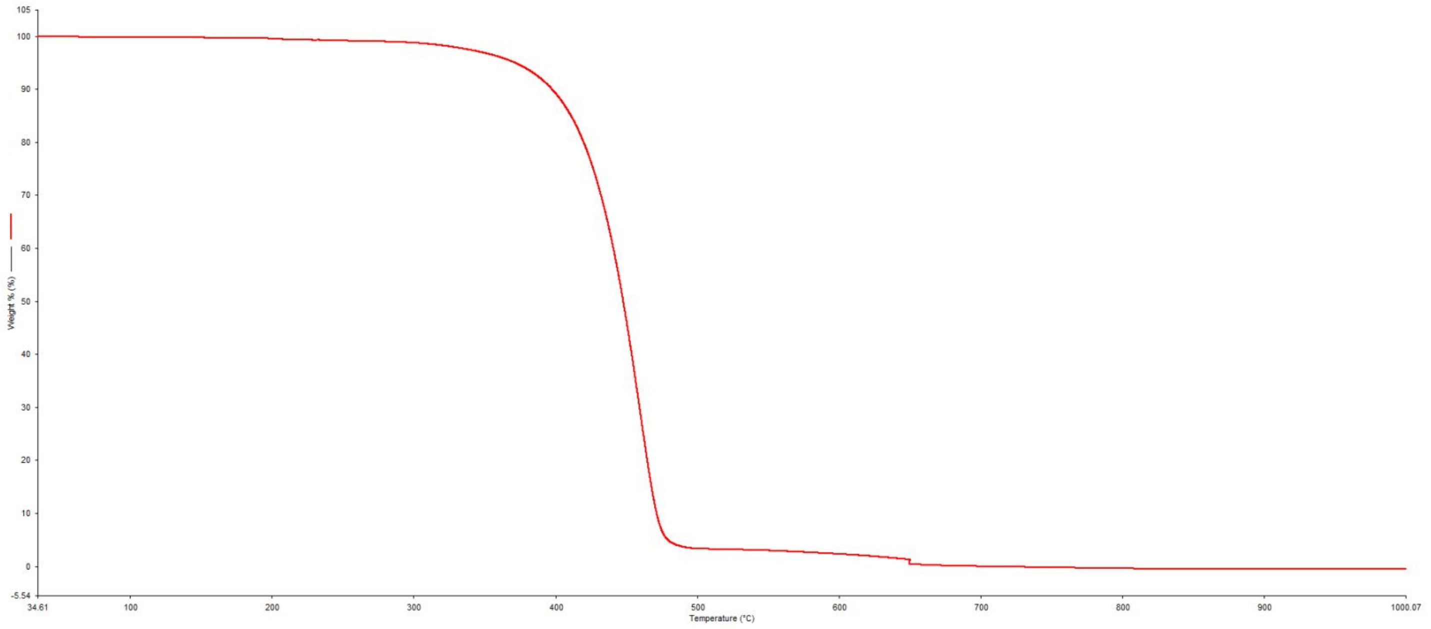

Figure S44. TGA trace for 3Me-NTf_2

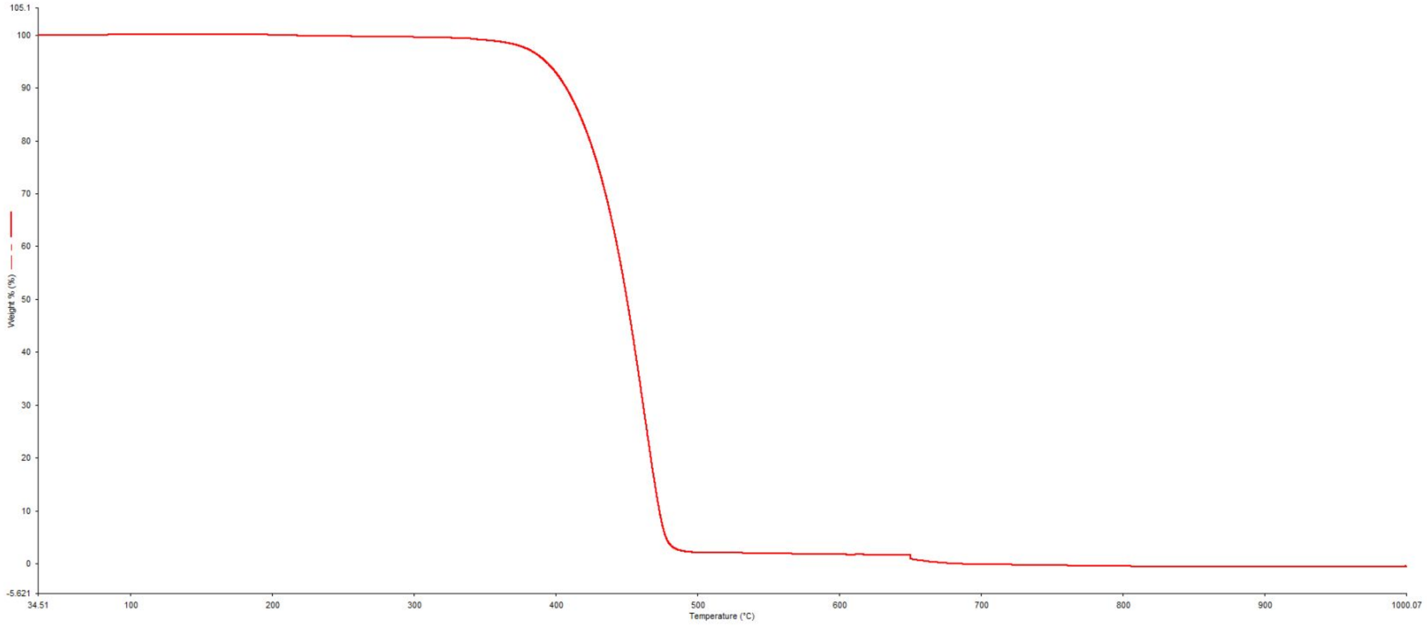

Figure S45. TGA trace for 4Me-NTf_2

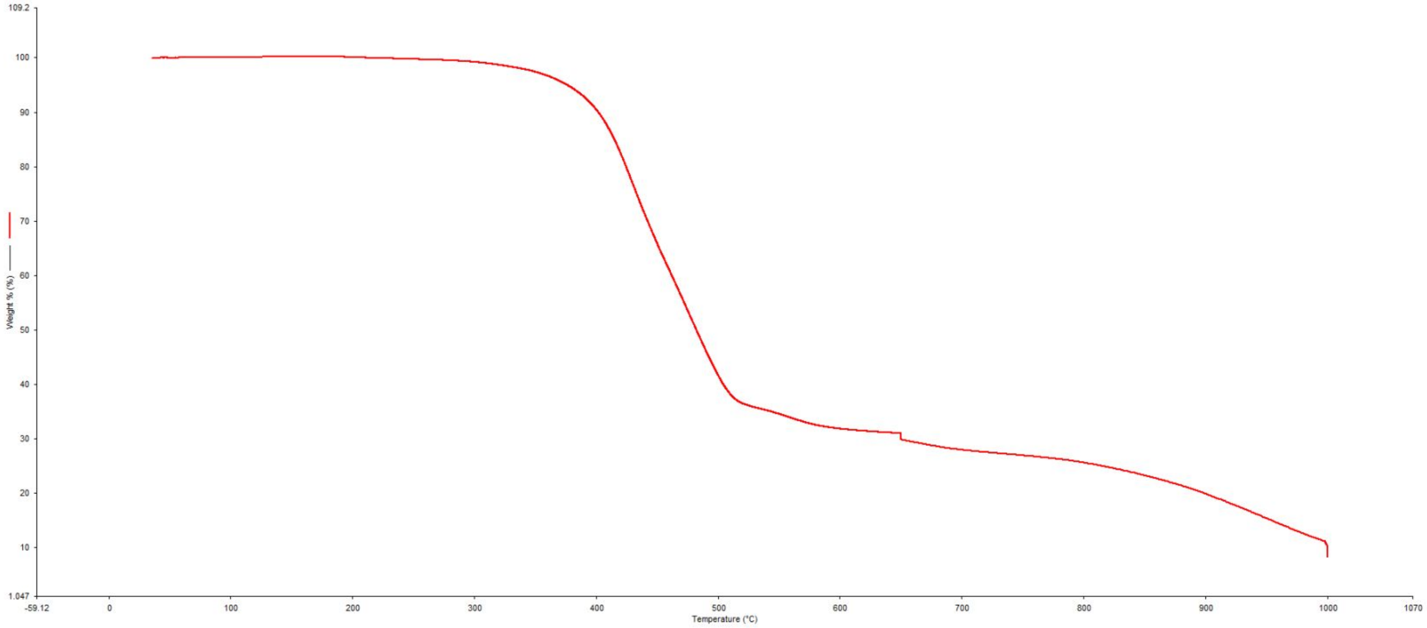

Figure S46. TGA trace for 4F-NTf_2

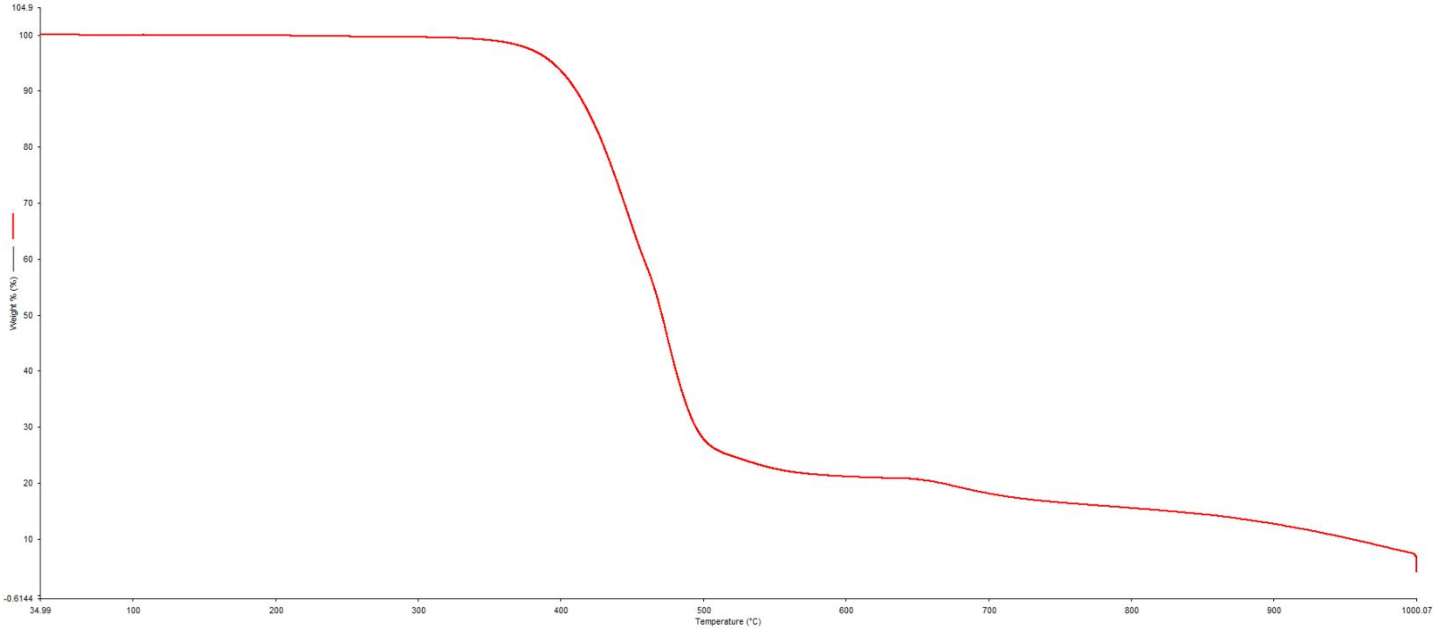

Figure S47. TGA trace for 4MeO-NTf_2.

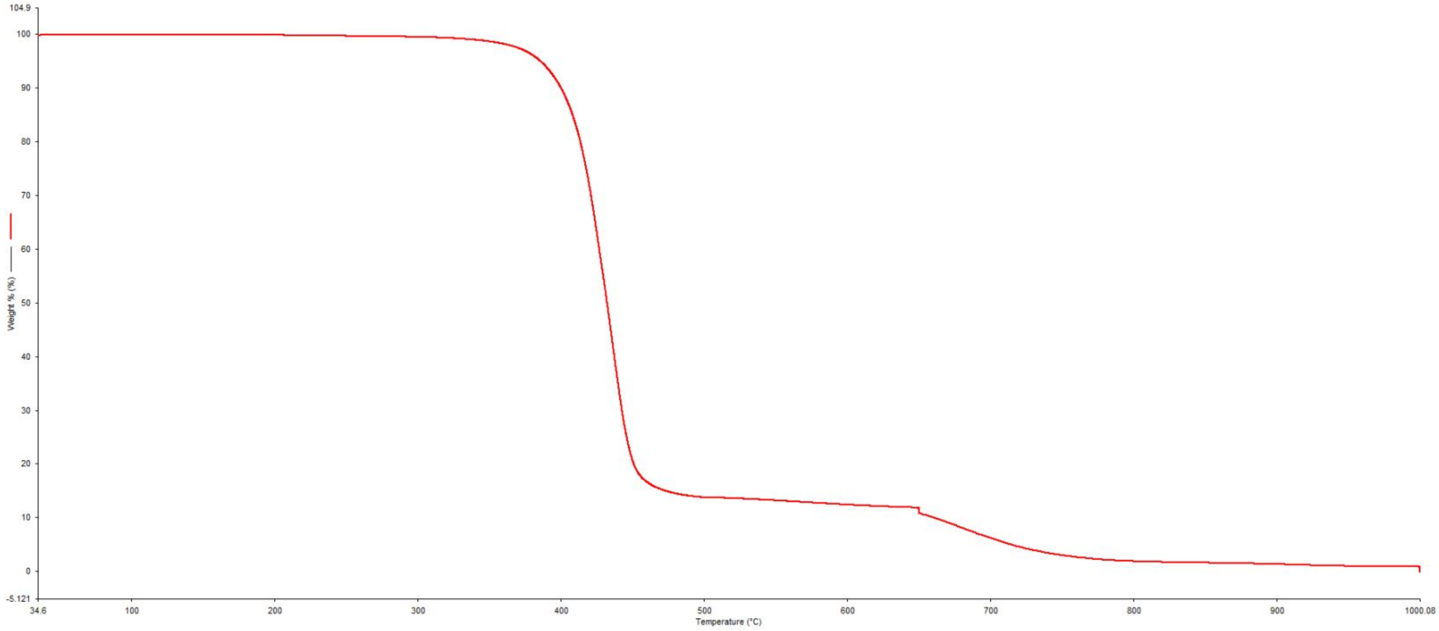

Figure S48. TGA trace for 4CF3-NTf_2

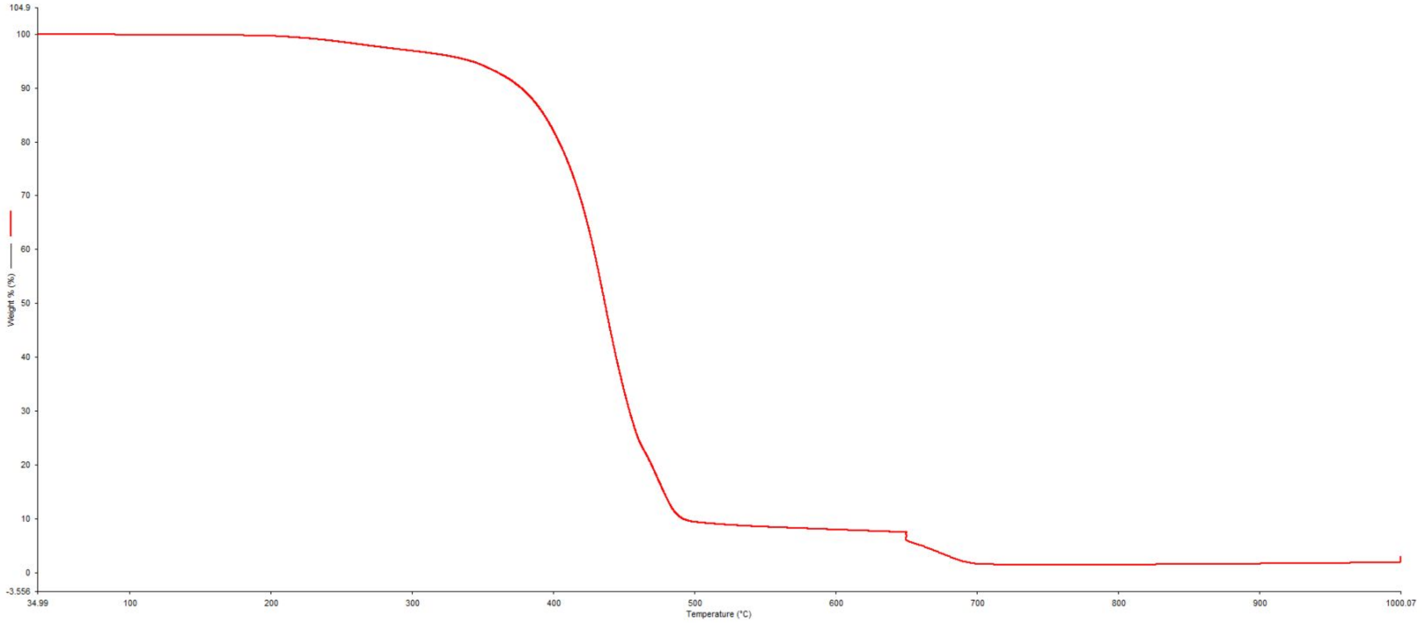

Figure S49. TGA trace for TFP-NTf_2

Differential Scanning Calorimetry traces for compounds

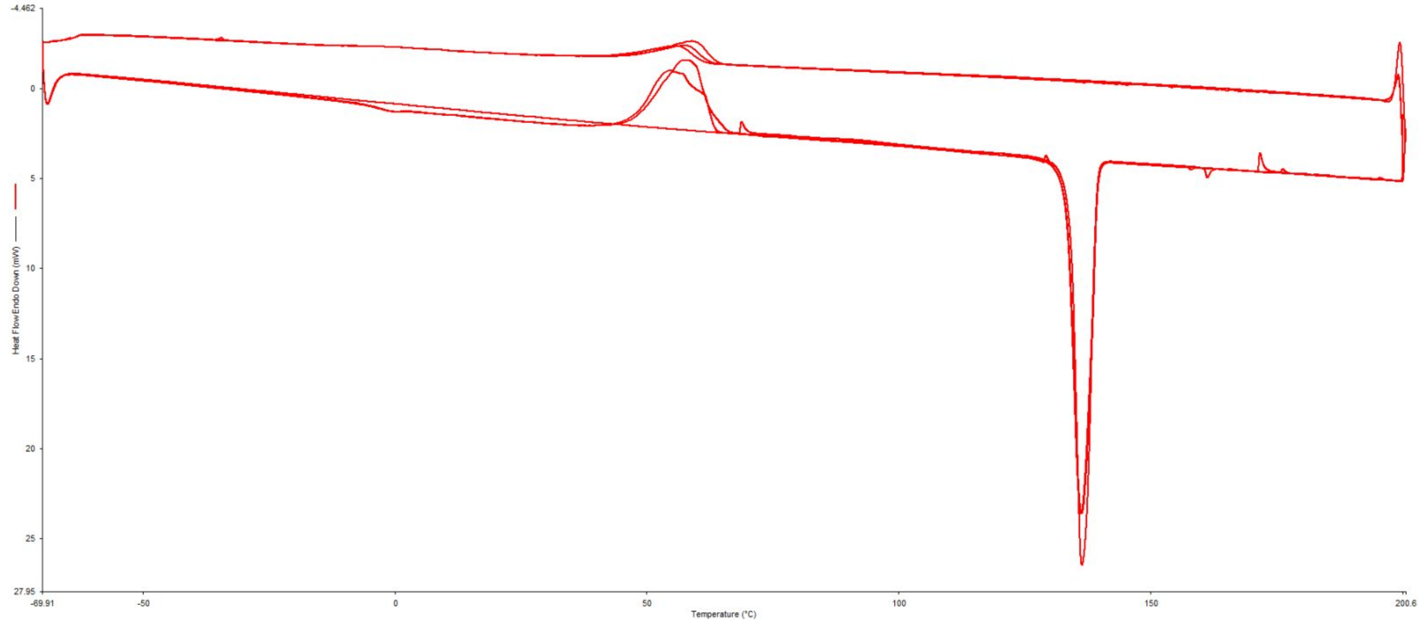

Figure S50. DSC trace of 2Me-NTf_2

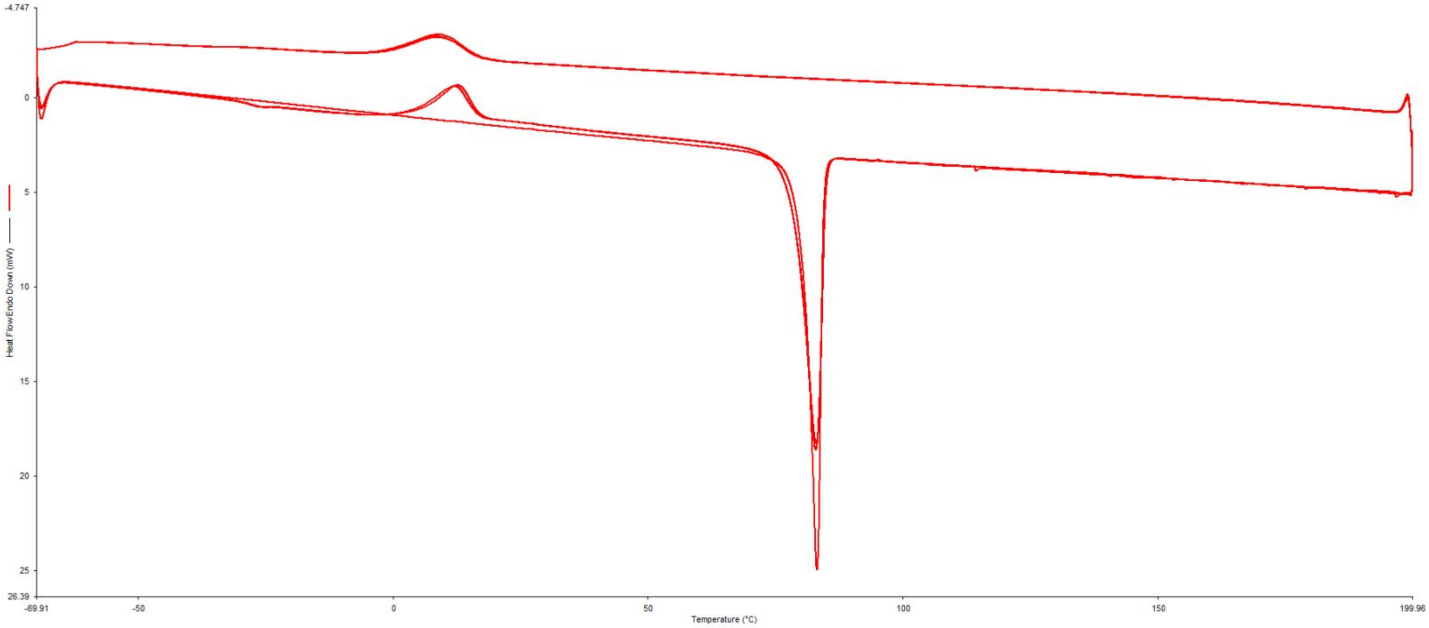

Figure S51. DSC trace of 3Me-NTf_2

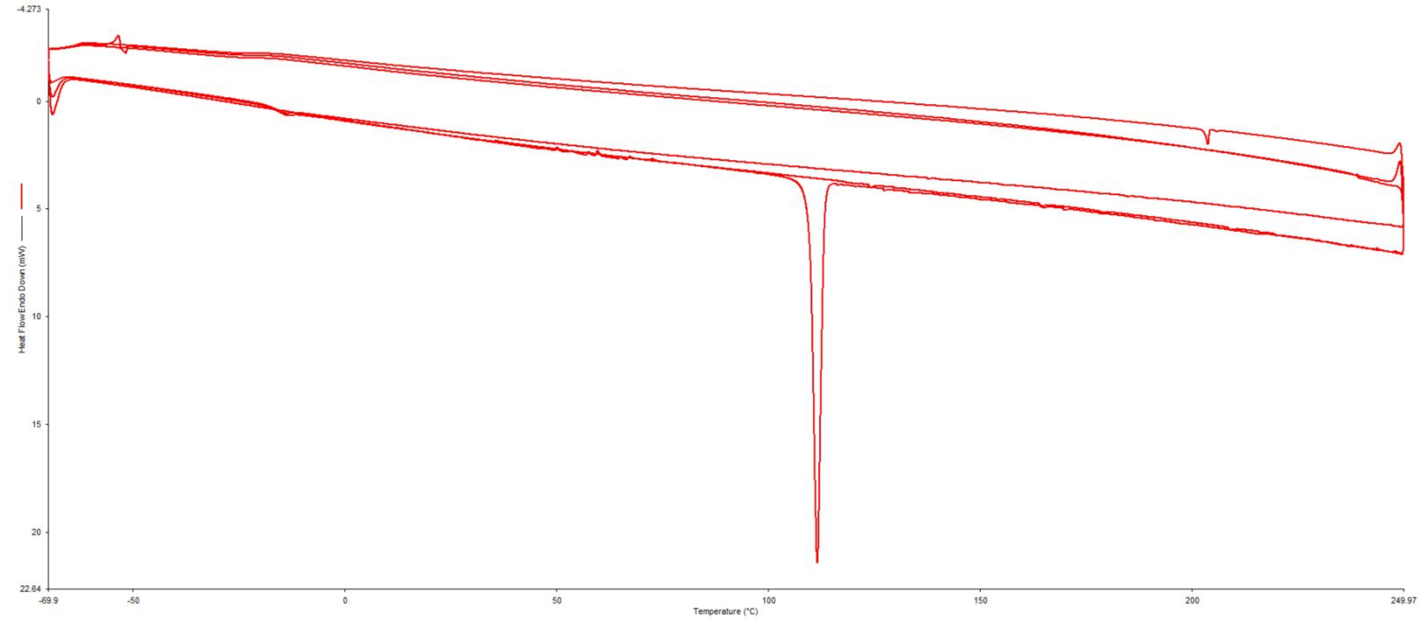

Figure S52. DSC trace of 4Me-NTf_2

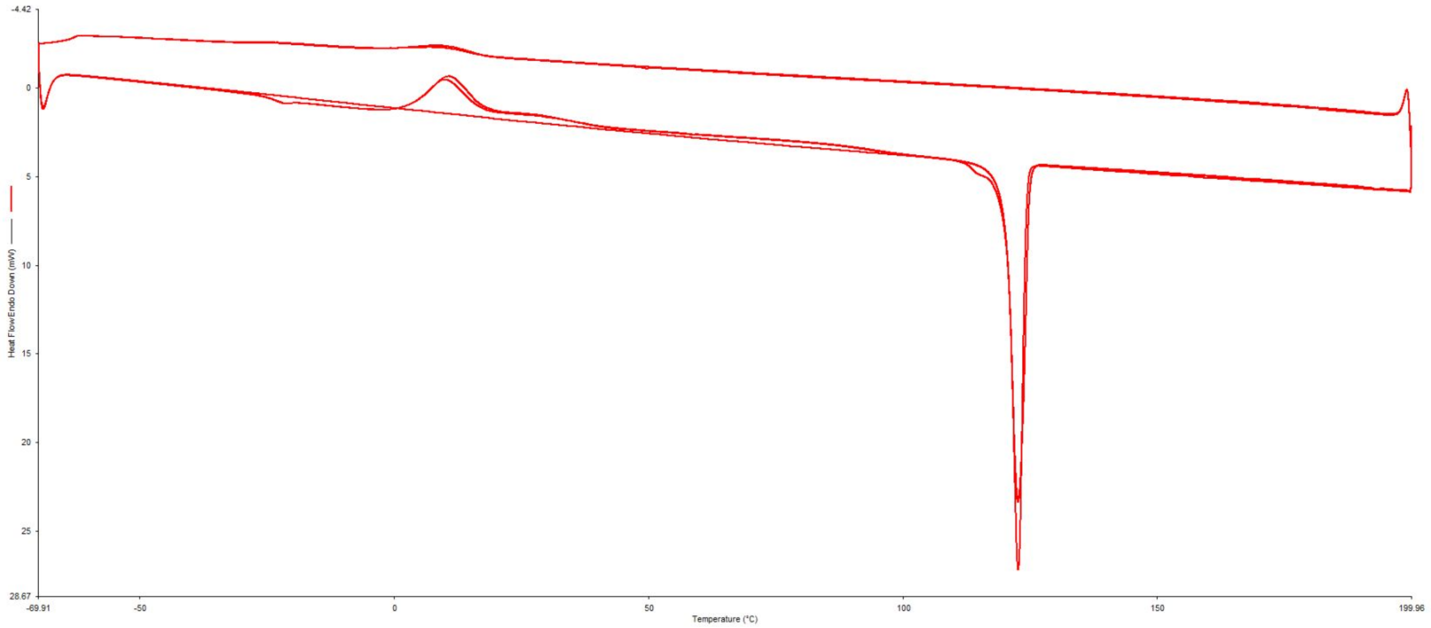

Figure S53. DSC trace of 4F-NTf_2

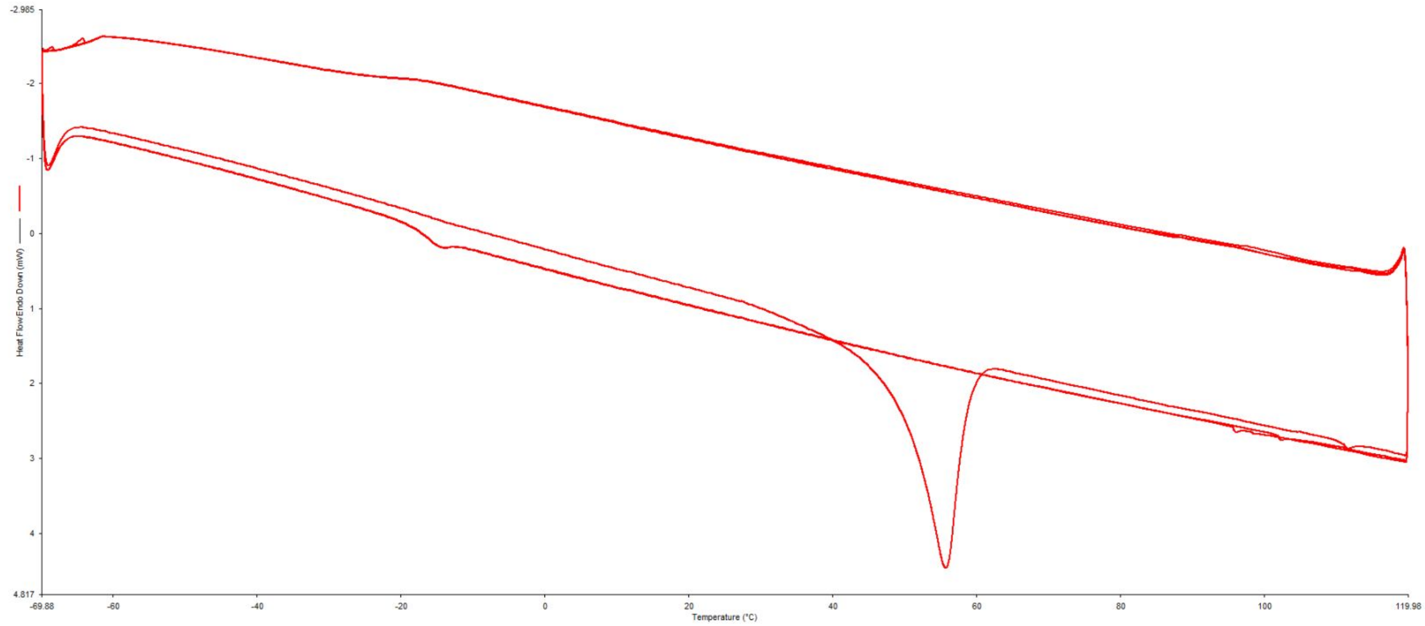

Figure S54. DSC trace of 4MeO-NTf_2

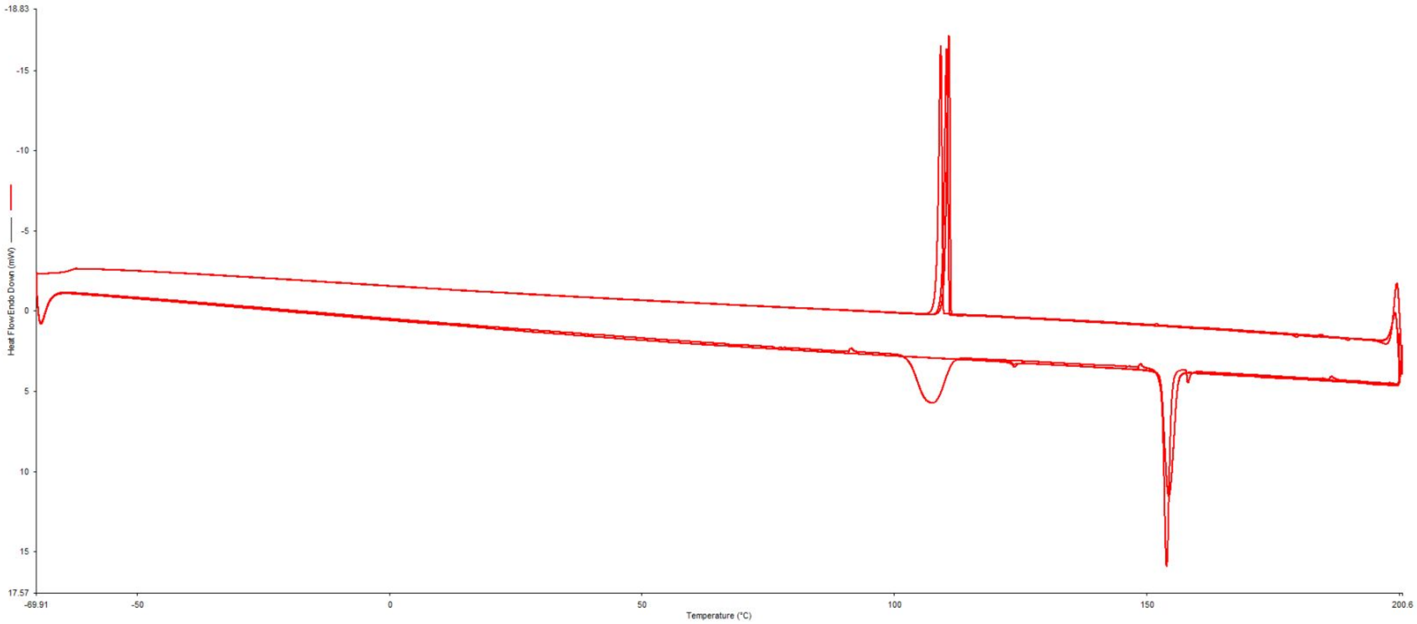

Figure S55. DSC trace of CF_3-NTf_2

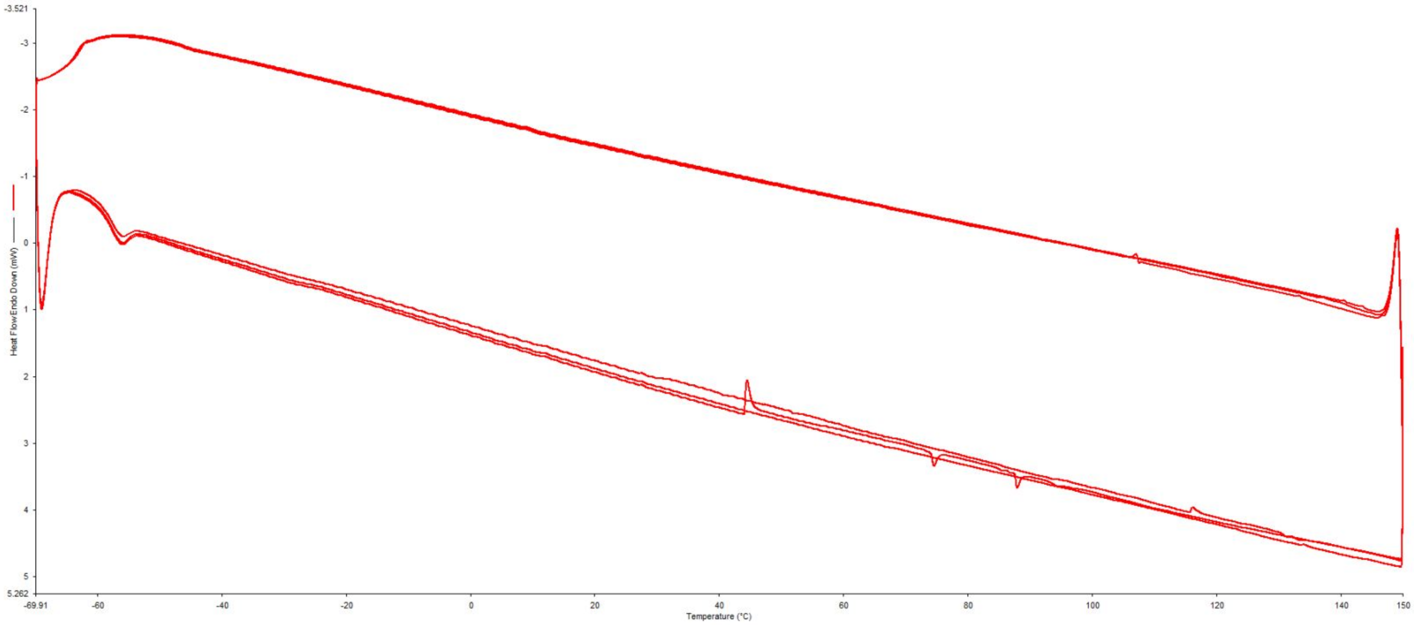

Figure S56. DSC trace of TFP-NTf_2

Computational Output Files

SPARTAN'20
build 1.1.4 (Dec 1 2021)
Wavefunction Developers:
B.J. Deppmeier, A.J. Driessen, W.J. Hehre, T.S. Hehre,
J.A. Johnson, W.S. Ohlinger, P.E. Klunzinger
Please cite Spartan as:
Spartan'20
Wavefunction Inc.
Irvine CA
QChem
5.1, QChem,
Inc., Pleasanton, CA (2020)
QChem
Developers:
Yihan Shao, Zhengting Gan, E. Epifanovsky, A. T. B. Gilbert, M. Wormit,
J. Kussmann, A. W. Lange, A. Behn, Jia Deng, Xintian Feng, D. Ghosh,
M. Goldey, P. R. Horn, L. D. Jacobson, I. Kaliman, T. Kus, A. Landau, Jie Liu,
E. I. Proynov, R. M. Richard, R. P. Steele, E. J. Sundstrom,
H. L. Woodcock III, P. M. Zimmerman, D. Zuev, B. Alam, B. Albrecht,
E. Alguire, S. A. Baeppler, D. Barton, Z. Benda, Y. A. Bernard,
E. J. Berquist, K. B. Bravaya, H. Burton, K. CarterFenk,
D. Casanova,
ChunMin
Chang, Yunqing Chen, A. Chien, K. D. Closser, M. P. Coons,
S. Coriani, S. Dasgupta, A. L. Dempwolff, M. Diedenhofen, Hainam Do,
R. G. Edgar, PoTung
Fang, S. Faraji, S. Fatehi, Qingguo Feng, J. FossoTande,
J. Gayvert, Qinghui Ge, A. Ghysels, G. Gidofalvi, J. Gomes, J. Gonthier,
A. Gunina, D. Hait, M. W. D. HansonHeine,
P. H. P. Harbach, A. W. Hauser,
M. F. Herbst, J. E. Herr, E. G. Hohenstein, Z. C. Holden, Kerwin Hui,
B. C. Huynh, T.C.
Jagau, Hyunjun Ji, B. Kaduk, K. Khistyaev, Jaehoon Kim,
P. Klunzinger, K. Koh, D. Kosenkov, L. Koulias, T. Kowalczyk, C. M. Krauter,
A. Kunitsa, Ka Un Lao, A. Laurent, K. V. Lawler, Joonho Lee, D. Lefrancois,
S. Lehtola, D. S. Levine, YiPei
Li, YouSheng
Lin, Fenglai Liu, KuanYu
Liu,
E. Livshits, M. Loipersberger, A. Luenser, P. Manohar, E. Mansoor,
S. F. Manzer, ShanPing
Mao, Yuezhi Mao, N. Mardirossian, A. V. Marenich,
T. Markovich, L. A. MartinezMartinez,
S. A. Maurer, N. J. Mayhall,
S. C. McKenzie, J.M.
Mewes, P. Morgante, A. F. Morrison, J. W. Mullinax,
K. Nanda, T. S. NguyenBeck,
R. OlivaresAmaya,
J. A. Parkhill, S. K. Paul,
Zheng Pei, T. M. Perrine, F. Plasser, P. Pokhilko, S. Prager, A. Prociuk,
E. Ramos, B. Rana, D. R. Rehn, F. Rob, M. Scheurer, M. Schneider, N. Sergueev,
S. M. Sharada, S. Sharma, D. W. Small, T. Stauch, C. J. Stein, T. Stein,
YuChuan
Su, S. P. Veccham, A. J. W. Thom, A. Tkatchenko, T. Tsuchimochi,
N. M. Tubman, L. Vogt, M. L. Vidal, O. Vydrov, M. A. Watson, J. Wenzel,
M. de Wergifosse, T. A. Wesolowski, A. White, J. Witte, A. Yamada, Jun Yang,
K. Yao, S. Yeganeh, S. R. Yost, ZhiQiang
You, A. Zech, Igor Ying Zhang,

```
Xing Zhang, Yan Zhao, Ying Zhu, B. R. Brooks, G. K. L. Chan, C. J. Cramer,
M. S. Gordon, W. J. Hehre, A. Klamt, M. W. Schmidt, C. D. Sherrill,
D. G. Truhlar, A. AspuruGuzik,
R. Baer, A. T. Bell, N. A. Besley,
JengDa
Chai, A. E. DePrince, III, R. A. DiStasio Jr., A. Dreuw,
B. D. Dunietz, T. R. Furlani, ChaoPing
Hsu, Yousung Jung, Jing Kong,
D. S. Lambrecht, WanZhen Liang, C. Ochsenfeld, V. A. Rassolov,
L. V. Slipchenko, J. E. Subotnik, T. Van Voorhis, J. M. Herbert, A. I. Krylov,
P. M. W. Gill, M. HeadGordon,
Contributors to earlier versions of QChem
not listed above:
R. D. Adamson, B. Austin, J. Baker, G. J. O. Beran, K. Brandhorst,
S. T. Brown, E. F. C. Byrd, A. K. Chakraborty, C.L.
Cheng, Siu Hung Chien,
D. M. Chipman, D. L. Crittenden, H. Dachsel, R. J. Doerksen, A. D. Dutoi,
L. FustiMolnar,
W. A. Goddard III, A. GolubevaZadorozhnaya,
S. R. Gwaltney,
G. Hawkins, A. Heyden, S. Hirata, G. Kedziora, F. J. Keil, C. Kelley,
Jihan Kim, R. A. King, R. Z. Khaliullin, P. P. Korambath, W. Kurlancheek,
A. M. Lee, M. S. Lee, S. V. Levchenko, Ching Yeh Lin, D. Liotard,
R. C. Lochan, I. Lotan, P. E. Maslen, N. Nair, D. P. O'Neill, D. Neuhauser,
E. Neuscamman, C. M. Oana, R. Olson, B. Peters, R. Peverati, P. A. Pieniazek,
Y. M. Rhee, J. Ritchie, M. A. Rohrdanz, E. Rosta, N. J. Russ,
H. F. Schaefer III, N. E. Schultz, N. Shenvi, A. C. Simmonett, A. Sodt,
D. Stuck, K. S. Thanthiriwatte, V. Vanovschi, Tao Wang, A. Warshel,
C. F. Williams, Q. Wu, X. Xu, W. Zhang,
Please cite QChem
as follows :
Y.Shao et al., Mol.Phys. 113, 184 215
(2015)
DOI : 10.1080/00268976.2014.952696
Parts of QChem
use Armadillo 8.300.2 (tropical Shenanigans).
http://arma.sourceforge.net/
Wavefunction Inc. Sales: sales@wavefun.com
Irvine CA Support: support@wavefun.com
Web: www.wavefun.com
Copyright © 1995 2021
Wavefunction
Version of QChem
Parts of QChem
use Armadillo 8.300.2 (Tropical Shenanigans).
http://arma.sourceforge.net/
QChem
begins on Fri Jan 13 07:08:22 2023
Scratch files written to
C:/Users/PATRIC~1/AppData/Local/Temp/WF044ED6B01B1F207C//scratch///
Processing $rem in system registry
... MEM_TOTAL 20000 # MB
Processing $rem in C:/Program
Files/Wavefunction/Spartan20v114/P4e//../auxdir/config/preferences:
(site specific preferences)
... THRESH 9
... SMALL_PROD_XCMAT 9
... BASIS_LIN_DEP_THRESH 5
```

```
... SCF_ALGORITHM DIIS_GDM
... MAXSCF 250
... MAXDIIS 45
... THRESHDIIS 1
(i.e. don't switch on deltaE)
... ECP_FIT TRUE (Convert deprecated ECP files)
... GUI GUI_SPARTAN
... TERSE_OUTPUT TRUE !turn on spartan printing
... SCF_CONVERGENCE 7
... CCMAN2 FALSE (qc4.3)
... SYMMETRY FALSE ! turn of symmetry for spartan16
... SYM_IGNORE TRUE ! ..use FORCESYMMETRY to override
... GEOM_OPT_TOL_GRADIENT 700 ! loosen tolernaces for organic geometries
... GEOM_OPT_TOL_DISPLACEMENT 1400 ! was 1200 = .0012
... GEOM_OPT_TOL_ENERGY 2000 ! was 100 = .000 001
... GEN_SCFMAN FALSE
Processing $rem in input file
... JOBTYPE SP
... SCF_CONVERGENCE 7 (sp default for single point energy)
... METHOD WB97MV
... xc_grid 75000302 (75,302)
... BASIS 6311+
G(2DF,2P)
... THRESH 12 #diffuse default
... MAXSCF 350 #diffuse default
... VARTHRESH 2 (default DFT)
... INCDFT TRUE (default DFT)
... GUI GUI_SPARTAN
... TERSE_OUTPUT TRUE
NAlpha2: 194
NElect 194
Mult 1
Warning: disabling incdft.
Checking the input file for inconsistencies... ...done.
User
input:
$
comment
2methyl
BuTPP
$end
$molecule
1 1
15 0.17223293079 0.098703020948
0.051262767725
6 1.230544456
0.98235880858
0.81662784027
1 0.98305162945
1.1441792253
1.8686916321
1 1.2638448151
1.9715748206
0.35033009519
6 2.5804330199
0.26591043506
0.67024112973
1 2.8512552291
```

0.19604175259
0.3872041018
1 2.5120119551
0.76859941843 1.0251216124
6 0.43678786019
0.70578922794 1.4522019323
6 0.78097575517 1.1544745435 1.2114160432
6 1.0553203791
0.028718042874
2.4847789547
6 0.0028255533457 1.4386434968 2.3406213483
1 0.94091522887
0.9333467372 2.5019510924
6 2.0095650854 1.8154012414 1.0066889942
6 0.32497224939
2.0980369764 1.5363137675
1 0.1289405861 2.656980396 0.72596070065
6 3.6817574986
0.99960950296
1.4349412472
1 3.4179758724
1.046595867
2.4979410064
1 3.7333730934
2.0370709077
1.0847479163
6 1.5108225038 1.2546590033
0.34816159793
6 1.9935132386 1.2818471424
1.6617763951
1 1.5522340349 0.64005425291
2.4167637809
6 2.0973985034 2.0565324446
0.64843802251
6 2.4081307163 2.7459898947 1.9670563516
1 3.349298762 3.2657639381 1.8262390212
6 1.6324708364 2.0788804446
2.0838029109
1 0.73919506149 2.6998766748
2.197800663
1 2.4056289101 2.5073703317
2.7214819044
1 1.4110324592 1.0797965185
2.4669587275
6 0.42517299551 2.3695286179 3.2772962045
1 0.187281921
2.5805274579 4.1449325877
6 1.6363200904 3.0224193252 3.0877186531
1 1.9812241694 3.7513656603 3.8112874865
6 0.80052806175
2.7770952361 2.6488242786
1 0.70834324422
3.8544823732 2.7052448175
6 3.0486888865 2.1104156249
2.0075877906
1 3.4158467938 2.1238705999
3.0262142619
6 2.9118308734 1.5699540215 0.17722120792

```
1 2.3598755057 1.4908600476 1.1162717844
1 3.619804595 2.3927428022 0.28009537819
1 3.4852152837 0.64746861311 0.051110040344
6 1.5148435678
0.68043404943 3.5936303702
1 1.9857409256
0.13304577452 4.4029102049
6 1.3917291177
2.0618411061 3.6815879209
1 1.7635571974
2.5787482075 4.5582204413
6 3.6324678713 2.911825269
1.0330321609
1 4.461505426 3.5612120063
1.2877661194
6 5.0408677071
0.3268295981
1.2671070788
1 5.815661127
0.86051374816
1.8194953322
1 5.0191508625
0.70275581391 1.634392113
1 5.3400237146
0.29992216782
0.21582668311
6 3.1615624463 2.8777165646
0.27257216443
1 3.6302600396 3.5004236585
1.0265692656
6 1.2898324442
1.5200253564
2.4369437628
1 2.2462466401
1.7433036305
1.952705063
1 1.3412889354
1.9250077823
3.4482311596
1 0.50670116061
2.0654497738
1.9087999017
$end
$rem
JOBTYPE SP
SCF_CONVERGENCE 7 (sp default for single point energy)
METHOD WB97MV
xc_grid 75000302 (75,302)
BASIS 6311+
G(2DF,2P)
THRESH 12 #diffuse default
MAXSCF 350 #diffuse default
VARTHRESH 2 (default DFT)
INCDFT TRUE (default DFT)
GUI GUI_SPARTAN
TERSE_OUTPUT TRUE
$end
S
```

tandard Nuclear Orientation (Angstroms)
I Atom X Y Z
1
P 0.1722329308 0.0987030209
0.0512627677
2 C 1.2305444560
0.9823588086
0.8166278403
3 H 0.9830516295
1.1441792253
1.8686916321
4 H 1.2638448151
1.9715748206
0.3503300952
5 C 2.5804330199
0.2659104351
0.6702411297
6 H 2.8512552291
0.1960417526
0.3872041018
7 H 2.5120119551
0.7685994184 1.0251216124
8 C 0.4367878602
0.7057892279 1.4522019323
9 C 0.7809757552 1.1544745435 1.2114160432
10 C 1.0553203791
0.0287180429
2.4847789547
11 C 0.0028255533 1.4386434968 2.3406213483
12 H 0.9409152289
0.9333467372 2.5019510924
13 C 2.0095650854 1.8154012414 1.0066889942
14 C 0.3249722494
2.0980369764 1.5363137675
15 H 0.1289405861 2.6569803960 0.7259607006
16 C 3.6817574986
0.9996095030
1.4349412472
17 H 3.4179758724
1.0465958670
2.4979410064
18 H 3.7333730934
2.0370709077
1.0847479163
19 C 1.5108225038 1.2546590033
0.3481615979
20 C 1.9935132386 1.2818471424
1.6617763951
21 H 1.5522340349 0.6400542529
2.4167637809
22 C 2.0973985034 2.0565324446
0.6484380225
23 C 2.4081307163 2.7459898947 1.9670563516
24 H 3.3492987620 3.2657639381 1.8262390212
25 C 1.6324708364 2.0788804446
2.0838029109
26 H 0.7391950615 2.6998766748
2.1978006630

27 H 2.4056289101 2.5073703317
2.7214819044
28 H 1.4110324592 1.0797965185
2.4669587275
29 C 0.4251729955 2.3695286179 3.2772962045
30 H 0.1872819210
2.5805274579 4.1449325877
31 C 1.6363200904 3.0224193252 3.0877186531
32 H 1.9812241694 3.7513656603 3.8112874865
33 C 0.8005280618
2.7770952361 2.6488242786
34 H 0.7083432442
3.8544823732 2.7052448175
35 C 3.0486888865 2.1104156249
2.0075877906
36 H 3.4158467938 2.1238705999
3.0262142619
37 C 2.9118308734 1.5699540215 0.1772212079
38 H 2.3598755057 1.4908600476 1.1162717844
39 H 3.6198045950 2.3927428022 0.2800953782
40 H 3.4852152837 0.6474686131 0.0511100403
41 C 1.5148435678
0.6804340494 3.5936303702
42 H 1.9857409256
0.1330457745 4.4029102049
43 C 1.3917291177
2.0618411061 3.6815879209
44 H 1.7635571974
2.5787482075 4.5582204413
45 C 3.6324678713 2.9118252690
1.0330321609
46 H 4.4615054260 3.5612120063
1.2877661194
47 C 5.0408677071
0.3268295981
1.2671070788
48 H 5.8156611270
0.8605137482
1.8194953322
49 H 5.0191508625
0.7027558139 1.6343921130
50 H 5.3400237146
0.2999221678
0.2158266831
51 C 3.1615624463 2.8777165646
0.2725721644
52 H 3.6302600396 3.5004236585
1.0265692656
53 C 1.2898324442
1.5200253564
2.4369437628
54 H 2.2462466401
1.7433036305
1.9527050630
55 H 1.3412889354
1.9250077823
3.4482311596
56 H 0.5067011606

```
2.0654497738
1.9087999017
Nuclear
Repulsion Energy = 2582.05845156 hartrees
There are 97 alpha and 97 beta electrons
Requested basis set is 6311+
G(2df,2p)
There are 365 shells and 1162 basis functions
Total QAlloc Memory Limit 20000 MB
MegaArray
Size 188 MB
MEM_STATIC part 192 MB
.. (5.2.P)
Entering
fldman on Fri Jan 13 07:08:22 2023 A
cutoff of 1.0D12
yielded 44577 shell pairs
There are 458562 function pairs ( 557501 Cartesian)
Smallest overlap matrix eigenvalue = 1.51E07
Linear dependence detected in AO basis
Tighter screening thresholds may be required for diffuse basis sets
Use S2THRESH > 12 and THRESH = 14 in case of SCF convergence issues
Number of orthogonalized atomic orbitals = 1143
Maximum deviation from orthogonality = 2.215E11
Scale SEOQF with 1.000000e02/
1.000000e01/
1.000000e01
Standard Electronic Orientation quadrupole field applied
Nucleusfield
energy = 0.0000000315
hartrees
Entering
gesman on Fri Jan 13 07:08:26 2023 Guess
from superposition of atomic densities
Warning: Energy on first SCF cycle will be nonvariational
SAD guess density has 191.164804 electrons
Entering
scfman on Fri Jan 13 07:08:26 2023 Longrange
K will be added via erf
Coulomb attenuation parameter = 0.3 bohr**(1)
A restricted hybrid HFDFT
SCF calculation will be
performed using Pulay DIIS + Geometric Direct Minimization
Exchange: 0.1500 HartreeFock
+ 1.0000 wB97MV
+ LRHF
Correlation: 1.0000 wB97MV
Using EulerMaclaurinLebedev
(75,302) quadrature formula
Nonlocal Correlation: VV10 with C = 0.0100 and b = 6.00 and scale = 1.00000
Grid used for NLC: SG1
standard quadrature
SCF converges when RMS gradient is below 1.0E07
Exchange: 0.1500 HartreeFock
+ 1.0000 wB97MV
+ LRHF
Correlation: 1.0000 wB97MV
Using EulerMaclaurinLebedev
```

```
(75,302) quadrature formula
Nonlocal Correlation: VV10 with C = 0.0100 and b = 6.00 and scale = 1.00000
Grid used for NLC: SG1
standard quadrature
using 4 threads for integral computing
OpenMP
Integral computing Module
Release: version 1.0, May 2013, QChem
Inc. Pittsburgh
using
4 threads for integral computing
OpenMP
Integral computing Module
Release: version 1.0, May 2013, QChem
Inc. Pittsburgh
OpenMP
BLAS3 based DFT computing Module
Release: version 1.0, May 2013, QChem
Inc. Pittsburgh
OpenMP
BLAS3 based DFT computing Module
Release: version 1.0, May 2013, QChem
Inc. Pittsburgh
OpenMP
BLAS3 based DFT computing Module
Release: version 1.0, May 2013, QChem
Inc. Pittsburgh
Cycle
Energy DIIS Error
1
1311.9568176740
4.39E02
2 1200.4927441423
2.70E02
3 808.4749284125
3.08E02
4 1113.9803639220
2.68E02
5 1192.0213406800
2.15E02
6 971.0860300557
3.62E02
7 1306.2444623569
5.57E03
8 1304.9324543300
6.00E03
9 1311.2761260289
1.62E03
10 1311.7078148868
5.61E04
11 1311.7584519455
2.19E04
12 1311.7655883731
9.30E05
13 1311.7672158679
2.69E05
14 1311.7673752348
7.36E06
```

15 1311.7673944159
3.41E06
16 1311.7673995921
1.56E06
17 1311.7674011965
7.08E07
18 1311.7674015779
3.30E07
19 1311.7674016493
1.17E07
20 1311.7674016543
4.67E08
Convergence criterion met
SCF
time: CPU 35811.45 s wall 9005.99 s
SCF energy in the final basis set = 1311.76740165
Total energy in the final basis set = 1311.76740165
Entering
anlman on Fri Jan 13 09:38:34 2023 Orbital
Energies (a.u.)
Alpha
MOs
Occupied
77.5705
10.4925
10.4923
10.4915
10.4913
10.4911
10.4894
10.4892
10.4809
10.4780
10.4767
10.4684
10.4679
10.4672
10.4646
10.4640
10.4635
10.4632
10.4630
10.4626
10.4622
10.4567
10.4548
10.4506
10.4429
10.4191
6.9197
5.0317
5.0317
5.0314
1.1302
1.1133
1.1123
1.0643
1.0315

1.0244
1.0217
1.0167
0.9959
0.9937
0.9729
0.9363
0.9292
0.9277
0.9047
0.8644
0.8533
0.8441
0.8343
0.8252
0.8238
0.8096
0.7906
0.7487
0.7436
0.7245
0.7123
0.7052
0.7014
0.6960
0.6790
0.6775
0.6710
0.6673
0.6596
0.6496
0.6481
0.6426
0.6355
0.6344
0.6331
0.6298
0.6272
0.6218
0.6126
0.6086
0.6031
0.5956
0.5923
0.5849
0.5828
0.5737
0.5681
0.5642
0.5634
0.5626
0.5496
0.5452
0.5429
0.5308
0.5290
0.4761
0.4726

0.4659
0.4603
0.4582
0.4572
Virtual
0.0933
0.0854
0.0808
0.0674
0.0642
0.0602
0.0576
0.0554
0.0522
0.0495
0.0412
0.0368
0.0347
0.0326
0.0295
0.0224
0.0171
0.0165
0.0135
0.0127
0.0103
0.0066
0.0032
0.0021

0.0047	0.0092	0.0122	0.0134	0.0172	0.0200	0.0228	0.0243
0.0248	0.0308	0.0352	0.0353	0.0396	0.0419	0.0425	0.0459
0.0500	0.0514	0.0561	0.0573	0.0634	0.0649	0.0664	0.0722
0.0734	0.0772	0.0793	0.0807	0.0844	0.0870	0.0891	0.0898
0.0926	0.0958	0.0973	0.0994	0.1018	0.1048	0.1103	0.1135
0.1145	0.1175	0.1217	0.1237	0.1273	0.1301	0.1312	0.1342
0.1356	0.1390	0.1399	0.1407	0.1443	0.1465	0.1480	0.1508
0.1535	0.1579	0.1609	0.1623	0.1630	0.1658	0.1683	0.1725
0.1731	0.1759	0.1796	0.1814	0.1823	0.1838	0.1855	0.1877
0.1896	0.1902	0.1923	0.1950	0.1979	0.2020	0.2044	0.2063
0.2079	0.2106	0.2140	0.2161	0.2193	0.2201	0.2233	0.2267
0.2289	0.2295	0.2326	0.2343	0.2349	0.2398	0.2427	0.2452
0.2476	0.2525	0.2531	0.2620	0.2656	0.2703	0.2728	0.2764
0.2816	0.2842	0.2883	0.2913	0.2956	0.2993	0.3012	0.3034
0.3058	0.3092	0.3130	0.3136	0.3180	0.3274	0.3314	0.3346
0.3442	0.3455	0.3572	0.3611	0.3647	0.3746	0.3800	0.3900
0.3932	0.4024	0.4080	0.4125	0.4142	0.4167	0.4209	0.4346
0.4421	0.4433	0.4526	0.4539	0.4600	0.4632	0.4660	0.4685
0.4736	0.4772	0.4793	0.4845	0.4867	0.4885	0.4909	0.4919
0.4959	0.4989	0.5020	0.5048	0.5105	0.5134	0.5167	0.5208
0.5239	0.5275	0.5304	0.5319	0.5423	0.5447	0.5455	0.5503
0.5512	0.5562	0.5600	0.5613	0.5636	0.5687	0.5705	0.5729
0.5760	0.5799	0.5807	0.5857	0.5888	0.5894	0.5926	0.5945
0.5957	0.5982	0.6003	0.6019	0.6042	0.6059	0.6074	0.6102
0.6110	0.6133	0.6164	0.6194	0.6241	0.6263	0.6309	0.6322
0.6342	0.6363	0.6379	0.6407	0.6458	0.6474	0.6498	0.6511
0.6567	0.6573	0.6603	0.6619	0.6651	0.6685	0.6731	0.6764
0.6774	0.6812	0.6821	0.6838	0.6867	0.6943	0.6967	0.6996
0.7023	0.7051	0.7077	0.7126	0.7140	0.7183	0.7222	0.7251

0.7278	0.7291	0.7319	0.7372	0.7395	0.7414	0.7477	0.7517
0.7563	0.7566	0.7632	0.7670	0.7765	0.7787	0.7801	0.7839
0.7916	0.7941	0.7972	0.8039	0.8051	0.8063	0.8172	0.8217
0.8235	0.8273	0.8333	0.8394	0.8431	0.8495	0.8525	0.8558
0.8634	0.8691	0.8730	0.8788	0.8810	0.8883	0.8941	0.9036
0.9065	0.9095	0.9136	0.9170	0.9256	0.9289	0.9356	0.9387
0.9410	0.9510	0.9551	0.9586	0.9637	0.9650	0.9723	0.9752
0.9817	0.9846	0.9884	0.9954	1.0002	1.0022	1.0065	1.0130
1.0152	1.0211	1.0328	1.0343	1.0382	1.0447	1.0496	1.0523
1.0566	1.0634	1.0658	1.0713	1.0749	1.0796	1.0844	1.0883
1.0903	1.0966	1.1000	1.1065	1.1123	1.1154	1.1182	1.1265
1.1289	1.1312	1.1337	1.1369	1.1484	1.1527	1.1535	1.1577
1.1602	1.1674	1.1700	1.1736	1.1770	1.1815	1.1883	1.1923
1.1955	1.1994	1.2025	1.2093	1.2112	1.2169	1.2207	1.2221
1.2313	1.2345	1.2356	1.2437	1.2458	1.2494	1.2520	1.2534
1.2598	1.2657	1.2667	1.2693	1.2773	1.2792	1.2829	1.2864
1.2888	1.2935	1.2977	1.3003	1.3044	1.3092	1.3097	1.3120
1.3157	1.3208	1.3220	1.3248	1.3272	1.3320	1.3384	1.3461
1.3510	1.3513	1.3581	1.3585	1.3623	1.3661	1.3701	1.3766
1.3808	1.3844	1.3893	1.3965	1.4006	1.4054	1.4100	1.4136
1.4159	1.4182	1.4279	1.4299	1.4353	1.4368	1.4422	1.4453
1.4472	1.4529	1.4538	1.4575	1.4611	1.4679	1.4712	1.4741
1.4812	1.4858	1.4864	1.4951	1.5020	1.5078	1.5110	1.5182
1.5229	1.5297	1.5345	1.5378	1.5436	1.5515	1.5575	1.5602
1.5699	1.5732	1.5841	1.5877	1.5894	1.5952	1.5963	1.6027
1.6039	1.6084	1.6174	1.6231	1.6282	1.6368	1.6413	1.6428
1.6446	1.6538	1.6568	1.6631	1.6657	1.6747	1.6789	1.6819
1.6891	1.7029	1.7148	1.7209	1.7240	1.7320	1.7375	1.7533
1.7652	1.7739	1.7966	1.8098	1.8195	1.8271	1.8414	1.8462
1.8533	1.8666	1.8713	1.8880	1.8948	1.9100	1.9203	1.9364
1.9516	1.9748	1.9849	1.9951	1.9975	2.0072	2.0153	2.0197
2.0320	2.0496	2.0866	2.1060	2.1469	2.1593	2.2235	2.2672
2.2842	2.3057	2.3139	2.3396	2.3576	2.3966	2.4012	2.4201
2.4286	2.4484	2.4616	2.4648	2.4768	2.4902	2.5013	2.5055
2.5071	2.5154	2.5335	2.5421	2.5481	2.5558	2.5723	2.5817
2.5854	2.5997	2.6035	2.6059	2.6105	2.6163	2.6301	2.6328
2.6384	2.6502	2.6558	2.6699	2.6755	2.6849	2.6913	2.6926
2.6978	2.7019	2.7038	2.7085	2.7144	2.7185	2.7216	2.7271
2.7348	2.7389	2.7404	2.7455	2.7540	2.7608	2.7640	2.7670
2.7728	2.7780	2.7829	2.7876	2.7903	2.7956	2.8014	2.8072
2.8103	2.8119	2.8148	2.8199	2.8232	2.8266	2.8306	2.8370
2.8420	2.8449	2.8496	2.8535	2.8564	2.8587	2.8614	2.8709
2.8726	2.8740	2.8803	2.8806	2.8883	2.8898	2.8938	2.8975
2.9018	2.9038	2.9132	2.9175	2.9213	2.9233	2.9310	2.9342
2.9372	2.9404	2.9435	2.9479	2.9525	2.9564	2.9668	2.9709
2.9720	2.9745	2.9790	2.9847	2.9904	2.9945	2.9974	3.0014
3.0144	3.0167	3.0221	3.0342	3.0376	3.0419	3.0495	3.0562
3.0598	3.0671	3.0706	3.0754	3.0761	3.0821	3.0869	3.0933
3.0965	3.1058	3.1082	3.1157	3.1175	3.1184	3.1273	3.1290
3.1346	3.1380	3.1417	3.1446	3.1453	3.1504	3.1595	3.1645
3.1662	3.1694	3.1753	3.1803	3.1857	3.1888	3.1946	3.2002
3.2019	3.2031	3.2097	3.2160	3.2214	3.2239	3.2262	3.2316
3.2353	3.2375	3.2445	3.2531	3.2549	3.2582	3.2698	3.2703
3.2718	3.2766	3.2830	3.2848	3.2898	3.2913	3.3033	3.3110
3.3120	3.3154	3.3163	3.3245	3.3272	3.3281	3.3310	3.3402
3.3414	3.3471	3.3504	3.3538	3.3581	3.3617	3.3665	3.3716
3.3779	3.3817	3.3848	3.3879	3.3920	3.3974	3.4057	3.4127
3.4182	3.4186	3.4202	3.4271	3.4297	3.4348	3.4407	3.4428

3.4462 3.4500 3.4517 3.4624 3.4654 3.4711 3.4733 3.4788
3.4862 3.4909 3.4916 3.4996 3.5054 3.5187 3.5228 3.5308
3.5339 3.5390 3.5446 3.5491 3.5538 3.5582 3.5596 3.5721
3.5744 3.5842 3.5864 3.5933 3.5974 3.6049 3.6093 3.6158
3.6311 3.6326 3.6418 3.6486 3.6496 3.6533 3.6589 3.6625
3.6650 3.6758 3.6810 3.6816 3.6914 3.6968 3.7027 3.7102
3.7135 3.7178 3.7270 3.7300 3.7322 3.7402 3.7484 3.7509
3.7608 3.7637 3.7717 3.7724 3.7798 3.7893 3.7904 3.7929
3.7959 3.8002 3.8034 3.8071 3.8094 3.8114 3.8170 3.8175
3.8207 3.8250 3.8299 3.8354 3.8384 3.8440 3.8466 3.8521
3.8574 3.8691 3.8794 3.8798 3.8904 3.8996 3.9068 3.9140
3.9178 3.9253 3.9293 3.9329 3.9423 3.9447 3.9472 3.9575
3.9606 3.9719 3.9789 3.9876 3.9913 3.9931 4.0007 4.0106
4.0129 4.0163 4.0254 4.0282 4.0383 4.0424 4.0503 4.0566
4.0579 4.0668 4.0683 4.0703 4.0744 4.0812 4.0918 4.0997
4.1094 4.1188 4.1285 4.1299 4.1440 4.1488 4.1524 4.1568
4.1640 4.1718 4.1766 4.1871 4.1948 4.1962 4.2023 4.2141
4.2252 4.2257 4.2278 4.2336 4.2410 4.2511 4.2558 4.2659
4.2731 4.2759 4.2812 4.2851 4.2905 4.2948 4.3000 4.3064
4.3100 4.3128 4.3175 4.3248 4.3297 4.3367 4.3389 4.3412
4.3490 4.3523 4.3557 4.3587 4.3680 4.3736 4.3791 4.3843
4.3894 4.3955 4.4019 4.4049 4.4124 4.4159 4.4195 4.4219
4.4252 4.4292 4.4368 4.4456 4.4539 4.4573 4.4603 4.4647
4.4706 4.4759 4.4795 4.4857 4.4945 4.4973 4.5063 4.5122
4.5148 4.5201 4.5259 4.5337 4.5359 4.5372 4.5383 4.5466
4.5557 4.5573 4.5715 4.5834 4.5941 4.6051 4.6091 4.6154
4.6272 4.6285 4.6327 4.6365 4.6478 4.6495 4.6586 4.6718
4.6876 4.7009 4.7022 4.7143 4.7188 4.7344 4.7386 4.7413
4.7452 4.7457 4.7692 4.7984 4.8202 4.8330 4.8597 4.8782
4.8921 4.8926 4.9033 4.9366 4.9459 4.9497 5.0461 5.0561
5.0609 5.0824 5.0919 5.0979 5.1057 5.1142 5.1199 5.1291
5.1412 5.1509 5.1550 5.2425 5.2891 5.2983 5.3110 5.3245
5.3472 5.3549 5.4113 5.4218 5.4277 5.4400 5.4572 5.4749
5.4828 5.5010 5.5400 5.5844 5.6169 5.6302 5.6363 5.6489
5.6567 5.6589 5.6615 5.6659 5.6800 5.6876 5.6963 5.6999
5.7132 5.7161 5.7193 5.7467 5.7574 5.7615 5.7748 5.7969
5.8230 5.8281 6.0767 6.0784 6.0834 6.4696 6.4718 6.4754
8.1301 14.7476 14.7695 14.8586 24.1364 24.1427 24.1635 24.4441
24.5284 24.5972 24.6514 24.6612 24.6723 24.6777 24.6828 24.6901
24.7082 24.7188 24.7265 24.7294 24.9126 24.9169 24.9208 24.9360
24.9384 24.9413 25.3878 25.3936 25.3955 165.2459
GroundState
Mulliken Net Atomic Charges
Atom Charge (a.u.)
1
P 0.280148
2 C 0.370286
3 H 0.256089
4 H 0.251788
5 C 0.406237
6 H 0.231530
7 H 0.214665
8 C 0.312553
9 C 0.285415
10 C 0.180832
11 C 0.283585
12 H 0.233932
13 C 0.153973

14 C 0.350765
15 H 0.245200
16 C 0.304586
17 H 0.174091
18 H 0.176514
19 C 0.311391
20 C 0.257300
21 H 0.266862
22 C 0.222126
23 C 0.232900
24 H 0.192457
25 C 0.697826
26 H 0.215361
27 H 0.188149
28 H 0.220149
29 C 0.176504
30 H 0.191274
31 C 0.190335
32 H 0.192115
33 C 0.204338
34 H 0.191562
35 C 0.150573
36 H 0.189925
37 C 0.681287
38 H 0.207267
39 H 0.188223
40 H 0.249226
41 C 0.171315
42 H 0.192652
43 C 0.232411
44 H 0.192627
45 C 0.299788
46 H 0.192639
47 C 0.579126
48 H 0.169039
49 H 0.159347
50 H 0.160273
51 C 0.133662
52 H 0.192915
53 C 0.633318
54 H 0.232393
55 H 0.190988
56 H 0.210748
Sum
of atomic charges = 1.000000
Cartesian
Multipole Moments
Charge
(ESU x 10^10)
4.8032
Dipole Moment (Debye)
X 1.5510 Y 0.1228 Z 0.2698
Tot 1.5791
Quadrupole Moments (DebyeAng)
XX 132.0852
XY 2.3852
YY 135.5054
XZ 3.7096

YZ 3.3589
ZZ 128.3935
Traceless Quadrupole Moments (DebyeAng)
QXX 0.2715
QYY 10.5321
QZZ 10.8036
QXY 7.1557
QXZ 11.1287
QYZ 10.0768
Octopole Moments (DebyeAng^
2)
XXX 76.1654
XXY 51.2765
XYY 19.7799
YYY 19.1320
XXZ 11.3052
XYZ 27.5199
YYZ 22.8942
XZZ 52.5582
YZZ 24.6131
ZZZ 6.4727
Traceless Octopole Moments (DebyeAng^
2)
XXX 161.9883
YYY 125.1789 ZZZ 268.9584
XXY 631.7616
XXZ 47.5611
XYY 623.5298
XYZ 412.7983
XZZ 461.5415
YYZ 221.3973
YZZ 506.5826
Hexadecapole Moments (DebyeAng^
3)
XXXX 4625.5348
XXXY 142.3942 XXYY 1142.7759
XYYY 166.3042 YYYY 3183.8749
XXXZ 242.8660
XXYZ 0.3761 XYYZ 39.2870
YYYZ 186.0648
XXZZ 1346.1183
XYZZ 27.9290 YYZZ 968.6969
XZZZ 18.4994 YZZZ 115.7416 ZZZZ 3460.5746
Traceless Hexadecapole Moments (DebyeAng^
3)
XXXX 9049.0418
XXXY 196.8393
XXXZ 15507.4035
XXYY 11599.6786 XXYZ 4482.1208
XXZZ 2550.6368
XYYY 2313.7068 XYYZ 7456.3099
XYZZ 2116.8675
XZZZ 8051.0937
YYYY 21392.0656
YYYZ 5971.9650
YYZZ 9792.3870 YZZZ 1489.8442
ZZZZ 7241.7502
Total

```
job time: 9013.58s(wall), 35821.34s(cpu)
Fri Jan 13 09:38:36 2023
```

SPARTAN'20
build 1.1.4 (Dec 1 2021)
Wavefunction Developers:
B.J. Deppmeier, A.J. Driessen, W.J. Hehre, T.S. Hehre,
J.A. Johnson, W.S. Ohlinger, P.E. Klunzinger
Please cite Spartan as:
Spartan'20
Wavefunction Inc.
Irvine CA
QChem
5.1, QChem,
Inc., Pleasanton, CA (2020)
QChem
Developers:
Yihan Shao, Zhengting Gan, E. Epifanovsky, A. T. B. Gilbert, M. Wormit,
J. Kussmann, A. W. Lange, A. Behn, Jia Deng, Xintian Feng, D. Ghosh,
M. Goldey, P. R. Horn, L. D. Jacobson, I. Kaliman, T. Kus, A. Landau, Jie Liu,
E. I. Proynov, R. M. Richard, R. P. Steele, E. J. Sundstrom,
H. L. Woodcock III, P. M. Zimmerman, D. Zuev, B. Alam, B. Albrecht,
E. Alguire, S. A. Baeppler, D. Barton, Z. Benda, Y. A. Bernard,
E. J. Berquist, K. B. Bravaya, H. Burton, K. CarterFenk,
D. Casanova,
ChunMin
Chang, Yunqing Chen, A. Chien, K. D. Closser, M. P. Coons,
S. Coriani, S. Dasgupta, A. L. Dempwolff, M. Diedenhofen, Hainam Do,
R. G. Edgar, PoTung
Fang, S. Faraji, S. Fatehi, Qingguo Feng, J. FossoTande,
J. Gayvert, Qinghui Ge, A. Ghysels, G. Gidofalvi, J. Gomes, J. Gonthier,
A. Gunina, D. Hait, M. W. D. HansonHeine,
P. H. P. Harbach, A. W. Hauser,
M. F. Herbst, J. E. Herr, E. G. Hohenstein, Z. C. Holden, Kerwin Hui,
B. C. Huynh, T.C.
Jagau, Hyunjun Ji, B. Kaduk, K. Khistyaev, Jaehoon Kim,
P. Klunzinger, K. Koh, D. Kosenkov, L. Koulias, T. Kowalczyk, C. M. Krauter,
A. Kunitsa, Ka Un Lao, A. Laurent, K. V. Lawler, Joonho Lee, D. Lefrancois,
S. Lehtola, D. S. Levine, YiPei
Li, YouSheng
Lin, Fenglai Liu, KuanYu
Liu,
E. Livshits, M. Loipersberger, A. Luenser, P. Manohar, E. Mansoor,
S. F. Manzer, ShanPing
Mao, Yuezhi Mao, N. Mardirossian, A. V. Marenich,
T. Markovich, L. A. MartinezMartinez,
S. A. Maurer, N. J. Mayhall,
S. C. McKenzie, J.M.
Mewes, P. Morgante, A. F. Morrison, J. W. Mullinax,
K. Nanda, T. S. NguyenBeck,
R. OlivaresAmaya,
J. A. Parkhill, S. K. Paul,
Zheng Pei, T. M. Perrine, F. Plasser, P. Pokhilko, S. Prager, A. Prociuk,
E. Ramos, B. Rana, D. R. Rehn, F. Rob, M. Scheurer, M. Schneider, N. Sergueev,
S. M. Sharada, S. Sharma, D. W. Small, T. Stauch, C. J. Stein, T. Stein,
YuChuan
Su, S. P. Veccham, A. J. W. Thom, A. Tkatchenko, T. Tsuchimochi,
N. M. Tubman, L. Vogt, M. L. Vidal, O. Vydrov, M. A. Watson, J. Wenzel,
M. de Wergifosse, T. A. Wesolowski, A. White, J. Witte, A. Yamada, Jun Yang,
K. Yao, S. Yeganeh, S. R. Yost, ZhiQiang
You, A. Zech, Igor Ying Zhang,

```
Xing Zhang, Yan Zhao, Ying Zhu, B. R. Brooks, G. K. L. Chan, C. J. Cramer,
M. S. Gordon, W. J. Hehre, A. Klamt, M. W. Schmidt, C. D. Sherrill,
D. G. Truhlar, A. AspuruGuzik,
R. Baer, A. T. Bell, N. A. Besley,
JengDa
Chai, A. E. DePrince, III, R. A. DiStasio Jr., A. Dreuw,
B. D. Dunietz, T. R. Furlani, ChaoPing
Hsu, Yousung Jung, Jing Kong,
D. S. Lambrecht, WanZhen Liang, C. Ochsenfeld, V. A. Rassolov,
L. V. Slipchenko, J. E. Subotnik, T. Van Voorhis, J. M. Herbert, A. I. Krylov,
P. M. W. Gill, M. HeadGordon,
Contributors to earlier versions of QChem
not listed above:
R. D. Adamson, B. Austin, J. Baker, G. J. O. Beran, K. Brandhorst,
S. T. Brown, E. F. C. Byrd, A. K. Chakraborty, C.L.
Cheng, Siu Hung Chien,
D. M. Chipman, D. L. Crittenden, H. Dachsel, R. J. Doerksen, A. D. Dutoi,
L. FustiMolnar,
W. A. Goddard III, A. GolubevaZadorozhnaya,
S. R. Gwaltney,
G. Hawkins, A. Heyden, S. Hirata, G. Kedziora, F. J. Keil, C. Kelley,
Jihan Kim, R. A. King, R. Z. Khaliullin, P. P. Korambath, W. Kurlancheek,
A. M. Lee, M. S. Lee, S. V. Levchenko, Ching Yeh Lin, D. Liotard,
R. C. Lochan, I. Lotan, P. E. Maslen, N. Nair, D. P. O'Neill, D. Neuhauser,
E. Neuscamman, C. M. Oana, R. Olson, B. Peters, R. Peverati, P. A. Pieniazek,
Y. M. Rhee, J. Ritchie, M. A. Rohrdanz, E. Rosta, N. J. Russ,
H. F. Schaefer III, N. E. Schultz, N. Shenvi, A. C. Simmonett, A. Sodt,
D. Stuck, K. S. Thanthiriwatte, V. Vanovschi, Tao Wang, A. Warshel,
C. F. Williams, Q. Wu, X. Xu, W. Zhang,
Please cite QChem
as follows :
Y.Shao et al., Mol.Phys. 113, 184 215
(2015)
DOI : 10.1080/00268976.2014.952696
Parts of QChem
use Armadillo 8.300.2 (tropical Shenanigans).
http://arma.sourceforge.net/
Wavefunction Inc. Sales: sales@wavefun.com
Irvine CA Support: support@wavefun.com
Web: www.wavefun.com
Copyright © 1995 2021
Wavefunction
Version of QChem
Parts of QChem
use Armadillo 8.300.2 (Tropical Shenanigans).
http://arma.sourceforge.net/
QChem
begins on Fri Jan 13 18:23:45 2023
Scratch files written to
C:/Users/PATRIC~1/AppData/Local/Temp/WFBFAC19B3A50C4C83//scratch///
Processing $rem in system registry
... MEM_TOTAL 20000 # MB
Processing $rem in C:/Program
Files/Wavefunction/Spartan20v114/P4e//../auxdir/config/preferences:
(site specific preferences)
... THRESH 9
... SMALL_PROD_XCMAT 9
... BASIS_LIN_DEP_THRESH 5
```

```
... SCF_ALGORITHM DIIS_GDM
... MAXSCF 250
... MAXDIIS 45
... THRESHDIIS 1
(i.e. don't switch on deltaE)
... ECP_FIT TRUE (Convert deprecated ECP files)
... GUI GUI_SPARTAN
... TERSE_OUTPUT TRUE !turn on spartan printing
... SCF_CONVERGENCE 7
... CCMAN2 FALSE (qc4.3)
... SYMMETRY FALSE ! turn of symmetry for spartan16
... SYM_IGNORE TRUE ! ..use FORCESYMMETRY to override
... GEOM_OPT_TOL_GRADIENT 700 ! loosen tolernaces for organic geometries
... GEOM_OPT_TOL_DISPLACEMENT 1400 ! was 1200 = .0012
... GEOM_OPT_TOL_ENERGY 2000 ! was 100 = .000 001
... GEN_SCFMAN FALSE
Processing $rem in input file
... JOBTYPE SP
... SCF_CONVERGENCE 7 (sp default for single point energy)
... METHOD WB97MV
... xc_grid 75000302 (75,302)
... BASIS 6311+
G(2DF,2P)
... THRESH 12 #diffuse default
... MAXSCF 350 #diffuse default
... VARTHRESH 2 (default DFT)
... INCDFT TRUE (default DFT)
... GUI GUI_SPARTAN
... TERSE_OUTPUT TRUE
NAlpha2: 194
NElect 194
Mult 1
Warning: disabling incdft.
Checking the input file for inconsistencies... ...done.
User
input:
$
comment
3methyl
BuTPP Cation
$end
$molecule
1 1
15 0.24262936961
0.022991294846
0.35764651197
6 1.3766126811 0.73090371133 0.67479225168
1 1.654875531 0.43556531868 1.6923722068
1 1.2543122648 1.8188655472 0.67633435003
6 0.85499465612
0.50490781662 1.2511040943
6 0.06607711206
1.8127968057
0.37448741865
6 1.3993433892
0.46858627904 1.6465534121
6 1.0397164099
1.401321697 2.6215853197
```

1 0.044920711694
1.833581494 2.6358880606
6 0.38607711557 2.475759216
0.77335682983
1 0.54837048221 1.9236490172
1.6942980243
6 2.454056337 0.29568558907 0.32570753011
1 2.1232404929 0.50023071226 1.3507014477
1 2.6041868647 0.78640268477
0.25144569973
6 0.4752916214
1.7511093971 1.7595212713
1 0.23982115361 2.3710507332 1.2276698688
6 0.62252134385 3.8463074464
0.75275317708
6 1.0027734793
2.2222661903 2.9580764148
6 1.9531718345
1.7973232389 3.5975758071
6 0.40100560712 4.536332664
0.44389417505
1 0.57791493045 5.6064924541
0.47619783709
6 1.925412047
1.4179969049 3.6332133682
1 2.3468368921
1.7691387107 4.5694124308
6 3.2310159198
1.2359942505 3.5704726027
1 3.9547779933
1.5327989365 4.3225236537
6 2.3112027035
0.182277141 3.1321467831
1 3.0272603256
0.42274556312
3.6750170814
6 3.5950291886
0.30739545935 2.6020356312
1 4.5930044835
0.11394648454
2.603789804
6 0.041743668207
3.883920456
1.585800108
1 0.20756675425
4.442240223
2.4990883013
6 0.27929372662
2.5136355356
1.5612015824
1 0.63321996681
2.0051923336
2.4511831467
6 2.6827022246
0.081297601663
1.6312317731
1 2.9684234835
0.80721904029

0.87708903452
6 1.7786057385
0.28511440917
1.9360275483
1 2.0726532357
1.2565990249
1.5540012798
6 1.1026505026 4.5731722057
1.9807923209
1 2.0729700838 5.0412645749
1.7994316125
1 0.4040260221 5.3646101085
2.2614501822
1 1.2064513828 3.8962132275
2.8296968948
6 0.60052942648
3.5620850184 3.5143292483
1 0.17994338635 4.027130692 2.9107893383
1 0.22676626819
3.4633457448 4.5356574248
1 1.4560271798
4.2410963699 3.5411983605
6 1.5754255138
2.7977891679 4.6574387211
1 0.57238372644
3.1938272146 4.4940300004
1 2.2740393435
3.6370777915 4.6659257948
1 1.5998071253
2.3392161749 5.6488219862
6 3.7789601533 1.0130526861 0.066438799694
1 4.0975800365 0.82111853274 0.96418852392
1 3.626941969 2.0955149984 0.14818525979
6 4.8690632917 0.56876267358 1.0369704173
1 5.8071014649 1.0905355305 0.84166948223
1 5.0607435841 0.50403733776
0.94880983651
1 4.5832188366 0.77438798655 2.0722759876
$end
$rem
JOBTYPE SP
SCF_CONVERGENCE 7 (sp default for single point energy)
METHOD WB97MV
xc_grid 75000302 (75,302)
BASIS 6311+
G(2DF,2P)
THRESH 12 #diffuse default
MAXSCF 350 #diffuse default
VARTHRESH 2 (default DFT)
INCDFT TRUE (default DFT)
GUI GUI_SPARTAN
TERSE_OUTPUT TRUE
$end
S
tandard Nuclear Orientation (Angstroms)
I Atom X Y Z
1
P 0.2426293696

0.0229912948
0.3576465120
2 C 1.3766126811 0.7309037113 0.6747922517
3 H 1.6548755310 0.4355653187 1.6923722068
4 H 1.2543122648 1.8188655472 0.6763343500
5 C 0.8549946561
0.5049078166 1.2511040943
6 C 0.0660771121
1.8127968057
0.3744874187
7 C 1.3993433892
0.4685862790 1.6465534121
8 C 1.0397164099
1.4013216970 2.6215853197
9 H 0.0449207117
1.8335814940 2.6358880606
10 C 0.3860771156 2.4757592160
0.7733568298
11 H 0.5483704822 1.9236490172
1.6942980243
12 C 2.4540563370 0.2956855891 0.3257075301
13 H 2.1232404929 0.5002307123 1.3507014477
14 H 2.6041868647 0.7864026848
0.2514456997
15 C 0.4752916214
1.7511093971 1.7595212713
16 H 0.2398211536 2.3710507332 1.2276698688
17 C 0.6225213439 3.8463074464
0.7527531771
18 C 1.0027734793
2.2222661903 2.9580764148
19 C 1.9531718345
1.7973232389 3.5975758071
20 C 0.4010056071 4.5363326640
0.4438941751
21 H 0.5779149304 5.6064924541
0.4761978371
22 C 1.9254120470
1.4179969049 3.6332133682
23 H 2.3468368921
1.7691387107 4.5694124308
24 C 3.2310159198
1.2359942505 3.5704726027
25 H 3.9547779933
1.5327989365 4.3225236537
26 C 2.3112027035
0.1822771410 3.1321467831
27 H 3.0272603256
0.4227455631
3.6750170814
28 C 3.5950291886
0.3073954594 2.6020356312
29 H 4.5930044835
0.1139464845
2.6037898040
30 C 0.0417436682
3.8839204560
1.5858001080

31 H 0.2075667543
4.4422402230
2.4990883013
32 C 0.2792937266
2.5136355356
1.5612015824
33 H 0.6332199668
2.0051923336
2.4511831467
34 C 2.6827022246
0.0812976017
1.6312317731
35 H 2.9684234835
0.8072190403
0.8770890345
36 C 1.7786057385
0.2851144092
1.9360275483
37 H 2.0726532357
1.2565990249
1.5540012798
38 C 1.1026505026 4.5731722057
1.9807923209
39 H 2.0729700838 5.0412645749
1.7994316125
40 H 0.4040260221 5.3646101085
2.2614501822
41 H 1.2064513828 3.8962132275
2.8296968948
42 C 0.6005294265
3.5620850184 3.5143292483
43 H 0.1799433864 4.0271306920 2.9107893383
44 H 0.2267662682
3.4633457448 4.5356574248
45 H 1.4560271798
4.2410963699 3.5411983605
46 C 1.5754255138
2.7977891679 4.6574387211
47 H 0.5723837264
3.1938272146 4.4940300004
48 H 2.2740393435
3.6370777915 4.6659257948
49 H 1.5998071253
2.3392161749 5.6488219862
50 C 3.7789601533 1.0130526861 0.0664387997
51 H 4.0975800365 0.8211185327 0.9641885239
52 H 3.6269419690 2.0955149984 0.1481852598
53 C 4.8690632917 0.5687626736 1.0369704173
54 H 5.8071014649 1.0905355305 0.8416694822
55 H 5.0607435841 0.5040373378
0.9488098365
56 H 4.5832188366 0.7743879865 2.0722759876
Nuclear
Repulsion Energy = 2464.47167265 hartrees
There are 97 alpha and 97 beta electrons
Requested basis set is 6311+
G(2df,2p)
There are 365 shells and 1162 basis functions

```
Total QAlloc Memory Limit 20000 MB
MegaArray
Size 188 MB
MEM_STATIC part 192 MB
.. (5.2.P)
Entering
fldman on Fri Jan 13 18:23:45 2023 A
cutoff of 1.0D12
yielded 41141 shell pairs
There are 424642 function pairs ( 514475 Cartesian)
Smallest overlap matrix eigenvalue = 2.10E07
Linear dependence detected in AO basis
Tighter screening thresholds may be required for diffuse basis sets
Use S2THRESH > 12 and THRESH = 14 in case of SCF convergence issues
Number of orthogonalized atomic orbitals = 1142
Maximum deviation from orthogonality = 1.851E11
Scale SEOQF with 1.000000e02/
1.000000e02/
1.000000e02
Standard Electronic Orientation quadrupole field applied
Nucleusfield
energy = 0.0000000009
hartrees
Entering
gesman on Fri Jan 13 18:23:49 2023 Guess
from superposition of atomic densities
Warning: Energy on first SCF cycle will be nonvariational
SAD guess density has 191.164804 electrons
Entering
scfman on Fri Jan 13 18:23:49 2023 Longrange
K will be added via erf
Coulomb attenuation parameter = 0.3 bohr**(1)
A restricted hybrid HFDFT
SCF calculation will be
performed using Pulay DIIS + Geometric Direct Minimization
Exchange: 0.1500 HartreeFock
+ 1.0000 wB97MV
+ LRHF
Correlation: 1.0000 wB97MV
Using EulerMaclaurinLebedev
(75,302) quadrature formula
Nonlocal Correlation: VV10 with C = 0.0100 and b = 6.00 and scale = 1.00000
Grid used for NLC: SG1
standard quadrature
SCF converges when RMS gradient is below 1.0E07
Exchange: 0.1500 HartreeFock
+ 1.0000 wB97MV
+ LRHF
Correlation: 1.0000 wB97MV
Using EulerMaclaurinLebedev
(75,302) quadrature formula
Nonlocal Correlation: VV10 with C = 0.0100 and b = 6.00 and scale = 1.00000
Grid used for NLC: SG1
standard quadrature
using 4 threads for integral computing
OpenMP
Integral computing Module
Release: version 1.0, May 2013, QChem
```

Inc. Pittsburgh
using
4 threads for integral computing
OpenMP
Integral computing Module
Release: version 1.0, May 2013, QChem
Inc. Pittsburgh
OpenMP
BLAS3 based DFT computing Module
Release: version 1.0, May 2013, QChem
Inc. Pittsburgh
OpenMP
BLAS3 based DFT computing Module
Release: version 1.0, May 2013, QChem
Inc. Pittsburgh
OpenMP
BLAS3 based DFT computing Module
Release: version 1.0, May 2013, QChem
Inc. Pittsburgh
Cycle
Energy DIIS Error
1
1311.8565882340
4.38E02
2 1195.3584256486
2.75E02
3 858.1553037055
2.76E02
4 1001.4798571182
3.68E02
5 1134.4765368427
2.74E02
6 1175.5966459845
2.42E02
7 1217.2035451831
2.04E02
8 1257.8023036156
1.45E02
9 1184.2449396469
1.88E02
10 1231.4394665778
1.40E02
11 1201.2942899901
1.67E02
12 1304.4583927532
4.39E03
13 1243.7829130075
1.38E02
14 1311.3531352601
1.51E03
15 1311.6982705573
5.72E04
16 1311.7499857997
3.12E04
17 1311.7680695871
1.11E04
18 1311.7705690556
5.13E05

19 1311.7710676557
1.63E05
20 1311.7711320129
6.24E06
21 1311.7711395002
1.79E06
22 1311.7711404778
7.30E07
23 1311.7711407727
3.45E07
24 1311.7711408605
1.42E07
25 1311.7711408760
5.56E08
Convergence criterion met
SCF
time: CPU 41107.27 s wall 10344.29 s
SCF energy in the final basis set = 1311.77114088
Total energy in the final basis set = 1311.77114088
Entering
anlman on Fri Jan 13 21:16:15 2023 Orbital
Energies (a.u.)
Alpha
MOs
Occupied
77.5666
10.4900
10.4891
10.4880
10.4880
10.4753
10.4751
10.4749
10.4746
10.4744
10.4739
10.4739
10.4735
10.4730
10.4645
10.4635
10.4633
10.4628
10.4626
10.4622
10.4617
10.4409
10.4369
10.4362
10.4358
10.4177
6.9164
5.0285
5.0283
5.0282
1.1260
1.1101
1.1092

1.0657
1.0328
1.0165
1.0133
1.0075
0.9943
0.9929
0.9709
0.9239
0.9201
0.9163
0.9083
0.8667
0.8466
0.8406
0.8289
0.8232
0.8201
0.8062
0.7793
0.7471
0.7423
0.7328
0.7095
0.7004
0.6980
0.6913
0.6742
0.6706
0.6641
0.6595
0.6519
0.6412
0.6375
0.6357
0.6321
0.6305
0.6284
0.6279
0.6244
0.6218
0.5992
0.5921
0.5918
0.5909
0.5866
0.5818
0.5813
0.5730
0.5672
0.5590
0.5573
0.5560
0.5463
0.5434
0.5413
0.5294
0.5268

0.4773
0.4692
0.4645
0.4571
0.4556
0.4471
Virtual
0.0961
0.0861
0.0733
0.0675
0.0631
0.0613
0.0561
0.0507
0.0492
0.0438
0.0374
0.0344
0.0322
0.0309
0.0264
0.0222
0.0179
0.0156
0.0137
0.0108
0.0105
0.0059
0.0027 0.0037
0.0049 0.0101 0.0110 0.0150 0.0160 0.0190 0.0199 0.0239
0.0255 0.0291 0.0298 0.0329 0.0380 0.0392 0.0401 0.0426
0.0455 0.0503 0.0532 0.0590 0.0613 0.0615 0.0619 0.0654
0.0687 0.0724 0.0762 0.0790 0.0817 0.0832 0.0871 0.0889
0.0910 0.0974 0.0991 0.1005 0.1057 0.1071 0.1083 0.1109
0.1124 0.1148 0.1190 0.1201 0.1240 0.1263 0.1284 0.1315
0.1323 0.1355 0.1383 0.1415 0.1446 0.1458 0.1488 0.1518
0.1533 0.1549 0.1560 0.1597 0.1639 0.1657 0.1689 0.1714
0.1743 0.1762 0.1792 0.1817 0.1831 0.1857 0.1879 0.1888
0.1918 0.1941 0.1955 0.1978 0.1999 0.2025 0.2061 0.2084
0.2087 0.2118 0.2132 0.2136 0.2177 0.2195 0.2212 0.2256
0.2298 0.2312 0.2333 0.2338 0.2384 0.2409 0.2445 0.2482
0.2492 0.2502 0.2539 0.2556 0.2636 0.2669 0.2710 0.2745
0.2754 0.2791 0.2816 0.2858 0.2890 0.2953 0.2996 0.3020
0.3087 0.3111 0.3149 0.3203 0.3213 0.3271 0.3350 0.3502
0.3557 0.3648 0.3705 0.3795 0.3803 0.3862 0.3914 0.3983
0.4019 0.4071 0.4114 0.4139 0.4216 0.4247 0.4307 0.4334
0.4393 0.4456 0.4500 0.4543 0.4560 0.4618 0.4639 0.4696
0.4709 0.4746 0.4797 0.4809 0.4840 0.4894 0.4912 0.4964
0.4972 0.4993 0.5033 0.5052 0.5062 0.5143 0.5181 0.5195
0.5211 0.5278 0.5316 0.5332 0.5356 0.5371 0.5388 0.5436
0.5458 0.5481 0.5512 0.5559 0.5592 0.5615 0.5639 0.5701
0.5723 0.5752 0.5797 0.5821 0.5827 0.5857 0.5892 0.5900
0.5951 0.5958 0.5991 0.5994 0.6009 0.6024 0.6084 0.6095
0.6108 0.6118 0.6144 0.6180 0.6200 0.6210 0.6252 0.6285
0.6304 0.6310 0.6317 0.6340 0.6373 0.6395 0.6420 0.6443
0.6494 0.6511 0.6517 0.6552 0.6609 0.6631 0.6661 0.6679
0.6722 0.6781 0.6797 0.6842 0.6847 0.6899 0.6920 0.6989

0.7035	0.7052	0.7075	0.7093	0.7125	0.7175	0.7195	0.7230
0.7233	0.7262	0.7305	0.7328	0.7365	0.7400	0.7410	0.7432
0.7468	0.7495	0.7542	0.7603	0.7618	0.7699	0.7713	0.7810
0.7858	0.7876	0.7950	0.7963	0.7981	0.8091	0.8111	0.8162
0.8221	0.8229	0.8314	0.8331	0.8384	0.8420	0.8451	0.8468
0.8492	0.8543	0.8560	0.8638	0.8671	0.8737	0.8756	0.8786
0.8810	0.8848	0.8924	0.8985	0.9068	0.9117	0.9202	0.9227
0.9283	0.9359	0.9394	0.9407	0.9467	0.9496	0.9514	0.9636
0.9664	0.9703	0.9759	0.9817	0.9831	0.9878	0.9886	0.9931
1.0040	1.0045	1.0078	1.0117	1.0161	1.0216	1.0277	1.0305
1.0348	1.0396	1.0439	1.0472	1.0496	1.0591	1.0604	1.0689
1.0732	1.0752	1.0798	1.0886	1.0916	1.0936	1.1000	1.1036
1.1068	1.1114	1.1230	1.1270	1.1283	1.1326	1.1376	1.1393
1.1423	1.1510	1.1526	1.1557	1.1600	1.1648	1.1714	1.1737
1.1761	1.1822	1.1831	1.1886	1.1917	1.1975	1.2042	1.2055
1.2095	1.2164	1.2193	1.2278	1.2294	1.2350	1.2379	1.2402
1.2424	1.2557	1.2567	1.2627	1.2650	1.2700	1.2718	1.2734
1.2757	1.2796	1.2819	1.2856	1.2910	1.2942	1.2996	1.3004
1.3057	1.3105	1.3130	1.3182	1.3194	1.3242	1.3296	1.3329
1.3378	1.3399	1.3425	1.3440	1.3462	1.3560	1.3635	1.3653
1.3692	1.3741	1.3795	1.3830	1.3860	1.3904	1.3956	1.4007
1.4044	1.4046	1.4079	1.4150	1.4156	1.4197	1.4269	1.4327
1.4340	1.4389	1.4434	1.4447	1.4513	1.4534	1.4588	1.4608
1.4632	1.4685	1.4719	1.4771	1.4812	1.4891	1.4944	1.5060
1.5070	1.5093	1.5200	1.5276	1.5320	1.5350	1.5389	1.5477
1.5525	1.5540	1.5622	1.5699	1.5712	1.5749	1.5809	1.5874
1.5899	1.5949	1.6027	1.6088	1.6153	1.6184	1.6263	1.6290
1.6357	1.6380	1.6400	1.6487	1.6571	1.6586	1.6653	1.6810
1.6863	1.6946	1.7056	1.7111	1.7284	1.7341	1.7418	1.7532
1.7546	1.7708	1.7768	1.7871	1.8075	1.8107	1.8323	1.8413
1.8514	1.8552	1.8691	1.8771	1.8823	1.9016	1.9121	1.9153
1.9798	1.9816	1.9853	2.0067	2.0086	2.0186	2.0291	2.0456
2.0496	2.0810	2.1000	2.1106	2.1315	2.2062	2.2491	2.2709
2.2803	2.2880	2.3117	2.3204	2.3378	2.3538	2.3629	2.3772
2.4022	2.4232	2.4425	2.4533	2.4744	2.4874	2.4916	2.4956
2.5099	2.5195	2.5267	2.5287	2.5387	2.5474	2.5519	2.5682
2.5778	2.5802	2.5859	2.5958	2.6020	2.6077	2.6103	2.6121
2.6265	2.6377	2.6523	2.6636	2.6705	2.6851	2.6874	2.6885
2.6959	2.6983	2.6994	2.7041	2.7053	2.7107	2.7252	2.7276
2.7361	2.7391	2.7424	2.7507	2.7551	2.7599	2.7620	2.7648
2.7709	2.7766	2.7793	2.7849	2.7870	2.7943	2.7981	2.8070
2.8097	2.8116	2.8151	2.8155	2.8168	2.8225	2.8261	2.8296
2.8343	2.8389	2.8438	2.8465	2.8492	2.8583	2.8599	2.8618
2.8637	2.8739	2.8814	2.8848	2.8864	2.8926	2.8990	2.9006
2.9044	2.9065	2.9110	2.9192	2.9193	2.9203	2.9231	2.9281
2.9291	2.9324	2.9353	2.9398	2.9449	2.9464	2.9484	2.9531
2.9560	2.9649	2.9697	2.9767	2.9794	2.9832	2.9858	2.9921
3.0031	3.0121	3.0158	3.0177	3.0277	3.0347	3.0456	3.0482
3.0536	3.0572	3.0576	3.0608	3.0696	3.0717	3.0816	3.0824
3.0890	3.0910	3.0969	3.1041	3.1077	3.1106	3.1127	3.1185
3.1243	3.1276	3.1313	3.1368	3.1462	3.1475	3.1491	3.1548
3.1565	3.1613	3.1623	3.1656	3.1682	3.1726	3.1790	3.1823
3.1871	3.1896	3.1904	3.1990	3.2073	3.2107	3.2133	3.2149
3.2210	3.2236	3.2279	3.2352	3.2385	3.2415	3.2539	3.2574
3.2581	3.2591	3.2684	3.2730	3.2758	3.2766	3.2811	3.2886
3.2920	3.2982	3.3006	3.3054	3.3102	3.3142	3.3224	3.3293
3.3305	3.3340	3.3367	3.3425	3.3496	3.3534	3.3576	3.3605
3.3677	3.3687	3.3823	3.3867	3.3889	3.3893	3.3931	3.4005

3.4116 3.4137 3.4202 3.4243 3.4285 3.4342 3.4377 3.4404
3.4444 3.4451 3.4594 3.4636 3.4692 3.4739 3.4791 3.4826
3.4851 3.4923 3.4937 3.5005 3.5070 3.5103 3.5117 3.5211
3.5290 3.5323 3.5350 3.5417 3.5428 3.5450 3.5536 3.5564
3.5601 3.5668 3.5713 3.5764 3.5825 3.5843 3.5947 3.6048
3.6155 3.6193 3.6272 3.6422 3.6483 3.6525 3.6591 3.6700
3.6711 3.6763 3.6772 3.6805 3.6858 3.6894 3.6946 3.6976
3.6992 3.7063 3.7095 3.7125 3.7141 3.7175 3.7204 3.7309
3.7315 3.7333 3.7462 3.7552 3.7587 3.7618 3.7759 3.7795
3.7848 3.7874 3.7957 3.7973 3.8056 3.8065 3.8071 3.8131
3.8157 3.8238 3.8269 3.8294 3.8354 3.8404 3.8448 3.8473
3.8540 3.8606 3.8678 3.8766 3.8793 3.8870 3.8924 3.9012
3.9072 3.9085 3.9124 3.9209 3.9270 3.9301 3.9403 3.9470
3.9615 3.9674 3.9763 3.9810 3.9905 3.9961 3.9990 4.0049
4.0082 4.0188 4.0200 4.0274 4.0316 4.0367 4.0482 4.0576
4.0587 4.0632 4.0676 4.0699 4.0732 4.0766 4.0823 4.0858
4.0892 4.1026 4.1167 4.1207 4.1272 4.1405 4.1448 4.1528
4.1593 4.1680 4.1726 4.1806 4.1840 4.1890 4.1900 4.1912
4.1988 4.2042 4.2109 4.2177 4.2234 4.2266 4.2372 4.2401
4.2481 4.2520 4.2616 4.2669 4.2739 4.2763 4.2885 4.2979
4.3108 4.3137 4.3148 4.3177 4.3199 4.3212 4.3261 4.3301
4.3388 4.3449 4.3464 4.3513 4.3552 4.3615 4.3656 4.3679
4.3823 4.3862 4.3936 4.3952 4.3977 4.4009 4.4080 4.4144
4.4294 4.4308 4.4400 4.4463 4.4489 4.4501 4.4525 4.4560
4.4591 4.4634 4.4651 4.4700 4.4779 4.4841 4.4849 4.4863
4.4936 4.4957 4.5043 4.5121 4.5136 4.5164 4.5294 4.5352
4.5513 4.5588 4.5595 4.5717 4.5787 4.5951 4.6008 4.6022
4.6114 4.6135 4.6343 4.6423 4.6491 4.6552 4.6591 4.6627
4.6680 4.6718 4.6831 4.7026 4.7138 4.7256 4.7475 4.7476
4.7512 4.7525 4.7844 4.8394 4.8465 4.8536 4.8635 4.8697
4.8846 4.8962 4.9226 4.9282 4.9303 5.0148 5.0192 5.0215
5.0338 5.0655 5.0776 5.0959 5.1129 5.1168 5.1239 5.1633
5.1689 5.1695 5.2489 5.2730 5.2827 5.2893 5.3727 5.3817
5.3892 5.4115 5.4223 5.4251 5.4340 5.4445 5.4521 5.4659
5.4772 5.5399 5.5829 5.5945 5.5950 5.5956 5.6200 5.6238
5.6255 5.6284 5.6329 5.6345 5.6376 5.6655 5.6830 5.6980
5.7408 5.7532 5.7560 5.7679 5.7770 5.7891 5.8153 5.8247
5.8367 6.0365 6.0406 6.0454 6.4754 6.4759 6.4774 8.1587
14.6205 14.7547 14.7849 24.1127 24.1437 24.1599 24.4296 24.5278
24.5956 24.6538 24.6803 24.6880 24.6890 24.6939 24.6988 24.7056
24.7194 24.7301 24.7362 24.9041 24.9095 24.9132 24.9550 24.9555
24.9625 25.3776 25.3849 25.3895 165.2932
GroundState
Mulliken Net Atomic Charges
Atom Charge (a.u.)
1
P 0.307673
2 C 0.413726
3 H 0.240319
4 H 0.245280
5 C 0.383713
6 C 0.232917
7 C 0.244170
8 C 0.241582
9 H 0.204894
10 C 0.240801
11 H 0.235027
12 C 0.385463

13 H 0.217102
14 H 0.226334
15 C 0.234854
16 H 0.189831
17 C 0.163277
18 C 0.154114
19 C 0.176270
20 C 0.085444
21 H 0.196400
22 C 0.083469
23 H 0.196079
24 C 0.119082
25 H 0.195267
26 C 0.175432
27 H 0.188223
28 C 0.130985
29 H 0.187478
30 C 0.190254
31 H 0.190568
32 C 0.274104
33 H 0.231946
34 C 0.248631
35 H 0.203889
36 C 0.252042
37 H 0.232129
38 C 0.638200
39 H 0.192277
40 H 0.190020
41 H 0.158623
42 C 0.615559
43 H 0.153648
44 H 0.184921
45 H 0.192093
46 C 0.616093
47 H 0.155946
48 H 0.182817
49 H 0.188687
50 C 0.335323
51 H 0.172669
52 H 0.169390
53 C 0.571642
54 H 0.169378
55 H 0.158378
56 H 0.156286
Sum
of atomic charges = 1.000000
Cartesian
Multipole Moments
Charge
(ESU x 10^10)
4.8032
Dipole Moment (Debye)
X 1.6561
Y 0.2476
Z 0.7341
Tot 1.8283
Quadrupole Moments (DebyeAng)
XX 137.8612

XY 3.1574
YY 123.2357
XZ 5.6628 YZ 2.1504
ZZ 122.2292
Traceless Quadrupole Moments (DebyeAng)
QXX 30.2574
QYY 13.6190 QZZ 16.6384
QXY 9.4721
QXZ 16.9885 QYZ 6.4512
Octopole Moments (DebyeAng^
2)
XXX 112.0680 XXY 27.9647 XYY 57.3146
YYY 40.4645
XXZ 8.6804
XYZ 4.6487
YYZ 22.3850 XZZ 37.9208
YZZ 79.4285
ZZZ 17.0445
Traceless Octopole Moments (DebyeAng^
2)
XXX 497.8639 YYY 1209.3260
ZZZ 21.0748
XXY 218.6842 XXZ 222.4531
XYY 465.3336
XYZ 69.7310 XZZ 963.1976
YYZ 243.5280
YZZ 990.6418
Hexadecapole Moments (DebyeAng^
3)
XXXX 3811.5993
XXXY 453.5169 XXYY 1529.8087
XYYY 277.2644 YYYY 4242.5777
XXXZ 394.0966
XXYZ 34.9206
XYYZ 140.6107
YYYZ 106.1186
XXZZ 1311.7914
XYZZ 5.0461
YYZZ 1393.7238
XZZZ 485.2283
YZZZ 24.0294 ZZZZ 4527.2992
Traceless Hexadecapole Moments (DebyeAng^
3)
XXXX 9100.9053 XXXY 14961.1885 XXXZ 4516.9645
XXYY 16496.6388
XXYZ 5094.6330
XXZZ 7395.7335
XYYY 3545.3230
XYYZ 534.9089 XYZZ 11415.8655
XZZZ 5051.8734
YYYY 10010.1391 YYYZ 6858.5407
YYZZ 6486.4997 YZZZ 1763.9077
ZZZZ 13882.2332
Total
job time: 10352.33s(wall), 41117.52s(cpu)
Fri Jan 13 21:16:17 2023

SPARTAN'20
build 1.1.4 (Dec 1 2021)
Wavefunction Developers:
B.J. Deppmeier, A.J. Driessen, W.J. Hehre, T.S. Hehre,
J.A. Johnson, W.S. Ohlinger, P.E. Klunzinger
Please cite Spartan as:
Spartan'20
Wavefunction Inc.
Irvine CA
QChem
5.1, QChem,
Inc., Pleasanton, CA (2020)
QChem
Developers:
Yihan Shao, Zhengting Gan, E. Epifanovsky, A. T. B. Gilbert, M. Wormit,
J. Kussmann, A. W. Lange, A. Behn, Jia Deng, Xintian Feng, D. Ghosh,
M. Goldey, P. R. Horn, L. D. Jacobson, I. Kaliman, T. Kus, A. Landau, Jie Liu,
E. I. Proynov, R. M. Richard, R. P. Steele, E. J. Sundstrom,
H. L. Woodcock III, P. M. Zimmerman, D. Zuev, B. Alam, B. Albrecht,
E. Alguire, S. A. Baeppler, D. Barton, Z. Benda, Y. A. Bernard,
E. J. Berquist, K. B. Bravaya, H. Burton, K. CarterFenk,
D. Casanova,
ChunMin
Chang, Yunqing Chen, A. Chien, K. D. Closser, M. P. Coons,
S. Coriani, S. Dasgupta, A. L. Dempwolff, M. Diedenhofen, Hainam Do,
R. G. Edgar, PoTung
Fang, S. Faraji, S. Fatehi, Qingguo Feng, J. FossoTande,
J. Gayvert, Qinghui Ge, A. Ghysels, G. Gidofalvi, J. Gomes, J. Gonthier,
A. Gunina, D. Hait, M. W. D. HansonHeine,
P. H. P. Harbach, A. W. Hauser,
M. F. Herbst, J. E. Herr, E. G. Hohenstein, Z. C. Holden, Kerwin Hui,
B. C. Huynh, T.C.
Jagau, Hyunjun Ji, B. Kaduk, K. Khistyaev, Jaehoon Kim,
P. Klunzinger, K. Koh, D. Kosenkov, L. Koulias, T. Kowalczyk, C. M. Krauter,
A. Kunitsa, Ka Un Lao, A. Laurent, K. V. Lawler, Joonho Lee, D. Lefrancois,
S. Lehtola, D. S. Levine, YiPei
Li, YouSheng
Lin, Fenglai Liu, KuanYu
Liu,
E. Livshits, M. Loipersberger, A. Luenser, P. Manohar, E. Mansoor,
S. F. Manzer, ShanPing
Mao, Yuezhi Mao, N. Mardirossian, A. V. Marenich,
T. Markovich, L. A. MartinezMartinez,
S. A. Maurer, N. J. Mayhall,
S. C. McKenzie, J.M.
Mewes, P. Morgante, A. F. Morrison, J. W. Mullinax,
K. Nanda, T. S. NguyenBeck,
R. OlivaresAmaya,
J. A. Parkhill, S. K. Paul,
Zheng Pei, T. M. Perrine, F. Plasser, P. Pokhilko, S. Prager, A. Prociuk,
E. Ramos, B. Rana, D. R. Rehn, F. Rob, M. Scheurer, M. Schneider, N. Sergueev,
S. M. Sharada, S. Sharma, D. W. Small, T. Stauch, C. J. Stein, T. Stein,
YuChuan
Su, S. P. Veccham, A. J. W. Thom, A. Tkatchenko, T. Tsuchimochi,
N. M. Tubman, L. Vogt, M. L. Vidal, O. Vydrov, M. A. Watson, J. Wenzel,
M. de Wergifosse, T. A. Wesolowski, A. White, J. Witte, A. Yamada, Jun Yang,
K. Yao, S. Yeganeh, S. R. Yost, ZhiQiang
You, A. Zech, Igor Ying Zhang,

```
Xing Zhang, Yan Zhao, Ying Zhu, B. R. Brooks, G. K. L. Chan, C. J. Cramer,
M. S. Gordon, W. J. Hehre, A. Klamt, M. W. Schmidt, C. D. Sherrill,
D. G. Truhlar, A. AspuruGuzik,
R. Baer, A. T. Bell, N. A. Besley,
JengDa
Chai, A. E. DePrince, III, R. A. DiStasio Jr., A. Dreuw,
B. D. Dunietz, T. R. Furlani, ChaoPing
Hsu, Yousung Jung, Jing Kong,
D. S. Lambrecht, WanZhen Liang, C. Ochsenfeld, V. A. Rassolov,
L. V. Slipchenko, J. E. Subotnik, T. Van Voorhis, J. M. Herbert, A. I. Krylov,
P. M. W. Gill, M. HeadGordon,
Contributors to earlier versions of QChem
not listed above:
R. D. Adamson, B. Austin, J. Baker, G. J. O. Beran, K. Brandhorst,
S. T. Brown, E. F. C. Byrd, A. K. Chakraborty, C.L.
Cheng, Siu Hung Chien,
D. M. Chipman, D. L. Crittenden, H. Dachsel, R. J. Doerksen, A. D. Dutoi,
L. FustiMolnar,
W. A. Goddard III, A. GolubevaZadorozhnaya,
S. R. Gwaltney,
G. Hawkins, A. Heyden, S. Hirata, G. Kedziora, F. J. Keil, C. Kelley,
Jihan Kim, R. A. King, R. Z. Khaliullin, P. P. Korambath, W. Kurlancheek,
A. M. Lee, M. S. Lee, S. V. Levchenko, Ching Yeh Lin, D. Liotard,
R. C. Lochan, I. Lotan, P. E. Maslen, N. Nair, D. P. O'Neill, D. Neuhauser,
E. Neuscamman, C. M. Oana, R. Olson, B. Peters, R. Peverati, P. A. Pieniazek,
Y. M. Rhee, J. Ritchie, M. A. Rohrdanz, E. Rosta, N. J. Russ,
H. F. Schaefer III, N. E. Schultz, N. Shenvi, A. C. Simmonett, A. Sodt,
D. Stuck, K. S. Thanthiriwatte, V. Vanovschi, Tao Wang, A. Warshel,
C. F. Williams, Q. Wu, X. Xu, W. Zhang,
Please cite QChem
as follows :
Y.Shao et al., Mol.Phys. 113, 184 215
(2015)
DOI : 10.1080/00268976.2014.952696
Parts of QChem
use Armadillo 8.300.2 (tropical Shenanigans).
http://arma.sourceforge.net/
Wavefunction Inc. Sales: sales@wavefun.com
Irvine CA Support: support@wavefun.com
Web: www.wavefun.com
Copyright © 1995 2021
Wavefunction
Version of QChem
Parts of QChem
use Armadillo 8.300.2 (Tropical Shenanigans).
http://arma.sourceforge.net/
QChem
begins on Tue Jan 10 14:20:26 2023
Scratch files written to
C:/Users/PATRIC~1/AppData/Local/Temp/WF1B20A4A6B6D3FB5D//scratch///
Processing $rem in system registry
... MEM_TOTAL 20000 # MB
Processing $rem in C:/Program
Files/Wavefunction/Spartan20v114/P4e//../auxdir/config/preferences:
(site specific preferences)
... THRESH 9
... SMALL_PROD_XCMAT 9
... BASIS_LIN_DEP_THRESH 5
```

```
... SCF_ALGORITHM DIIS_GDM
... MAXSCF 250
... MAXDIIS 45
... THRESHDIIS 1
(i.e. don't switch on deltaE)
... ECP_FIT TRUE (Convert deprecated ECP files)
... GUI GUI_SPARTAN
... TERSE_OUTPUT TRUE !turn on spartan printing
... SCF_CONVERGENCE 7
... CCMAN2 FALSE (qc4.3)
... SYMMETRY FALSE ! turn of symmetry for spartan16
... SYM_IGNORE TRUE ! ..use FORCESYMMETRY to override
... GEOM_OPT_TOL_GRADIENT 700 ! loosen tolernaces for organic geometries
... GEOM_OPT_TOL_DISPLACEMENT 1400 ! was 1200 = .0012
... GEOM_OPT_TOL_ENERGY 2000 ! was 100 = .000 001
... GEN_SCFMAN FALSE
Processing $rem in input file
... JOBTYPE SP
... SCF_CONVERGENCE 7 (sp default for single point energy)
... METHOD WB97MV
... xc_grid 75000302 (75,302)
... BASIS 6311+
G(2DF,2P)
... THRESH 12 #diffuse default
... MAXSCF 350 #diffuse default
... VARTHRESH 2 (default DFT)
... INCDFT TRUE (default DFT)
... GUI GUI_SPARTAN
... TERSE_OUTPUT TRUE
NAlpha2: 194
NElect 194
Mult 1
Warning: disabling incdft.
Checking the input file for inconsistencies... ...done.
User
input:
$
comment
4 Methyl TPP
$end
$molecule
1 1
15 0.23399922236
0.30461969597
0.078491614428
6 0.14993604835 1.1022359436 1.1267811395
6 0.73488020958 0.20958904324
1.4314193492
6 1.984803008
0.28489042272
0.32628450171
6 2.1111233401 0.04972306325 1.3711233426
1 2.5940879154 0.25366850553 0.42109815813
6 2.7082179931
1.4687959545
0.48123669291
1 2.2427275761
2.4351961742
```

0.32419455747
6 0.12054057558
1.0304347087 2.4984933377
1 0.50841301949
0.11927837537 2.94228811
6 4.0494243534
1.4170125233
0.84000846079
1 4.6055872697
2.340886447
0.9527788609
6 0.10697977984 2.1319149862 3.3051979865
1 0.098412783126
2.0663606552 4.3682532284
6 2.8601916677 0.062309843586 2.535186492
1 3.9244774973 0.26516819189 2.4821866123
6 0.59750684619 3.3290951911 2.7711410653
6 4.6908977295
0.19729551471
1.0555395645
6 2.266393778 0.17943954648
3.7796402838
6 3.9531434646
0.98107946737 0.89896940714
1 4.4368994862
1.9386946 1.0588879596
6 0.89621580299 0.43325208119
3.8251104492
1 0.41858065994 0.62001841052
4.7804412777
6 0.8543041998 3.3903166983 1.401951134
1 1.2343654438 4.3093673699 0.96990364194
6 0.63474506369 2.2911676983 0.58011511037
1 0.84574323867 2.3635154254 0.48139633538
6 0.12984283754 0.44820997288
2.6661199803
1 0.93594931693
0.64007464889
2.7278729565
6 2.6174750401
0.94571605057 0.53553990338
1 2.0701859752
1.8737576949 0.40584453792
6 6.1451001797
0.13780426423
1.4338787095
1 6.7289135034
0.32857032028 0.63630593806
1 6.287165138
0.4600949257 2.3366701417
1 6.5521798939
1.1327690512
1.6137682988
6 3.0913153418 0.13931422126
5.0362564974
1 3.3143622816 0.89541848617 5.3107910594
1 4.04324567 0.65487825784
4.8973583351

1 2.5665333055 0.60220470764
5.8721555507
6 0.82710955842 4.5187177745 3.6618204523
1 0.12508615927
4.9078162572 4.0317048811
1 1.4287561175 4.2442331248 4.5307745935
1 1.3373815599 5.3224141736 3.1310405733
6 0.14913265344 1.8439321999
0.96314576333
6 1.6069256723 1.9561924704
1.4220298915
6 1.8399838467 3.2303399644
2.2345117754
6 3.2909548505 3.3629394188
2.6870101096
1 3.9692545747 3.397481116
1.8301513889
1 3.5867856896 2.5183228764
3.3153159876
1 3.4411575239 4.2756499546
3.2655710966
1 0.096553383214
2.6633261724
0.27984467612
1 0.54223223888
1.9131814369
1.8102068809
1 1.8800960498 1.0831770582
2.0258570981
1 2.2668270167 1.9607011055
0.54839799915
1 1.557714384 4.1001469062
1.6309013382
1 1.1769968847 3.2294279134
3.1071633397
$end
$rem
JOBTYPE SP
SCF_CONVERGENCE 7 (sp default for single point energy)
METHOD WB97MV
xc_grid 75000302 (75,302)
BASIS 6311+
G(2DF,2P)
THRESH 12 #diffuse default
MAXSCF 350 #diffuse default
VARTHRESH 2 (default DFT)
INCDFT TRUE (default DFT)
GUI GUI_SPARTAN
TERSE_OUTPUT TRUE
$end
S
tandard Nuclear Orientation (Angstroms)
I Atom X Y Z
1
P 0.2339992224
0.3046196960
0.0784916144
2 C 0.1499360483 1.1022359436 1.1267811395

3 C 0.7348802096 0.2095890432
1.4314193492
4 C 1.9848030080
0.2848904227
0.3262845017
5 C 2.1111233401 0.0497230632 1.3711233426
6 H 2.5940879154 0.2536685055 0.4210981581
7 C 2.7082179931
1.4687959545
0.4812366929
8 H 2.2427275761
2.4351961742
0.3241945575
9 C 0.1205405756
1.0304347087 2.4984933377
10 H 0.5084130195
0.1192783754 2.9422881100
11 C 4.0494243534
1.4170125233
0.8400084608
12 H 4.6055872697
2.3408864470
0.9527788609
13 C 0.1069797798 2.1319149862 3.3051979865
14 H 0.0984127831
2.0663606552 4.3682532284
15 C 2.8601916677 0.0623098436 2.5351864920
16 H 3.9244774973 0.2651681919 2.4821866123
17 C 0.5975068462 3.3290951911 2.7711410653
18 C 4.6908977295
0.1972955147
1.0555395645
19 C 2.2663937780 0.1794395465
3.7796402838
20 C 3.9531434646
0.9810794674 0.8989694071
21 H 4.4368994862
1.9386946000 1.0588879596
22 C 0.8962158030 0.4332520812
3.8251104492
23 H 0.4185806599 0.6200184105
4.7804412777
24 C 0.8543041998 3.3903166983 1.4019511340
25 H 1.2343654438 4.3093673699 0.9699036419
26 C 0.6347450637 2.2911676983 0.5801151104
27 H 0.8457432387 2.3635154254 0.4813963354
28 C 0.1298428375 0.4482099729
2.6661199803
29 H 0.9359493169
0.6400746489
2.7278729565
30 C 2.6174750401
0.9457160506 0.5355399034
31 H 2.0701859752
1.8737576949 0.4058445379
32 C 6.1451001797
0.1378042642
1.4338787095

33 H 6.7289135034
0.3285703203 0.6363059381
34 H 6.2871651380
0.4600949257 2.3366701417
35 H 6.5521798939
1.1327690512
1.6137682988
36 C 3.0913153418 0.1393142213
5.0362564974
37 H 3.3143622816 0.8954184862 5.3107910594
38 H 4.0432456700 0.6548782578
4.8973583351
39 H 2.5665333055 0.6022047076
5.8721555507
40 C 0.8271095584 4.5187177745 3.6618204523
41 H 0.1250861593
4.9078162572 4.0317048811
42 H 1.4287561175 4.2442331248 4.5307745935
43 H 1.3373815599 5.3224141736 3.1310405733
44 C 0.1491326534 1.8439321999
0.9631457633
45 C 1.6069256723 1.9561924704
1.4220298915
46 C 1.8399838467 3.2303399644
2.2345117754
47 C 3.2909548505 3.3629394188
2.6870101096
48 H 3.9692545747 3.3974811160
1.8301513889
49 H 3.5867856896 2.5183228764
3.3153159876
50 H 3.4411575239 4.2756499546
3.2655710966
51 H 0.0965533832
2.6633261724
0.2798446761
52 H 0.5422322389
1.9131814369
1.8102068809
53 H 1.8800960498 1.0831770582
2.0258570981
54 H 2.2668270167 1.9607011055
0.5483979991
55 H 1.5577143840 4.1001469062
1.6309013382
56 H 1.1769968847 3.2294279134
3.1071633397
Nuclear
Repulsion Energy = 2433.07245751 hartrees
There are 97 alpha and 97 beta electrons
Requested basis set is 6311+
G(2df,2p)
There are 365 shells and 1162 basis functions
Total QAlloc Memory Limit 20000 MB
MegaArray
Size 188 MB
MEM_STATIC part 192 MB
.. (5.2.P)

Entering
fldman on Tue Jan 10 14:20:26 2023 A
cutoff of 1.0D12
yielded 39872 shell pairs
There are 414227 function pairs (502008 Cartesian)
Smallest overlap matrix eigenvalue = 2.69E07
Linear dependence detected in AO basis
Tighter screening thresholds may be required for diffuse basis sets
Use S2THRESH > 12 and THRESH = 14 in case of SCF convergence issues
Number of orthogonalized atomic orbitals = 1142
Maximum deviation from orthogonality = 1.632E11
Scale SEOQF with 1.000000e02/
1.000000e02/
1.000000e02
Standard Electronic Orientation quadrupole field applied
Nucleusfield
energy = 0.0000000016
hartrees
Entering
gesman on Tue Jan 10 14:20:30 2023 Guess
from superposition of atomic densities
Warning: Energy on first SCF cycle will be nonvariational
SAD guess density has 191.164804 electrons
Entering
scfman on Tue Jan 10 14:20:30 2023 Longrange
K will be added via erf
Coulomb attenuation parameter = 0.3 bohr**(1)
A restricted hybrid HFDFT
SCF calculation will be
performed using Pulay DIIS + Geometric Direct Minimization
Exchange: 0.1500 HartreeFock
+ 1.0000 wB97MV
+ LRHF
Correlation: 1.0000 wB97MV
Using EulerMaclaurinLebedev
(75,302) quadrature formula
Nonlocal Correlation: VV10 with C = 0.0100 and b = 6.00 and scale = 1.00000
Grid used for NLC: SG1
standard quadrature
SCF converges when RMS gradient is below 1.0E07
Exchange: 0.1500 HartreeFock
+ 1.0000 wB97MV
+ LRHF
Correlation: 1.0000 wB97MV
Using EulerMaclaurinLebedev
(75,302) quadrature formula
Nonlocal Correlation: VV10 with C = 0.0100 and b = 6.00 and scale = 1.00000
Grid used for NLC: SG1
standard quadrature
using 4 threads for integral computing
OpenMP
Integral computing Module
Release: version 1.0, May 2013, QChem
Inc. Pittsburgh
using
4 threads for integral computing
OpenMP
Integral computing Module

Release: version 1.0, May 2013, QChem
Inc. Pittsburgh
OpenMP
BLAS3 based DFT computing Module
Release: version 1.0, May 2013, QChem
Inc. Pittsburgh
OpenMP
BLAS3 based DFT computing Module
Release: version 1.0, May 2013, QChem
Inc. Pittsburgh
OpenMP
BLAS3 based DFT computing Module
Release: version 1.0, May 2013, QChem
Inc. Pittsburgh
Cycle
Energy DIIS Error
1
1311.8526736540
4.38E02
2 1182.7015512488
2.88E02
3 758.4882482782
3.61E02
4 1182.3328572786
2.07E02
5 1039.9374416839
3.06E02
6 1184.2277973687
1.64E02
7 464.2139675599
5.33E02
8 599.9317956417
4.65E02
9 629.2971480221
4.85E02
10 630.3983415266
4.84E02
VarThresh incresed to 3 to improve convergence.
11 637.1933783963
4.66E02
12 637.0820626122
4.66E02
13 637.2200887758
4.66E02
14 639.0062879793
4.61E02
VarThresh incresed to 4 to improve convergence.
15 639.0864717647
4.61E02
VarThresh incresed to 5 to improve convergence.
16 639.2635598081
4.60E02
17 596.4986067507
4.78E02
18 597.4212638672
5.09E02
19 344.4367984263
5.74E02

20 914.0265945888
3.63E02
21 1056.8138232468
2.56E02
22 155.8563747811 7.36E02
23 476.9811925285
4.49E02
24 1060.7591382458
2.12E02
25 739.4994978661
4.20E02
26 1249.4491503405
1.37E02
27 1032.8831823407
3.16E02
28 1297.8835366731
6.95E03
29 1297.6590854206
7.68E03
30 1311.4368610379
1.32E03
31 1311.6683008344
7.77E04
32 1311.7626090028
2.09E04
33 1311.7714014419
1.08E04
34 1311.7738512814
3.64E05
35 1311.7741919595
1.02E05
36 1311.7742207264
4.76E06
37 1311.7742281161
1.95E06
38 1311.7742304577
1.09E06
39 1311.7742309581
3.58E07
40 1311.7742310353
1.45E07
41 1311.7742310464
6.28E08
Convergence criterion met
SCF
time: CPU 64974.14 s wall 16360.68 s
SCF energy in the final basis set = 1311.77423105
Total energy in the final basis set = 1311.77423105
Entering
anlman on Tue Jan 10 18:53:12 2023 Orbital
Energies (a.u.)
Alpha
MOs
Occupied
77.5638
10.4865
10.4852
10.4842

10.4838
10.4781
10.4778
10.4766
10.4766
10.4759
10.4754
10.4752
10.4749
10.4748
10.4613
10.4605
10.4599
10.4597
10.4597
10.4594
10.4582
10.4384
10.4337
10.4327
10.4326
10.4156
6.9135
5.0256
5.0255
5.0253
1.1242
1.1091
1.1079
1.0648
1.0323
1.0155
1.0141
0.9943
0.9925
0.9909
0.9781
0.9414
0.9163
0.9152
0.8821
0.8547
0.8491
0.8443
0.8392
0.8173
0.8166
0.8058
0.7942
0.7454
0.7400
0.7086
0.7047
0.6956
0.6934
0.6908
0.6764
0.6759

0.6671
0.6515
0.6481
0.6479
0.6467
0.6411
0.6348
0.6339
0.6309
0.6271
0.6151
0.6115
0.5928
0.5900
0.5886
0.5876
0.5852
0.5811
0.5792
0.5709
0.5636
0.5558
0.5550
0.5524
0.5463
0.5438
0.5401
0.5270
0.5250
0.4707
0.4666
0.4642
0.4611
0.4540
0.4505
Virtual
0.0858
0.0839
0.0749
0.0671
0.0656
0.0578
0.0556
0.0519
0.0500
0.0487
0.0378
0.0355
0.0308
0.0298
0.0246
0.0202
0.0187
0.0152
0.0126
0.0109
0.0078
0.0024

0.0011	0.0029						
0.0050	0.0066	0.0104	0.0144	0.0181	0.0204	0.0236	0.0259
0.0268	0.0301	0.0314	0.0344	0.0371	0.0429	0.0453	0.0470
0.0487	0.0531	0.0557	0.0586	0.0606	0.0631	0.0655	0.0686
0.0702	0.0712	0.0746	0.0753	0.0795	0.0804	0.0840	0.0880
0.0928	0.0963	0.0999	0.1039	0.1057	0.1078	0.1089	0.1110
0.1149	0.1170	0.1195	0.1218	0.1231	0.1270	0.1290	0.1306
0.1350	0.1378	0.1402	0.1412	0.1435	0.1458	0.1473	0.1505
0.1514	0.1554	0.1593	0.1611	0.1627	0.1665	0.1688	0.1725
0.1732	0.1758	0.1761	0.1799	0.1827	0.1839	0.1882	0.1890
0.1909	0.1925	0.1944	0.1982	0.2010	0.2028	0.2059	0.2086
0.2100	0.2149	0.2161	0.2166	0.2187	0.2209	0.2244	0.2260
0.2272	0.2312	0.2334	0.2346	0.2359	0.2390	0.2423	0.2455
0.2466	0.2501	0.2528	0.2559	0.2609	0.2649	0.2746	0.2758
0.2789	0.2818	0.2822	0.2851	0.2888	0.2916	0.2951	0.3002
0.3052	0.3060	0.3135	0.3236	0.3317	0.3346	0.3427	0.3465
0.3513	0.3538	0.3703	0.3729	0.3812	0.3901	0.3914	0.3990
0.4019	0.4052	0.4056	0.4092	0.4178	0.4222	0.4290	0.4333
0.4360	0.4408	0.4457	0.4538	0.4572	0.4619	0.4662	0.4666
0.4730	0.4743	0.4786	0.4825	0.4862	0.4864	0.4891	0.4941
0.4987	0.5012	0.5052	0.5088	0.5102	0.5116	0.5152	0.5197
0.5226	0.5254	0.5300	0.5313	0.5338	0.5397	0.5416	0.5463
0.5475	0.5495	0.5533	0.5548	0.5574	0.5604	0.5655	0.5681
0.5723	0.5736	0.5750	0.5802	0.5838	0.5859	0.5872	0.5890
0.5937	0.5945	0.5998	0.6017	0.6036	0.6058	0.6095	0.6109
0.6140	0.6145	0.6182	0.6187	0.6214	0.6225	0.6264	0.6269
0.6295	0.6321	0.6340	0.6369	0.6376	0.6407	0.6433	0.6449
0.6480	0.6491	0.6497	0.6537	0.6561	0.6604	0.6641	0.6671
0.6692	0.6730	0.6743	0.6762	0.6824	0.6881	0.6905	0.6958
0.6981	0.7014	0.7017	0.7041	0.7116	0.7167	0.7204	0.7244
0.7267	0.7282	0.7305	0.7369	0.7406	0.7418	0.7452	0.7481
0.7536	0.7545	0.7569	0.7637	0.7657	0.7709	0.7739	0.7802
0.7808	0.7827	0.7930	0.7973	0.7997	0.8036	0.8089	0.8113
0.8129	0.8177	0.8255	0.8291	0.8323	0.8375	0.8418	0.8488
0.8522	0.8612	0.8627	0.8645	0.8696	0.8744	0.8795	0.8802
0.8820	0.8942	0.8957	0.9020	0.9062	0.9126	0.9150	0.9155
0.9260	0.9345	0.9358	0.9396	0.9438	0.9468	0.9526	0.9567
0.9596	0.9601	0.9680	0.9728	0.9783	0.9800	0.9915	0.9983
1.0013	1.0019	1.0098	1.0124	1.0182	1.0244	1.0280	1.0344
1.0349	1.0400	1.0461	1.0499	1.0569	1.0634	1.0645	1.0672
1.0696	1.0765	1.0795	1.0850	1.0858	1.0909	1.0966	1.0970
1.1058	1.1090	1.1138	1.1174	1.1214	1.1251	1.1280	1.1306
1.1355	1.1386	1.1468	1.1517	1.1539	1.1572	1.1645	1.1705
1.1820	1.1849	1.1882	1.1888	1.1963	1.1985	1.2012	1.2039
1.2078	1.2162	1.2210	1.2225	1.2296	1.2382	1.2417	1.2439
1.2468	1.2531	1.2540	1.2563	1.2578	1.2609	1.2685	1.2711
1.2752	1.2785	1.2793	1.2840	1.2888	1.2904	1.2940	1.2970
1.3013	1.3040	1.3108	1.3127	1.3142	1.3235	1.3322	1.3338
1.3389	1.3454	1.3479	1.3550	1.3639	1.3660	1.3694	1.3722
1.3780	1.3820	1.3827	1.3857	1.3895	1.3979	1.3984	1.4049
1.4061	1.4094	1.4103	1.4121	1.4163	1.4195	1.4224	1.4297
1.4316	1.4360	1.4394	1.4429	1.4486	1.4510	1.4534	1.4598
1.4614	1.4634	1.4721	1.4729	1.4808	1.4848	1.4863	1.4915
1.5036	1.5116	1.5144	1.5230	1.5274	1.5296	1.5330	1.5416
1.5441	1.5491	1.5540	1.5593	1.5631	1.5695	1.5733	1.5848
1.5880	1.5927	1.5966	1.5991	1.6056	1.6106	1.6147	1.6223
1.6237	1.6317	1.6359	1.6487	1.6502	1.6584	1.6623	1.6782
1.6853	1.6865	1.6970	1.7096	1.7223	1.7262	1.7463	1.7613

1.7677	1.7769	1.7839	1.7910	1.7922	1.8065	1.8222	1.8228
1.8347	1.8618	1.8721	1.8772	1.8845	1.8929	1.9107	1.9220
1.9779	1.9818	1.9883	1.9906	2.0023	2.0173	2.0241	2.0632
2.0753	2.0814	2.1273	2.1379	2.1467	2.1850	2.2272	2.2417
2.2545	2.2763	2.3156	2.3254	2.3312	2.3417	2.3671	2.3817
2.4130	2.4249	2.4400	2.4471	2.4726	2.4734	2.4826	2.4910
2.5033	2.5119	2.5190	2.5356	2.5445	2.5507	2.5626	2.5733
2.5779	2.5803	2.5826	2.5860	2.5913	2.6042	2.6100	2.6139
2.6171	2.6254	2.6299	2.6451	2.6702	2.6778	2.6818	2.6838
2.6959	2.7100	2.7149	2.7166	2.7218	2.7251	2.7280	2.7351
2.7385	2.7398	2.7441	2.7490	2.7498	2.7544	2.7622	2.7659
2.7726	2.7774	2.7796	2.7861	2.7894	2.7939	2.8022	2.8040
2.8071	2.8143	2.8202	2.8245	2.8272	2.8294	2.8334	2.8389
2.8411	2.8444	2.8477	2.8525	2.8559	2.8585	2.8617	2.8622
2.8706	2.8758	2.8798	2.8807	2.8839	2.8864	2.8912	2.8934
2.8979	2.9028	2.9045	2.9100	2.9137	2.9166	2.9180	2.9210
2.9230	2.9279	2.9298	2.9323	2.9384	2.9413	2.9456	2.9473
2.9505	2.9565	2.9625	2.9692	2.9731	2.9806	2.9870	2.9927
2.9980	3.0063	3.0152	3.0182	3.0295	3.0319	3.0355	3.0453
3.0563	3.0584	3.0702	3.0738	3.0753	3.0821	3.0823	3.0853
3.0921	3.0944	3.1013	3.1037	3.1073	3.1089	3.1169	3.1209
3.1269	3.1290	3.1309	3.1391	3.1411	3.1469	3.1501	3.1538
3.1564	3.1608	3.1646	3.1694	3.1725	3.1795	3.1845	3.1876
3.1883	3.1891	3.1991	3.2014	3.2021	3.2085	3.2136	3.2158
3.2192	3.2236	3.2277	3.2319	3.2338	3.2411	3.2441	3.2492
3.2529	3.2617	3.2632	3.2693	3.2739	3.2753	3.2861	3.2872
3.2930	3.2958	3.2998	3.3046	3.3091	3.3161	3.3166	3.3232
3.3277	3.3326	3.3376	3.3474	3.3490	3.3526	3.3574	3.3629
3.3697	3.3709	3.3761	3.3797	3.3857	3.3927	3.3960	3.4013
3.4035	3.4082	3.4099	3.4163	3.4169	3.4316	3.4361	3.4465
3.4480	3.4516	3.4576	3.4637	3.4678	3.4701	3.4772	3.4813
3.4899	3.4986	3.5033	3.5051	3.5127	3.5128	3.5177	3.5281
3.5313	3.5323	3.5359	3.5408	3.5429	3.5508	3.5529	3.5543
3.5562	3.5624	3.5647	3.5682	3.5736	3.5837	3.5897	3.5961
3.6113	3.6124	3.6225	3.6265	3.6328	3.6440	3.6478	3.6523
3.6619	3.6726	3.6735	3.6775	3.6816	3.6854	3.6897	3.6952
3.6981	3.7025	3.7051	3.7111	3.7135	3.7168	3.7243	3.7269
3.7315	3.7421	3.7461	3.7515	3.7553	3.7647	3.7656	3.7686
3.7816	3.7882	3.7917	3.7970	3.7996	3.8050	3.8066	3.8115
3.8165	3.8206	3.8255	3.8302	3.8400	3.8458	3.8507	3.8613
3.8668	3.8722	3.8772	3.8858	3.8901	3.8945	3.9039	3.9121
3.9147	3.9202	3.9282	3.9381	3.9557	3.9623	3.9656	3.9764
3.9783	3.9826	3.9852	3.9883	3.9921	3.9972	4.0034	4.0083
4.0114	4.0138	4.0165	4.0221	4.0235	4.0258	4.0346	4.0386
4.0488	4.0555	4.0575	4.0616	4.0641	4.0707	4.0840	4.0931
4.0964	4.1041	4.1073	4.1129	4.1166	4.1252	4.1275	4.1385
4.1522	4.1589	4.1675	4.1787	4.1845	4.1858	4.1910	4.1945
4.2007	4.2076	4.2134	4.2189	4.2225	4.2290	4.2325	4.2376
4.2390	4.2441	4.2513	4.2622	4.2792	4.2839	4.2862	4.2995
4.3018	4.3047	4.3156	4.3195	4.3210	4.3298	4.3318	4.3351
4.3402	4.3439	4.3514	4.3557	4.3588	4.3627	4.3692	4.3693
4.3741	4.3767	4.3816	4.3844	4.3928	4.3946	4.4028	4.4055
4.4103	4.4107	4.4225	4.4433	4.4475	4.4569	4.4580	4.4610
4.4681	4.4709	4.4713	4.4744	4.4788	4.4817	4.4847	4.4945
4.4970	4.5032	4.5123	4.5202	4.5228	4.5312	4.5359	4.5423
4.5467	4.5478	4.5626	4.5691	4.5856	4.5947	4.5968	4.6058
4.6123	4.6162	4.6287	4.6363	4.6385	4.6580	4.6614	4.6653
4.6717	4.6812	4.6832	4.7333	4.7351	4.7428	4.7460	4.7513

4.7594 4.7694 4.7719 4.7865 4.8043 4.8440 4.8498 4.8882
4.9115 4.9188 4.9269 4.9330 4.9398 5.0302 5.0369 5.0473
5.0528 5.0658 5.0730 5.0766 5.0818 5.0859 5.1114 5.1626
5.1671 5.1768 5.2649 5.2734 5.2808 5.2902 5.3549 5.3632
5.3824 5.4039 5.4147 5.4242 5.4347 5.4567 5.4663 5.4696
5.4730 5.5383 5.5855 5.5973 5.5986 5.6081 5.6225 5.6295
5.6315 5.6324 5.6536 5.6607 5.6646 5.6707 5.6847 5.6891
5.6957 5.6976 5.7008 5.7707 5.7744 5.7843 5.8003 5.8111
5.8184 6.0379 6.0451 6.0497 6.4719 6.4728 6.4747 8.1780
14.6629 14.7388 14.7717 24.1049 24.1172 24.1275 24.4284 24.5243
24.5972 24.6560 24.6731 24.6746 24.6812 24.7061 24.7074 24.7170
24.7223 24.7254 24.7294 24.8918 24.8948 24.9029 24.9753 24.9759
24.9850 25.3761 25.3817 25.3878 165.3160
GroundState
Mulliken Net Atomic Charges
Atom Charge (a.u.)
1
P 0.115995
2 C 0.275976
3 C 0.289161
4 C 0.156072
5 C 0.276199
6 H 0.248592
7 C 0.222472
8 H 0.202525
9 C 0.242883
10 H 0.195712
11 C 0.240604
12 H 0.185703
13 C 0.213461
14 H 0.187607
15 C 0.213356
16 H 0.187239
17 C 0.097585
18 C 0.101824
19 C 0.115899
20 C 0.169898
21 H 0.186580
22 C 0.218625
23 H 0.185993
24 C 0.211898
25 H 0.187071
26 C 0.314078
27 H 0.244908
28 C 0.281545
29 H 0.237949
30 C 0.239471
31 H 0.217239
32 C 0.422632
33 H 0.181357
34 H 0.175486
35 H 0.161291
36 C 0.396333
37 H 0.182903
38 H 0.170378
39 H 0.159318
40 C 0.370424
41 H 0.182691

42 H 0.175118
43 H 0.156567
44 C 0.335039
45 C 0.384714
46 C 0.342810
47 C 0.559021
48 H 0.157471
49 H 0.156603
50 H 0.168837
51 H 0.214245
52 H 0.240013
53 H 0.214514
54 H 0.225085
55 H 0.173726
56 H 0.172220
Sum
of atomic charges = 1.000000
Cartesian
Multipole Moments
Charge
(ESU x 10^10)
4.8032
Dipole Moment (Debye)
X 1.2272
Y 0.7119 Z 0.5704
Tot 1.5291
Quadrupole Moments (DebyeAng)
XX 119.3612
XY 2.9143 YY 133.8555
XZ 0.5208
YZ 7.9447
ZZ 123.5496
Traceless Quadrupole Moments (DebyeAng)
QXX 18.6828 QYY 24.8002
QZZ 6.1175
QXY 8.7428 QXZ 1.5624
QYZ 23.8342
Octopole Moments (DebyeAng^
2)
XXX 71.7382
XXY 27.5383
XYY 29.1769
YYY 5.1584 XXZ 66.6522 XYZ 2.7804
YYZ 79.1194
XZZ 76.1054 YZZ 14.0890
ZZZ 7.6331
Traceless Octopole Moments (DebyeAng^
2)
XXX 1377.9705
YYY 151.9942 ZZZ 66.4055
XXY 388.2013
XXZ 1060.0839 XYY 337.0215
XYZ 41.7057 XZZ 1040.9490 YYZ 1126.4894
YZZ 236.2071
Hexadecapole Moments (DebyeAng^
3)
XXXX 5097.4198
XXXY 147.0429 XXYY 1570.6070

XYYY 213.3817 YYYY 3379.3115
XXXZ 33.6339
XXYZ 173.5161 XYYZ 44.8219
YYYZ 293.2440
XXZZ 1735.1650
XYZZ 49.5399 YYZZ 1335.2487
XZZZ 382.5177 YZZZ 260.2488 ZZZZ 4564.5687
Traceless Hexadecapole Moments (DebyeAng^
3)
XXXX 20148.1088 XXXY 3008.8947
XXXZ 13178.2817
XXYY 11558.3697
XXYZ 7317.7901 XXZZ 8589.7391
XYYY 3956.6745 XYYZ 10276.2414
XYZZ 947.7798
XZZZ 23454.5231 YYYY 9927.2647 YYYZ 1913.5857
YYZZ 1631.1050 YZZZ 5404.2044
ZZZZ 6958.6341
Total
job time: 16368.20s(wall), 64983.78s(cpu)
Tue Jan 10 18:53:14 2023

SPARTAN'20
build 1.1.4 (Dec 1 2021)
Wavefunction Developers:
B.J. Deppmeier, A.J. Driessen, W.J. Hehre, T.S. Hehre,
J.A. Johnson, W.S. Ohlinger, P.E. Klunzinger
Please cite Spartan as:
Spartan'20
Wavefunction Inc.
Irvine CA
QChem
5.1, QChem,
Inc., Pleasanton, CA (2020)
QChem
Developers:
Yihan Shao, Zhengting Gan, E. Epifanovsky, A. T. B. Gilbert, M. Wormit,
J. Kussmann, A. W. Lange, A. Behn, Jia Deng, Xintian Feng, D. Ghosh,
M. Goldey, P. R. Horn, L. D. Jacobson, I. Kaliman, T. Kus, A. Landau, Jie Liu,
E. I. Proynov, R. M. Richard, R. P. Steele, E. J. Sundstrom,
H. L. Woodcock III, P. M. Zimmerman, D. Zuev, B. Alam, B. Albrecht,
E. Alguire, S. A. Baeppler, D. Barton, Z. Benda, Y. A. Bernard,
E. J. Berquist, K. B. Bravaya, H. Burton, K. CarterFenk,
D. Casanova,
ChunMin
Chang, Yunqing Chen, A. Chien, K. D. Closser, M. P. Coons,
S. Coriani, S. Dasgupta, A. L. Dempwolff, M. Diedenhofen, Hainam Do,
R. G. Edgar, PoTung
Fang, S. Faraji, S. Fatehi, Qingguo Feng, J. FossoTande,
J. Gayvert, Qinghui Ge, A. Ghysels, G. Gidofalvi, J. Gomes, J. Gonthier,
A. Gunina, D. Hait, M. W. D. HansonHeine,
P. H. P. Harbach, A. W. Hauser,
M. F. Herbst, J. E. Herr, E. G. Hohenstein, Z. C. Holden, Kerwin Hui,
B. C. Huynh, T.C.
Jagau, Hyunjun Ji, B. Kaduk, K. Khistyaev, Jaehoon Kim,
P. Klunzinger, K. Koh, D. Kosenkov, L. Koulias, T. Kowalczyk, C. M. Krauter,
A. Kunitsa, Ka Un Lao, A. Laurent, K. V. Lawler, Joonho Lee, D. Lefrancois,
S. Lehtola, D. S. Levine, YiPei
Li, YouSheng
Lin, Fenglai Liu, KuanYu
Liu,
E. Livshits, M. Loipersberger, A. Luenser, P. Manohar, E. Mansoor,
S. F. Manzer, ShanPing
Mao, Yuezhi Mao, N. Mardirossian, A. V. Marenich,
T. Markovich, L. A. MartinezMartinez,
S. A. Maurer, N. J. Mayhall,
S. C. McKenzie, J.M.
Mewes, P. Morgante, A. F. Morrison, J. W. Mullinax,
K. Nanda, T. S. NguyenBeck,
R. OlivaresAmaya,
J. A. Parkhill, S. K. Paul,
Zheng Pei, T. M. Perrine, F. Plasser, P. Pokhilko, S. Prager, A. Prociuk,
E. Ramos, B. Rana, D. R. Rehn, F. Rob, M. Scheurer, M. Schneider, N. Sergueev,
S. M. Sharada, S. Sharma, D. W. Small, T. Stauch, C. J. Stein, T. Stein,
YuChuan
Su, S. P. Veccham, A. J. W. Thom, A. Tkatchenko, T. Tsuchimochi,
N. M. Tubman, L. Vogt, M. L. Vidal, O. Vydrov, M. A. Watson, J. Wenzel,
M. de Wergifosse, T. A. Wesolowski, A. White, J. Witte, A. Yamada, Jun Yang,
K. Yao, S. Yeganeh, S. R. Yost, ZhiQiang
You, A. Zech, Igor Ying Zhang,

```
Xing Zhang, Yan Zhao, Ying Zhu, B. R. Brooks, G. K. L. Chan, C. J. Cramer,
M. S. Gordon, W. J. Hehre, A. Klamt, M. W. Schmidt, C. D. Sherrill,
D. G. Truhlar, A. AspuruGuzik,
R. Baer, A. T. Bell, N. A. Besley,
JengDa
Chai, A. E. DePrince, III, R. A. DiStasio Jr., A. Dreuw,
B. D. Dunietz, T. R. Furlani, ChaoPing
Hsu, Yousung Jung, Jing Kong,
D. S. Lambrecht, WanZhen Liang, C. Ochsenfeld, V. A. Rassolov,
L. V. Slipchenko, J. E. Subotnik, T. Van Voorhis, J. M. Herbert, A. I. Krylov,
P. M. W. Gill, M. HeadGordon,
Contributors to earlier versions of QChem
not listed above:
R. D. Adamson, B. Austin, J. Baker, G. J. O. Beran, K. Brandhorst,
S. T. Brown, E. F. C. Byrd, A. K. Chakraborty, C.L.
Cheng, Siu Hung Chien,
D. M. Chipman, D. L. Crittenden, H. Dachsel, R. J. Doerksen, A. D. Dutoi,
L. FustiMolnar,
W. A. Goddard III, A. GolubevaZadorozhnaya,
S. R. Gwaltney,
G. Hawkins, A. Heyden, S. Hirata, G. Kedziora, F. J. Keil, C. Kelley,
Jihan Kim, R. A. King, R. Z. Khaliullin, P. P. Korambath, W. Kurlancheek,
A. M. Lee, M. S. Lee, S. V. Levchenko, Ching Yeh Lin, D. Liotard,
R. C. Lochan, I. Lotan, P. E. Maslen, N. Nair, D. P. O'Neill, D. Neuhauser,
E. Neuscamman, C. M. Oana, R. Olson, B. Peters, R. Peverati, P. A. Pieniazek,
Y. M. Rhee, J. Ritchie, M. A. Rohrdanz, E. Rosta, N. J. Russ,
H. F. Schaefer III, N. E. Schultz, N. Shenvi, A. C. Simmonett, A. Sodt,
D. Stuck, K. S. Thanthiriwatte, V. Vanovschi, Tao Wang, A. Warshel,
C. F. Williams, Q. Wu, X. Xu, W. Zhang,
Please cite QChem
as follows :
Y.Shao et al., Mol.Phys. 113, 184 215
(2015)
DOI : 10.1080/00268976.2014.952696
Parts of QChem
use Armadillo 8.300.2 (tropical Shenanigans).
http://arma.sourceforge.net/
Wavefunction Inc. Sales: sales@wavefun.com
Irvine CA Support: support@wavefun.com
Web: www.wavefun.com
Copyright © 1995 2021
Wavefunction
Version of QChem
Parts of QChem
use Armadillo 8.300.2 (Tropical Shenanigans).
http://arma.sourceforge.net/
QChem
begins on Sun Jan 8 18:42:51 2023
Scratch files written to
C:/Users/PATRIC~1/AppData/Local/Temp/WF5ECB430A93ABF53F//scratch///
Processing $rem in system registry
... MEM_TOTAL 20000 # MB
Processing $rem in C:/Program
Files/Wavefunction/Spartan20v114/P4e//../auxdir/config/preferences:
(site specific preferences)
... THRESH 9
... SMALL_PROD_XCMAT 9
... BASIS_LIN_DEP_THRESH 5
```

```
... SCF_ALGORITHM DIIS_GDM
... MAXSCF 250
... MAXDIIS 45
... THRESHDIIS 1
(i.e. don't switch on deltaE)
... ECP_FIT TRUE (Convert deprecated ECP files)
... GUI GUI_SPARTAN
... TERSE_OUTPUT TRUE !turn on spartan printing
... SCF_CONVERGENCE 7
... CCMAN2 FALSE (qc4.3)
... SYMMETRY FALSE ! turn of symmetry for spartan16
... SYM_IGNORE TRUE ! ..use FORCESYMMETRY to override
... GEOM_OPT_TOL_GRADIENT 700 ! loosen tolernaces for organic geometries
... GEOM_OPT_TOL_DISPLACEMENT 1400 ! was 1200 = .0012
... GEOM_OPT_TOL_ENERGY 2000 ! was 100 = .000 001
... GEN_SCFMAN FALSE
Processing $rem in input file
... JOBTYPE SP
... SCF_CONVERGENCE 7 (sp default for single point energy)
... METHOD WB97MV
... xc_grid 75000302 (75,302)
... BASIS 6311+
G(2DF,2P)
... THRESH 12 #diffuse default
... MAXSCF 350 #diffuse default
... VARTHRESH 2 (default DFT)
... INCDFT TRUE (default DFT)
... GUI GUI_SPARTAN
... TERSE_OUTPUT TRUE
NAlpha2: 194
NElect 194
Mult 1
Warning: disabling incdft.
Checking the input file for inconsistencies... ...done.
User
input:
$
comment
4F
BuTPP Cation
$end
$molecule
1 1
15 0.020464733131 0.010105990555
0.39765102578
9 2.6201630242 4.997810648
1.3179561784
9 0.27500649981 0.4313816966 6.2566937876
9 2.7875357178 4.6677856625 1.8407212028
6 1.7188602812
0.023511564619
0.12369063561
1 2.17917007
0.87785608543 0.29606842508
1 2.1879299005
0.87728189138
0.37582476325
6 1.9338442265
```

0.10742252454
1.6387154765
1 1.5668908283
1.0701376428
2.0088729298
1 1.3575714519
0.67462633983 2.1472155267
6 3.4133186676
0.03819887545 1.9976433467
1 3.7798547615
1.0042598719 1.6325610393
1 3.9893905473
0.73149633777
1.4722517742
6 3.6510407924
0.072215310107
3.5005218165
1 3.1049743013
0.70308606966 4.0448596288
1 3.3245434044
1.0436698845
3.8812906014
1 4.7096317475
0.038104939053 3.7396083972
6 0.84377878159 1.5227822797
0.11619316907
6 0.85876090379 1.3949059071 0.34352591532
6 0.075168802513 0.14167048529 2.1880756195
6 1.0672657964 0.92627435886 2.7850882192
1 1.7803802905 1.4694308348 2.1751189044
6 2.1998758993 1.299376715 0.72917203584
1 2.7346708976 0.36074496143 0.6362544743
6 1.2156842327 2.4805992552
0.83158177097
1 1.0465024884 2.3055922471
1.8877575101
6 2.8593178342 2.4089278518 1.2379793098
1 3.8956476935 2.3646844215 1.5472433457
6 1.1401469792 1.0265603868 4.1662363079
1 1.8939371987 1.6297000183 4.6556454636
6 1.8179111738 3.6639347829
0.42800663092
1 2.1189111922 4.4221037779
1.1393628504
6 2.1592272692 3.5991695853 1.3493474991
6 2.0397297529 3.8633912085
0.92388598445
6 0.21172836377 0.33576969057 4.9278954237
6 0.8305011223 3.7270106357 0.97075824761
1 0.33148393935 4.6815077366 1.0777319104
6 0.78456187179
0.44929763542
4.3665920312
1 1.4871478178
0.96498022263
5.0083530472
6 1.6921445525 2.9277052866
1.8886353866

1 1.8991063002 3.1306094881
2.9315255095
6 1.0903605812 1.7504960214
1.4781465748
1 0.82978102553 1.00411205
2.2207766109
6 0.84908011318
0.54511013558
2.9858655336
1 1.6233425056
1.1614042355
2.542925673
6 0.18116461515 2.6149313989 0.46152369807
1 0.85520437223
2.7098058928 0.15613476222
$end
$rem
JOBTYPE SP
SCF_CONVERGENCE 7 (sp default for single point energy)
METHOD WB97MV
xc_grid 75000302 (75,302)
BASIS 6311+
G(2DF,2P)
THRESH 12 #diffuse default
MAXSCF 350 #diffuse default
VARTHRESH 2 (default DFT)
INCDFT TRUE (default DFT)
GUI GUI_SPARTAN
TERSE_OUTPUT TRUE
$end
S
tandard Nuclear Orientation (Angstroms)
I Atom X Y Z
1
P 0.0204647331 0.0101059906
0.3976510258
2 F 2.6201630242 4.9978106480
1.3179561784
3 F 0.2750064998 0.4313816966 6.2566937876
4 F 2.7875357178 4.6677856625 1.8407212028
5 C 1.7188602812
0.0235115646
0.1236906356
6 H 2.1791700700
0.8778560854 0.2960684251
7 H 2.1879299005
0.8772818914
0.3758247633
8 C 1.9338442265
0.1074225245
1.6387154765
9 H 1.5668908283
1.0701376428
2.0088729298
10 H 1.3575714519
0.6746263398 2.1472155267
11 C 3.4133186676
0.0381988755 1.9976433467

12 H 3.7798547615
1.0042598719 1.6325610393
13 H 3.9893905473
0.7314963378
1.4722517742
14 C 3.6510407924
0.0722153101
3.5005218165
15 H 3.1049743013
0.7030860697 4.0448596288
16 H 3.3245434044
1.0436698845
3.8812906014
17 H 4.7096317475
0.0381049391 3.7396083972
18 C 0.8437787816 1.5227822797
0.1161931691
19 C 0.8587609038 1.3949059071 0.3435259153
20 C 0.0751688025 0.1416704853 2.1880756195
21 C 1.0672657964 0.9262743589 2.7850882192
22 H 1.7803802905 1.4694308348 2.1751189044
23 C 2.1998758993 1.2993767150 0.7291720358
24 H 2.7346708976 0.3607449614 0.6362544743
25 C 1.2156842327 2.4805992552
0.8315817710
26 H 1.0465024884 2.3055922471
1.8877575101
27 C 2.8593178342 2.4089278518 1.2379793098
28 H 3.8956476935 2.3646844215 1.5472433457
29 C 1.1401469792 1.0265603868 4.1662363079
30 H 1.8939371987 1.6297000183 4.6556454636
31 C 1.8179111738 3.6639347829
0.4280066309
32 H 2.1189111922 4.4221037779
1.1393628504
33 C 2.1592272692 3.5991695853 1.3493474991
34 C 2.0397297529 3.8633912085
0.9238859845
35 C 0.2117283638 0.3357696906 4.9278954237
36 C 0.8305011223 3.7270106357 0.9707582476
37 H 0.3314839393 4.6815077366 1.0777319104
38 C 0.7845618718
0.4492976354
4.3665920312
39 H 1.4871478178
0.9649802226
5.0083530472
40 C 1.6921445525 2.9277052866
1.8886353866
41 H 1.8991063002 3.1306094881
2.9315255095
42 C 1.0903605812 1.7504960214
1.4781465748
43 H 0.8297810255 1.0041120500
2.2207766109
44 C 0.8490801132
0.5451101356
2.9858655336

45 H 1.6233425056
1.1614042355
2.5429256730
46 C 0.1811646152 2.6149313989 0.4615236981
47 H 0.8552043722
2.7098058928 0.1561347622
Nuclear
Repulsion Energy = 2447.99701716 hartrees
There are 97 alpha and 97 beta electrons
Requested basis set is 6311+
G(2df,2p)
There are 320 shells and 1081 basis functions
Total QAlloc Memory Limit 20000 MB
MegaArray
Size 188 MB
MEM_STATIC part 192 MB
.. (5.2.P)
Entering
fldman on Sun Jan 8 18:42:51 2023 A
cutoff of 1.0D12
yielded 33023 shell pairs
There are 373201 function pairs (456643 Cartesian)
Smallest overlap matrix eigenvalue = 4.60E07
Linear dependence detected in AO basis
Tighter screening thresholds may be required for diffuse basis sets
Use S2THRESH > 12 and THRESH = 14 in case of SCF convergence issues
Number of orthogonalized atomic orbitals = 1067
Maximum deviation from orthogonality = 3.421E11
Scale SEOQF with 1.000000e01/
1.000000e01/
1.000000e02
Standard Electronic Orientation quadrupole field applied
Nucleusfield
energy = 0.0000000875 hartrees
Entering
gesman on Sun Jan 8 18:42:55 2023 Guess
from superposition of atomic densities
Warning: Energy on first SCF cycle will be nonvariational
SAD guess density has 191.164804 electrons
Entering
scfman on Sun Jan 8 18:42:55 2023 Longrange
K will be added via erf
Coulomb attenuation parameter = 0.3 bohr**(1)
A restricted hybrid HFDFT
SCF calculation will be
performed using Pulay DIIS + Geometric Direct Minimization
Exchange: 0.1500 HartreeFock
+ 1.0000 wB97MV
+ LRHF
Correlation: 1.0000 wB97MV
Using EulerMaclaurinLebedev
(75,302) quadrature formula
Nonlocal Correlation: VV10 with C = 0.0100 and b = 6.00 and scale = 1.00000
Grid used for NLC: SG1
standard quadrature
SCF converges when RMS gradient is below 1.0E07
Exchange: 0.1500 HartreeFock
+ 1.0000 wB97MV

```
+ LRHF
Correlation: 1.0000 wB97MV
Using EulerMaclaurinLebedev
(75,302) quadrature formula
Nonlocal Correlation: VV10 with C = 0.0100 and b = 6.00 and scale = 1.00000
Grid used for NLC: SG1
standard quadrature
using 4 threads for integral computing
OpenMP
Integral computing Module
Release: version 1.0, May 2013, QChem
Inc. Pittsburgh
using
4 threads for integral computing
OpenMP
Integral computing Module
Release: version 1.0, May 2013, QChem
Inc. Pittsburgh
OpenMP
BLAS3 based DFT computing Module
Release: version 1.0, May 2013, QChem
Inc. Pittsburgh
OpenMP
BLAS3 based DFT computing Module
Release: version 1.0, May 2013, QChem
Inc. Pittsburgh
OpenMP
BLAS3 based DFT computing Module
Release: version 1.0, May 2013, QChem
Inc. Pittsburgh
Cycle
Energy DIIS Error
1
1491.3042065741
4.68E02
2 1375.5601907816
2.93E02
3 1055.3405995907
3.11E02
4 1204.5630528124
3.64E02
5 1440.6041537688
1.60E02
6 1305.8219447952
2.49E02
7 1456.9225375026
1.01E02
8 1249.1730820652
2.85E02
9 1414.8809117462
1.54E02
10 1485.2582650125
4.75E03
11 1448.1818111902
1.16E02
12 1491.1265566853
1.69E03
13 1491.5181801710
```

6.97E04
14 1491.5943646434
3.47E04
15 1491.6157744340
1.21E04
16 1491.6185502951
5.29E05
17 1491.6190043825
2.30E05
18 1491.6190817409
9.87E06
19 1491.6190957659
3.49E06
20 1491.6190980905
1.57E06
21 1491.6190986436
6.00E07
22 1491.6190987265
1.78E07
23 1491.6190987326
7.61E08
Convergence criterion met
SCF
time: CPU 29673.39 s wall 7492.16 s
SCF energy in the final basis set = 1491.61909873
Total energy in the final basis set = 1491.61909873
Entering
anlman on Sun Jan 8 20:47:48 2023 Orbital
Energies (a.u.)
Alpha
MOs
Occupied
77.5845
25.0373
25.0356
25.0353
10.5691
10.5673
10.5670
10.5071
10.5059
10.5054
10.5046
10.5036
10.5029
10.5028
10.5019
10.5010
10.5008
10.4865
10.4856
10.4851
10.4845
10.4837
10.4829
10.4743
10.4498
10.4248

6.9340
5.0461
5.0460
5.0456
1.4871
1.4854
1.4851
1.1477
1.1328
1.1315
1.0802
1.0430
1.0241
1.0227
1.0206
1.0111
1.0105
0.9831
0.9254
0.8898
0.8838
0.8808
0.8712
0.8640
0.8634
0.8210
0.8178
0.7940
0.7913
0.7876
0.7510
0.7485
0.7423
0.7306
0.7271
0.7157
0.7097
0.7081
0.7077
0.6937
0.6899
0.6865
0.6830
0.6748
0.6740
0.6723
0.6564
0.6499
0.6447
0.6395
0.6385
0.6310
0.6215
0.6156
0.6117
0.5988
0.5930
0.5883

0.5829
0.5781
0.5771
0.5746
0.5536
0.5393
0.5380
0.4970
0.4912
0.4891
0.4829
0.4755
0.4735
Virtual
0.1030
0.0992
0.0909
0.0865
0.0773
0.0746
0.0723
0.0659
0.0635
0.0595
0.0473
0.0452
0.0398
0.0365
0.0305
0.0237
0.0212
0.0207
0.0157
0.0147
0.0127
0.0072
0.0030
0.0005

0.0038	0.0051	0.0103	0.0127	0.0140	0.0173	0.0173	0.0220
0.0289	0.0329	0.0338	0.0382	0.0386	0.0426	0.0433	0.0462
0.0541	0.0557	0.0584	0.0598	0.0642	0.0647	0.0675	0.0697
0.0714	0.0755	0.0765	0.0813	0.0837	0.0854	0.0883	0.0926
0.0955	0.1000	0.1005	0.1018	0.1038	0.1070	0.1079	0.1090
0.1125	0.1185	0.1209	0.1228	0.1281	0.1304	0.1336	0.1350
0.1382	0.1397	0.1410	0.1434	0.1465	0.1512	0.1529	0.1566
0.1582	0.1621	0.1641	0.1655	0.1672	0.1692	0.1733	0.1739
0.1749	0.1771	0.1809	0.1816	0.1863	0.1898	0.1915	0.1937
0.1986	0.1991	0.2010	0.2054	0.2076	0.2116	0.2133	0.2162
0.2171	0.2200	0.2242	0.2275	0.2295	0.2330	0.2361	0.2409
0.2436	0.2471	0.2503	0.2533	0.2591	0.2605	0.2655	0.2711
0.2810	0.2844	0.2889	0.2985	0.3027	0.3065	0.3107	0.3136
0.3198	0.3242	0.3298	0.3311	0.3339	0.3390	0.3424	0.3436
0.3480	0.3491	0.3585	0.3595	0.3622	0.3734	0.3751	0.3833
0.3878	0.3923	0.3930	0.3951	0.4027	0.4052	0.4164	0.4229
0.4252	0.4313	0.4399	0.4467	0.4514	0.4541	0.4567	0.4637
0.4702	0.4753	0.4823	0.4857	0.4970	0.5011	0.5034	0.5043
0.5097	0.5102	0.5143	0.5169	0.5214	0.5231	0.5264	0.5300
0.5330	0.5354	0.5387	0.5417	0.5436	0.5469	0.5495	0.5544

0.5556	0.5584	0.5623	0.5674	0.5689	0.5724	0.5753	0.5799
0.5831	0.5857	0.5889	0.5910	0.5922	0.5940	0.5996	0.6028
0.6036	0.6051	0.6094	0.6123	0.6147	0.6169	0.6196	0.6215
0.6273	0.6296	0.6311	0.6325	0.6337	0.6364	0.6397	0.6467
0.6480	0.6518	0.6542	0.6594	0.6611	0.6629	0.6658	0.6704
0.6745	0.6747	0.6782	0.6840	0.6849	0.6919	0.6932	0.6954
0.7008	0.7062	0.7085	0.7168	0.7173	0.7238	0.7262	0.7286
0.7373	0.7397	0.7412	0.7470	0.7491	0.7543	0.7568	0.7640
0.7651	0.7740	0.7776	0.7836	0.7923	0.7980	0.8001	0.8046
0.8103	0.8132	0.8170	0.8210	0.8246	0.8294	0.8327	0.8360
0.8410	0.8424	0.8469	0.8517	0.8585	0.8598	0.8666	0.8681
0.8742	0.8860	0.8925	0.9004	0.9011	0.9087	0.9160	0.9202
0.9277	0.9311	0.9393	0.9447	0.9504	0.9527	0.9661	0.9689
0.9748	0.9814	0.9866	0.9929	0.9977	1.0034	1.0089	1.0142
1.0163	1.0260	1.0294	1.0414	1.0471	1.0515	1.0549	1.0647
1.0710	1.0729	1.0810	1.0846	1.0875	1.0969	1.1020	1.1069
1.1120	1.1227	1.1271	1.1284	1.1318	1.1425	1.1446	1.1477
1.1583	1.1667	1.1676	1.1748	1.1785	1.1839	1.1883	1.2005
1.2051	1.2121	1.2182	1.2244	1.2262	1.2305	1.2343	1.2371
1.2422	1.2462	1.2506	1.2544	1.2589	1.2614	1.2645	1.2675
1.2712	1.2755	1.2836	1.2907	1.2937	1.2960	1.3012	1.3091
1.3124	1.3190	1.3213	1.3288	1.3318	1.3334	1.3395	1.3397
1.3438	1.3483	1.3529	1.3555	1.3601	1.3652	1.3687	1.3704
1.3808	1.3839	1.3916	1.3939	1.3978	1.3999	1.4025	1.4041
1.4067	1.4107	1.4133	1.4201	1.4263	1.4321	1.4328	1.4397
1.4425	1.4469	1.4541	1.4555	1.4615	1.4636	1.4742	1.4785
1.4883	1.4949	1.4997	1.5046	1.5122	1.5186	1.5204	1.5298
1.5341	1.5383	1.5438	1.5551	1.5661	1.5756	1.5855	1.5879
1.5982	1.5990	1.6145	1.6211	1.6222	1.6334	1.6438	1.6456
1.6592	1.6626	1.6802	1.6847	1.6947	1.7011	1.7158	1.7269
1.7371	1.7485	1.7507	1.7592	1.7642	1.7695	1.7806	1.7914
1.7993	1.8036	1.8127	1.8291	1.8477	1.8487	1.8563	1.8667
1.8850	1.8909	1.9074	1.9158	1.9172	1.9621	1.9854	1.9875
2.0108	2.0519	2.0539	2.0733	2.0820	2.0914	2.0934	2.1013
2.1037	2.1135	2.1438	2.1609	2.1845	2.1909	2.2280	2.2426
2.2457	2.2502	2.2972	2.3353	2.3418	2.3535	2.3626	2.3715
2.3893	2.4109	2.4305	2.4462	2.4552	2.4620	2.4704	2.4832
2.4890	2.5321	2.5333	2.5529	2.5594	2.5632	2.5656	2.5694
2.5736	2.5829	2.5888	2.5945	2.6043	2.6074	2.6103	2.6108
2.6240	2.6451	2.6639	2.6774	2.6807	2.6869	2.6877	2.6893
2.6949	2.6968	2.7009	2.7067	2.7089	2.7145	2.7215	2.7254
2.7305	2.7387	2.7409	2.7496	2.7521	2.7554	2.7573	2.7628
2.7695	2.7748	2.7770	2.7806	2.7867	2.7889	2.7909	2.7949
2.7994	2.8035	2.8086	2.8102	2.8137	2.8164	2.8217	2.8271
2.8282	2.8365	2.8389	2.8425	2.8497	2.8579	2.8593	2.8615
2.8644	2.8657	2.8719	2.8822	2.8862	2.8881	2.8901	2.8987
2.9025	2.9050	2.9109	2.9174	2.9186	2.9306	2.9330	2.9392
2.9487	2.9544	2.9602	2.9645	2.9766	2.9834	2.9868	3.0012
3.0018	3.0061	3.0164	3.0216	3.0284	3.0318	3.0389	3.0428
3.0501	3.0530	3.0606	3.0725	3.0785	3.0811	3.0831	3.0845
3.0874	3.0921	3.0947	3.0993	3.1004	3.1042	3.1076	3.1117
3.1168	3.1248	3.1347	3.1384	3.1438	3.1506	3.1572	3.1629
3.1652	3.1701	3.1731	3.1781	3.1904	3.1920	3.1997	3.2050
3.2179	3.2215	3.2271	3.2324	3.2363	3.2374	3.2467	3.2515
3.2548	3.2607	3.2623	3.2711	3.2744	3.2790	3.2873	3.2915
3.2988	3.3050	3.3085	3.3114	3.3179	3.3221	3.3265	3.3315
3.3345	3.3422	3.3481	3.3488	3.3493	3.3537	3.3598	3.3607
3.3658	3.3732	3.3747	3.3836	3.3894	3.3972	3.4017	3.4099

3.4110 3.4256 3.4327 3.4380 3.4442 3.4473 3.4523 3.4554
3.4572 3.4639 3.4689 3.4728 3.4808 3.4873 3.4901 3.4946
3.5000 3.5082 3.5112 3.5160 3.5217 3.5323 3.5350 3.5376
3.5466 3.5590 3.5671 3.5722 3.5878 3.5928 3.5945 3.6023
3.6097 3.6230 3.6252 3.6275 3.6362 3.6400 3.6461 3.6538
3.6608 3.6658 3.6680 3.6689 3.6761 3.6832 3.6866 3.7011
3.7034 3.7108 3.7159 3.7235 3.7292 3.7365 3.7433 3.7443
3.7515 3.7557 3.7649 3.7684 3.7719 3.7758 3.7843 3.7866
3.7901 3.7951 3.8022 3.8084 3.8101 3.8240 3.8263 3.8295
3.8398 3.8453 3.8592 3.8728 3.8815 3.8883 3.8954 3.9036
3.9055 3.9151 3.9163 3.9227 3.9280 3.9454 3.9573 3.9659
3.9717 3.9752 3.9768 3.9845 3.9891 3.9949 3.9989 4.0017
4.0112 4.0156 4.0251 4.0367 4.0443 4.0638 4.0814 4.0830
4.0863 4.0886 4.0986 4.1030 4.1130 4.1190 4.1368 4.1410
4.1450 4.1641 4.1665 4.1738 4.1831 4.1857 4.1964 4.2115
4.2150 4.2179 4.2281 4.2340 4.2357 4.2408 4.2539 4.2683
4.2771 4.2807 4.2865 4.2934 4.3070 4.3199 4.3221 4.3286
4.3324 4.3363 4.3437 4.3545 4.3574 4.3576 4.3621 4.3674
4.3758 4.3794 4.3850 4.3930 4.3954 4.4124 4.4134 4.4159
4.4230 4.4250 4.4258 4.4307 4.4502 4.4592 4.4631 4.4664
4.4747 4.4824 4.4894 4.4910 4.4989 4.5017 4.5044 4.5150
4.5215 4.5296 4.5468 4.5665 4.5736 4.5799 4.5890 4.5998
4.6088 4.6160 4.6236 4.6339 4.6428 4.6488 4.6516 4.6569
4.6631 4.6671 4.6693 4.6800 4.6863 4.7215 4.7578 4.8124
4.8250 4.8330 4.8524 4.8773 4.8812 4.8843 4.9464 4.9682
4.9736 4.9781 5.0057 5.0102 5.0240 5.0257 5.0395 5.0647
5.0726 5.0845 5.0891 5.2382 5.2424 5.2513 5.2832 5.3058
5.3170 5.3247 5.3934 5.3973 5.4100 5.4159 5.4419 5.4465
5.4569 5.4589 5.5229 5.5758 5.6140 5.6504 5.6546 5.6567
5.6606 5.6746 5.6824 5.6849 5.7020 5.7125 5.7584 5.7604
5.7674 5.9795 5.9894 5.9932 6.3568 6.3582 6.3595 6.4377
6.4387 6.4408 6.6154 6.6174 6.6197 6.6478 6.6493 6.6496
6.6887 6.6903 6.6913 6.7760 6.7774 6.7776 6.8318 6.8331
6.8333 7.0066 7.0108 7.0113 7.1128 7.1137 7.1140 7.4745
7.4756 7.4757 7.7277 7.7299 7.7314 8.1460 9.1457 9.1473
9.1476 9.2439 9.2458 9.2465 9.5447 9.5459 9.5461 9.7625
9.7646 9.7651 10.0715 10.0741 10.0754 14.6680 14.7139 14.7345
24.0969 24.1050 24.1183 24.4093 24.5100 24.5862 24.6430 24.6528
24.6592 24.6730 24.6753 24.6831 24.6843 24.8870 24.8921 24.8979
24.9433 24.9464 24.9534 25.3760 25.3785 25.3874 67.4399 67.4423
67.4430 165.2813
GroundState
Mulliken Net Atomic Charges
Atom Charge (a.u.)
1
P 0.067488
2 F 0.139453
3 F 0.138225
4 F 0.140613
5 C 0.279530
6 H 0.229325
7 H 0.216161
8 C 0.372193
9 H 0.223470
10 H 0.214138
11 C 0.370595
12 H 0.173966
13 H 0.174857

14 C 0.543950
15 H 0.157151
16 H 0.158695
17 H 0.171041
18 C 0.352940
19 C 0.226632
20 C 0.248746
21 C 0.167116
22 H 0.230235
23 C 0.263084
24 H 0.245241
25 C 0.186312
26 H 0.238617
27 C 0.129441
28 H 0.204372
29 C 0.117582
30 H 0.202061
31 C 0.108214
32 H 0.202796
33 C 0.053024
34 C 0.087790
35 C 0.115242
36 C 0.106512
37 H 0.202108
38 C 0.098846
39 H 0.201845
40 C 0.116355
41 H 0.201857
42 C 0.174174
43 H 0.245717
44 C 0.173845
45 H 0.201665
46 C 0.175030
47 H 0.200976
Sum
of atomic charges = 1.000000
Cartesian
Multipole Moments
Charge
(ESU x 10^10)
4.8032
Dipole Moment (Debye)
X 0.1091 Y 0.0844 Z 1.2670
Tot 1.2745
Quadrupole Moments (DebyeAng)
XX 135.3929
XY 0.4582 YY 139.0102
XZ 1.6050
YZ 2.6073 ZZ 134.5140
Traceless Quadrupole Moments (DebyeAng)
QXX 2.7385 QYY 8.1134
QZZ 5.3749
QXY 1.3747 QXZ 4.8151
QYZ 7.8218
Octopole Moments (DebyeAng^
2)
XXX 183.7102
XXY 5.5995 XYY 62.3990

YYY 14.4006 XXZ 52.9889 XYZ 14.2163
YYZ 26.7124 XZZ 65.8808
YZZ 24.1008
ZZZ 125.0078
Traceless Octopole Moments (DebyeAng^
2)
XXX 52.2570 YYY 252.9154 ZZZ 32.7346
XXY 96.2944 XXZ 180.7063 XYY 0.0150
XYZ 213.2447
XZZ 52.2420
YYZ 213.4410
YZZ 349.2098
Hexadecapole Moments (DebyeAng^
3)
XXXX 3071.2982
XXXY 65.3545 XXYY 1374.1435
XYYY 91.0190
YYYY 4778.9406
XXXZ 145.5685
XXYZ 11.3864 XYYZ 9.0606
YYYZ 82.9277
XXZZ 1336.8168
XYZZ 38.7684 YYZZ 1680.6590
XZZZ 202.2652 YZZZ 25.1581
ZZZZ 5614.6466
Traceless Hexadecapole Moments (DebyeAng^
3)
XXXX 2316.1586
XXXY 6272.5446 XXXZ 39.8987
XXYY 6789.4179
XXYZ 155.8613 XXZZ 9105.5765
XYYY 10146.6683
XYYZ 6032.9559
XYZZ 3874.1238
XZZZ 5993.0572 YYYY 3014.9981 YYYZ 5588.2718
YYZZ 3774.4197 YZZZ 5744.1331
ZZZZ 12879.9962
Total
job time: 7498.77s(wall), 29681.75s(cpu)
Sun Jan 8 20:47:50 2023

SPARTAN'20
build 1.1.4 (Dec 1 2021)
Wavefunction Developers:
B.J. Deppmeier, A.J. Driessen, W.J. Hehre, T.S. Hehre,
J.A. Johnson, W.S. Ohlinger, P.E. Klunzinger
Please cite Spartan as:
Spartan'20
Wavefunction Inc.
Irvine CA
QChem
5.1, QChem,
Inc., Pleasanton, CA (2020)
QChem
Developers:
Yihan Shao, Zhengting Gan, E. Epifanovsky, A. T. B. Gilbert, M. Wormit,
J. Kussmann, A. W. Lange, A. Behn, Jia Deng, Xintian Feng, D. Ghosh,
M. Goldey, P. R. Horn, L. D. Jacobson, I. Kaliman, T. Kus, A. Landau, Jie Liu,
E. I. Proynov, R. M. Richard, R. P. Steele, E. J. Sundstrom,
H. L. Woodcock III, P. M. Zimmerman, D. Zuev, B. Alam, B. Albrecht,
E. Alguire, S. A. Baeppler, D. Barton, Z. Benda, Y. A. Bernard,
E. J. Berquist, K. B. Bravaya, H. Burton, K. CarterFenk,
D. Casanova,
ChunMin
Chang, Yunqing Chen, A. Chien, K. D. Closser, M. P. Coons,
S. Coriani, S. Dasgupta, A. L. Dempwolff, M. Diedenhofen, Hainam Do,
R. G. Edgar, PoTung
Fang, S. Faraji, S. Fatehi, Qingguo Feng, J. FossoTande,
J. Gayvert, Qinghui Ge, A. Ghysels, G. Gidofalvi, J. Gomes, J. Gonthier,
A. Gunina, D. Hait, M. W. D. HansonHeine,
P. H. P. Harbach, A. W. Hauser,
M. F. Herbst, J. E. Herr, E. G. Hohenstein, Z. C. Holden, Kerwin Hui,
B. C. Huynh, T.C.
Jagau, Hyunjun Ji, B. Kaduk, K. Khistyaev, Jaehoon Kim,
P. Klunzinger, K. Koh, D. Kosenkov, L. Koulias, T. Kowalczyk, C. M. Krauter,
A. Kunitsa, Ka Un Lao, A. Laurent, K. V. Lawler, Joonho Lee, D. Lefrancois,
S. Lehtola, D. S. Levine, YiPei
Li, YouSheng
Lin, Fenglai Liu, KuanYu
Liu,
E. Livshits, M. Loipersberger, A. Luenser, P. Manohar, E. Mansoor,
S. F. Manzer, ShanPing
Mao, Yuezhi Mao, N. Mardirossian, A. V. Marenich,
T. Markovich, L. A. MartinezMartinez,
S. A. Maurer, N. J. Mayhall,
S. C. McKenzie, J.M.
Mewes, P. Morgante, A. F. Morrison, J. W. Mullinax,
K. Nanda, T. S. NguyenBeck,
R. OlivaresAmaya,
J. A. Parkhill, S. K. Paul,
Zheng Pei, T. M. Perrine, F. Plasser, P. Pokhilko, S. Prager, A. Prociuk,
E. Ramos, B. Rana, D. R. Rehn, F. Rob, M. Scheurer, M. Schneider, N. Sergueev,
S. M. Sharada, S. Sharma, D. W. Small, T. Stauch, C. J. Stein, T. Stein,
YuChuan
Su, S. P. Veccham, A. J. W. Thom, A. Tkatchenko, T. Tsuchimochi,
N. M. Tubman, L. Vogt, M. L. Vidal, O. Vydrov, M. A. Watson, J. Wenzel,
M. de Wergifosse, T. A. Wesolowski, A. White, J. Witte, A. Yamada, Jun Yang,
K. Yao, S. Yeganeh, S. R. Yost, ZhiQiang
You, A. Zech, Igor Ying Zhang,

Xing Zhang, Yan Zhao, Ying Zhu, B. R. Brooks, G. K. L. Chan, C. J. Cramer,
M. S. Gordon, W. J. Hehre, A. Klamt, M. W. Schmidt, C. D. Sherrill,
D. G. Truhlar, A. AspuruGuzik,
R. Baer, A. T. Bell, N. A. Besley,
JengDa
Chai, A. E. DePrince, III, R. A. DiStasio Jr., A. Dreuw,
B. D. Dunietz, T. R. Furlani, ChaoPing
Hsu, Yousung Jung, Jing Kong,
D. S. Lambrecht, WanZhen Liang, C. Ochsenfeld, V. A. Rassolov,
L. V. Slipchenko, J. E. Subotnik, T. Van Voorhis, J. M. Herbert, A. I. Krylov,
P. M. W. Gill, M. HeadGordon,
Contributors to earlier versions of QChem
not listed above:
R. D. Adamson, B. Austin, J. Baker, G. J. O. Beran, K. Brandhorst,
S. T. Brown, E. F. C. Byrd, A. K. Chakraborty, C.L.
Cheng, Siu Hung Chien,
D. M. Chipman, D. L. Crittenden, H. Dachsel, R. J. Doerksen, A. D. Dutoi,
L. FustiMolnar,
W. A. Goddard III, A. GolubevaZadorozhnaya,
S. R. Gwaltney,
G. Hawkins, A. Heyden, S. Hirata, G. Kedziora, F. J. Keil, C. Kelley,
Jihan Kim, R. A. King, R. Z. Khaliullin, P. P. Korambath, W. Kurlancheek,
A. M. Lee, M. S. Lee, S. V. Levchenko, Ching Yeh Lin, D. Liotard,
R. C. Lochan, I. Lotan, P. E. Maslen, N. Nair, D. P. O'Neill, D. Neuhauser,
E. Neuscamman, C. M. Oana, R. Olson, B. Peters, R. Peverati, P. A. Pieniazek,
Y. M. Rhee, J. Ritchie, M. A. Rohrdanz, E. Rosta, N. J. Russ,
H. F. Schaefer III, N. E. Schultz, N. Shenvi, A. C. Simmonett, A. Sodt,
D. Stuck, K. S. Thanthiriwatte, V. Vanovschi, Tao Wang, A. Warshel,
C. F. Williams, Q. Wu, X. Xu, W. Zhang,
Please cite QChem
as follows :
Y.Shao et al., Mol.Phys. 113, 184 215
(2015)
DOI : 10.1080/00268976.2014.952696
Parts of QChem
use Armadillo 8.300.2 (tropical Shenanigans).
http://arma.sourceforge.net/
Wavefunction Inc. Sales: sales@wavefun.com
Irvine CA Support: support@wavefun.com
Web: www.wavefun.com
Copyright © 1995 2021
Wavefunction
Version of QChem
Parts of QChem
use Armadillo 8.300.2 (Tropical Shenanigans).
http://arma.sourceforge.net/
QChem
begins on Thu Jan 19 03:17:50 2023
Scratch files written to
C:/Users/PATRIC~1/AppData/Local/Temp/WF8AD97F3A18127AB3//scratch///
Processing $rem in system registry
... MEM_TOTAL 20000 # MB
Processing $rem in C:/Program
Files/Wavefunction/Spartan20v114/P4e//../auxdir/config/preferences:
(site specific preferences)
... THRESH 9
... SMALL_PROD_XCMAT 9
... BASIS_LIN_DEP_THRESH 5

```
... SCF_ALGORITHM DIIS_GDM
... MAXSCF 250
... MAXDIIS 45
... THRESHDIIS 1
(i.e. don't switch on deltaE)
... ECP_FIT TRUE (Convert deprecated ECP files)
... GUI GUI_SPARTAN
... TERSE_OUTPUT TRUE !turn on spartan printing
... SCF_CONVERGENCE 7
... CCMAN2 FALSE (qc4.3)
... SYMMETRY FALSE ! turn of symmetry for spartan16
... SYM_IGNORE TRUE ! ..use FORCESYMMETRY to override
... GEOM_OPT_TOL_GRADIENT 700 ! loosen tolernaces for organic geometries
... GEOM_OPT_TOL_DISPLACEMENT 1400 ! was 1200 = .0012
... GEOM_OPT_TOL_ENERGY 2000 ! was 100 = .000 001
... GEN_SCFMAN FALSE
Processing $rem in input file
... JOBTYPE SP
... SCF_CONVERGENCE 7 (sp default for single point energy)
... METHOD WB97MV
... xc_grid 75000302 (75,302)
... BASIS 6311+
G(2DF,2P)
... THRESH 12 #diffuse default
... MAXSCF 350 #diffuse default
... VARTHRESH 2 (default DFT)
... INCDFT TRUE (default DFT)
... GUI GUI_SPARTAN
... TERSE_OUTPUT TRUE
NAlpha2: 218
NElect 218
Mult 1
Warning: disabling incdft.
Checking the input file for inconsistencies... ...done.
User
input:
$
comment
4MeO
TPP Cation
$end
$molecule
1 1
15 0.21515607151
0.15227187026
0.10155344798
8 3.1820598565 3.59027592 3.1779497359
8 5.2853191637
2.4910950598 1.4360258376
8 2.5099474659 1.6770048555
4.9302903507
6 0.51574013011
1.5964243019
1.1600302585
1 1.1026121891
1.2343510854
2.0110155315
1 1.1472218545
```

2.3008780153
0.60808180902
6 0.77147600509 2.2695736612
1.6494217929
1 1.4080996541 2.5325408823
0.79675204882
1 1.3408010402 1.5638373612
2.2633489315
6 0.47091688034 3.5259293235
2.4667791724
1 0.18151359357
3.2626631741
3.3068766219
1 0.091033927095
4.2347507316
1.8477262779
6 1.7429951592 4.1880404632
2.9873175701
1 2.3991328472 4.4838007225
2.1640491251
1 1.5129374194 5.0834784829
3.5667516497
1 2.3035667307 3.5081281057
3.6344444024
6 0.79407006212 1.0389867194 0.978341472
6 2.186686201 0.86102372051 1.065062716
1 2.6696132235 0.043562465374 0.5396925804
6 2.9528363522 1.7310053464 1.8075011931
1 4.0269994481 1.6160605465 1.8822032418
6 2.3471945714 2.8001795567 2.4869000953
6 0.96417123802 2.983916037 2.4092391258
1 0.47860609249 3.8033171998 2.9207087086
6 0.1984556875 2.1018964854 1.6568549285
1 0.87380485836
2.2562929111 1.596854349
6 2.6360515591 4.6970453385 3.8934886678
1 2.1450816384 5.3973709678 3.2126518684
1 3.4823424645 5.1848549166 4.3687595597
1 1.9323752256 4.3576422224 4.6582118211
6 1.7852411446
0.60494505027 0.31636545682
6 1.785787183
1.7327346221 1.1559007144
1 0.85153661649
2.1373335428 1.5324579439
6 2.9693769633
2.3392739064 1.5072435945
1 2.9902356097
3.2097215989 2.1509405162
6 4.1897121601
1.8291165496 1.0343024549
6 4.2011132443
0.706037728 0.20513861191
1 5.128630228
0.29435135914 0.16772303826
6 2.9981155993
0.10323316886 0.14832206794
1 3.0309102081

0.7671619232
0.79354961588
6 6.5604039452
2.0204542257 1.0039711871
1 7.2873666491
2.6968935843 1.4444513111
1 6.6408525892
2.056291358 0.085761564585
1 6.7420653117
1.0039220659 1.3628236375
6 0.65710446394 0.66904397773
1.3767958834
6 1.456963226 0.24532674532 2.0821145583
1 1.6198029866 1.245689513 1.6946691163
6 2.0581174951 0.12260589011
3.2660296208
1 2.6808291631 0.56760442284 3.8214063985
6 1.8770968161 1.4162819821
3.7766244702
6 1.0812196519 2.3336514919
3.0842996826
1 0.92855734917 3.3365192124
3.4581285308
6 0.47436828025 1.9506314888
1.8957887944
1 0.14439023413
2.6765344136
1.3789590384
6 2.3726301147 2.9757585556
5.5039810043
1 1.3277784194 3.1864469009
5.7469135252
1 2.7661959085 3.7425914037
4.8314039868
1 2.9610587781 2.9565545771
6.4168987633
$end
$rem
JOBTYPE SP
SCF_CONVERGENCE 7 (sp default for single point energy)
METHOD WB97MV
xc_grid 75000302 (75,302)
BASIS 6311+
G(2DF,2P)
THRESH 12 #diffuse default
MAXSCF 350 #diffuse default
VARTHRESH 2 (default DFT)
INCDFT TRUE (default DFT)
GUI GUI_SPARTAN
TERSE_OUTPUT TRUE
$end
S
tandard Nuclear Orientation (Angstroms)
I Atom X Y Z
1
P 0.2151560715
0.1522718703
0.1015534480

2 O 3.1820598565 3.5902759200 3.1779497359
3 O 5.2853191637
2.4910950598 1.4360258376
4 O 2.5099474659 1.6770048555
4.9302903507
5 C 0.5157401301
1.5964243019
1.1600302585
6 H 1.1026121891
1.2343510854
2.0110155315
7 H 1.1472218545
2.3008780153
0.6080818090
8 C 0.7714760051 2.2695736612
1.6494217929
9 H 1.4080996541 2.5325408823
0.7967520488
10 H 1.3408010402 1.5638373612
2.2633489315
11 C 0.4709168803 3.5259293235
2.4667791724
12 H 0.1815135936
3.2626631741
3.3068766219
13 H 0.0910339271
4.2347507316
1.8477262779
14 C 1.7429951592 4.1880404632
2.9873175701
15 H 2.3991328472 4.4838007225
2.1640491251
16 H 1.5129374194 5.0834784829
3.5667516497
17 H 2.3035667307 3.5081281057
3.6344444024
18 C 0.7940700621 1.0389867194 0.9783414720
19 C 2.1866862010 0.8610237205 1.0650627160
20 H 2.6696132235 0.0435624654 0.5396925804
21 C 2.9528363522 1.7310053464 1.8075011931
22 H 4.0269994481 1.6160605465 1.8822032418
23 C 2.3471945714 2.8001795567 2.4869000953
24 C 0.9641712380 2.9839160370 2.4092391258
25 H 0.4786060925 3.8033171998 2.9207087086
26 C 0.1984556875 2.1018964854 1.6568549285
27 H 0.8738048584
2.2562929111 1.5968543490
28 C 2.6360515591 4.6970453385 3.8934886678
29 H 2.1450816384 5.3973709678 3.2126518684
30 H 3.4823424645 5.1848549166 4.3687595597
31 H 1.9323752256 4.3576422224 4.6582118211
32 C 1.7852411446
0.6049450503 0.3163654568
33 C 1.7857871830
1.7327346221 1.1559007144
34 H 0.8515366165
2.1373335428 1.5324579439
35 C 2.9693769633

2.3392739064 1.5072435945
36 H 2.9902356097
3.2097215989 2.1509405162
37 C 4.1897121601
1.8291165496 1.0343024549
38 C 4.2011132443
0.7060377280 0.2051386119
39 H 5.1286302280
0.2943513591 0.1677230383
40 C 2.9981155993
0.1032331689 0.1483220679
41 H 3.0309102081
0.7671619232
0.7935496159
42 C 6.5604039452
2.0204542257 1.0039711871
43 H 7.2873666491
2.6968935843 1.4444513111
44 H 6.6408525892
2.0562913580 0.0857615646
45 H 6.7420653117
1.0039220659 1.3628236375
46 C 0.6571044639 0.6690439777
1.3767958834
47 C 1.4569632260 0.2453267453 2.0821145583
48 H 1.6198029866 1.2456895130 1.6946691163
49 C 2.0581174951 0.1226058901
3.2660296208
50 H 2.6808291631 0.5676044228 3.8214063985
51 C 1.8770968161 1.4162819821
3.7766244702
52 C 1.0812196519 2.3336514919
3.0842996826
53 H 0.9285573492 3.3365192124
3.4581285308
54 C 0.4743682803 1.9506314888
1.8957887944
55 H 0.1443902341
2.6765344136
1.3789590384
56 C 2.3726301147 2.9757585556
5.5039810043
57 H 1.3277784194 3.1864469009
5.7469135252
58 H 2.7661959085 3.7425914037
4.8314039868
59 H 2.9610587781 2.9565545771
6.4168987633
Nuclear
Repulsion Energy = 2896.92587428 hartrees
There are 109 alpha and 109 beta electrons
Requested basis set is 6311+
G(2df,2p)
There are 389 shells and 1264 basis functions
Total QAlloc Memory Limit 20000 MB
MegaArray
Size 188 MB
MEM_STATIC part 192 MB

```
.. (5.2.P)
Entering
fldman on Thu Jan 19 03:17:50 2023 A
cutoff of 1.0D12
yielded 42531 shell pairs
There are 456872 function pairs ( 554033 Cartesian)
Smallest overlap matrix eigenvalue = 1.85E07
Linear dependence detected in AO basis
Tighter screening thresholds may be required for diffuse basis sets
Use S2THRESH > 12 and THRESH = 14 in case of SCF convergence issues
Number of orthogonalized atomic orbitals = 1247
Maximum deviation from orthogonality = 2.109E11
Scale SEOQF with 1.000000e02/
1.000000e02/
1.000000e02
Standard Electronic Orientation quadrupole field applied
Nucleusfield
energy = 0.0000000005
hartrees
Entering
gesman on Thu Jan 19 03:17:54 2023 Guess
from superposition of atomic densities
Warning: Energy on first SCF cycle will be nonvariational
SAD guess density has 215.164804 electrons
Entering
scfman on Thu Jan 19 03:17:55 2023 Longrange
K will be added via erf
Coulomb attenuation parameter = 0.3 bohr**(1)
A restricted hybrid HFDFT
SCF calculation will be
performed using Pulay DIIS + Geometric Direct Minimization
Exchange: 0.1500 HartreeFock
+ 1.0000 wB97MV
+ LRHF
Correlation: 1.0000 wB97MV
Using EulerMaclaurinLebedev
(75,302) quadrature formula
Nonlocal Correlation: VV10 with C = 0.0100 and b = 6.00 and scale = 1.00000
Grid used for NLC: SG1
standard quadrature
SCF converges when RMS gradient is below 1.0E07
Exchange: 0.1500 HartreeFock
+ 1.0000 wB97MV
+ LRHF
Correlation: 1.0000 wB97MV
Using EulerMaclaurinLebedev
(75,302) quadrature formula
Nonlocal Correlation: VV10 with C = 0.0100 and b = 6.00 and scale = 1.00000
Grid used for NLC: SG1
standard quadrature
using 4 threads for integral computing
OpenMP
Integral computing Module
Release: version 1.0, May 2013, QChem
Inc. Pittsburgh
using
4 threads for integral computing
OpenMP
```

Integral computing Module
Release: version 1.0, May 2013, QChem
Inc. Pittsburgh
OpenMP
BLAS3 based DFT computing Module
Release: version 1.0, May 2013, QChem
Inc. Pittsburgh
OpenMP
BLAS3 based DFT computing Module
Release: version 1.0, May 2013, QChem
Inc. Pittsburgh
OpenMP
BLAS3 based DFT computing Module
Release: version 1.0, May 2013, QChem
Inc. Pittsburgh
Cycle
Energy DIIS Error
1
1538.2133704873
4.04E02
2 1395.6735610542
2.65E02
3 835.1454165554
3.60E02
4 1306.9108970464
2.53E02
5 1363.7187163653
1.92E02
6 1190.0846830752
2.63E02
7 1527.5009300368
5.64E03
8 1504.5401671469
1.13E02
9 1536.5504776199
2.15E03
10 1537.1690066819
1.13E03
11 1537.4091137634
3.26E04
12 1537.4296215864
1.58E04
13 1537.4356422481
6.28E05
14 1537.4368780916
1.92E05
15 1537.4370168442
9.40E06
16 1537.4370561987
4.09E06
17 1537.4370687283
2.12E06
18 1537.4370721788
7.78E07
19 1537.4370725966
3.18E07
20 1537.4370726409
1.45E07

21 1537.4370726492
5.43E08
Convergence criterion met
SCF
time: CPU 35210.97 s wall 8874.89 s
SCF energy in the final basis set = 1537.43707265
Total energy in the final basis set = 1537.43707265
Entering
anlman on Thu Jan 19 05:45:51 2023 Orbital
Energies (a.u.)
Alpha
MOs
Occupied
77.5576
19.4703
19.4701
19.4699
10.5269
10.5268
10.5267
10.4851
10.4848
10.4846
10.4825
10.4790
10.4787
10.4762
10.4756
10.4755
10.4746
10.4739
10.4738
10.4736
10.4589
10.4584
10.4573
10.4564
10.4555
10.4546
10.4545
10.4364
10.4137
6.9070
5.0191
5.0189
5.0188
1.3078
1.3076
1.3075
1.1182
1.1018
1.1005
1.0566
1.0218
0.9985
0.9983
0.9958
0.9897

0.9863
0.9698
0.9366
0.9242
0.9228
0.8857
0.8638
0.8519
0.8427
0.8359
0.8211
0.8193
0.8035
0.7930
0.7615
0.7594
0.7487
0.7201
0.7156
0.7121
0.7031
0.6960
0.6896
0.6832
0.6828
0.6827
0.6724
0.6708
0.6650
0.6601
0.6498
0.6467
0.6457
0.6357
0.6325
0.6252
0.6236
0.6199
0.6124
0.6085
0.6018
0.6006
0.5825
0.5781
0.5774
0.5733
0.5692
0.5616
0.5593
0.5447
0.5432
0.5383
0.5352
0.5345
0.5343
0.5325
0.5243
0.5211

0.4692
0.4684
0.4619
0.4364
0.4279
0.4217
Virtual
0.0792
0.0718
0.0662
0.0631
0.0599
0.0571
0.0525
0.0500
0.0474
0.0407
0.0384
0.0345
0.0284
0.0272
0.0261
0.0247
0.0197
0.0143
0.0126
0.0114
0.0061
0.0050
0.0026
0.0003

0.0040	0.0073	0.0103	0.0111	0.0131	0.0191	0.0210	0.0243
0.0266	0.0284	0.0318	0.0328	0.0372	0.0379	0.0427	0.0439
0.0466	0.0514	0.0544	0.0558	0.0580	0.0605	0.0641	0.0647
0.0682	0.0709	0.0719	0.0750	0.0760	0.0792	0.0819	0.0832
0.0886	0.0922	0.0945	0.0987	0.1002	0.1018	0.1060	0.1094
0.1105	0.1125	0.1160	0.1186	0.1200	0.1215	0.1224	0.1260
0.1294	0.1306	0.1318	0.1350	0.1371	0.1414	0.1434	0.1443
0.1495	0.1503	0.1520	0.1554	0.1568	0.1592	0.1614	0.1643
0.1649	0.1680	0.1706	0.1724	0.1749	0.1762	0.1787	0.1810
0.1825	0.1854	0.1884	0.1908	0.1944	0.1956	0.1963	0.1982
0.1994	0.2030	0.2050	0.2053	0.2082	0.2113	0.2130	0.2137
0.2179	0.2202	0.2233	0.2244	0.2286	0.2304	0.2317	0.2318
0.2351	0.2377	0.2395	0.2441	0.2458	0.2470	0.2527	0.2550
0.2567	0.2594	0.2638	0.2669	0.2694	0.2725	0.2745	0.2785
0.2804	0.2813	0.2851	0.2857	0.2867	0.2910	0.2936	0.2982
0.3009	0.3080	0.3090	0.3147	0.3173	0.3264	0.3265	0.3330
0.3360	0.3381	0.3411	0.3435	0.3500	0.3526	0.3548	0.3605
0.3731	0.3746	0.3801	0.3845	0.3874	0.3906	0.3990	0.4072
0.4126	0.4178	0.4201	0.4218	0.4253	0.4325	0.4422	0.4445
0.4471	0.4512	0.4526	0.4568	0.4599	0.4611	0.4709	0.4760
0.4792	0.4815	0.4896	0.4927	0.4934	0.5013	0.5017	0.5032
0.5078	0.5092	0.5151	0.5168	0.5188	0.5197	0.5208	0.5268
0.5305	0.5317	0.5328	0.5400	0.5424	0.5444	0.5454	0.5494
0.5505	0.5528	0.5563	0.5582	0.5659	0.5671	0.5709	0.5711
0.5763	0.5776	0.5799	0.5817	0.5835	0.5853	0.5871	0.5925
0.5937	0.5956	0.5990	0.6013	0.6041	0.6071	0.6119	0.6129
0.6156	0.6161	0.6199	0.6229	0.6245	0.6266	0.6296	0.6347

0.6365	0.6378	0.6415	0.6428	0.6438	0.6448	0.6478	0.6495
0.6521	0.6559	0.6586	0.6602	0.6629	0.6676	0.6683	0.6703
0.6729	0.6770	0.6785	0.6817	0.6832	0.6861	0.6879	0.6921
0.6964	0.6998	0.7034	0.7112	0.7117	0.7177	0.7202	0.7216
0.7274	0.7301	0.7318	0.7340	0.7426	0.7449	0.7466	0.7508
0.7516	0.7552	0.7601	0.7632	0.7646	0.7667	0.7717	0.7754
0.7782	0.7810	0.7890	0.7916	0.7974	0.7993	0.8017	0.8084
0.8123	0.8135	0.8174	0.8245	0.8274	0.8319	0.8360	0.8391
0.8415	0.8440	0.8476	0.8499	0.8577	0.8607	0.8658	0.8679
0.8699	0.8716	0.8765	0.8786	0.8837	0.8868	0.8895	0.8896
0.8962	0.9006	0.9042	0.9115	0.9133	0.9247	0.9306	0.9329
0.9373	0.9423	0.9503	0.9521	0.9553	0.9612	0.9648	0.9688
0.9751	0.9824	0.9857	0.9921	0.9939	0.9975	1.0056	1.0074
1.0097	1.0119	1.0180	1.0209	1.0299	1.0371	1.0393	1.0429
1.0471	1.0485	1.0514	1.0585	1.0624	1.0683	1.0717	1.0770
1.0814	1.0855	1.0872	1.0892	1.0934	1.0975	1.1000	1.1020
1.1075	1.1132	1.1173	1.1189	1.1231	1.1246	1.1296	1.1312
1.1339	1.1398	1.1416	1.1468	1.1524	1.1562	1.1629	1.1642
1.1693	1.1746	1.1778	1.1800	1.1873	1.1897	1.1909	1.1936
1.1976	1.1994	1.2026	1.2107	1.2190	1.2258	1.2304	1.2335
1.2377	1.2465	1.2509	1.2532	1.2563	1.2594	1.2626	1.2665
1.2721	1.2734	1.2817	1.2852	1.2870	1.2928	1.2948	1.2971
1.3039	1.3066	1.3148	1.3155	1.3230	1.3243	1.3276	1.3296
1.3311	1.3345	1.3385	1.3433	1.3498	1.3509	1.3534	1.3579
1.3620	1.3656	1.3703	1.3734	1.3748	1.3792	1.3813	1.3858
1.3875	1.3968	1.3976	1.4004	1.4024	1.4075	1.4153	1.4163
1.4184	1.4192	1.4226	1.4265	1.4314	1.4348	1.4363	1.4433
1.4459	1.4490	1.4546	1.4594	1.4618	1.4695	1.4712	1.4752
1.4804	1.4868	1.4880	1.4951	1.4976	1.5047	1.5112	1.5161
1.5217	1.5277	1.5326	1.5374	1.5484	1.5504	1.5540	1.5569
1.5618	1.5624	1.5755	1.5806	1.5846	1.5935	1.5961	1.6010
1.6088	1.6126	1.6230	1.6277	1.6336	1.6386	1.6475	1.6489
1.6581	1.6676	1.6785	1.6846	1.6899	1.6971	1.7064	1.7095
1.7151	1.7216	1.7308	1.7370	1.7499	1.7570	1.7691	1.7788
1.7820	1.7986	1.8039	1.8048	1.8215	1.8271	1.8395	1.8493
1.8754	1.8841	1.8894	1.8978	1.9057	1.9100	1.9409	1.9440
1.9446	1.9582	1.9601	1.9675	1.9883	1.9952	2.0028	2.0154
2.0203	2.0210	2.0258	2.0508	2.0591	2.0826	2.0926	2.1191
2.1251	2.1901	2.2186	2.2290	2.2480	2.2747	2.3017	2.3117
2.3220	2.3373	2.3436	2.3481	2.3489	2.3497	2.3695	2.3741
2.3856	2.3924	2.4064	2.4352	2.4588	2.4656	2.4689	2.4713
2.4757	2.4822	2.4869	2.4952	2.5063	2.5089	2.5390	2.5527
2.5567	2.5591	2.5713	2.5783	2.5862	2.5888	2.5895	2.5979
2.6019	2.6147	2.6240	2.6363	2.6401	2.6608	2.6653	2.6852
2.6935	2.7015	2.7094	2.7128	2.7160	2.7197	2.7227	2.7327
2.7359	2.7415	2.7471	2.7541	2.7597	2.7632	2.7694	2.7735
2.7759	2.7804	2.7888	2.7954	2.7969	2.7986	2.8054	2.8074
2.8125	2.8145	2.8229	2.8253	2.8283	2.8312	2.8365	2.8415
2.8438	2.8453	2.8507	2.8526	2.8596	2.8611	2.8619	2.8651
2.8676	2.8707	2.8711	2.8733	2.8765	2.8815	2.8869	2.8908
2.8996	2.9002	2.9016	2.9063	2.9100	2.9146	2.9174	2.9197
2.9226	2.9257	2.9282	2.9316	2.9345	2.9389	2.9457	2.9496
2.9555	2.9635	2.9668	2.9683	2.9690	2.9778	2.9839	2.9889
2.9903	2.9949	3.0015	3.0055	3.0170	3.0242	3.0326	3.0374
3.0406	3.0450	3.0501	3.0559	3.0578	3.0621	3.0660	3.0679
3.0702	3.0732	3.0760	3.0829	3.0892	3.0899	3.0946	3.1011
3.1066	3.1161	3.1194	3.1227	3.1248	3.1284	3.1308	3.1391
3.1421	3.1446	3.1497	3.1543	3.1548	3.1612	3.1653	3.1684

3.1715 3.1762 3.1774 3.1833 3.1848 3.1918 3.1927 3.1956
3.1991 3.2019 3.2061 3.2111 3.2175 3.2287 3.2298 3.2354
3.2369 3.2421 3.2509 3.2543 3.2569 3.2616 3.2716 3.2745
3.2772 3.2832 3.2859 3.2925 3.2965 3.3008 3.3057 3.3066
3.3156 3.3194 3.3300 3.3322 3.3350 3.3387 3.3423 3.3482
3.3501 3.3549 3.3569 3.3601 3.3643 3.3675 3.3720 3.3753
3.3791 3.3849 3.3859 3.3916 3.3941 3.4001 3.4038 3.4092
3.4138 3.4191 3.4214 3.4240 3.4254 3.4355 3.4364 3.4419
3.4509 3.4561 3.4578 3.4610 3.4678 3.4739 3.4792 3.4868
3.4931 3.4974 3.5055 3.5095 3.5130 3.5152 3.5219 3.5253
3.5299 3.5336 3.5363 3.5448 3.5512 3.5536 3.5577 3.5639
3.5673 3.5725 3.5777 3.5810 3.5844 3.5899 3.5931 3.5980
3.6053 3.6079 3.6136 3.6203 3.6306 3.6326 3.6381 3.6416
3.6430 3.6483 3.6542 3.6588 3.6701 3.6733 3.6809 3.6858
3.6937 3.7005 3.7011 3.7032 3.7158 3.7180 3.7272 3.7306
3.7326 3.7357 3.7428 3.7507 3.7592 3.7684 3.7715 3.7749
3.7762 3.7801 3.7892 3.7916 3.7976 3.8025 3.8067 3.8096
3.8146 3.8179 3.8217 3.8264 3.8318 3.8394 3.8430 3.8446
3.8490 3.8516 3.8575 3.8616 3.8655 3.8679 3.8736 3.8762
3.8824 3.8979 3.9034 3.9079 3.9119 3.9270 3.9285 3.9310
3.9389 3.9465 3.9534 3.9571 3.9671 3.9799 3.9877 3.9924
3.9948 4.0061 4.0106 4.0156 4.0198 4.0219 4.0268 4.0319
4.0457 4.0527 4.0676 4.0688 4.0698 4.0781 4.0848 4.0885
4.0950 4.1030 4.1076 4.1133 4.1148 4.1199 4.1290 4.1318
4.1397 4.1429 4.1482 4.1589 4.1729 4.1743 4.1812 4.1873
4.1953 4.2004 4.2033 4.2103 4.2138 4.2197 4.2306 4.2351
4.2392 4.2465 4.2582 4.2666 4.2765 4.2804 4.2841 4.2846
4.2873 4.2933 4.2967 4.2990 4.3137 4.3200 4.3248 4.3305
4.3328 4.3374 4.3386 4.3436 4.3506 4.3576 4.3633 4.3661
4.3711 4.3734 4.3793 4.3817 4.3858 4.3865 4.3905 4.3996
4.4014 4.4085 4.4094 4.4148 4.4163 4.4226 4.4329 4.4372
4.4386 4.4497 4.4540 4.4616 4.4654 4.4707 4.4729 4.4755
4.4796 4.4812 4.4879 4.4899 4.5004 4.5057 4.5072 4.5146
4.5158 4.5236 4.5280 4.5363 4.5458 4.5526 4.5723 4.5823
4.5957 4.6075 4.6170 4.6199 4.6232 4.6386 4.6533 4.6589
4.6641 4.6681 4.6745 4.6845 4.6900 4.6930 4.7069 4.7184
4.7296 4.7465 4.7923 4.8281 4.8420 4.8551 4.8722 4.8775
4.8850 4.8939 4.9288 4.9425 4.9482 5.0064 5.0091 5.0125
5.0187 5.0276 5.0321 5.0571 5.0602 5.0627 5.0688 5.0976
5.1054 5.1169 5.1655 5.1666 5.1686 5.2291 5.2451 5.2457
5.2460 5.2549 5.2620 5.2944 5.3105 5.3145 5.3253 5.3508
5.3564 5.3681 5.4152 5.4192 5.4248 5.4517 5.4568 5.4689
5.4748 5.4768 5.5407 5.5593 5.5597 5.5599 5.5776 5.5852
5.5877 5.5902 5.6154 5.6248 5.6301 5.6404 5.6688 5.6832
5.6871 5.6927 5.6930 5.7030 5.7033 5.7053 5.7112 5.7763
5.7765 5.7767 5.7962 5.8010 5.8047 5.8204 5.8230 5.8294
5.9535 5.9540 5.9545 6.0291 6.0377 6.0450 6.3687 6.3701
6.3730 6.5451 6.5474 6.5478 6.6508 6.6513 6.6535 7.0064
7.0074 7.0088 7.1339 7.1343 7.1348 7.3240 7.3244 7.3249
7.3323 7.3324 7.3327 7.8105 7.8144 7.8154 7.8828 7.8842
7.8850 8.2067 14.6341 14.7653 14.8008 24.1729 24.2081 24.2233
24.4388 24.5335 24.6011 24.6234 24.6236 24.6244 24.6596 24.6843
24.6929 24.7086 24.7445 24.7511 24.7675 24.9313 24.9340 24.9371
24.9916 24.9942 25.0023 25.4117 25.4204 25.4247 50.6170 50.6181
50.6191 165.3525
GroundState
Mulliken Net Atomic Charges
Atom Charge (a.u.)

1
P 0.185674
2 O 0.124713
3 O 0.121334
4 O 0.126791
5 C 0.498801
6 H 0.230536
7 H 0.247738
8 C 0.420243
9 H 0.218355
10 H 0.231693
11 C 0.359939
12 H 0.172050
13 H 0.169329
14 C 0.576920
15 H 0.155773
16 H 0.168160
17 H 0.158679
18 C 0.277656
19 C 0.195850
20 H 0.250619
21 C 0.223243
22 H 0.195875
23 C 0.015134
24 C 0.153539
25 H 0.194031
26 C 0.226958
27 H 0.234711
28 C 0.342450
29 H 0.172519
30 H 0.171452
31 H 0.172260
32 C 0.259361
33 C 0.147809
34 H 0.205778
35 C 0.227372
36 H 0.194307
37 C 0.037931
38 C 0.157069
39 H 0.193016
40 C 0.176109
41 H 0.214174
42 C 0.350102
43 H 0.172433
44 H 0.171820
45 H 0.170955
46 C 0.371960
47 C 0.239788
48 H 0.237711
49 C 0.190119
50 H 0.195316
51 C 0.056340
52 C 0.184834
53 H 0.191533
54 C 0.172740
55 H 0.194575
56 C 0.339807
57 H 0.171455

58 H 0.171905
59 H 0.170928
Sum
of atomic charges = 1.000000
Cartesian
Multipole Moments
Charge
(ESU x 10^10)
4.8032
Dipole Moment (Debye)
X 2.4281
Y 0.0238
Z 0.1146
Tot 2.4309
Quadrupole Moments (DebyeAng)
XX 129.1728
XY 5.3809
YY 132.4396
XZ 1.5903
YZ 26.0508 ZZ 138.5952
Traceless Quadrupole Moments (DebyeAng)
QXX 12.6893 QYY 2.8887 QZZ 15.5780
QXY 16.1428
QXZ 4.7709
QYZ 78.1524
Octopole Moments (DebyeAng^
2)
XXX 261.4932
XXY 21.8186 XYY 55.8061
YYY 75.8898
XXZ 6.5584 XYZ 71.7403
YYZ 64.2651 XZZ 84.4481 YZZ 67.3891
ZZZ 19.0677
Traceless Octopole Moments (DebyeAng^
2)
XXX 2831.2470
YYY 45.2039
ZZZ 523.0054
XXY 691.6594 XXZ 171.2973
XYY 1200.8089
XYZ 1076.1051 XZZ 1630.4381 YYZ 694.3028
YZZ 646.4556
Hexadecapole Moments (DebyeAng^
3)
XXXX 5425.6937
XXXY 220.6380 XXYY 1982.4056
XYYY 780.6380 YYYY 5079.8944
XXXZ 231.0163
XXYZ 223.5414
XYYZ 41.7965
YYYZ 375.8425
XXZZ 2159.4337
XYZZ 165.1118 YYZZ 1296.7175
XZZZ 628.5493
YZZZ 187.3686
ZZZZ 5624.1591
Traceless Hexadecapole Moments (DebyeAng^
3)

```
XXXX 48318.3781 XXXY 29320.4640
XXXZ 16304.5829
XXYY 20274.9099
XXYZ 11676.0877
XXZZ 28043.4682
XYYY 29479.5384 XYYZ 9131.7978 XYZZ 159.0744
XZZZ 25436.3807
YYYY 24139.0893
YYYZ 4076.1883
YYZZ 44413.9992 YZZZ 15752.2760 ZZZZ 16370.5310
Total
job time: 8883.75s(wall), 35221.98s(cpu)
Thu Jan 19 05:45:54 2023
```

SPARTAN'20
build 1.1.4 (Dec 1 2021)
Wavefunction Developers:
B.J. Deppmeier, A.J. Driessen, W.J. Hehre, T.S. Hehre,
J.A. Johnson, W.S. Ohlinger, P.E. Klunzinger
Please cite Spartan as:
Spartan'20
Wavefunction Inc.
Irvine CA
QChem
5.1, QChem,
Inc., Pleasanton, CA (2020)
QChem
Developers:
Yihan Shao, Zhengting Gan, E. Epifanovsky, A. T. B. Gilbert, M. Wormit,
J. Kussmann, A. W. Lange, A. Behn, Jia Deng, Xintian Feng, D. Ghosh,
M. Goldey, P. R. Horn, L. D. Jacobson, I. Kaliman, T. Kus, A. Landau, Jie Liu,
E. I. Proynov, R. M. Richard, R. P. Steele, E. J. Sundstrom,
H. L. Woodcock III, P. M. Zimmerman, D. Zuev, B. Alam, B. Albrecht,
E. Alguire, S. A. Baeppler, D. Barton, Z. Benda, Y. A. Bernard,
E. J. Berquist, K. B. Bravaya, H. Burton, K. CarterFenk,
D. Casanova,
ChunMin
Chang, Yunqing Chen, A. Chien, K. D. Closser, M. P. Coons,
S. Coriani, S. Dasgupta, A. L. Dempwolff, M. Diedenhofen, Hainam Do,
R. G. Edgar, PoTung
Fang, S. Faraji, S. Fatehi, Qingguo Feng, J. FossoTande,
J. Gayvert, Qinghui Ge, A. Ghysels, G. Gidofalvi, J. Gomes, J. Gonthier,
A. Gunina, D. Hait, M. W. D. HansonHeine,
P. H. P. Harbach, A. W. Hauser,
M. F. Herbst, J. E. Herr, E. G. Hohenstein, Z. C. Holden, Kerwin Hui,
B. C. Huynh, T.C.
Jagau, Hyunjun Ji, B. Kaduk, K. Khistyaev, Jaehoon Kim,
P. Klunzinger, K. Koh, D. Kosenkov, L. Koulias, T. Kowalczyk, C. M. Krauter,
A. Kunitsa, Ka Un Lao, A. Laurent, K. V. Lawler, Joonho Lee, D. Lefrancois,
S. Lehtola, D. S. Levine, YiPei
Li, YouSheng
Lin, Fenglai Liu, KuanYu
Liu,
E. Livshits, M. Loipersberger, A. Luenser, P. Manohar, E. Mansoor,
S. F. Manzer, ShanPing
Mao, Yuezhi Mao, N. Mardirossian, A. V. Marenich,
T. Markovich, L. A. MartinezMartinez,
S. A. Maurer, N. J. Mayhall,
S. C. McKenzie, J.M.
Mewes, P. Morgante, A. F. Morrison, J. W. Mullinax,
K. Nanda, T. S. NguyenBeck,
R. OlivaresAmaya,
J. A. Parkhill, S. K. Paul,
Zheng Pei, T. M. Perrine, F. Plasser, P. Pokhilko, S. Prager, A. Prociuk,
E. Ramos, B. Rana, D. R. Rehn, F. Rob, M. Scheurer, M. Schneider, N. Sergueev,
S. M. Sharada, S. Sharma, D. W. Small, T. Stauch, C. J. Stein, T. Stein,
YuChuan
Su, S. P. Veccham, A. J. W. Thom, A. Tkatchenko, T. Tsuchimochi,
N. M. Tubman, L. Vogt, M. L. Vidal, O. Vydrov, M. A. Watson, J. Wenzel,
M. de Wergifosse, T. A. Wesolowski, A. White, J. Witte, A. Yamada, Jun Yang,
K. Yao, S. Yeganeh, S. R. Yost, ZhiQiang
You, A. Zech, Igor Ying Zhang,

```
Xing Zhang, Yan Zhao, Ying Zhu, B. R. Brooks, G. K. L. Chan, C. J. Cramer,
M. S. Gordon, W. J. Hehre, A. Klamt, M. W. Schmidt, C. D. Sherrill,
D. G. Truhlar, A. AspuruGuzik,
R. Baer, A. T. Bell, N. A. Besley,
JengDa
Chai, A. E. DePrince, III, R. A. DiStasio Jr., A. Dreuw,
B. D. Dunietz, T. R. Furlani, ChaoPing
Hsu, Yousung Jung, Jing Kong,
D. S. Lambrecht, WanZhen Liang, C. Ochsenfeld, V. A. Rassolov,
L. V. Slipchenko, J. E. Subotnik, T. Van Voorhis, J. M. Herbert, A. I. Krylov,
P. M. W. Gill, M. HeadGordon,
Contributors to earlier versions of QChem
not listed above:
R. D. Adamson, B. Austin, J. Baker, G. J. O. Beran, K. Brandhorst,
S. T. Brown, E. F. C. Byrd, A. K. Chakraborty, C.L.
Cheng, Siu Hung Chien,
D. M. Chipman, D. L. Crittenden, H. Dachsel, R. J. Doerksen, A. D. Dutoi,
L. FustiMolnar,
W. A. Goddard III, A. GolubevaZadorozhnaya,
S. R. Gwaltney,
G. Hawkins, A. Heyden, S. Hirata, G. Kedziora, F. J. Keil, C. Kelley,
Jihan Kim, R. A. King, R. Z. Khaliullin, P. P. Korambath, W. Kurlancheek,
A. M. Lee, M. S. Lee, S. V. Levchenko, Ching Yeh Lin, D. Liotard,
R. C. Lochan, I. Lotan, P. E. Maslen, N. Nair, D. P. O'Neill, D. Neuhauser,
E. Neuscamman, C. M. Oana, R. Olson, B. Peters, R. Peverati, P. A. Pieniazek,
Y. M. Rhee, J. Ritchie, M. A. Rohrdanz, E. Rosta, N. J. Russ,
H. F. Schaefer III, N. E. Schultz, N. Shenvi, A. C. Simmonett, A. Sodt,
D. Stuck, K. S. Thanthiriwatte, V. Vanovschi, Tao Wang, A. Warshel,
C. F. Williams, Q. Wu, X. Xu, W. Zhang,
Please cite QChem
as follows :
Y.Shao et al., Mol.Phys. 113, 184 215
(2015)
DOI : 10.1080/00268976.2014.952696
Parts of QChem
use Armadillo 8.300.2 (tropical Shenanigans).
http://arma.sourceforge.net/
Wavefunction Inc. Sales: sales@wavefun.com
Irvine CA Support: support@wavefun.com
Web: www.wavefun.com
Copyright © 1995 2021
Wavefunction
Version of QChem
Parts of QChem
use Armadillo 8.300.2 (Tropical Shenanigans).
http://arma.sourceforge.net/
QChem
begins on Wed Jan 11 10:31:08 2023
Scratch files written to
C:/Users/PATRIC~1/AppData/Local/Temp/WF5AF19F1846E17881//scratch///
Processing $rem in system registry
... MEM_TOTAL 20000 # MB
Processing $rem in C:/Program
Files/Wavefunction/Spartan20v114/P4e//../auxdir/config/preferences:
(site specific preferences)
... THRESH 9
... SMALL_PROD_XCMAT 9
... BASIS_LIN_DEP_THRESH 5
```

```
... SCF_ALGORITHM DIIS_GDM
... MAXSCF 250
... MAXDIIS 45
... THRESHDIIS 1
(i.e. don't switch on deltaE)
... ECP_FIT TRUE (Convert deprecated ECP files)
... GUI GUI_SPARTAN
... TERSE_OUTPUT TRUE !turn on spartan printing
... SCF_CONVERGENCE 7
... CCMAN2 FALSE (qc4.3)
... SYMMETRY FALSE ! turn of symmetry for spartan16
... SYM_IGNORE TRUE ! ..use FORCESYMMETRY to override
... GEOM_OPT_TOL_GRADIENT 700 ! loosen tolernaces for organic geometries
... GEOM_OPT_TOL_DISPLACEMENT 1400 ! was 1200 = .0012
... GEOM_OPT_TOL_ENERGY 2000 ! was 100 = .000 001
... GEN_SCFMAN FALSE
Processing $rem in input file
... JOBTYPE SP
... SCF_CONVERGENCE 7 (sp default for single point energy)
... METHOD WB97MV
... xc_grid 75000302 (75,302)
... BASIS 6311+
G(2DF,2P)
... THRESH 12 #diffuse default
... MAXSCF 350 #diffuse default
... VARTHRESH 2 (default DFT)
... INCDFT TRUE (default DFT)
... GUI GUI_SPARTAN
... TERSE_OUTPUT TRUE
NAlpha2: 266
NElect 266
Mult 1
Warning: disabling incdft.
Checking the input file for inconsistencies... ...done.
User
input:
$
comment
CF3 Cation From Xtal
$end
$molecule
1 1
15 0.25997326718
0.065065591323
0.32114083889
6 5.6372400786
2.8347660336
0.26547173935
9 5.9678878205
3.0093691506
1.552873568
9 6.6183872123
2.1392381586
0.32596255197
9 5.5927048575
4.0480997371
0.3116491435
6 4.2030953707 3.3856271254
```

2.1112408364
9 4.572496215 4.3705632356
1.2795713928
9 5.2869288923 2.6512185396
2.3953241744
9 3.7837996725 3.9554757887
3.2540446649
6 0.80695888059
5.3172355722 2.4478892046
9 0.41098970499 5.8474312233 2.6539031355
9 1.4144269882
5.2134520522 3.6361478429
9 1.5021690637
6.1848327066 1.6979584997
6 0.17392026824 0.13281679279 2.0704625252
1 0.51477094871
0.85789859026 2.5165041506
1 0.028874503112
0.83217496146
2.546896962
6 1.6393877574 0.54486235999 2.2781651634
1 2.2926359957 0.26258704368
1.9320679553
1 1.8851363874 1.4232999878 1.6694530505
6 1.9300989431 0.850760959 3.7478397044
1 1.2817460988 1.669356576 4.0818739929
1 1.6673326586 0.021030255933
4.3570367581
6 3.3929474363 1.2197417308 3.974713558
1 4.0554144348 0.39847314976 3.6865941534
1 3.5824395061 1.4450906732 5.0251656658
1 3.6742453 2.1005499205 3.3906118784
6 1.8321051401
0.93250061544
0.16660577145
6 2.0845764876
1.7331175233
0.95057077816
1 1.3187908817
1.8998182823
1.7002337196
6 3.3263693596
2.3357831718
1.0965430226
1 3.5350966204
2.9556521358
1.9595514241
6 4.3051718341
2.1344110571
0.12955804761
6 4.0610118522
1.3377850505
0.98348421577
1 4.8355731308
1.1866719313
1.7249364989
6 2.821740558
0.73074740782

```
1.1317784544
1 2.6412256969
0.10379929797
1.9990837147
6 1.0286979992 1.0383999058
0.47481421895
6 1.0929508853 2.4100176609
0.20242448853
1 0.33440668834 2.8934635906
0.40523329104
6 2.1339222312 3.1632366663
0.7218792966
1 2.1962061754 4.2248007918
0.51699467536
6 3.1041355884 2.5438405771
1.5062050858
6 3.0496459395 1.1822671637
1.7740889011
1 3.8167610009 0.71571733577
2.3775918906
6 2.0088929152 0.42183458175
1.2531836902
1 1.9685378145 0.64214307762 1.4558511065
6 0.38963134374
1.5433799585 0.48138478191
6 0.2680830817
2.7277156067 0.24761714422
1 0.058163665501
2.7158273214 1.3105852504
6 0.41981597589
3.9483600477 0.39670106252
1 0.33370277477
4.8735203694 0.1602328217
6 0.68960067218
3.9771839532 1.7599134842
6 0.81615482229
2.801276473 2.4920257657
1 1.0367055178
2.840583602 3.5508796915
6 0.67071030652
1.5802210093 1.8503018439
1 0.77914853919
0.66306665525 2.4205731804
$end
$rem
JOBTYPE SP
SCF_CONVERGENCE 7 (sp default for single point energy)
METHOD WB97MV
xc_grid 75000302 (75,302)
BASIS 6311+
G(2DF,2P)
THRESH 12 #diffuse default
MAXSCF 350 #diffuse default
VARTHRESH 2 (default DFT)
INCDFT TRUE (default DFT)
GUI GUI_SPARTAN
TERSE_OUTPUT TRUE
$end
```

S
tandard Nuclear Orientation (Angstroms)
I Atom X Y Z
1
P 0.2599732672
0.0650655913
0.3211408389
2 C 5.6372400786
2.8347660336
0.2654717394
3 F 5.9678878205
3.0093691506
1.5528735680
4 F 6.6183872123
2.1392381586
0.3259625520
5 F 5.5927048575
4.0480997371
0.3116491435
6 C 4.2030953707 3.3856271254
2.1112408364
7 F 4.5724962150 4.3705632356
1.2795713928
8 F 5.2869288923 2.6512185396
2.3953241744
9 F 3.7837996725 3.9554757887
3.2540446649
10 C 0.8069588806
5.3172355722 2.4478892046
11 F 0.4109897050 5.8474312233 2.6539031355
12 F 1.4144269882
5.2134520522 3.6361478429
13 F 1.5021690637
6.1848327066 1.6979584997
14 C 0.1739202682 0.1328167928 2.0704625252
15 H 0.5147709487
0.8578985903 2.5165041506
16 H 0.0288745031
0.8321749615
2.5468969620
17 C 1.6393877574 0.5448623600 2.2781651634
18 H 2.2926359957 0.2625870437
1.9320679553
19 H 1.8851363874 1.4232999878 1.6694530505
20 C 1.9300989431 0.8507609590 3.7478397044
21 H 1.2817460988 1.6693565760 4.0818739929
22 H 1.6673326586 0.0210302559
4.3570367581
23 C 3.3929474363 1.2197417308 3.9747135580
24 H 4.0554144348 0.3984731498 3.6865941534
25 H 3.5824395061 1.4450906732 5.0251656658
26 H 3.6742453000 2.1005499205 3.3906118784
27 C 1.8321051401
0.9325006154
0.1666057714
28 C 2.0845764876
1.7331175233
0.9505707782

29 H 1.3187908817
1.8998182823
1.7002337196
30 C 3.3263693596
2.3357831718
1.0965430226
31 H 3.5350966204
2.9556521358
1.9595514241
32 C 4.3051718341
2.1344110571
0.1295580476
33 C 4.0610118522
1.3377850505
0.9834842158
34 H 4.8355731308
1.1866719313
1.7249364989
35 C 2.8217405580
0.7307474078
1.1317784544
36 H 2.6412256969
0.1037992980
1.9990837147
37 C 1.0286979992 1.0383999058
0.4748142189
38 C 1.0929508853 2.4100176609
0.2024244885
39 H 0.3344066883 2.8934635906
0.4052332910
40 C 2.1339222312 3.1632366663
0.7218792966
41 H 2.1962061754 4.2248007918
0.5169946754
42 C 3.1041355884 2.5438405771
1.5062050858
43 C 3.0496459395 1.1822671637
1.7740889011
44 H 3.8167610009 0.7157173358
2.3775918906
45 C 2.0088929152 0.4218345817
1.2531836902
46 H 1.9685378145 0.6421430776 1.4558511065
47 C 0.3896313437
1.5433799585 0.4813847819
48 C 0.2680830817
2.7277156067 0.2476171442
49 H 0.0581636655
2.7158273214 1.3105852504
50 C 0.4198159759
3.9483600477 0.3967010625
51 H 0.3337027748
4.8735203694 0.1602328217
52 C 0.6896006722
3.9771839532 1.7599134842
53 C 0.8161548223
2.8012764730 2.4920257657
54 H 1.0367055178

2.8405836020 3.5508796915
55 C 0.6707103065
1.5802210093 1.8503018439
56 H 0.7791485392
0.6630666552 2.4205731804
Nuclear
Repulsion Energy = 3962.07536779 hartrees
There are 133 alpha and 133 beta electrons
Requested basis set is 6311+
G(2df,2p)
There are 392 shells and 1387 basis functions
Total QAlloc Memory Limit 20000 MB
MegaArray
Size 188 MB
MEM_STATIC part 192 MB
.. (5.2.P)
Entering
fldman on Wed Jan 11 10:31:08 2023 A
cutoff of 1.0D12
yielded 42219 shell pairs
There are 510256 function pairs (624920 Cartesian)
Smallest overlap matrix eigenvalue = 1.80E07
Linear dependence detected in AO basis
Tighter screening thresholds may be required for diffuse basis sets
Use S2THRESH > 12 and THRESH = 14 in case of SCF convergence issues
Number of orthogonalized atomic orbitals = 1367
Maximum deviation from orthogonality = 2.077E11
Scale SEOQF with 1.000000e02/
1.000000e02/
1.000000e01
Standard Electronic Orientation quadrupole field applied
Nucleusfield
energy = 0.0000000790
hartrees
Entering
gesman on Wed Jan 11 10:31:13 2023 Guess
from superposition of atomic densities
Warning: Energy on first SCF cycle will be nonvariational
SAD guess density has 263.164804 electrons
Entering
scfman on Wed Jan 11 10:31:14 2023 Longrange
K will be added via erf
Coulomb attenuation parameter = 0.3 bohr**(1)
A restricted hybrid HFDFT
SCF calculation will be
performed using Pulay DIIS + Geometric Direct Minimization
Exchange: 0.1500 HartreeFock
+ 1.0000 wB97MV
+ LRHF
Correlation: 1.0000 wB97MV
Using EulerMaclaurinLebedev
(75,302) quadrature formula
Nonlocal Correlation: VV10 with C = 0.0100 and b = 6.00 and scale = 1.00000
Grid used for NLC: SG1
standard quadrature
SCF converges when RMS gradient is below 1.0E07
Exchange: 0.1500 HartreeFock
+ 1.0000 wB97MV

```
+ LRHF
Correlation: 1.0000 wB97MV
Using EulerMaclaurinLebedev
(75,302) quadrature formula
Nonlocal Correlation: VV10 with C = 0.0100 and b = 6.00 and scale = 1.00000
Grid used for NLC: SG1
standard quadrature
using 4 threads for integral computing
OpenMP
Integral computing Module
Release: version 1.0, May 2013, QChem
Inc. Pittsburgh
using
4 threads for integral computing
OpenMP
Integral computing Module
Release: version 1.0, May 2013, QChem
Inc. Pittsburgh
OpenMP
BLAS3 based DFT computing Module
Release: version 1.0, May 2013, QChem
Inc. Pittsburgh
OpenMP
BLAS3 based DFT computing Module
Release: version 1.0, May 2013, QChem
Inc. Pittsburgh
OpenMP
BLAS3 based DFT computing Module
Release: version 1.0, May 2013, QChem
Inc. Pittsburgh
Cycle
Energy DIIS Error
1
2206.7229391185
3.70E02
2 2056.8837732790
2.43E02
3 1444.8643809834
3.46E02
4 1694.7022363744
3.14E02
5 2042.9857738562
1.63E02
6 1305.0457075773
4.55E02
7 1704.1503409119
3.20E02
8 1782.3506098791
2.96E02
9 1898.1717225772
2.46E02
10 2077.6811049267
1.67E02
11 2101.5666529702
1.07E02
12 1308.7526658037
4.15E02
13 1639.2041651546
```

3.21E02
14 1703.3432171537
3.05E02
15 1709.8978903679
3.02E02
VarThresh incresed to 3 to improve convergence.
16 1711.6934229833
3.05E02
17 1703.1335870126
3.07E02
18 1639.0620348633
3.32E02
19 1640.9230777185
3.28E02
20 1645.8700516673
3.28E02
21 465.9422170203
7.06E02
22 563.6489788173
7.07E02
VarThresh incresed to 4 to improve convergence.
23 594.7591381669
6.79E02
VarThresh incresed to 5 to improve convergence.
24 590.3423827339
6.82E02
25 582.8162646395
6.97E02
VarThresh incresed to 6 to improve convergence.
26 464.1528324066
7.02E02
27 1522.4140197075 1.04E01
28 1109.2242849059
4.21E02
29 864.0032462058
5.73E02
30 1739.6670812699
2.67E02
31 1207.4815907707
4.15E02
32 1748.0071579084
2.68E02
33 2109.5683749197
1.50E02
34 1352.0946829293
3.81E02
35 1983.5835094140
1.83E02
36 2185.3971732720
7.20E03
37 2089.7770046541
1.32E02
38 2108.7739610607
1.19E02
39 2182.5455288989
7.02E03
40 2155.2691781315
9.08E03

```
41 2184.3568231471
6.39E03
42 2193.9257197592
5.17E03
43 2200.7108642371
3.20E03
44 2204.8083333405
1.08E03
45 2205.0314564530
6.80E04
Done DIIS. Switching to GDM
46 2205.1283981637
3.42E02
Normal BFGS step
47 2205.1332409585
2.80E02
Normal BFGS step
48 2205.1409586189
3.07E03
Normal BFGS step
49 2205.1411848347
2.29E03
Normal BFGS step
50 2205.1413221843
7.56E04
Normal BFGS step
51 2205.1413294633
3.73E04
Normal BFGS step
52 2205.1413347129
1.40E04
Normal BFGS step
53 2205.1413350579
6.07E05
Normal BFGS step
54 2205.1413351904
1.99E05
Normal BFGS step
55 2205.1413352002
6.15E06
Normal BFGS step
56 2205.1413352028
3.05E06
Normal BFGS step
57 2205.1413352032
1.14E06
Normal BFGS step
58 2205.1413352032
6.74E07
Normal BFGS step
59 2205.1413352032
2.14E07
Normal BFGS step
60 2205.1413352033
6.29E08
Convergence criterion met
SCF
time: CPU 117275.72 s wall 29622.70 s
```

SCF energy in the final basis set = 2205.14133520
Total energy in the final basis set = 2205.14133520
Entering
anlman on Wed Jan 11 18:45:01 2023 Orbital
Energies (a.u.)
Alpha
MOs
Occupied
77.5977
25.0404
25.0402
25.0394
25.0384
25.0384
25.0383
25.0364
25.0362
25.0350
10.7045
10.7042
10.7032
10.5228
10.5219
10.5215
10.5156
10.5110
10.5107
10.5104
10.5096
10.5096
10.5093
10.5091
10.5089
10.5082
10.4993
10.4991
10.4988
10.4984
10.4977
10.4977
10.4839
10.4571
10.4303
6.9476
5.0597
5.0595
5.0593
1.5533
1.5532
1.5518
1.4667
1.4666
1.4652
1.4628
1.4624
1.4614
1.1600
1.1461

1.1451
1.0980
1.0598
1.0497
1.0485
1.0294
1.0286
1.0264
1.0031
0.9663
0.9449
0.9443
0.9126
0.8928
0.8881
0.8825
0.8761
0.8709
0.8692
0.8303
0.8254
0.8165
0.8153
0.8141
0.8107
0.8105
0.8102
0.8096
0.8092
0.8081
0.7583
0.7494
0.7299
0.7281
0.7194
0.7146
0.7087
0.7067
0.7034
0.6980
0.6858
0.6856
0.6844
0.6840
0.6833
0.6829
0.6818
0.6806
0.6792
0.6674
0.6641
0.6559
0.6545
0.6528
0.6490
0.6423
0.6412
0.6399

0.6385
0.6312
0.6274
0.6253
0.6249
0.6238
0.6210
0.6153
0.6095
0.5976
0.5973
0.5959
0.5917
0.5862
0.5774
0.5757
0.5610
0.5464
0.5446
0.5142
0.5049
0.5018
0.4987
0.4962
0.4925
Virtual
0.1298
0.1194
0.1089
0.0947
0.0904
0.0840
0.0754
0.0715
0.0695
0.0646
0.0526
0.0467
0.0448
0.0405
0.0338
0.0271
0.0265
0.0214
0.0181
0.0172
0.0140
0.0127
0.0100
0.0049
0.0010
0.0007 0.0024 0.0055 0.0078 0.0100 0.0127 0.0141
0.0175 0.0190 0.0232 0.0260 0.0281 0.0298 0.0328 0.0391
0.0412 0.0434 0.0450 0.0483 0.0502 0.0516 0.0538 0.0567
0.0577 0.0615 0.0626 0.0649 0.0663 0.0716 0.0744 0.0747
0.0781 0.0799 0.0846 0.0873 0.0908 0.0923 0.0944 0.0989
0.0992 0.1021 0.1041 0.1097 0.1120 0.1130 0.1147 0.1186
0.1198 0.1208 0.1235 0.1250 0.1262 0.1275 0.1306 0.1308

0.1340	0.1352	0.1395	0.1439	0.1454	0.1460	0.1494	0.1542
0.1552	0.1596	0.1619	0.1633	0.1668	0.1676	0.1692	0.1737
0.1752	0.1782	0.1792	0.1812	0.1827	0.1845	0.1873	0.1893
0.1904	0.1937	0.1984	0.2011	0.2025	0.2036	0.2058	0.2078
0.2093	0.2124	0.2138	0.2172	0.2215	0.2234	0.2240	0.2254
0.2263	0.2282	0.2286	0.2340	0.2371	0.2405	0.2414	0.2429
0.2463	0.2500	0.2519	0.2536	0.2572	0.2622	0.2662	0.2687
0.2700	0.2745	0.2775	0.2809	0.2821	0.2850	0.2934	0.2959
0.3019	0.3059	0.3098	0.3122	0.3172	0.3195	0.3216	0.3242
0.3258	0.3291	0.3314	0.3320	0.3355	0.3377	0.3417	0.3443
0.3475	0.3501	0.3547	0.3562	0.3573	0.3590	0.3625	0.3652
0.3683	0.3705	0.3722	0.3745	0.3785	0.3812	0.3832	0.3905
0.3925	0.3977	0.3999	0.4044	0.4066	0.4092	0.4124	0.4150
0.4173	0.4212	0.4243	0.4279	0.4358	0.4385	0.4419	0.4430
0.4451	0.4482	0.4494	0.4558	0.4617	0.4681	0.4687	0.4733
0.4764	0.4802	0.4838	0.4897	0.4949	0.4965	0.4992	0.5091
0.5108	0.5154	0.5179	0.5212	0.5243	0.5270	0.5282	0.5292
0.5355	0.5419	0.5444	0.5483	0.5504	0.5523	0.5573	0.5590
0.5626	0.5659	0.5666	0.5689	0.5742	0.5751	0.5793	0.5811
0.5849	0.5880	0.5885	0.5900	0.5945	0.5963	0.6009	0.6039
0.6050	0.6069	0.6114	0.6131	0.6144	0.6157	0.6172	0.6213
0.6215	0.6258	0.6278	0.6303	0.6319	0.6377	0.6397	0.6415
0.6470	0.6489	0.6522	0.6558	0.6593	0.6600	0.6616	0.6643
0.6652	0.6665	0.6710	0.6722	0.6753	0.6790	0.6858	0.6864
0.6888	0.6913	0.6925	0.6964	0.7029	0.7079	0.7112	0.7126
0.7137	0.7180	0.7248	0.7266	0.7335	0.7374	0.7389	0.7432
0.7481	0.7502	0.7508	0.7580	0.7620	0.7692	0.7722	0.7752
0.7783	0.7834	0.7842	0.7906	0.7981	0.7999	0.8010	0.8036
0.8076	0.8102	0.8158	0.8179	0.8246	0.8249	0.8280	0.8332
0.8344	0.8402	0.8428	0.8439	0.8535	0.8611	0.8638	0.8751
0.8820	0.8857	0.8877	0.8966	0.9049	0.9062	0.9109	0.9150
0.9206	0.9264	0.9312	0.9322	0.9397	0.9462	0.9538	0.9559
0.9599	0.9670	0.9763	0.9798	0.9830	0.9855	0.9876	0.9940
0.9994	1.0018	1.0060	1.0112	1.0131	1.0161	1.0205	1.0244
1.0281	1.0322	1.0431	1.0492	1.0510	1.0575	1.0662	1.0684
1.0762	1.0815	1.0851	1.0887	1.0928	1.0966	1.1030	1.1050
1.1122	1.1172	1.1230	1.1311	1.1356	1.1382	1.1435	1.1502
1.1531	1.1575	1.1675	1.1711	1.1747	1.1771	1.1813	1.1818
1.1903	1.1966	1.2004	1.2069	1.2115	1.2149	1.2210	1.2249
1.2290	1.2367	1.2397	1.2425	1.2438	1.2486	1.2517	1.2568
1.2575	1.2623	1.2648	1.2705	1.2739	1.2823	1.2845	1.2859
1.2921	1.2965	1.2987	1.2998	1.3105	1.3121	1.3130	1.3145
1.3228	1.3267	1.3273	1.3291	1.3353	1.3377	1.3427	1.3484
1.3529	1.3554	1.3607	1.3635	1.3709	1.3771	1.3787	1.3817
1.3843	1.3876	1.3906	1.3919	1.3961	1.3973	1.4027	1.4034
1.4063	1.4071	1.4114	1.4126	1.4166	1.4188	1.4207	1.4250
1.4273	1.4297	1.4306	1.4385	1.4394	1.4442	1.4457	1.4474
1.4503	1.4521	1.4562	1.4587	1.4626	1.4688	1.4710	1.4779
1.4816	1.4836	1.4895	1.4915	1.4965	1.4970	1.5023	1.5052
1.5128	1.5141	1.5156	1.5223	1.5235	1.5296	1.5313	1.5364
1.5420	1.5463	1.5523	1.5616	1.5728	1.5755	1.5806	1.5866
1.5888	1.5954	1.5980	1.6072	1.6110	1.6168	1.6318	1.6383
1.6410	1.6455	1.6528	1.6614	1.6669	1.6781	1.6994	1.7023
1.7063	1.7150	1.7178	1.7294	1.7332	1.7441	1.7557	1.7601
1.7635	1.7688	1.7808	1.7862	1.7875	1.7997	1.8097	1.8278
1.8496	1.8675	1.8731	1.8957	1.9092	1.9130	1.9375	1.9429
1.9472	1.9528	1.9563	1.9613	1.9633	1.9727	1.9774	1.9836
1.9965	2.0032	2.0036	2.0090	2.0148	2.0193	2.0247	2.0307

2.0393	2.0519	2.0545	2.0616	2.0757	2.0817	2.0830	2.0919
2.1028	2.1102	2.1242	2.1294	2.1346	2.1421	2.1429	2.1444
2.1482	2.1550	2.1613	2.1692	2.1708	2.1808	2.1929	2.2061
2.2227	2.2406	2.2585	2.2834	2.2916	2.2964	2.3030	2.3109
2.3161	2.3318	2.3420	2.3623	2.3797	2.4072	2.4250	2.4503
2.4628	2.4655	2.4702	2.4851	2.4886	2.5090	2.5117	2.5164
2.5325	2.5412	2.5480	2.5491	2.5537	2.5558	2.5630	2.5684
2.5759	2.5826	2.5860	2.5895	2.5908	2.5918	2.5944	2.5990
2.6087	2.6176	2.6228	2.6282	2.6324	2.6376	2.6387	2.6474
2.6553	2.6589	2.6790	2.6816	2.6926	2.6935	2.6982	2.7035
2.7040	2.7110	2.7134	2.7208	2.7222	2.7268	2.7320	2.7376
2.7419	2.7454	2.7574	2.7674	2.7727	2.7789	2.7824	2.7849
2.7879	2.7932	2.7941	2.7982	2.8049	2.8106	2.8117	2.8150
2.8204	2.8223	2.8242	2.8278	2.8312	2.8325	2.8379	2.8436
2.8509	2.8538	2.8565	2.8567	2.8619	2.8647	2.8652	2.8735
2.8765	2.8817	2.8842	2.8894	2.8971	2.8976	2.9026	2.9066
2.9096	2.9137	2.9170	2.9224	2.9323	2.9384	2.9390	2.9493
2.9521	2.9581	2.9689	2.9762	2.9865	2.9878	2.9958	3.0106
3.0126	3.0167	3.0226	3.0270	3.0307	3.0366	3.0405	3.0434
3.0471	3.0508	3.0584	3.0607	3.0668	3.0729	3.0785	3.0836
3.0896	3.0920	3.1027	3.1045	3.1073	3.1149	3.1154	3.1215
3.1241	3.1264	3.1326	3.1410	3.1442	3.1461	3.1508	3.1547
3.1576	3.1646	3.1693	3.1723	3.1802	3.1847	3.1932	3.1992
3.2014	3.2056	3.2168	3.2194	3.2217	3.2244	3.2313	3.2381
3.2398	3.2453	3.2486	3.2493	3.2588	3.2635	3.2702	3.2770
3.2836	3.2846	3.2871	3.2912	3.2964	3.3064	3.3091	3.3114
3.3143	3.3187	3.3224	3.3255	3.3295	3.3365	3.3378	3.3406
3.3427	3.3461	3.3500	3.3543	3.3560	3.3581	3.3627	3.3681
3.3708	3.3730	3.3754	3.3816	3.3889	3.3913	3.3942	3.3977
3.4097	3.4111	3.4153	3.4238	3.4273	3.4330	3.4368	3.4399
3.4545	3.4567	3.4596	3.4673	3.4688	3.4730	3.4764	3.4794
3.4823	3.4864	3.4918	3.4942	3.4971	3.5051	3.5080	3.5111
3.5177	3.5248	3.5293	3.5313	3.5356	3.5375	3.5391	3.5425
3.5475	3.5579	3.5618	3.5644	3.5709	3.5726	3.5813	3.5889
3.5911	3.5951	3.5967	3.6028	3.6135	3.6199	3.6245	3.6275
3.6296	3.6347	3.6428	3.6467	3.6509	3.6548	3.6601	3.6657
3.6698	3.6743	3.6756	3.6810	3.6879	3.6926	3.6966	3.7018
3.7057	3.7067	3.7122	3.7199	3.7234	3.7343	3.7351	3.7357
3.7427	3.7452	3.7514	3.7597	3.7631	3.7691	3.7709	3.7818
3.7862	3.7929	3.8014	3.8090	3.8104	3.8247	3.8284	3.8325
3.8361	3.8384	3.8415	3.8555	3.8615	3.8651	3.8688	3.8842
3.8934	3.9014	3.9045	3.9083	3.9156	3.9178	3.9201	3.9244
3.9261	3.9419	3.9489	3.9525	3.9621	3.9715	3.9750	3.9782
3.9785	3.9800	3.9838	3.9872	3.9951	3.9987	4.0129	4.0237
4.0301	4.0475	4.0603	4.0703	4.0717	4.0766	4.0810	4.0832
4.0870	4.0911	4.0978	4.0996	4.1060	4.1132	4.1213	4.1284
4.1443	4.1485	4.1549	4.1717	4.1812	4.1906	4.1977	4.2033
4.2135	4.2188	4.2214	4.2280	4.2318	4.2369	4.2425	4.2463
4.2559	4.2576	4.2626	4.2753	4.2834	4.2896	4.3016	4.3093
4.3126	4.3195	4.3269	4.3293	4.3318	4.3379	4.3407	4.3460
4.3495	4.3565	4.3580	4.3629	4.3687	4.3726	4.3800	4.3838
4.3886	4.3894	4.3929	4.3974	4.3989	4.4027	4.4066	4.4116
4.4148	4.4216	4.4258	4.4263	4.4296	4.4352	4.4560	4.4609
4.4644	4.4694	4.4753	4.4862	4.4897	4.5032	4.5103	4.5181
4.5274	4.5293	4.5326	4.5393	4.5448	4.5563	4.5716	4.5822
4.5920	4.5940	4.6001	4.6140	4.6158	4.6206	4.6247	4.6325
4.6421	4.6886	4.6931	4.7004	4.7065	4.7137	4.7223	4.7331
4.7602	4.7907	4.8155	4.8401	4.8500	4.8840	4.8877	4.8891

4.9583 4.9607 4.9669 4.9949 5.0017 5.0109 5.0167 5.0273
5.0388 5.0416 5.0647 5.0715 5.0942 5.1328 5.1348 5.1423
5.2690 5.3066 5.3212 5.3455 5.3536 5.3628 5.3832 5.3944
5.4033 5.4075 5.4242 5.4401 5.5166 5.5442 5.5518 5.5586
5.5683 5.6053 5.6509 5.6598 5.6618 5.6664 5.6682 5.6949
5.7404 5.7437 5.7497 5.7581 5.7615 5.7670 6.0158 6.0215
6.0273 6.4159 6.4174 6.4197 6.4283 6.4373 6.4431 6.4468
6.4534 6.4619 6.4728 6.4751 6.4783 6.4900 6.4906 6.4923
6.5107 6.5169 6.5189 6.5233 6.5257 6.5329 6.6321 6.6323
6.6336 6.6354 6.6358 6.6363 6.6367 6.6381 6.6405 6.6408
6.6409 6.6418 6.6484 6.6493 6.6500 6.6501 6.6508 6.6517
6.6545 6.6553 6.6565 6.6709 6.6716 6.6722 6.6848 6.6857
6.6862 6.7462 6.7474 6.7487 6.7564 6.7578 6.7591 6.7626
6.7635 6.7638 6.8545 6.8551 6.8562 6.8668 6.8738 6.8793
6.8817 6.8848 6.8926 7.0159 7.0159 7.0177 7.0318 7.0326
7.0338 7.1214 7.1223 7.1232 7.2664 7.2701 7.2724 7.2984
7.2992 7.3047 7.3676 7.3696 7.3698 7.4342 7.4364 7.4376
7.7869 7.7908 7.7926 7.8029 7.8036 7.8106 8.1273 9.1018
9.1022 9.1034 9.1238 9.1251 9.1264 9.1290 9.1333 9.1358
9.2291 9.2345 9.2406 9.2419 9.2445 9.2509 9.2813 9.2818
9.2824 9.4460 9.4474 9.4490 9.5361 9.5377 9.5393 9.5473
9.5476 9.5526 9.6333 9.6337 9.6378 9.7962 9.7973 9.7988
9.9362 9.9373 9.9395 9.9725 9.9739 9.9747 10.1691 10.1734
10.1772 10.1879 10.1888 10.1913 14.6076 14.7070 14.7294 24.0729
24.0804 24.1110 24.4094 24.5012 24.5765 24.6353 24.6522 24.6549
24.6724 24.6818 24.6887 24.6943 24.8116 24.8130 24.8175 24.8836
24.8896 24.8910 24.9431 24.9483 24.9573 25.3625 25.3704 25.3736
67.4262 67.4305 67.4326 67.4333 67.4345 67.4352 67.4579 67.4590
67.4671 165.2591
GroundState
Mulliken Net Atomic Charges
Atom Charge (a.u.)
1
P 0.222819
2 C 0.628860
3 F 0.176303
4 F 0.177695
5 F 0.140463
6 C 0.656670
7 F 0.171297
8 F 0.183903
9 F 0.138522
10 C 0.638115
11 F 0.140267
12 F 0.193523
13 F 0.165722
14 C 0.514961
15 H 0.253832
16 H 0.249945
17 C 0.474978
18 H 0.241886
19 H 0.219342
20 C 0.339132
21 H 0.170119
22 H 0.176045
23 C 0.568198
24 H 0.160727
25 H 0.171690

26 H 0.156695
27 C 0.415279
28 C 0.265250
29 H 0.238897
30 C 0.177703
31 H 0.202682
32 C 0.025367
33 C 0.177973
34 H 0.201688
35 C 0.177610
36 H 0.198430
37 C 0.333344
38 C 0.278708
39 H 0.232696
40 C 0.191415
41 H 0.203511
42 C 0.045982
43 C 0.192687
44 H 0.205824
45 C 0.257163
46 H 0.245032
47 C 0.263856
48 C 0.265245
49 H 0.224327
50 C 0.167452
51 H 0.201461
52 C 0.070170
53 C 0.174213
54 H 0.203580
55 C 0.235350
56 H 0.215532
Sum
of atomic charges = 1.000000
Cartesian
Multipole Moments
Charge
(ESU x 10^10)
4.8032
Dipole Moment (Debye)
X 0.2190
Y 0.1173 Z 2.6080
Tot 2.6199
Quadrupole Moments (DebyeAng)
XX 198.7898
XY 2.9409 YY 195.4915
XZ 2.2548 YZ 5.2885 ZZ 182.3607
Traceless Quadrupole Moments (DebyeAng)
QXX 19.7275
QYY 9.8326
QZZ 29.5601
QXY 8.8226 QXZ 6.7645 QYZ 15.8654
Octopole Moments (DebyeAng^
2)
XXX 185.8461 XXY 51.2035 XYY 64.0675
YYY 36.9296 XXZ 126.2757 XYZ 10.6029
YYZ 127.0301 XZZ 53.9697 YZZ 36.0123
ZZZ 309.3558
Traceless Octopole Moments (DebyeAng^

2)
XXX 52.7414 YYY 563.3649
ZZZ 423.6178
XXY 395.6163 XXZ 206.1503 XYY 49.3628
XYZ 159.0430
XZZ 102.1041
YYZ 217.4674
YZZ 167.7486
Hexadecapole Moments (DebyeAng^
3)
XXXX 11739.0569
XXXY 42.7325 XXYY 3843.1657
XYYY 53.0428 YYYY 10571.9026
XXXZ 9.5409
XXYZ 67.1490 XYYZ 101.3611 YYYZ 449.0499
XXZZ 2536.5133
XYZZ 95.4579 YYZZ 2552.7993
XZZZ 177.6874 YZZZ 259.2973 ZZZZ 4301.7763
Traceless Hexadecapole Moments (DebyeAng^
3)
XXXX 2213.9732
XXXY 4118.5813
XXXZ 11984.7295
XXYY 10666.4227
XXYZ 4577.3362
XXZZ 12880.3959
XYYY 3036.0001
XYYZ 6314.0748 XYZZ 7154.5814
XZZZ 5670.6547 YYYY 16759.0786 YYYZ 12266.2871
YYZZ 6092.6559
YZZZ 7688.9509
ZZZZ 6787.7399
Total
job time: 29636.10s(wall), 117291.41s(cpu)
Wed Jan 11 18:45:04 2023

SPARTAN'20
build 1.1.4 (Dec 1 2021)
Wavefunction Developers:
B.J. Deppmeier, A.J. Driessen, W.J. Hehre, T.S. Hehre,
J.A. Johnson, W.S. Ohlinger, P.E. Klunzinger
Please cite Spartan as:
Spartan'20
Wavefunction Inc.
Irvine CA
QChem
5.1, QChem,
Inc., Pleasanton, CA (2020)
QChem
Developers:
Yihan Shao, Zhengting Gan, E. Epifanovsky, A. T. B. Gilbert, M. Wormit,
J. Kussmann, A. W. Lange, A. Behn, Jia Deng, Xintian Feng, D. Ghosh,
M. Goldey, P. R. Horn, L. D. Jacobson, I. Kaliman, T. Kus, A. Landau, Jie Liu,
E. I. Proynov, R. M. Richard, R. P. Steele, E. J. Sundstrom,
H. L. Woodcock III, P. M. Zimmerman, D. Zuev, B. Alam, B. Albrecht,
E. Alguire, S. A. Baeppler, D. Barton, Z. Benda, Y. A. Bernard,
E. J. Berquist, K. B. Bravaya, H. Burton, K. CarterFenk,
D. Casanova,
ChunMin
Chang, Yunqing Chen, A. Chien, K. D. Closser, M. P. Coons,
S. Coriani, S. Dasgupta, A. L. Dempwolff, M. Diedenhofen, Hainam Do,
R. G. Edgar, PoTung
Fang, S. Faraji, S. Fatehi, Qingguo Feng, J. FossoTande,
J. Gayvert, Qinghui Ge, A. Ghysels, G. Gidofalvi, J. Gomes, J. Gonthier,
A. Gunina, D. Hait, M. W. D. HansonHeine,
P. H. P. Harbach, A. W. Hauser,
M. F. Herbst, J. E. Herr, E. G. Hohenstein, Z. C. Holden, Kerwin Hui,
B. C. Huynh, T.C.
Jagau, Hyunjun Ji, B. Kaduk, K. Khistyaev, Jaehoon Kim,
P. Klunzinger, K. Koh, D. Kosenkov, L. Koulias, T. Kowalczyk, C. M. Krauter,
A. Kunitsa, Ka Un Lao, A. Laurent, K. V. Lawler, Joonho Lee, D. Lefrancois,
S. Lehtola, D. S. Levine, YiPei
Li, YouSheng
Lin, Fenglai Liu, KuanYu
Liu,
E. Livshits, M. Loipersberger, A. Luenser, P. Manohar, E. Mansoor,
S. F. Manzer, ShanPing
Mao, Yuezhi Mao, N. Mardirossian, A. V. Marenich,
T. Markovich, L. A. MartinezMartinez,
S. A. Maurer, N. J. Mayhall,
S. C. McKenzie, J.M.
Mewes, P. Morgante, A. F. Morrison, J. W. Mullinax,
K. Nanda, T. S. NguyenBeck,
R. OlivaresAmaya,
J. A. Parkhill, S. K. Paul,
Zheng Pei, T. M. Perrine, F. Plasser, P. Pokhilko, S. Prager, A. Prociuk,
E. Ramos, B. Rana, D. R. Rehn, F. Rob, M. Scheurer, M. Schneider, N. Sergueev,
S. M. Sharada, S. Sharma, D. W. Small, T. Stauch, C. J. Stein, T. Stein,
YuChuan
Su, S. P. Veccham, A. J. W. Thom, A. Tkatchenko, T. Tsuchimochi,
N. M. Tubman, L. Vogt, M. L. Vidal, O. Vydrov, M. A. Watson, J. Wenzel,
M. de Wergifosse, T. A. Wesolowski, A. White, J. Witte, A. Yamada, Jun Yang,
K. Yao, S. Yeganeh, S. R. Yost, ZhiQiang
You, A. Zech, Igor Ying Zhang,

```
Xing Zhang, Yan Zhao, Ying Zhu, B. R. Brooks, G. K. L. Chan, C. J. Cramer,
M. S. Gordon, W. J. Hehre, A. Klamt, M. W. Schmidt, C. D. Sherrill,
D. G. Truhlar, A. AspuruGuzik,
R. Baer, A. T. Bell, N. A. Besley,
JengDa
Chai, A. E. DePrince, III, R. A. DiStasio Jr., A. Dreuw,
B. D. Dunietz, T. R. Furlani, ChaoPing
Hsu, Yousung Jung, Jing Kong,
D. S. Lambrecht, WanZhen Liang, C. Ochsenfeld, V. A. Rassolov,
L. V. Slipchenko, J. E. Subotnik, T. Van Voorhis, J. M. Herbert, A. I. Krylov,
P. M. W. Gill, M. HeadGordon,
Contributors to earlier versions of QChem
not listed above:
R. D. Adamson, B. Austin, J. Baker, G. J. O. Beran, K. Brandhorst,
S. T. Brown, E. F. C. Byrd, A. K. Chakraborty, C.L.
Cheng, Siu Hung Chien,
D. M. Chipman, D. L. Crittenden, H. Dachsel, R. J. Doerksen, A. D. Dutoi,
L. FustiMolnar,
W. A. Goddard III, A. GolubevaZadorozhnaya,
S. R. Gwaltney,
G. Hawkins, A. Heyden, S. Hirata, G. Kedziora, F. J. Keil, C. Kelley,
Jihan Kim, R. A. King, R. Z. Khaliullin, P. P. Korambath, W. Kurlancheek,
A. M. Lee, M. S. Lee, S. V. Levchenko, Ching Yeh Lin, D. Liotard,
R. C. Lochan, I. Lotan, P. E. Maslen, N. Nair, D. P. O'Neill, D. Neuhauser,
E. Neuscamman, C. M. Oana, R. Olson, B. Peters, R. Peverati, P. A. Pieniazek,
Y. M. Rhee, J. Ritchie, M. A. Rohrdanz, E. Rosta, N. J. Russ,
H. F. Schaefer III, N. E. Schultz, N. Shenvi, A. C. Simmonett, A. Sodt,
D. Stuck, K. S. Thanthiriwatte, V. Vanovschi, Tao Wang, A. Warshel,
C. F. Williams, Q. Wu, X. Xu, W. Zhang,
Please cite QChem
as follows :
Y.Shao et al., Mol.Phys. 113, 184 215
(2015)
DOI : 10.1080/00268976.2014.952696
Parts of QChem
use Armadillo 8.300.2 (tropical Shenanigans).
http://arma.sourceforge.net/
Wavefunction Inc. Sales: sales@wavefun.com
Irvine CA Support: support@wavefun.com
Web: www.wavefun.com
Copyright © 1995 2021
Wavefunction
Version of QChem
Parts of QChem
use Armadillo 8.300.2 (Tropical Shenanigans).
http://arma.sourceforge.net/
QChem
begins on Sun Jan 15 11:58:35 2023
Scratch files written to
C:/Users/PATRIC~1/AppData/Local/Temp/WF72E21C309522A7F1//scratch///
Processing $rem in system registry
... MEM_TOTAL 20000 # MB
Processing $rem in C:/Program
Files/Wavefunction/Spartan20v114/P4e//../auxdir/config/preferences:
(site specific preferences)
... THRESH 9
... SMALL_PROD_XCMAT 9
... BASIS_LIN_DEP_THRESH 5
```

```
... SCF_ALGORITHM DIIS_GDM
... MAXSCF 250
... MAXDIIS 45
... THRESHDIIS 1
(i.e. don't switch on deltaE)
... ECP_FIT TRUE (Convert deprecated ECP files)
... GUI GUI_SPARTAN
... TERSE_OUTPUT TRUE !turn on spartan printing
... SCF_CONVERGENCE 7
... CCMAN2 FALSE (qc4.3)
... SYMMETRY FALSE ! turn of symmetry for spartan16
... SYM_IGNORE TRUE ! ..use FORCESYMMETRY to override
... GEOM_OPT_TOL_GRADIENT 700 ! loosen tolernaces for organic geometries
... GEOM_OPT_TOL_DISPLACEMENT 1400 ! was 1200 = .0012
... GEOM_OPT_TOL_ENERGY 2000 ! was 100 = .000 001
... GEN_SCFMAN FALSE
Processing $rem in input file
... JOBTYPE SP
... SCF_CONVERGENCE 7 (sp default for single point energy)
... METHOD WB97MV
... xc_grid 75000302 (75,302)
... BASIS 6311+
G(2DF,2P)
... THRESH 12 #diffuse default
... MAXSCF 350 #diffuse default
... VARTHRESH 2 (default DFT)
... INCDFT TRUE (default DFT)
... GUI GUI_SPARTAN
... TERSE_OUTPUT TRUE
NAlpha2: 152
NElect 152
Mult 1
Warning: disabling incdft.
Checking the input file for inconsistencies... ...done.
User
input:
$
comment
C4 TFP Cation
$end
$molecule
1 1
15 0.2213936337
0.58602010032
0.1793783717
6 1.4432519917
0.40038773648 0.99599406295
6 1.5668694734
0.85135790445 2.2742502365
6 2.7957837088
1.5761437338 2.3112135271
6 3.314903125
1.5051816203 1.0591225653
8 2.508132959
0.7928467473 0.23720501163
6 0.74291792758
2.2665380084
0.039990858695
```

```
6 0.86225710301
3.049562674
1.1494055976
6 1.3701029485
4.3011317292
0.68996820843
6 1.5208246116
4.1749306365
0.65326630504
8 1.1453191474
2.9446059322
1.0722562298
6 1.2022438233 0.62118768952
1.2337225215
8 1.6894089249 0.56367382635 1.7054342163
6 2.7763708419 0.25516237154 2.4517837207
6 2.9939581052 1.0845101443
2.4691956663
6 1.962709735 1.6635394724
1.6713435195
1 1.8014784108 2.7086887297
1.4568206002
1 3.7860027478 1.5987843897
2.9880542076
1 3.2835173287 1.0918211147 2.9032873012
1 0.86883065739
0.69109964855 3.0808828339
1 3.2301497019
2.0834885053 3.1567662372
1 4.2126392567
1.8948018817 0.60855175014
1 1.8710236407
4.8427322985
1.422891987
1 1.5945533313
5.172693716
1.2823940436
1 0.62378125357
2.7750363692
2.1653406498
6 0.10140198055 0.11318678099 1.4541557841
1 0.81471722405 0.54995486617
1.9541419581
1 0.84791486526
0.07259999759 2.0000366383
6 0.63195936228 1.5510550526 1.3734537711
1 1.5734777359 1.5691769585 0.81496980638
1 0.077319419682
2.1751344886 0.81859845172
6 0.84922519544 2.1393437568 2.7678190372
1 1.5546491901 1.5088351387 3.3202779734
1 0.095497838625
2.1113004637 3.3218804418
6 1.3719921965 3.5713302679 2.7064345538
1 2.3283966295 3.6195828605 2.1790769484
1 0.6679403522 4.2258128313 2.1857157753
1 1.5240168099 3.9765930687 3.7077603743
$end
```

```
$rem
JOBTYPE SP
SCF_CONVERGENCE 7 (sp default for single point energy)
METHOD WB97MV
xc_grid 75000302 (75,302)
BASIS 6311+
G(2DF,2P)
THRESH 12 #diffuse default
MAXSCF 350 #diffuse default
VARTHRESH 2 (default DFT)
INCDFT TRUE (default DFT)
GUI GUI_SPARTAN
TERSE_OUTPUT TRUE
$end
S
tandard Nuclear Orientation (Angstroms)
I Atom X Y Z
1
P 0.2213936337
0.5860201003
0.1793783717
2 C 1.4432519917
0.4003877365 0.9959940630
3 C 1.5668694734
0.8513579045 2.2742502365
4 C 2.7957837088
1.5761437338 2.3112135271
5 C 3.3149031250
1.5051816203 1.0591225653
6 O 2.5081329590
0.7928467473 0.2372050116
7 C 0.7429179276
2.2665380084
0.0399908587
8 C 0.8622571030
3.0495626740
1.1494055976
9 C 1.3701029485
4.3011317292
0.6899682084
10 C 1.5208246116
4.1749306365
0.6532663050
11 O 1.1453191474
2.9446059322
1.0722562298
12 C 1.2022438233 0.6211876895
1.2337225215
13 O 1.6894089249 0.5636738263 1.7054342163
14 C 2.7763708419 0.2551623715 2.4517837207
15 C 2.9939581052 1.0845101443
2.4691956663
16 C 1.9627097350 1.6635394724
1.6713435195
17 H 1.8014784108 2.7086887297
1.4568206002
18 H 3.7860027478 1.5987843897
2.9880542076
```

```
19 H 3.2835173287 1.0918211147 2.9032873012
20 H 0.8688306574
0.6910996485 3.0808828339
21 H 3.2301497019
2.0834885053 3.1567662372
22 H 4.2126392567
1.8948018817 0.6085517501
23 H 1.8710236407
4.8427322985
1.4228919870
24 H 1.5945533313
5.1726937160
1.2823940436
25 H 0.6237812536
2.7750363692
2.1653406498
26 C 0.1014019806 0.1131867810 1.4541557841
27 H 0.8147172241 0.5499548662
1.9541419581
28 H 0.8479148653
0.0725999976 2.0000366383
29 C 0.6319593623 1.5510550526 1.3734537711
30 H 1.5734777359 1.5691769585 0.8149698064
31 H 0.0773194197
2.1751344886 0.8185984517
32 C 0.8492251954 2.1393437568 2.7678190372
33 H 1.5546491901 1.5088351387 3.3202779734
34 H 0.0954978386
2.1113004637 3.3218804418
35 C 1.3719921965 3.5713302679 2.7064345538
36 H 2.3283966295 3.6195828605 2.1790769484
37 H 0.6679403522 4.2258128313 2.1857157753
38 H 1.5240168099 3.9765930687 3.7077603743
Nuclear
Repulsion Energy = 1724.19154895 hartrees
There are 76 alpha and 76 beta electrons
Requested basis set is 6311+
G(2df,2p)
There are 257 shells and 850 basis functions
Total QAlloc Memory Limit 20000 MB
MegaArray
Size 188 MB
MEM_STATIC part 192 MB
.. (5.2.P)
Entering
fldman on Sun Jan 15 11:58:35 2023 A
cutoff of 1.0D12
yielded 22687 shell pairs
There are 252159 function pairs ( 309290 Cartesian)
Smallest overlap matrix eigenvalue = 1.86E06
Linear dependence detected in AO basis
Tighter screening thresholds may be required for diffuse basis sets
Use S2THRESH > 12 and THRESH = 14 in case of SCF convergence issues
Number of orthogonalized atomic orbitals = 844
Maximum deviation from orthogonality = 2.128E11
Scale SEOQF with 1.000000e01/
1.000000e01/
1.000000e01
```

```
Standard Electronic Orientation quadrupole field applied
Nucleusfield
energy = 0.0000000206 hartrees
Entering
gesman on Sun Jan 15 11:58:37 2023 Guess
from superposition of atomic densities
Warning: Energy on first SCF cycle will be nonvariational
SAD guess density has 149.164804 electrons
Entering
scfman on Sun Jan 15 11:58:37 2023 Longrange
K will be added via erf
Coulomb attenuation parameter = 0.3 bohr**(1)
A restricted hybrid HFDFT
SCF calculation will be
performed using Pulay DIIS + Geometric Direct Minimization
Exchange: 0.1500 HartreeFock
+ 1.0000 wB97MV
+ LRHF
Correlation: 1.0000 wB97MV
Using EulerMaclaurinLebedev
(75,302) quadrature formula
Nonlocal Correlation: VV10 with C = 0.0100 and b = 6.00 and scale = 1.00000
Grid used for NLC: SG1
standard quadrature
SCF converges when RMS gradient is below 1.0E07
Exchange: 0.1500 HartreeFock
+ 1.0000 wB97MV
+ LRHF
Correlation: 1.0000 wB97MV
Using EulerMaclaurinLebedev
(75,302) quadrature formula
Nonlocal Correlation: VV10 with C = 0.0100 and b = 6.00 and scale = 1.00000
Grid used for NLC: SG1
standard quadrature
using 4 threads for integral computing
OpenMP
Integral computing Module
Release: version 1.0, May 2013, QChem
Inc. Pittsburgh
using
4 threads for integral computing
OpenMP
Integral computing Module
Release: version 1.0, May 2013, QChem
Inc. Pittsburgh
OpenMP
BLAS3 based DFT computing Module
Release: version 1.0, May 2013, QChem
Inc. Pittsburgh
OpenMP
BLAS3 based DFT computing Module
Release: version 1.0, May 2013, QChem
Inc. Pittsburgh
OpenMP
BLAS3 based DFT computing Module
Release: version 1.0, May 2013, QChem
Inc. Pittsburgh
Cycle
```

Energy DIIS Error
1
1185.0750979574
5.85E02
2 1087.5585583394
3.43E02
3 631.4619141890
4.99E02
4 1062.7857579073
2.28E02
5 439.8185113494
7.29E02
6 766.4977703695
5.21E02
7 917.9416654122
4.18E02
8 1052.0248663097
2.90E02
9 1184.5963588150
5.32E03
10 1180.9319088413
6.19E03
11 1186.9898032299
1.50E03
12 1187.1953617505
6.18E04
13 1187.2281445833
2.65E04
14 1187.2340157814
1.08E04
15 1187.2352691271
5.27E05
16 1187.2355565906
1.34E05
17 1187.2355850194
6.48E06
18 1187.2355903840
3.02E06
19 1187.2355921718
1.30E06
20 1187.2355923981
4.65E07
21 1187.2355924310
1.23E07
22 1187.2355924328
5.64E08
Convergence criterion met
SCF
time: CPU 14213.81 s wall 3578.99 s
SCF energy in the final basis set = 1187.23559243
Total energy in the final basis set = 1187.23559243
Entering
anlman on Sun Jan 15 12:58:16 2023 Orbital
Energies (a.u.)
Alpha
MOs
Occupied
77.5994

19.5471
19.5465
19.5435
10.5451
10.5449
10.5398
10.5356
10.5355
10.5320
10.5034
10.4994
10.4978
10.4937
10.4763
10.4762
10.4724
10.4704
10.4455
10.4207
6.9481
5.0606
5.0602
5.0596
1.3908
1.3898
1.3864
1.1000
1.0719
1.0704
1.0663
1.0416
1.0113
1.0090
0.9810
0.9230
0.8760
0.8614
0.8548
0.8516
0.8226
0.8187
0.8129
0.8038
0.7933
0.7916
0.7414
0.7245
0.7195
0.7160
0.7063
0.6870
0.6784
0.6681
0.6581
0.6537
0.6486
0.6447
0.6412

0.6364
0.6335
0.6309
0.6240
0.6175
0.6115
0.5930
0.5793
0.5509
0.5383
0.5338
0.5251
0.5216
0.5178
0.4779
0.4708
0.4614
Virtual
0.1017
0.0876
0.0784
0.0723
0.0615
0.0601
0.0550
0.0407
0.0386
0.0371
0.0331
0.0316
0.0272
0.0234
0.0181
0.0170
0.0135
0.0117
0.0110
0.0075
0.0034
0.0031
0.0017 0.0041
0.0085 0.0127 0.0190 0.0241 0.0261 0.0281 0.0312 0.0352
0.0366 0.0400 0.0437 0.0465 0.0517 0.0544 0.0607 0.0639
0.0649 0.0674 0.0701 0.0775 0.0796 0.0826 0.0840 0.0852
0.0908 0.0939 0.0967 0.0976 0.1026 0.1046 0.1063 0.1102
0.1126 0.1186 0.1254 0.1255 0.1322 0.1351 0.1354 0.1435
0.1474 0.1488 0.1522 0.1546 0.1580 0.1594 0.1609 0.1648
0.1681 0.1695 0.1748 0.1760 0.1776 0.1792 0.1829 0.1852
0.1859 0.1913 0.1961 0.2002 0.2030 0.2069 0.2089 0.2146
0.2170 0.2184 0.2224 0.2315 0.2354 0.2363 0.2431 0.2504
0.2551 0.2611 0.2669 0.2716 0.2766 0.2861 0.2938 0.2980
0.3001 0.3086 0.3157 0.3166 0.3212 0.3254 0.3345 0.3384
0.3525 0.3540 0.3643 0.3714 0.3778 0.3798 0.3871 0.3914
0.3952 0.3982 0.4081 0.4091 0.4163 0.4212 0.4258 0.4367
0.4383 0.4474 0.4528 0.4590 0.4638 0.4681 0.4740 0.4784
0.4809 0.4826 0.4925 0.4967 0.4979 0.5061 0.5121 0.5137
0.5215 0.5274 0.5348 0.5400 0.5435 0.5482 0.5504 0.5549
0.5567 0.5588 0.5669 0.5684 0.5701 0.5737 0.5773 0.5826

0.5857	0.5906	0.5920	0.5962	0.6001	0.6039	0.6100	0.6139
0.6162	0.6192	0.6199	0.6260	0.6276	0.6317	0.6378	0.6399
0.6421	0.6486	0.6522	0.6568	0.6570	0.6580	0.6607	0.6657
0.6705	0.6766	0.6798	0.6849	0.6893	0.6935	0.6999	0.7083
0.7124	0.7170	0.7274	0.7302	0.7325	0.7476	0.7512	0.7617
0.7648	0.7707	0.7789	0.7861	0.7881	0.7932	0.8013	0.8055
0.8080	0.8171	0.8254	0.8305	0.8354	0.8446	0.8466	0.8523
0.8543	0.8598	0.8658	0.8792	0.8812	0.8845	0.8869	0.8982
0.8997	0.9181	0.9201	0.9237	0.9312	0.9403	0.9532	0.9607
0.9688	0.9803	0.9817	0.9836	0.9878	0.9935	1.0010	1.0066
1.0145	1.0193	1.0252	1.0319	1.0405	1.0434	1.0543	1.0594
1.0677	1.0749	1.0783	1.0862	1.0915	1.0941	1.1047	1.1117
1.1192	1.1226	1.1282	1.1308	1.1393	1.1435	1.1460	1.1565
1.1592	1.1636	1.1713	1.1754	1.1760	1.1795	1.1811	1.1914
1.2009	1.2083	1.2131	1.2148	1.2229	1.2272	1.2322	1.2327
1.2393	1.2449	1.2483	1.2536	1.2613	1.2710	1.2759	1.2778
1.2882	1.2909	1.3006	1.3070	1.3083	1.3229	1.3316	1.3418
1.3509	1.3569	1.3640	1.3689	1.3703	1.3749	1.3768	1.3850
1.3919	1.3978	1.4022	1.4083	1.4128	1.4212	1.4337	1.4349
1.4420	1.4475	1.4526	1.4559	1.4768	1.4869	1.4911	1.5058
1.5129	1.5149	1.5205	1.5315	1.5493	1.5550	1.5613	1.5676
1.5778	1.5813	1.5983	1.6072	1.6155	1.6246	1.6390	1.6511
1.6633	1.6692	1.6763	1.6819	1.6874	1.6961	1.7132	1.7169
1.7244	1.7297	1.7434	1.7467	1.7579	1.7624	1.7648	1.7732
1.7792	1.7898	1.8032	1.8144	1.8289	1.8487	1.8662	1.8751
1.9033	1.9096	1.9236	1.9454	1.9741	1.9841	2.0218	2.0376
2.0618	2.0782	2.1053	2.1850	2.2055	2.2580	2.2723	2.2823
2.2964	2.3102	2.3134	2.3271	2.3334	2.3621	2.3834	2.4071
2.4185	2.4571	2.4647	2.4673	2.4686	2.4878	2.4995	2.5238
2.5338	2.5466	2.5520	2.5563	2.5808	2.5898	2.5982	2.6246
2.6325	2.6426	2.6457	2.6580	2.6611	2.6711	2.6824	2.6923
2.7019	2.7103	2.7136	2.7143	2.7243	2.7288	2.7301	2.7351
2.7520	2.7600	2.7606	2.7645	2.7680	2.7724	2.7796	2.7870
2.7914	2.7981	2.8012	2.8141	2.8180	2.8262	2.8307	2.8397
2.8462	2.8498	2.8580	2.8657	2.8694	2.8779	2.8910	2.9011
2.9086	2.9107	2.9189	2.9221	2.9311	2.9419	2.9458	2.9512
2.9600	2.9633	2.9732	2.9783	2.9865	2.9913	2.9975	3.0102
3.0210	3.0288	3.0351	3.0588	3.0682	3.0711	3.0809	3.0969
3.1095	3.1096	3.1157	3.1224	3.1269	3.1342	3.1399	3.1491
3.1543	3.1583	3.1659	3.1688	3.1762	3.1799	3.1812	3.1914
3.1952	3.2003	3.2041	3.2077	3.2142	3.2206	3.2319	3.2322
3.2421	3.2452	3.2488	3.2513	3.2556	3.2666	3.2745	3.2844
3.2943	3.3024	3.3065	3.3162	3.3173	3.3249	3.3299	3.3313
3.3370	3.3381	3.3439	3.3504	3.3576	3.3595	3.3666	3.3727
3.3756	3.3813	3.3877	3.3917	3.3981	3.4019	3.4118	3.4183
3.4235	3.4268	3.4320	3.4457	3.4591	3.4655	3.4729	3.4821
3.4912	3.5064	3.5151	3.5284	3.5321	3.5379	3.5438	3.5506
3.5557	3.5598	3.5670	3.5773	3.5790	3.5886	3.5940	3.5976
3.6047	3.6111	3.6175	3.6229	3.6244	3.6279	3.6320	3.6393
3.6424	3.6490	3.6539	3.6614	3.6685	3.6728	3.6946	3.7008
3.7102	3.7129	3.7235	3.7302	3.7431	3.7459	3.7467	3.7531
3.7590	3.7736	3.7808	3.7929	3.7972	3.8156	3.8228	3.8304
3.8351	3.8469	3.8603	3.8683	3.8865	3.9022	3.9284	3.9375
3.9397	3.9489	3.9598	3.9646	3.9657	3.9758	3.9782	3.9954
4.0024	4.0049	4.0135	4.0164	4.0314	4.0576	4.0649	4.0729
4.0750	4.0978	4.0987	4.1042	4.1103	4.1172	4.1208	4.1306
4.1389	4.1408	4.1717	4.1777	4.1941	4.2009	4.2077	4.2108
4.2210	4.2245	4.2287	4.2375	4.2407	4.2545	4.2903	4.3002

4.3061 4.3259 4.3525 4.3531 4.3575 4.3718 4.3813 4.4415
4.4555 4.4618 4.4651 4.4737 4.4781 4.4891 4.4959 4.4973
4.5132 4.5169 4.5244 4.5256 4.5330 4.5415 4.5443 4.5547
4.5760 4.5867 4.5957 4.6216 4.6264 4.6300 4.6326 4.6380
4.6738 4.6832 4.6906 4.7221 4.7300 4.7360 4.7474 4.8104
4.8386 4.9185 4.9355 4.9412 4.9697 4.9747 4.9824 5.0537
5.0764 5.0808 5.0910 5.1132 5.1309 5.1442 5.1709 5.1915
5.2031 5.2597 5.2938 5.3116 5.3141 5.3179 5.3329 5.3359
5.3438 5.3491 5.3559 5.3963 5.4339 5.5027 5.5040 5.5099
5.5229 5.5386 5.5718 5.5928 5.6317 5.6397 5.6617 5.6693
5.6776 5.7093 5.7107 5.7204 5.7321 5.7357 5.7411 5.7816
5.7875 5.7892 5.9192 5.9384 5.9539 6.2922 6.2949 6.2999
6.4378 6.4528 6.4560 6.5651 6.5722 6.5803 6.8664 6.8820
6.8871 7.1202 7.1244 7.1341 7.2002 7.2019 7.2129 7.3927
7.3992 7.4111 7.7228 7.7562 7.7636 7.7706 7.7985 7.8033
8.1232 14.5939 14.7020 14.7524 24.2874 24.2957 24.3027 24.3984
24.5112 24.5854 24.5975 24.6085 24.6250 24.6513 24.9425 24.9487
24.9588 25.1440 25.1518 25.1548 50.5299 50.5497 50.5633 165.2243
GroundState
Mulliken Net Atomic Charges
Atom Charge (a.u.)
1
P 0.158628
2 C 0.110122
3 C 0.218004
4 C 0.284937
5 C 0.045995
6 O 0.028730
7 C 0.129012
8 C 0.173916
9 C 0.297272
10 C 0.016855
11 O 0.001134
12 C 0.111466
13 O 0.002971
14 C 0.013672
15 C 0.275693
16 C 0.176636
17 H 0.213111
18 H 0.183006
19 H 0.207154
20 H 0.210312
21 H 0.181009
22 H 0.203367
23 H 0.205780
24 H 0.183175
25 H 0.200308
26 C 0.444482
27 H 0.215090
28 H 0.250533
29 C 0.414717
30 H 0.215500
31 H 0.213291
32 C 0.273140
33 H 0.172519
34 H 0.174350
35 C 0.573583
36 H 0.157378

37 H 0.158386
38 H 0.169528
Sum
of atomic charges = 1.000000
Cartesian
Multipole Moments
Charge
(ESU x 10^10)
4.8032
Dipole Moment (Debye)
X 0.6986
Y 3.2003
Z 1.9987
Tot 3.8373
Quadrupole Moments (DebyeAng)
XX 96.0247
XY 0.0646
YY 86.9634
XZ 3.5908 YZ 1.1347 ZZ 98.2589
Traceless Quadrupole Moments (DebyeAng)
QXX 6.8270
QYY 20.3567 QZZ 13.5297
QXY 0.1939
QXZ 10.7723 QYZ 3.4041
Octopole Moments (DebyeAng^
2)
XXX 46.2821 XXY 55.9721 XYY 26.4035
YYY 12.8128
XXZ 21.7997 XYZ 20.8577
YYZ 35.8243
XZZ 35.6427 YZZ 44.9439
ZZZ 88.7000
Traceless Octopole Moments (DebyeAng^
2)
XXX 194.5407 YYY 985.1214
ZZZ 405.9781
XXY 575.2725 XXZ 635.1690 XYY 562.6170
XYZ 312.8648
XZZ 368.0763 YYZ 229.1910
YZZ 409.8489
Hexadecapole Moments (DebyeAng^
3)
XXXX 2087.0940
XXXY 323.9227
XXYY 818.2227
XYYY 44.1931 YYYY 2815.9097
XXXZ 123.1684
XXYZ 116.1672 XYYZ 5.0661
YYYZ 251.9248
XXZZ 601.0762
XYZZ 70.7877
YYZZ 805.9150
XZZZ 61.1607 YZZZ 335.6590 ZZZZ 2053.0367
Traceless Hexadecapole Moments (DebyeAng^
3)
XXXX 6227.7280
XXXY 18238.6032
XXXZ 4865.8438

XXYY 936.1795
XXYZ 1651.1782 XXZZ 7163.9074
XYYY 20413.5569 XYYZ 3220.8831
XYZZ 2174.9538
XZZZ 1644.9607
YYYY 1275.5339 YYYZ 5187.0352
YYZZ 339.3544
YZZZ 3535.8570 ZZZZ 6824.5530
Total
job time: 3582.92s(wall), 14218.86s(cpu)
Sun Jan 15 12:58:17 2023
